# Metal (n+1)p‐nd Orbital Hybridization and Excited‐State Metal–Ligand π‐Interactions Enable d^10^ Carbene‐Metal‐Amide TADF OLEDs with High Efficiency and Long Operational Lifetime

**DOI:** 10.1002/advs.202600075

**Published:** 2026-06-29

**Authors:** Shuo Xu, Rui Tang, Qingyun Wan, Gang Cheng, Jun Yang, Chi‐Ming Che

**Affiliations:** ^1^ Department of Chemistry State Key Laboratory of Synthetic Chemistry CAS‐HKU Joint Laboratory on New Materials The University of Hong Kong Hong Kong P. R. China; ^2^ Department of Chemistry, Shanghai Hong Kong Joint Laboratory in Chemical Synthesis The Chinese University of Hong Kong Shatin Hong Kong P. R. China; ^3^ Hong Kong Quantum AI Lab Limited Units 909‐915, Building 17W, Science Park West Avenue, Hong Kong Science Park, Pak Shek Kok Hong Kong P. R. China; ^4^ HKU Shenzhen Institute of Research & Innovation Shenzhen Guangdong P. R. China

**Keywords:** bond theory, carbene‐metal‐amides, organic light‐emitting diodes, photophysics, thermally activated delayed fluorescence

## Abstract

Luminescent d^10^ carbene‐metal‐amide (CMA) complexes are a promising class of thermally activated delayed fluorescence (TADF) organic light‐emitting diode (OLED) emitters. However, the principles for modifying ligands to maximize OLED efficiency and operational stability remain unclear. Here, we reveal the key role of metal (n+1)p‐nd orbital hybridization and excited‐state metal‐ligand π‐interactions in affecting the excited‐state stability and electro‐/photoluminescence efficiency of CMA emitters. Using density functional theory (DFT), high‐level coupled cluster singles and doubles (CCSD) method, and combined DFT and multireference configuration interaction (DFT/MRCI) calculations, we found that in the excited state, metal atoms and carbazole nitrogen atoms form π‐interactions, which is weakened by the weakening of metal (n+1)p‐nd orbital hybridization. The weakened metal–nitrogen (M─N) π‐interaction is conducive to more flexible rotation of the excited‐state dihedral angle, thereby increasing the radiative decay rate (*k*_TADF_). This rationalizes the general trend of *k*_TADF_ for CMA emitters: Ag > Au > Cu. However, the weakening of the excited‐state M─N π‐interaction reduces the strength of the M─N bond and facilitates bond dissociation in the excited state, thereby impairing the stability of the emitter. Our calculations show that introducing electron‐withdrawing or π‐extended substituents on carbazole ligands reduces excited‐state M─N π‐interactions, thereby improving *k*_TADF,_ but may impair emitter stability and device operational lifetime.

## Graphical abstract

We elucidate the key roles of metal p‐d orbital hybridization, excited‐state metal–ligand π‐interactions, and structural rotation in TADF of d^10^ CMA complexes. Introducing electron‐withdrawing or π‐extended ligand substituents weakens excited‐state metal‐ligand π‐interactions and improves *k*_TADF_, but may compromise emitter stability in device operation. These insights guide the design of CMA TADF OLEDs with high efficiency and long operational lifetime.

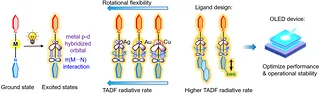

## 1 Introduction

Luminescent d^10^ metal complexes exhibit diverse excited‐state and photophysical properties. Of particular interest are the coinage d^10^ (Au(I), Ag(I), and Cu(I)) carbene‐metal‐amide (CMA) complexes first reported in 2017 by Credgington and coworkers [1]. CMA complexes have emerged as a promising class of thermally activated delayed fluorescence (TADF) emitters with high radiative decay rate (*k*_TADF_), easy synthesis, tunable emission colors, and high internal and external quantum efficiencies [2, 3, 4, 5, 6, 7, 8, 9, 10, 11, 12, 13, 14, 15, 16, 17, 18, 19, 20, 21, 22, 23, 24]. Despite these advantages, practical organic light‐emitting diode (OLED) devices using d^10^ CMA emitters with prolonged operational lifetimes remain extremely rare. Recently, we reported a class of Au(I) and Cu(I) CMA emitters with sterically bulky π‐conjugated *N*‐heterocyclic carbene ligands that exhibit high radiative decay rates, enabling OLEDs fabricated using these emitters to have ultrahigh luminance and significantly extended device operational lifetimes (LT) (LT_95_ up to 2082 h for Au(I) emitters and 3582 h for Cu(I) emitters at 1000 cd m^−2^) [25, 26, 27]. It appears that subtle changes in the electronic structure of auxiliary ligands, such as *N*‐heterocyclic carbene ligands, can significantly improve the operational stability of OLEDs fabricated using Cu(I)‐ and Au(I)‐CMA emitters. Extensive theoretical studies have been reported in the literature to elucidate the emission mechanisms and establish rational design principles for d^10^ CMA emitters [11, 21, 28, 29, 30, 31, 32, 33, 34, 35, 36, 37, 38, 39, 40, 41], though no consensus has been reached yet. Moreover, due to the high computational cost, high‐level/high‐precision methods beyond density functional theory (DFT) are rarely used for excited‐state calculations of CMA emitters [13, 28, 37].

A high TADF radiative decay rate of the emitter is advantageous because it reduces efficiency roll‐off at high brightness levels and improves overall OLED device stability by minimizing excited‐state saturation effects and chemical reactions in the device [42, 43]. Currently, two main strategies can be used to achieve high radiative decay rates in donor–acceptor‐type TADF emitters: 1) reducing the singlet‐triplet energy gap (*∆E*_S1‐T1_), which is conducive to efficient intersystem crossing (ISC) and reverse intersystem crossing (*r*ISC) processes; and 2) increasing the S_1_→S_0_ radiative decay rate (*k*_S1_) [44, 45]. Smaller *∆E*_S1‐T1_ values are usually achieved by reducing the wavefunction overlap between the highest occupied molecular orbital (HOMO) and the lowest unoccupied molecular orbital (LUMO) (i.e., donor and acceptor ligands). However, this approach has the drawback of reducing the oscillator strength of the S_1_→S_0_ transition (*f*_S1→S0_), thereby sacrificing *k*_S1_. This contradiction makes it challenging to simultaneously achieve a small *∆E*_S1‐T1_ and a large *k*_S1_, thus a high *k*_TADF_.

For d^10^ CMA emitters, much effort has been devoted to modulating excited‐state conformational changes to achieve high TADF radiative decay rates. This can be achieved by introducing sterically bulky substituents on the ligands to restrict the molecular rotation and confine the molecule in an orthogonal conformation, thereby obtaining a smaller *∆E*_S1‐T1_. However, this approach may lead to a decrease in *k*_S1_ [46]_._ Alternatively, the twist conformations can be constrained between coplanar and orthogonal geometries with specific dihedral angles to achieve a balance between *∆E*_S1‐T1_ and *k*_S1_ [21, 47]. Some studies have also emphasized the importance of the coplanar geometry in enhancing the TADF radiative decay rate of CMA emitters [13, 28]. Previous experimental studies have shown that the *k*_TADF_ values of d^10^ CMA emitters follow a general trend of Ag(I) > Au(I) > Cu(I) (Table 1) [5, 9, 20, 25, 26]. To the best of our knowledge, the mechanism behind this trend and the role of metal atoms in d^10^ CMA TADF emission remain unanswered. We believe a deeper understanding of the role of metal atoms, excited‐state metal–ligand (M─L) interactions, and excited‐state molecular rotation can pave the way for the better design of high‐performance d^10^ CMA emitters.

**TABLE 1:** Experimental *k*_TADF_ for emitters **M‐1, M‐2**, **M‐3**, **M‐5**, and **M‐6**, and computational *k*_TADF_ for emitters **M‐1**, **M‐2**, and **M‐3** calculated in this work.

		*k*_TADF_ [10^5^ s^−1^]		
		M = Au	M = Ag	M = Cu
**M‐1** [25, 26]	Expt. (2 wt.% mCP^c^ film)	24.1^a^	31.3^a)^, ^b)^	19.0
	Cal. (Toluene solution)	3.83	4.75	1.33

**M‐2** [9]	Expt.(Toluene solution)	9.1	16.4	3.0
	Expt. (MCH^d^ solution)	6.9	12.6	1.2
	Expt. (1 wt.% Zeonex matrix)	7.7	9.3	1.2
	Expt. (1 wt.% PS^e^ matrix)	7.6	11.8	1.0
	Cal. (Toluene solution)	5.10	9.36	1.11

**M‐3** [5]	Expt. (2‐MeTHF^f^ solution)	6.3	15	5.0
	Expt. (MCH^d^ solution)	8.0	18	5.6
	Expt. (1 wt.% PS^e^ matrix)	10	24	6.4
	Cal. (Toluene solution)	3.48	3.81	0.75

**M‐5** [5]^g^	Expt. (2‐MeTHF^f^ solution)	7.9	19	3.9
	Expt. (MCH^d^ solution)	8.2	20	4.0
	Expt. (1 wt.% PS^e^ matrix)	8.8	20	3.5

**M‐6^MeCN^** [20]^g^	Expt. (MCH^d^ solution)	6.9	16	4.2
	Expt. (Toluene solution)	8.2	19	6.5
	Expt. (2wt.% PS^e^ film)	6.2	20	5.5

**M‐6^PhCN^** [20]^g^	Expt. (MCH^d^ solution)	8.9	9.0	6.6
	Expt. (Toluene solution)	11	9.5	7.7
	Expt. (2wt.% PS^e^ film)	12	15	7.5

**M‐6^Me^** [20]^g^	Expt. (MCH^d^ solution)	5.1	10	4.1
	Expt. Toluene solution)	5.4	13	4.4
	Expt. (2wt.% PS^e^ film)	4.5	19	3.9

**M‐6^Ph^** [20]^g^	Expt. (MCH^d^ solution)	7.5	8.1	5.7
	Expt. (Toluene solution)	7.9	5.3	5.9
	Expt. (2wt.% PS^e^ film)	9.6	11	7.2

^a)^ Detailed photophysical measurements are shown in Section S1.6 and Table S4; Figures S15 and S16.

^b)^ Synthesis and characterization details are shown in Section S1.3.

^c)^ 1,3‐bis(*N*‐carbazolyl)benzene (mCP).

^d)^ methylcyclohexane (MCH).

^e)^ polystyrene (PS).

^f)^ 2‐methyltetrahydrofuran (2‐MeTHF).

^g)^ Molecular structures are shown in Figure S1.

**FIGURE 1:**
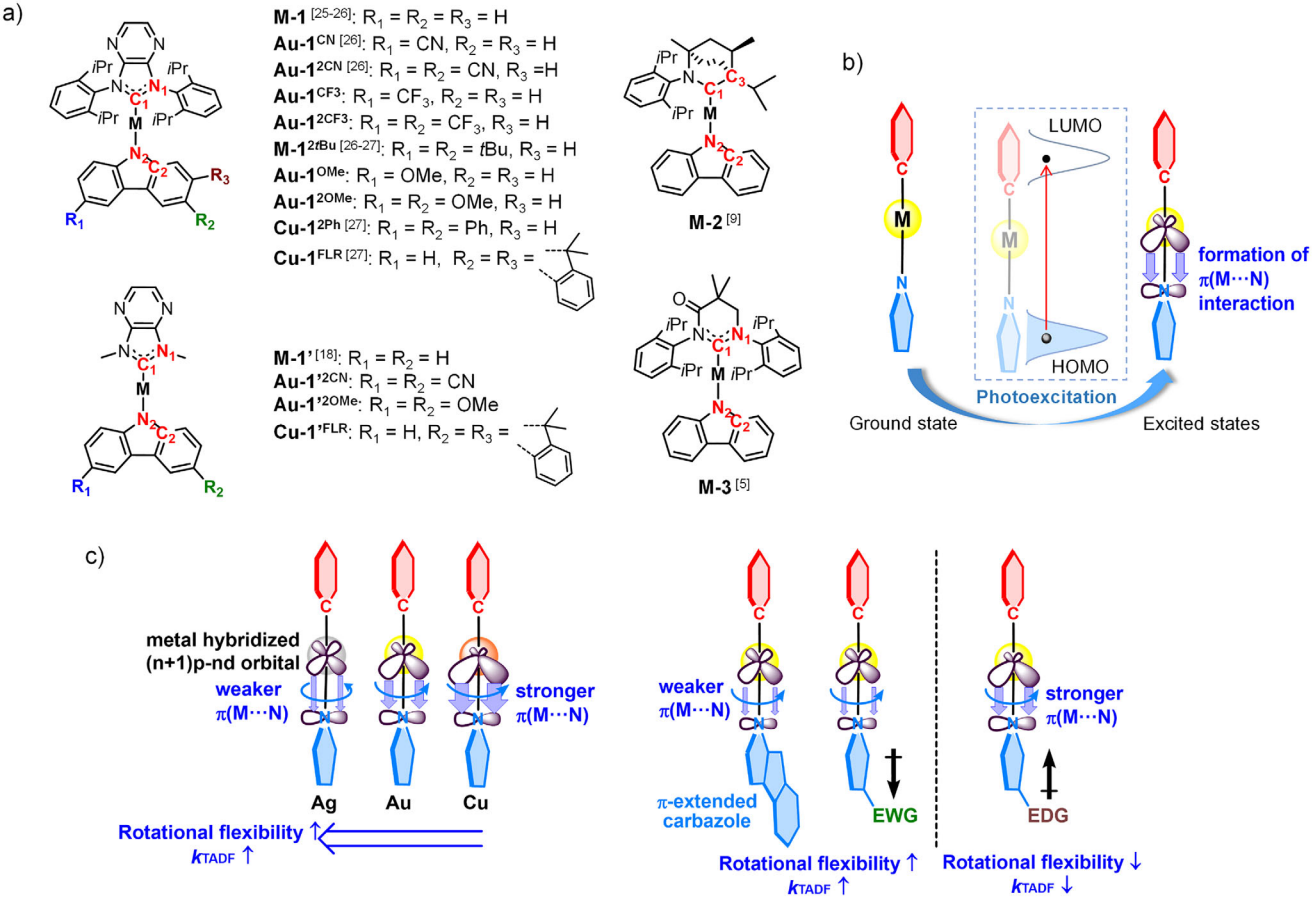
(a) Molecular structures of selected CMA TADF emitters. M = Ag(I), Au(I), and Cu(I). (b) Schematic diagram showing the formation of the metal‐nitrogen (M─N) π‐interactions in the excited states of emitters. (c) Schematic diagrams showing the impact of metal (n+1)p‐nd orbital hybridization, ligand structure, excited‐state M─N π‐interactions, and rotational flexibility on the TADF radiative decay rate of emitters. EWG and EDG represent electron‐withdrawing and electron‐donating substituents, respectively.

In this work, we utilized excited‐state analysis based on time‐dependent density functional theory (TDDFT), high‐level and high‐accuracy ab initio wave‐function theory, and a variety of bonding analysis methods to explore the following topics: 1) the role of metal and orbital hybridization in excited‐state M─L interactions; 2) the impact of excited‐state M─L interactions on the TADF radiative decay rate; 3) ligand design principles for d^10^ CMA TADF emitters aimed at achieving better OLED device performance (Figure 1).

## 2 Results and Discussion

### 2.1 Emitter Structures and Metal–Ligand Bond Strength

Among all the CMA complexes studied in this work, **Ag‐1**, **Au‐1^2CF3^**, **Au‐1^2^ *^t^* ^Bu^**, and **Au‐1^2OMe^** are newly synthesized, **Au‐1^CF3^** and **Au‐1^OMe^** are hypothetical structures, and **Ag‐1’**, **Cu‐1’**, **Au‐1’^2CN^**, **Au‐1’^2OMe^**, and **Cu‐1’^FLR^** are simplified structures (Figure 1a). All other complexes have been reported in the literature [5, 8, 9, 18, 20, 25, 26, 27, 36, 47, 48]. The solid‐state structures of complexes **Au‐1^2CF3^** and **Au‐1^2^ *^t^* ^Bu^** were determined by single‐crystal X‐ray diffraction (Figure S13 and Table S2). First, the structures of **M‐1**, **M‐2**, and **M‐3** (M = Au, Ag, Cu) were optimized using the validated functional PBE0 [49, 50] with the D3 version of dispersion correction using Becke–Johnson damping (D3BJ) [51, 52] (see Sections S2.1.1 and S2.1.2 for details of geometry optimization and functional validation). The optimized ground‐state structures adopt semi‐coplanar geometries (Figure S19), which are in agreement with the experimentally determined crystal structures (Table S6). In the S_1_ excited state, these complexes (except **Au‐2**) adopt two optimized geometries with semi‐coplanar or orthogonal orientation between carbazole and carbene ligands (Figure S19). This indicates that the CMA emitters can undergo flexible dihedral angle rotation along the carbon‐metal‐nitrogen (C─M─N) bond in the excited state.

Dihedral angle rotations of carbene and carbazole ligands are expected to occur at the metal–carbon (M─C) bond connecting the metal atom to the carbene, or at the metal–nitrogen (M─N) bond connecting the metal atom to the carbazole. We applied DFT calculations to calculate the bond order and bond length to investigate the bond strengths of M─C and M─N bonds in these CMA complexes (M = Au, Ag, and Cu). For all optimized structures of **M‐1**, **M‐2**, and **M‐3** in the ground state (S_0_) and excited states (S_1_ and T_1_), the calculated bond orders follow the trend M─C > M─N (Figure S21). Both the X‐ray crystal structures and optimized structures exhibit M─C and M─N bond lengths in a consistent order of Ag > Au > Cu (Figure S20). Within each set of complexes, the calculated M─N bond order increases in the order of Ag─N < Au─N < Cu─N (Figure S21). Therefore, the M─N bond is weaker than the M─C bond, with the Ag─N bond being the weakest and the Cu─N bond being the strongest among emitters.

It is noteworthy that σ‐bonds allow free rotation while π‐bonds restrict bond rotation. Therefore, the dihedral angle rotation of ligands in the excited state depends mainly on the strength of the M─L π‐interactions rather than the strength of the M─L σ‐bond. Compared to carbazole ligands, carbene ligands are considered to be stronger π‐acceptors because their vacant p orbital on the carbon atom can accept electron density from the filled d_π_ orbital of the metal atom. *N*‐heterocyclic carbenes (NHCs) with π‐extended substituents can further stabilize the vacant p orbital on the carbon atom, thereby showing stronger π‐acceptor ability. Therefore, M─C π‐interactions are expected to be stronger than M─N π‐interactions .

### 2.2 Metal Orbital Hybridization and Excited‐State Metal‐Ligand π‐Interactions

**FIGURE 2:**
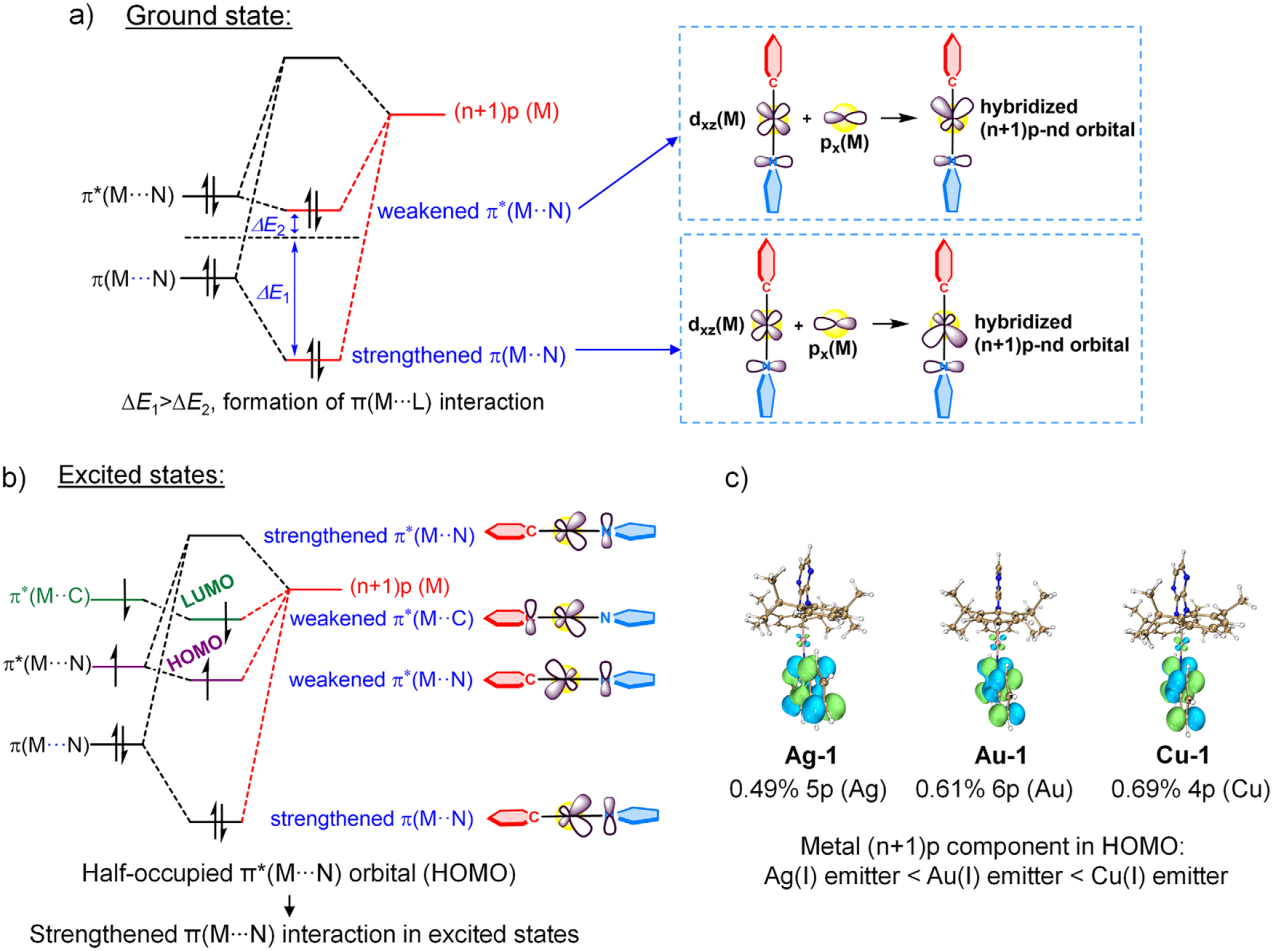
(a) Ground‐state interaction diagram of the π(M…L) orbitals by including metal (n+1)p‐nd orbital hybridization (left), and schematic diagrams showing hybridization of the metal nd orbital with the (n+1)p orbital leading to the formation of a strengthened π(M…L) bonding interaction and a weakened π^*^(M…L) anti‐bonding interaction in d^10^ metal complexes (right). (b) Excited‐state interaction diagram of π(M…L) orbitals by including (n+1)p‐nd orbital hybridization in d^10^ metal complexes. (c) Calculated HOMOs of emitters **M‐1** (M = Au, Ag, Cu) and the corresponding metal (n+1)p orbital components.

In d^10^ metal complexes, metal (n+1)s/(n+1)p‐nd orbital hybridization is crucial for M─L bonding interactions [53]. After hybridization with the (n+1)p orbital, the spatial distribution of the nd_xz_ orbitals becomes polarized, where the bonding lobe is enlarged in one direction while the antibonding lobe is suppressed in the opposite direction. This metal (n+1)p‐nd hybridization enhances the bonding character of the π(M…L) orbital and suppresses the anti‐bonding character of the π^*^(M…L) orbital, leading to the formation of M─L π‐interactions (Figure 2a) [53]. Similarly, hybridization between the metal (n+1)s and nd_z_^2^ orbitals leads to M─L σ‐bonds (Figure S30). Therefore, d‐s and d‐p orbital hybridizations are crucial to the formation of M─L σ‐bonds and π‐interactions, respectively, with greater degrees of d‐s/d‐p hybridization leading to stronger M─L σ‐bonds/π‐interactions. The calculated electronic configurations of metal atoms in d^10^ CMA emitters show that metal atoms have both (n+1)s and (n+1)p orbital components. The (n+1)p orbital of metal atoms has substantial electron populations, with 0.093–0.142 e^−^ in the ground state and 0.074–0.135 e^−^ in the excited states. The amount of the (n+1)p orbital component follows the trend of Ag(I) emitter < Au(I) emitter < Cu(I) emitter (Table 2). Therefore, M─L σ‐bonds and π‐interactions are formed simultaneously, among which the M─L π‐interaction of the Ag(I) emitter is the weakest and that of the Cu(I) emitter is the strongest.

**TABLE 2:** Calculated metal electronic configurations in CMA emitters **M‐1**, **M‐2**, and **M‐3**.

	Optimized structures	Calculated metal electronic configuration		
		M = Au	M = Ag	M = Cu
**M‐1**	S_0_ semi‐coplanar	[Xe] 5d^9.572^6s^0.956^6p^0.100^	[Kr] 4d^9.760^5s^0.639^5p^0.093^	[Ar] 3d^9.748^4s^0.595^4p^0.116^
	S_1_ semi‐coplanar	[Xe] 5d^9.591^6s^0.943^6p^0.091^	[Kr] 4d^9.779^5s^0.609^5p^0.079^	[Ar] 3d^9.759^4s^0.586^4p^0.103^
	S_1_ orthogonal	[Xe] 5d^9.590^6s^0.948^6p^0.079^	[Kr] 4d^9.774^5s^0.620^5p^0.074^	[Ar] 3d^9.758^4s^0.592^4p^0.085^
	T_1_ semi‐coplanar	[Xe] 5d^9.583^6s^0.945^6p^0.087^	[Kr] 4d^9.772^5s^0.617^5p^0.080^	[Ar] 3d^9.759^4s^0.586^4p^0.103^

**M‐2**	S_0_ semi‐coplanar	[Xe] 5d^9.576^6s^0.963^6p^0.105^	[Kr] 4d^9.765^5s^0.647^5p^0.104^	[Ar] 3d^9.746^4s^0.604^4p^0.126^
	S_1_ semi‐coplanar	[Xe] 5d^9.594^6s^0.938^6p^0.101^	[Kr] 4d^9.767^5s^0.611^5p^0.098^	[Ar] 3d^9.753^4s^0.585^4p^0.120^
	S_1_ orthogonal	[Xe] 5d^9.586^6s^0.950^6p^0.096^	[Kr] 4d^9.770^5s^0.624^5p^0.095^	[Ar] 3d^9.750^4s^0.591^4p^0.116^
	T_1_ semi‐coplanar	[Xe] 5d^9.574^6s^0.949^6p^0.102^	[Kr] 4d^9.764^5s^0.625^5p^0.100^	[Ar] 3d^9.739^4s^0.593^4p^0.121^

**M‐3**	S_0_ semi‐coplanar	[Xe] 5d^9.575^6s^0.954^6p^0.121^	[Kr] 4d^9.776^5s^0.626^5p^0.113^	[Ar] 3d^9.750^4s^0.582^4p^0.142^
	S_1_ semi‐coplanar	[Xe] 5d^9.587^6s^0.929^5p^0.119^	[Kr] 4d^9.775^5s^0.592^5p^0.109^	[Ar] 3d^9.753^4s^0.565^4p^0.135^
	S_1_ orthogonal	[Xe] 5d^9.583^6s^0.935^6p^0.110^	[Kr] 4d^9.771^5s^0.599^5p^0.102^	[Ar] 3d^9.750^4s^0.572^4p^0.123^
	T_1_ semi‐coplanar	[Xe] 5d^9.570^6s^0.935^6p^0.120^	[Kr] 4d^9.763^5s^0.603^5p^0.111^	[Ar] 3d^9.738^4s^0.574^4p^0.135^

The photoexcitation of electrons from HOMO to LUMO gives rise to S_0_→S_1_ ligand‐to‐ligand charge–transfer (^1^LLCT) transition, which makes the HOMO (i.e., anti‐bonding π*(M…N) orbital) half‐occupied. This enhances the formation of π(M…N) interaction in the excited state (Figures 1b and 2b). The calculated HOMO composition reveals that the metal (n+1)p orbital composition is the smallest for the Ag(I) emitter and the largest for the Cu(I) emitter (Figure 2c and Table S10). For example, the calculated metal (n+1)p orbital contributions to the HOMO are 0.49%, 0.61%, and 0.69% for **Ag‐1**, **Au‐1**, and **Cu‐1**, respectively. This means that the strength of π(M…N) interactions in the excited state follows the order of Ag(I) emitter < Au(I) emitter < Cu(I) emitter.

As shown in our previous works, metal (n+1)s‐nd and (n+1)p‐nd hybridizations are significantly affected by relativistic effects [53, 54]. The influences of relativistic effects on metal (n+1)p‐nd hybridization, M─L interactions, and the rotational dynamics of excited states of emitters are discussed in detail in Section S2.2.2.

### 2.3 Quantitative Analysis of Excited‐State Metal‐Ligand π‐Interactions

**FIGURE 3:**
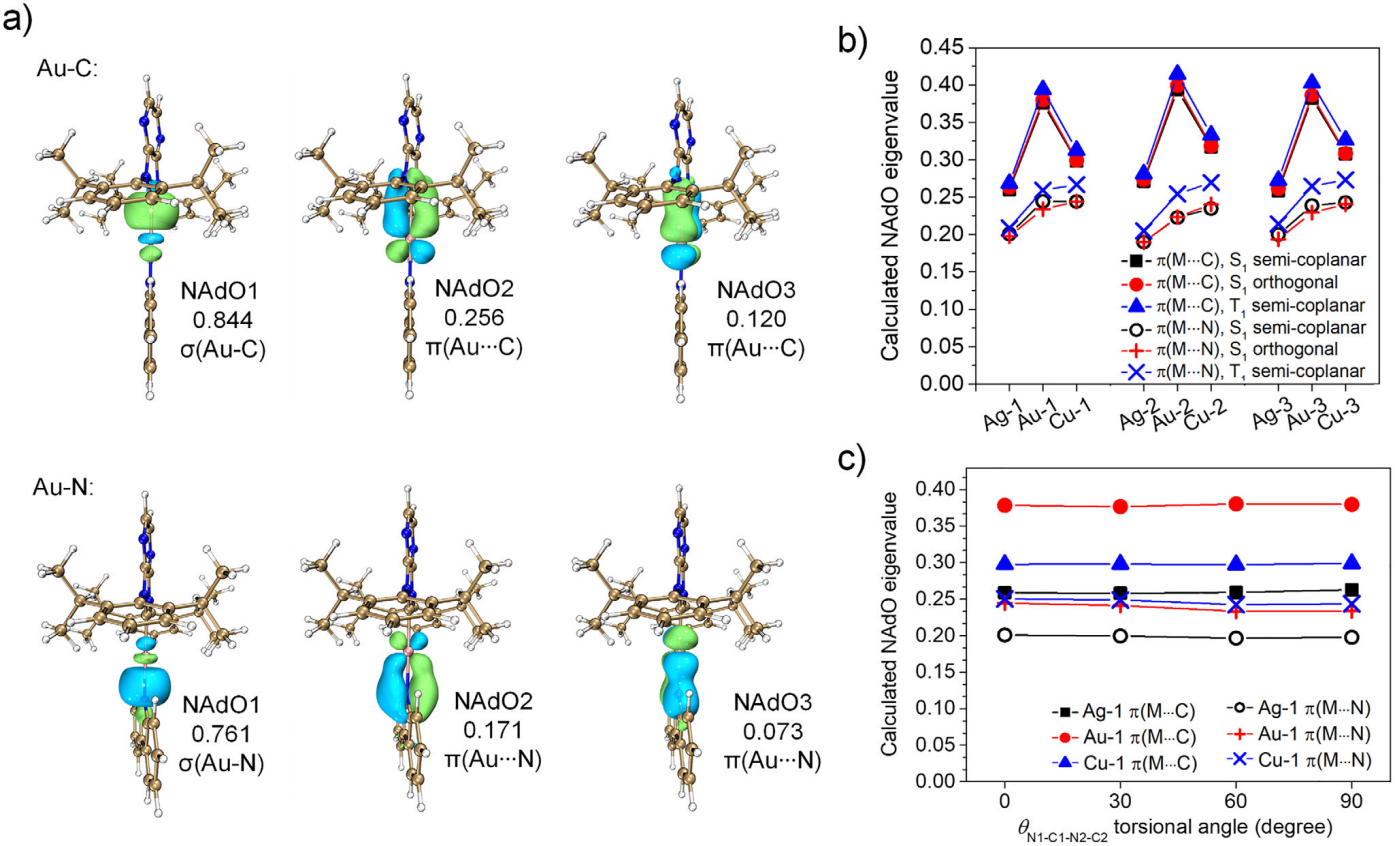
(a) Three major NAdOs of the Au─C and Au─N bonds of **Au‐1** in optimized semi‐coplanar geometries in the S_1_ excited state. NAdO1: σ‐bonding between the d_z_^2^ orbital of the metal and the p_z_ orbital of the attached C or N atom. NAdO2 and NAdO3: π‐interactions between the d_xz_/d_yz_ orbitals of the metal and the p_x_/p_y_ orbitals of the attached C or N atoms. (b) Sum of the eigenvalues of NAdOs (NAdO2 and NAdO3) representing the π(M…C) interaction or the π(M…N) interaction for **M‐1**, **M‐2**, and **M‐3** in optimized semi‐coplanar and orthogonal geometries in the S_1_ excited state, and in optimized semi‐coplanar geometries in the T_1_ excited state. (c) Sum of eigenvalues of NAdOs (NAdO2 and NAdO3) representing the π(M…C) and π(M…N) interactions for **M‐1** in twist geometries with various dihedral angles in the S_1_ excited state.

Although the strength of the emitter's M─L π‐interaction is related to the degree of metal (n+1)p‐nd hybridization, it remains a question whether the excited‐state dihedral angle rotation occurs on the M─C or M─N bonds. To graphically study the bonding modes of the M─C and M─N bonds, we performed natural adaptive orbital (NAdO) analysis [55] using the Multiwfn 3.8(dev) program [56]. NAdO analysis converts occupied molecular orbitals (MOs) into NAdOs, thereby providing intuitive visualization of electron contribution to the fuzzy bond order in three‐dimensional space. The NAdO eigenvalues indicate the magnitude of these contributions. Figure 3a shows the NAdO plots of Au─C and Au─N bonds in emitter **Au‐1** in the optimized semi‐coplanar S_1_ geometry. Similar NAdO plots for **M‐1**, **M‐2**, and **M‐3** in different geometries are shown in Figures S34–S36. Three major NAdOs are observed for both M─C and M─N bonds, including one σ‐type NAdO (NAdO1) and two π‐type NAdOs (NAdO2 and NAdO3). NAdO1 shows σ‐bonding between the d_z_^2^ orbital of the metal and the p_z_ orbital of the attached C or N atom. NAdO2 and NAdO3 show π‐interactions between the d_xz_/d_yz_ orbitals of the metal and the p_x_/p_y_ orbitals of the attached C or N atoms. For each emitter in semi‐coplanar geometry, orthogonal geometry, and twist geometries with various dihedral angles, the eigenvalues of π‐type NAdOs in the M─N bond are smaller than those in the M─C bond, and the order of eigenvalues of π‐type NAdOs in the M─N bond is Ag(I) emitter < Au(I) emitter < Cu(I) emitter (Figure 3b,c; Figures S32–S36, Tables S12 and S13). For example, the eigenvalues of π‐type NAdOs in M─C/M─N bonds are 0.260/0.201 for **Ag‐1**, 0.376/0.244 for **Au‐1**, and 0.298/0.250 for **Cu‐1**, respectively, in the S_1_ state‐optimized semi‐coplanar geometries (Figure 3a and Figure S34). Similar trends were observed for emitters with different twist geometries in the S_1_ and T_1_ states (Figure 3c and Figure S33). Therefore, we infer that the excited‐state dihedral angle rotation of the emitters is more likely to occur through the rotation of the M‐N bond rather than the rotation of the M─C bond due to the weaker M─N π‐interaction. Moreover, within a given set of emitters, this rotational flexibility decreases in the order of Ag(I) emitter > Au(I) emitter > Cu(I) emitter. The observed rotational flexibility trend is consistent with the results of excited‐state potential energy surface (PES) calculations at the TDDFT level, using the framework of the similarity‐transformed equation‐of‐motion domain‐based local pair natural orbital CCSD (STEOM‐DLPNO‐CCSD) [57, 58, 59, 60, 61, 62, 63, 64], and using the combined density functional theory and multireference configuration interaction (DFT/MRCI) method [65, 66, 67] (see Sections S2.2.1 and S2.2.4, Figures S24–S27 for detailed PES analysis).

**FIGURE 4:**
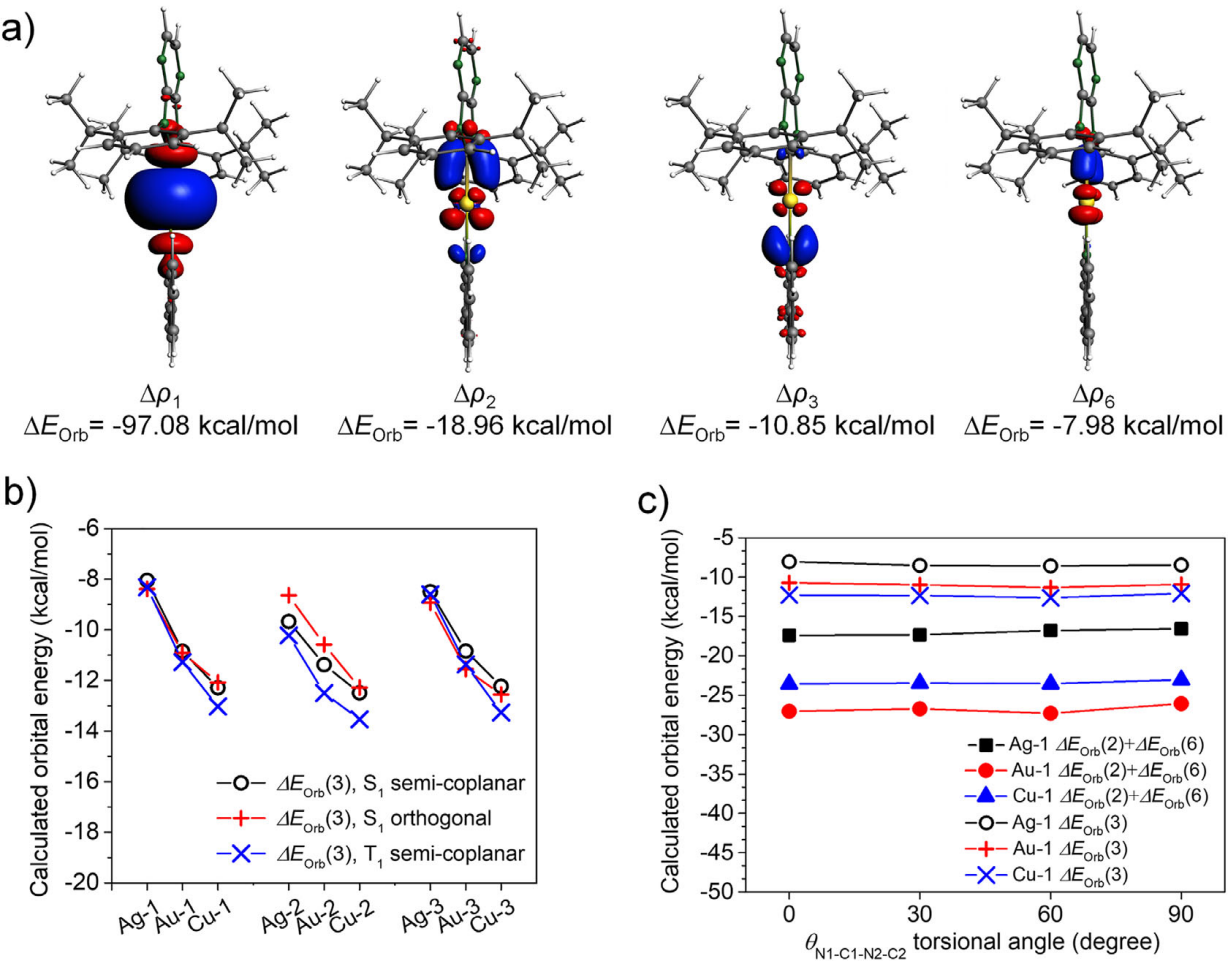
(a) Major ETS‐NOCV deformation density contributions to the M─C/M─N σ‐ and π‐interactions in **Au‐1** in optimized semi‐coplanar geometries in the S_1_ excited state. Isovalue = 0.002. Electron density transfer from red to blue isosurfaces. ∆*ρ*_1_: σ‐bonding, electron density shifts from C/N p_z_ orbitals to metal s‐d hybridized orbitals. ∆*ρ*_2_ and ∆*ρ*_6_: π‐interactions, electron density shifts from metal p‐d hybridized orbitals to C p_x_ or p_y_ orbitals, respectively. ∆*ρ*_3_: π‐interactions, electron density shifts from metal p‐d hybridized orbitals to N p_x_ orbitals. (b) Calculated orbital energies [*∆E*_Orb_(3)] of deformation density *∆ρ*_3_ contributing to the π(M…N) interactions for **M‐1**, **M‐2**, and **M‐3** in optimized semi‐coplanar and orthogonal geometries in the S_1_ excited state, and in optimized semi‐coplanar geometries in the T_1_ excited state. (c) Calculated orbital energies [*∆E*_Orb_(3)] of deformation density *∆ρ*_3_ contributing to the π(M…N) interactions and calculated orbital energies [*∆E*_Orb_(2) + *∆E*_Orb_(6)] of deformation densities (∆*ρ*_2_+∆*ρ*_6_) contributing to π(M…C) interactions for **M‐1** in twisted geometires with various dihedral angles in the S_1_ excited state.

The extended transition‐state method based on natural orbitals of chemical valence (ETS‐NOCV) [68] can be used to convert the electron density changes during bond formation into chemically intelligible components based on fragment molecular orbitals. ETS‐NOCV analysis differs from NAdO analysis in that it can reveal electron density flows and quantify energetic contributions to bond energies while resolving inter‐fragment interactions. In this work, we further elucidate the formation mechanisms of the aforementioned M─C/M─N σ‐ and π‐interactions using ETS‐NOCV analysis in the Amsterdam Density Functional (ADF) 2019 package [69, 70, 71]. When the interaction between the metal atom and the ligand residue is considered, NOCV deformation density channels representing M─C/M─N σ‐ and π‐interactions can be observed. Figure 4a shows the main NOCV deformation density channels of **Au‐1**, where the red and blue regions represent the electron density depletion and accumulation, respectively. Similar NOCV plots for **Ag‐1**, **Cu‐1**, **M‐2**, and **M‐3** are shown in Figure S40. Four main NOCV deformation density channels are observed, representing the M─C/M─N σ‐ and π‐interactions: 1) ∆*ρ*_1_ represents a σ‐bonding, where electron density is transferred from the C/N p_z_ orbital to the metal s‐d hybridized orbital. 2) ∆*ρ*_2_ and ∆*ρ*_6_ represent π‐interactions, where electron density is transferred from the metal p‐d hybridized orbital to the C p_x_ or p_y_ orbitals, respectively. 3) ∆*ρ*_3_, represents a π‐interaction, where electron density is transferred from the metal p‐d hybridized orbital to the N p_x_ orbital. Although carbazole is a strong electron‐donating ligand, π‐interactions are still possible between the N atom and the metal atom for the following reasons: 1) In the excited state, the HOMO, mainly localized on the carbazole ligand, is half‐occupied, resulting in a reduction of electron density on the carbazole ligand. 2) As shown by the resonance structures of the carbazole ligand (Figure S23), the negative charge of the lone‐pair electrons on the N atom is delocalized over the entire aromatic ring, making the N atom more susceptible to π‐electrons from the metal atom. Taking the optimized semi‐coplanar geometry in the S_1_ state of **Au‐1** as an example, the π‐interactions between Au and C (∆*ρ*_2_ and ∆*ρ*_6_) lead to energy stabilization of *∆E*_Orb_(2) + *∆E*_Orb_(6) = −26.94 kcal/mol. The weaker π‐interaction between Au and N (∆*ρ*_3_) leads to a relatively smaller energy stabilization of *∆E*_Orb_(3) = −10.85 kcal/mol (Figure 4a and Table S14). For each emitter in semi‐coplanar geometry, orthogonal geometry, and twist geometries with various dihedral angles, the energy stabilization due to M─N π‐interactions is smaller than that of the M─C π‐interactions (Figure 4b,c; Figures S40 and S41, Tables S14 and S15). Comparing emitters with different metal atoms, the Ag(I) emitter has the smallest *∆E*_Orb_(3), while the Cu(I) emitter has the largest *∆E*_Orb_(3) (see the results of different optimized geometries of **M‐1**, **M‐2**, and **M‐3** in S_1_ and T_1_ excited states in Figure 4b, Figure S40, and Table S14). The ETS‐NOCV analysis results provide additional insights into the electron density transfer and the energy of bonding interactions. The excited‐state dihedral angle rotation occurs at the M─N bond, and the rotational flexibility follows the order of Ag(I) emitters > Au(I) emitters > Cu(I) emitters, which is consistent with the NAdO analysis and the PES results (see Section S2.2.1 for detailed PES analysis).

After revealing the formation mechanism of M─C/M─N σ‐ and π‐interactions involving electron density flow, we further applied charge decomposition analysis (CDA) [56, 72, 73] using the Multiwfn 3.8(dev) [56] program to quantify the charge (electron) donation and back donation between the metal atom and the ligands in the emitter. The CDA results show that the electron flow is related to the formation of C/N→M σ‐bonds and M→C/N π‐interactions in the excited state. The results support those of the NAdO and ETS‐NOCV analyses, providing additional quantitative insights into the excited‐state dihedral angle rotational flexibility of the emitters through evaluation of electron density transfer. Please see Section S2.2.3 of the Supporting Information for detailed results and discussions.

### 2.4 TADF Radiative Decay Rate, Spin‐Orbit Coupling and Reverse Intersystem Crossing

**FIGURE 5:**
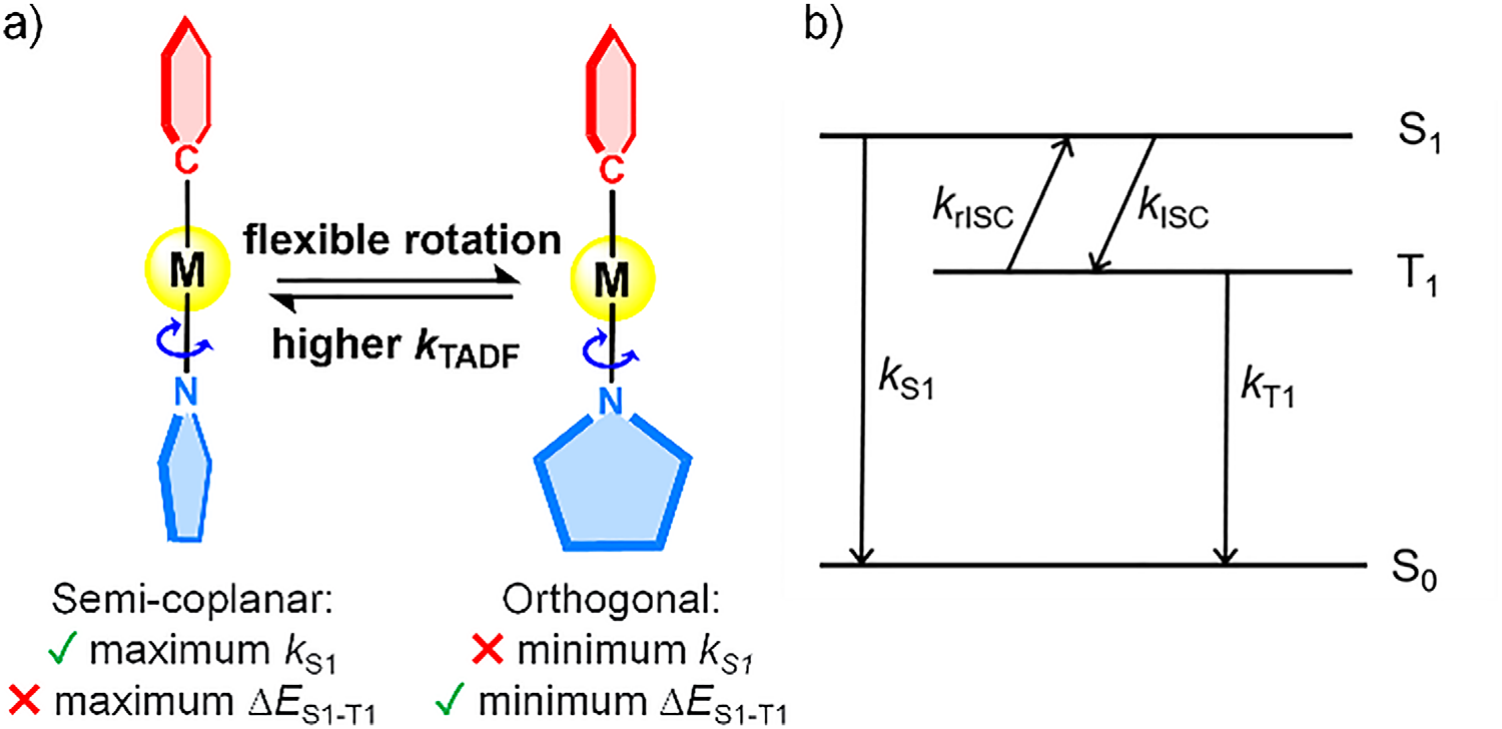
(a) Schematic diagram showing the impact of M─N bond rotation on the TADF radiative decay rate of CMA emitters. (b) Schematic diagram of the photophysical process in the three‐state model.

The radiative decay rate constant of TADF emitters can be increased by reducing *∆E*_S1‐T1_ and increasing *k*_S1_. In the semi‐coplanar configuration, *k*_TADF_ can be increased by promoting *k*_S1_ due to the increased overlap between the HOMO and LUMO (Figure 5a). However, a simultaneous increase in *∆E*_S1‐T1_ is detrimental to high *k*_TADF_. In contrast, in the orthogonal configuration, reducing *∆E*_S1‐T1_ increases *k*_TADF_ by promoting ISC and *r*ISC, but this effect is offset by a smaller *k*_S1_ (that reduces *k*_TADF_). Notably, this dilemma can be solved by achieving flexible dihedral angle rotations of CMA emitters. Such rotational flexibility allows for rapid switching between semi‐coplanar and orthogonal rotamers, paving the way for simultaneously achieving smaller *∆E*_S1‐T1_ and larger *k*_S1_. Thus, increasing excited‐state rotational flexibility can increase the radiative decay rate of CMA emitters.

We calculated the *k*_TADF_ values of the emitters by considering excited‐state rotational dynamics and the delicate inter‐state equilibria. First, based on a three‐state model (including the S_0_, S_1_, and T_1_ states) (Figure 5b), the *k*_TADF_ values of relaxed rotamers with different torsional angles 𝜃 (*𝜃* = 0°–180°, step = 15°, Figures S24–S27) were estimated [74, 75]. The reason for not considering higher excited states in the calculation of *k*_TADF_ is discussed in the later section. In this model, the rate equation for the excited‐state dynamics is as follows:

(1) $$\frac{d\left[S_{1}\right]}{\mathrm{dt}}=-\left(k_{S1}+k_{\mathrm{ISC}}\right)\left[S_{1}\right]+k_{\mathrm{rISC}}\left[T_{1}\right]$$

(2) $$\frac{d\left[T_{1}\right]}{\mathrm{dt}}=-\left(k_{T1}+k_{\mathrm{rISC}}\right)\left[T_{1}\right]+k_{\mathrm{ISC}}\left[S_{1}\right]$$

where [S_1_] and [T_1_] represent the exciton populations of the S_1_ and T_1_ states, respectively, *k*_T1_ represents the radiative decay rate constant of the T_1_ → S_0_ transition, and *k*_ISC_ and *k*_rISC_ represent the rate constants of the ISC and *r*ISC processes, respectively.

The differential Equations (1) and (2) can be reformulated as:

(3) $$\begin{array}{c} \frac{d}{\mathrm{dt}}\;\left(\begin{array}{c} \left[S_{1}\right]\\ \left[T_{1}\right] \end{array}\right)=\left(\begin{array}{cc} -k_{S1}-k_{\mathrm{ISC}} & k_{\mathrm{rISC}}\\ k_{\mathrm{ISC}} & -k_{T1}-k_{\mathrm{rISC}} \end{array}\right)\;\left(\begin{array}{c} \left[S_{1}\right]\\ \left[T_{1}\right] \end{array}\right)=\;A\left(\begin{array}{c} \left[S_{1}\right]\\ \left[T_{1}\right] \end{array}\right) \end{array}$$

Here, the eigenvalue λ of matrix A in Equation (3) is calculated using by:

(4) $$\det\left(A-\lambda E\right)=0$$

where E refers to the identity matrix. Equation (4) can be rewritten as:

(5) $$\begin{array}{ccc} & & \lambda^{2}+\left(k_{S1}+k_{\mathrm{ISC}}+k_{T1}+k_{\mathrm{rISC}}\right)\lambda \\ & & \;+\left(k_{S1}+k_{\mathrm{ISC}}\right)\left(k_{T1}+k_{\mathrm{rISC}}\right)-k_{\mathrm{ISC}}\;k_{\mathrm{rISC}}=0 \end{array}$$

By solving Equation (5), the eigenvalues λ_1,2_ are derived as:

(6) $$\begin{array}{ccc} \lambda_{1,2} & = & -\frac{1}{2}\left(k_{S1}+k_{\mathrm{ISC}}+k_{T1}+k_{\mathrm{rISC}}\right.\\ & & \pm \left.\sqrt{{\left(k_{S1}+k_{\mathrm{ISC}}-k_{T1}-k_{\mathrm{rISC}}\right)}^{2}+{4k}_{\mathrm{ISC}}k_{\mathrm{rISC}}}\right) \end{array}$$

Here, the radiative decay rate constants of the prompt fluorescence (*k*_PF_) and delayed fluorescence (*k*_TADF_) are given by *k*_PF_ = |λ_1_| and *k*_TADF_ = |λ_2_|, respectively. The TADF radiative decay rate constants of thermally accessible rotamers with different torsional angles 𝜃 (*k*_TADF,𝜃_) were calculated using Equation (6) and are listed in Table S18. The overall *k*_TADF_ of the emitter was estimated by considering the *k*_TADF,𝜃_ values of all the rotamers on the excited‐state potential energy surfaces, assuming rapid thermal equilibrium among these rotamers (see Section S2.1.7 for detailed calculation methods and results for *k*_S1_, *k*_T1_, *k*_ISC_, and *k*_rISC_).

The above method for calculating *k*_TADF_ takes into account the excited‐state rotational dynamics of the emitter. The calculated *k*_TADF_ values for the emitters in toluene solution are in good agreement with the experimental results measured under the same conditions (Table 1). When comparing emitters with different metal atoms, the order of *k*_TADF_ values is: Ag(I) emitter > Au(I) emitter > Cu(I) emitter. Silver has the smallest degree of (n + 1)p‐nd orbital hybridization and forms the weakest π‐interaction with the nitrogen atom of the carbazole ligand. This results in the most flexible excited‐state dihedral angle rotation of Ag(I) emitter, resulting in the largest *k*_TADF_ values.

For the CMA complex, although the S_1_ and T_1_ states have similar LLCT electronic nature and are dominated by the HOMO→LUMO transition, the STEOM‐DLPNO‐CCSD results show that the T_1_ state also has minor contributions from other transitions (Table S19). The slight difference in the electronic nature of the S_1_ and T_1_ states leads to a non‐zero spin‐orbit coupling matrix element (SOCME), thereby enabling ISC and *r*ISC between the states. For the T_1_ state, the contribution of other transitions (other than HOMO→LUMO transitions) is the largest for the Cu(I) emitter and the smallest for the Ag(I) emitter (Table S19). The SOCME values follow a trend of Cu(I) emitters > Au(I) emitters > Ag(I) emitters (Table S18). The SOCME trend mainly originates from differences in d‐orbital participation in the charge–transfer excited states. Cu(I) has the highest degree of d‐orbital participation due to its smaller crystal field splitting and stronger d‐orbital ligand mixing. For Au(I) and Ag(I) emitters with smaller d‐orbital contributions, the external heavy atom effect dominates, resulting in a larger SOCME for Au(I) emitters than for Ag(I) emitters [32]. It is worth noting that the *r*ISC rate constant is determined not only by the spin‐orbit coupling strength, but also by other factors such as the S_1_‐T_1_ energy gap and reorganization energy according to the Marcus theory [76]. Therefore, there is no fixed trend in *k*_rISC_ values across different series of Au(I), Ag(I), and Cu(I) emitters. The difference in the TADF radiative decay rates of Au(I), Ag(I), and Cu(I) emitters is mainly due to the differences in their excited‐state rotational flexibility, whereas the variations in spin‐orbit coupling strength and *r*ISC rate are less important.

### 2.5 Effect of Ligand on TADF Efficiency and OLED Device Performance

**FIGURE 6:**
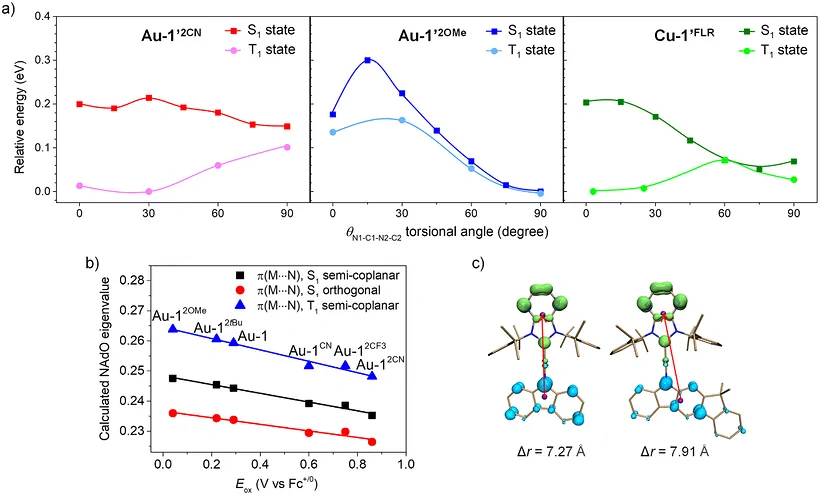
(a) STEOM‐DLPNO‐CCSD calculated potential energy surfaces of **Au‐1’^2CN^**, **Au‐1’^2OMe^**, and **Cu‐1’^FLR^** in excited states S_1_ and T_1_. (b) Sum of the calculated eigenvalues of NAdOs (NAdO2 and NAdO3) representing the π(M…N) interactions in difference optimized geometries (semi‐coplanar and orthogonal geometries in the S_1_ excited state and semi‐coplanar geometries in the T_1_ excited state) as a function of the cyclic voltammetry (CV) measured oxidation potential (*E*_ox_) of emitters **Au‐1**, **Au‐1^2OMe^**, **Au‐1^2^ *^t^* ^Bu^**, **Au‐1^CN^**, **Au‐1^2CF3^**, and **Au‐1^2CN^**. See Section S1.5 for details of the electrochemical measurement. (c) Calculated hole and electron distributions and the distance between centroids of hole and electron (Δ*r*) of **Cu‐1** and **Cu‐1^FLR^** in optimized semi‐coplanar geometries in the S_1_ excited state. Hole and electron distributions are represented by blue and green isosurfaces, respectively.

The results reported in the literature and the new results obtained in this work show that for most d^10^ CMA emitter derivatives with the same carbene ligand, the introduction of electron‐withdrawing substituents on the carbazole ligand increases the TADF radiative decay rate, while the introduction of electron‐donating substituents decreases the TADF radiative decay rate (Tables 1 and 3) [8, 20, 25, 26, 47]. We calculated the electronic configuration of Au in a series of **Au‐1** derivatives with electron‐withdrawing substituents (**Au‐1^CN^**, **Au‐1^2CN^**, **Au‐1^CF3^**, and **Au‐1^2CF3^**) or electron‐donating substituents (**Au‐1^2^ *^t^* ^Bu^**, **Au‐1^OMe^**, and **Au‐1^2OMe^**) on the carbazole ligand, and compared them with the electronic configuration of **Au‐1**. Taking the optimized semi‐coplanar geometry in the S_1_ state as an example, the calculated Au 6p orbital electron populations for **Au‐1^2CN^**, **Au‐1^2CF3^**, **Au‐1^CN^**, **Au‐1^CF3^**, **Au‐1**, **Au‐1^2^ *^t^* ^Bu^**, **Au‐1^OMe^**, and **Au‐1^2OMe^** are 0.0871, 0.0880, 0.0884, 0.0909, 0.0909, 0.0915, 0.0917, and 0.0921, respectively (Table S9). This means that electron‐withdrawing substituents on the carbazole ligand reduce the extent of Au 6p‐5d orbital hybridization, whereas electron‐donating substituents enhance this hybridization. The calculated metal (n+1)p orbital components in the HOMO are smaller for **Au‐1^2CN^**, **Au‐1^2CF3^**, **Au‐1^CN^**, and **Au‐1^CF3^**, while larger for **Au‐1^2^ *^t^* ^Bu^**, **Au‐1^OMe^**, and **Au‐1^2OMe^** (Table S11). For example, in the optimized S_1_ semi‐coplanar geometry, the calculated metal (n+1)p orbital components in the HOMO are 0.39%, 0.61%, and 0.64% for **Au‐1^2CN^**, **Au‐1**, and **Au‐1^2OMe^**, respectively. The NAdO analysis results indicate that emitters substituted with electron‐withdrawing groups have weaker π(M…N) interactions in the excited state, and vice versa (Figures S32 and S37–S39 and Table S12). The STEOM‐DLPNO‐CCSD calculated PES for simplified emitters shows that the excited‐state rotational flexibility follows the order of **Au‐1’^2CN^** > **Au‐1’** > **Au‐1’^2OMe^** (Figure S24b and Figure 6a). Therefore, the introduction of electron‐withdrawing substituents on the carbazole ligand reduces the Au (n+1)p‐nd orbital hybridization, leading to weakened excited‐state π(M…N) interaction and more flexible dihedral angle rotation, thereby increasing the *k*_TADF_ value of the emitter. In addition, we measured the oxidation potential (*E*_ox_) of the emitters by cyclic voltammetry (CV) to examine the influence of the carbazole substitutions. The results show that there is a linear relationship between the *E*_ox_ value and the calculated excited‐state π(M…N) interaction strength (Figure 6b). This relationship suggests that *E*_ox_ can serve as an experimental descriptor for estimating the excited‐state π(M…N) interaction strength in CMA emitters.

**TABLE 3:** Comparison of experimental *k*_TADF_ for CMA emitters with electron‐withdrawing or electron‐donating substituents on the carbazole ligands, and with or without π‐extended substituents on the carbazole ligands.

*k*_TADF_ (10^5^ s^−1^) (electron‐donating/withdrawing)				*k*_TADF_ (10^5^ s^−1^) (π‐extended)	
**Au‐1** [26]	**Au‐1^CN^** [26]	**Au‐1^2CN^** [26]	**Au‐1^2CF3^**	**Au‐1^BIM^** [36]	
10.0^a^	22.1^a^	20.0^a^	20.0^a^, ^f^	15^a^/38^d^	
**Cu‐1** [25]	**Cu‐1^CN^** [25]	**Cu‐1^2^ *^t^* ^Bu^** [25]	**Cu‐1^2Ph^** [25]	**Cu‐1^FLR^** [27]	
16.1^a^/21.5^c^	16.1^a^/21.7^c^	12.7^a^/17.8^c^	12.5^a^/21.1^c^	23.0^a^	
**Au‐1**	**Au‐1^2^ *^t^* ^Bu^**	**Au‐1^2OMe^**		**Au‐3** [5]	**Au‐4** [36]^g^
24.1^c^, ^f^	20.0^c^, ^f^	11.0^c^, ^f^		6.3^b^/10^d^	11^a^/22^d^
**Cu‐7** [8]^g^	**Cu‐7^CN^** [8]^g^	**Cu‐7^2CN^** [8]^g^		**Au‐10** [36]^g^	**Au‐10^BIM^** [36]^g^
1.6^d^	3.8^b^/4.0^d^	6.2^b^/6.5^d^		15^a^	23^a^/37^d^
**Cu‐8** [47]^g^	**Cu‐8^CF3^** [47]^g^	**Cu‐8^2Me^** [47]^g^	**Cu‐8^Ph^** [47]^g^	**Au‐11** [36]^g^	**Au‐11^BIM^** [36]^g^
3.5^d^	3.9^d^	2.6^d^	1.5^d^	10^a^/12^d^	18^a^/30^d^
**Au‐9^DPA^** [48]^g^	**Au‐9^ACD^** [48]^g^	**Au‐9^DPAC^** [48]^g^	**Au‐9^DMAC^** [48]^g^		
8.7^e^	26.0^e^	8.5^e^	6.3^e^		

Measured in

^a^ toluene solution.

^b^ 2‐MeTHF solution.

^c^ 2 wt.% mCP.

^d^ 1 wt.% PS film.

^e^ 5 wt.% Zeonex film.

^f^ See Section S1.6 for detailed photophysical measurement results.

^g^ Molecular structures are shown in Figure S1.

The literature also shows that the TADF radiative decay rate of CMA emitters can be increased by introducing π‐extended substituents on the carbazole ligand (Table 3). The NAdO analysis results of **Cu‐1** and **Cu‐1^FLR^** reveal that the emitter with π‐extended substituents on the carbazole ligand (**Cu‐1^FLR^**) has a weaker π(M…N) interaction in the excited state (Figures S37–S39 and Table S12). This weakening of π(M…N) interaction does not originate from reduced metal (n+1)p‐nd orbital hybridization; on the contrary, the degree of metal (n+1)p‐nd orbital hybridization is enhanced in **Cu‐1^FLR^** (Tables S8 and S9). The calculated hole (h^+^) and electron (e^−^) distributions show that, upon π‐extension, the hole center of **Cu‐1^FLR^** shifts toward the π‐extended substituent, resulting in a longer electron–hole distance compared to **Cu‐1** (Figure 6c and Table S17). This redistribution facilitates delocalization of the M─N π‐electrons toward the extended π system, thereby weakening the π‐interaction between the metal and the nitrogen atom. The PES of the emitters **Cu‐1’** and **Cu‐1’^FLR^** calculated using the STEOM‐DLPNO‐CCSD method shows that **Cu‐1’^FLR^** is more flexible in rotation than **Cu‐1’** in the excited state (Figure S24b and Figure 6a). It is worth noting that if the π‐extended ring on the carbazole ligand extends to the vicinity of the carbene ligand's bulky substituent, the steric hindrance may restrict the dihedral angle rotation, thereby reducing the TADF radiative decay rate of the emitter. Therefore, it is best to design the carbazole ligand with a π‐extended ring positioned away from the carbene ligand.

Notably, hole and electron analysis showed that introducing electron‐withdrawing or π‐extended substituents on the carbazole ligand reduces the hole–electron overlap in the emitter (Figure S43 and Table S17). This reduced overlap can narrow the singlet‐triplet energy gap (Table S22), thereby increasing the radiative decay rate of TADF. Combined with the mechanism of weakening the M─N π‐interaction discussed above, these two effects work synergistically to improve the radiative efficiency of TADF.

Introducing electron‐withdrawing substituents into carbazole ligands can not only improve the excited‐state rotational flexibility of the emitter and the TADF radiative decay rate, but also induce a blueshift in TADF emission [8, 20, 25, 26, 47, 48]. In some deep‐blue luminescent CMAs employing cyclic (alkyl)(amino)carbene (CAAC) ligands, the energy of the lowest ^1/3^LLCT excited state can approach the energy of the intraligand triplet state (^3^IL_cz_) of the carbazole ligand, thereby slowing down the emission kinetics [31, 32, 38]. The emission wavelength of ^3^IL_cz_ of non‐substituted carbazole is ∼ 430 nm [4, 5]. The CMA emitters investigated in this work emit red, yellow, green, and sky blue light with wavelengths > 470 nm, and therefore their emission energy is lower than that of a typical ^3^IL_cz_ emitter (Table S21). The vertical energies and transition properties of the first five lowest excited states were calculated and listed in Table S22. Only the T_1_ state lies below the S_1_ state, and the T_2_ state is 0.21–1.02 eV higher than the S_1_ state and 0.35–1.19 eV higher than the T_1_ state. Therefore, the effect of the higher excited states on the TADF emission is negligible. Even if the introduction of electron‐withdrawing substituents can lead to the mixing of the ^1/3^LLCT with the ^3^IL_cz_ state or other higher‐lying triplet states, thereby slowing down the emission kinetics, this effect can be mitigated through further ligand modification. For example, previous studies have shown that N‐substitution of the carbazole ligands can increase the energy of the ^3^IL_cz_ state, while modification of carbene ligands can decrease the energy of the ^1/3^LLCT state [38, 39].

By modifying carbazole ligands to weaken the excited‐state M─N π‐interactions, the radiative decay rate of TADF in solution can be increased because this allows the excited‐state dihedral angle to rotate more flexibly. However, this modification may also weaken the excited‐state M─N π‐interaction, thereby reducing the strength of the excited‐state M─N bond. This, in turn, makes the M─L bond more prone to dissociation, ultimately reducing the stability of the emitter during electroluminescence. We recognize that the operational lifetime of an OLED device depends on many factors, such as emission energy, steric hindrance of large substituents, radiative decay rate, molecular packing, and excited‐state bond dissociation processes. For example, higher energy emission typically increases the likelihood of exciton energy and material degradation, thereby reducing the operational stability of the OLED device [77, 78]. Given the need to find effective methods to improve the operational lifetime of d^10^ CMA‐based OLED devices (which are currently far from satisfactory to meet the demands of practical industrial applications), here, we share our findings and perspectives on the impact of excited‐state M**─**N π interaction strength on the operational stability of d^10^ CMA OLED devices. We fabricated and characterized OLED devices based on **Au‐1^2CF3^** and **Au‐1^2CN^** using the same device structure (see Section S1.1.4 for details of device structure, fabrication, and characterization). The **Au‐1^2CF3^**‐ and **Au‐1^2CN^**‐based devices exhibited almost identical electroluminescence maximum (469 and 467 nm, respectively), thus minimizing the impact of emission energy on device stability comparisons. In both Au emitter structures, since the ─CN and ─CF_3_ groups are far from the metal center, their steric hindrance or protection effect on the metal center is very small and can be ignored when comparing the operational stability of the devices (please see the calculated topological steric maps and buried volumes % *V*_bur_ in Figure S44 for details). At an initial luminance (L_0_) of 1000 cd m^−2^, the operational lifetime (LT_90_) of the **Au‐1^2CN^**‐based device was approximately 2.7 times shorter than that of the **Au‐1^2CF3^**‐based device. This is consistent with the weaker (though subtle) excited‐state M─N π‐interactions in **Au‐1^2CN^ (**compared to **Au‐1^2CF3^**, see Figure 6b and Table S12). Therefore, the weakening of excited‐state M─N π‐interactions caused by electron‐withdrawing substituents on the carbazole ligand may be one of the factors leading to the shortened device operational lifetime. We also fabricated and characterized OLED devices based on **Au‐1** and **Au‐1^CN^**, with the same device structure as those based on **Au‐1^2CF3^** and **Au‐1^2CN^**. As shown in Table S5, the LT_90_ values of OLED devices based on **Au‐1**, **Au‐1^CN^**, **Au‐1^2CF3^**, and **Au‐1^2CN^** emitters are 1709, 131, 1.01, and 0.37 h, respectively. The OLED devices using **Au‐1^CN^**, **Au‐1^2CF3^**, and **Au‐1^2CN^** emitters (with electron‐withdrawing carbazole ligands) have shorter operational lifetimes than those using **Au‐1**. Incidentally, this observation can be attributed to the weaker excited‐state M─N π‐interactions in **Au‐1^CN^**, **Au‐1^2CF3^**, and **Au‐1^2CN^** (compared to **Au‐1**, see Figure 6b and Table S12). While excited‐state M─N π interactions are certainly not the only factor affecting the lifetime of d^10^‐CMA‐based OLED devices, this study found a significant correlation between excited‐state M─N π interactions and the lifetime of **Au‐1**, **Au‐1^CN^**, **Au‐1^2CF3^**, and **Au‐1^2CN^**‐based OLED devices, despite differences in emission energies among these four gold emitters. Thus, the importance of excited‐state M─N π interactions among the many factors affecting the operating lifetime of d^10^‐CMA‐based OLED devices needs further investigation. We hope that this study can provide a new perspective for finding ways to improve the operating stability of d^10^‐CMA substrate devices, particularly blue and green emitting devices.

## 3 Conclusion

In this work, we elucidate the roles of metal orbital hybridization, excited‐state M─L π‐interactions, and rotational flexibility in TADF of Au(I), Ag(I), and Cu(I) CMA emitters, and describe how these factors can be modulated through ligand modification to improve the efficiency and excited‐state stability of the emitters. Through DFT/TDDFT calculations, high‐level STEOM‐DLPNO‐CCSD and DFT/MRCI calculations, and various wavefunction analyses, we report the following findings: 1) In the excited state, metal atoms form π‐interactions with carbazolyl nitrogen atoms. 2) A lower degree of metal (n+1)p‐nd orbital hybridization weakens the excited‐state M─N π‐interaction, thereby lowering the rotational barrier and allowing for more flexible rotation of the dihedral angles along the M─L backbone. This increased rotational flexibility enables the simultaneous achievement of smaller *∆E*_S1‐T1_ values and larger *k*_S1_ values, ultimately leading to a higher TADF radiative decay rate. This mechanism explains the general order of TADF radiative decay rates observed in d^10^ CMA emitters: Ag > Au > Cu. However, the weakening of excited‐state M─N π‐interactions also reduces the strength of excited‐state M─N bonds. This, in turn, makes the metal‐ligand bonds of CMA emitters more easily dissociate during electroluminescence. 3) Here, we systematically link the TADF radiative decay rate constant of d^10^ CMA emitters with the rotational dynamics of its excited state and the inter‐state equilibrium. 4) Based on these theoretical calculations, we propose that modulating the excited‐state M─N π‐interaction is an effective strategy to improve the radiative decay rate of TADFs. Introducing electron‐withdrawing substituents onto carbazole ligands can weaken the (n+1)p‐nd orbital hybridization of the metal, while introducing π‐extended substituents on carbazole ligands can delocalize the M─N π‐electrons. Both ligand design strategies can weaken the excited‐state M─N π‐interaction, thereby increasing the TADF radiative decay rate. 5) We observed a correlation between the strength of excited‐state M─N π‐interactions and the operational stability of OLED devices based on d^10^ CMA emitters. We think that there may be a balance between maximizing the *k*_TADF_ of the d^10^ CMA emitter and the operational stability of the corresponding OLED device.

This work presents new theoretical insights into the TADF mechanism of d^10^ CMA emitters by highlighting the role of metal orbital hybridization and excited‐state M─L interactions. These findings not only provide ligand design strategies for designing more efficient and stable OLED emitters but also contribute to further theoretical research on the TADF mechanism of d^10^ metal complexes. Future research is needed to refine molecular design strategies and balance various photophysical and stability‐related factors, with the aim of further optimizing the performance and operational stability of OLEDs based on d^10^ CMA emitters.

## Conflicts of Interest

The authors declare no conflicts of interest.

## Supporting information




**Supporting File**: advs76168‐sup‐0001‐SuppMat.pdf.

## Data Availability

The data that support the findings of this study are available from the corresponding author upon reasonable request.

## References

[1] D. Di , A. S. Romanov , L. Yang , et al., “High‐Performance Light‐Emitting Diodes Based on Carbene‐metal‐amides,” Science 356 (2017): 159–163, https://www.science.org/doi/10.1126/science.aah4345. 28360136 10.1126/science.aah4345

[2] P. J. Conaghan , S. M. Menke , A. S. Romanov , et al., “Efficient Vacuum‐Processed Light‐Emitting Diodes Based on Carbene–Metal–Amides,” Advanced Materials 30 (2018): 1802285, 10.1002/adma.201802285. 29984854

[3] A. S. Romanov , S. T. E. Jones , L. Yang , et al., “Mononuclear Silver Complexes for Efficient Solution and Vacuum‐Processed OLEDs,” Advanced Optical Materials 6 (2018): 1801347, 10.1002/adom.201801347.

[4] R. Hamze , J. L. Peltier , D. Sylvinson , et al., “Eliminating Nonradiative Decay in Cu(I) Emitters: >99% Quantum Efficiency and Microsecond Lifetime,” Science 363 (2019): 601, https://www.science.org/doi/10.1126/science.aav2865. 30733411 10.1126/science.aav2865

[5] R. Hamze , S. Shi , S. C. Kapper , et al., ““Quick‐Silver” from a Systematic Study of Highly Luminescent, Two‐Coordinate, d^10^ Coinage Metal Complexes,” Journal of the American Chemical Society 141 (2019): 8616–8626, 10.1021/jacs.9b03657. 31062972

[6] A. S. Romanov , F. Chotard , J. Rashid , and M. Bochmann , “Synthesis of Copper(I) Cyclic (Alkyl)(Amino)Carbene Complexes with Potentially Bidentate N^N, N^S and S^S Ligands for Efficient White Photoluminescence,” Dalton Transactions 48 (2019): 15445–15454, 10.1039/C9DT02036E. 31187841

[7] A. S. Romanov , L. Yang , S. T. E. Jones , et al., “Dendritic Carbene Metal Carbazole Complexes as Photoemitters for Fully Solution‐Processed OLEDs,” Chemistry of Materials 31 (2019): 3613–3623, 10.1021/acs.chemmater.8b05112.

[8] S. Shi , M. C. Jung , C. Coburn , et al., “Highly Efficient Photo‐ and Electroluminescence from Two‐Coordinate Cu(I) Complexes Featuring Nonconventional *N*‐Heterocyclic Carbenes,” Journal of the American Chemical Society 141 (2019): 3576–3588, 10.1021/jacs.8b12397. 30768250

[9] F. Chotard , V. Sivchik , M. Linnolahti , M. Bochmann , and A. S. Romanov , “Mono‐ versus Bicyclic Carbene Metal Amide Photoemitters: Which Design Leads to the Best Performance?,” Chemistry of Materials 32 (2020): 6114–6122, 10.1021/acs.chemmater.0c01769.

[10] P. J. Conaghan , C. S. B. Matthews , F. Chotard , et al., “Highly Efficient Blue Organic Light‐emitting Diodes Based on Carbene‐Metal‐Amides,” Nature Communications 11 (2020): 1758, 10.1038/s41467-020-15369-8. PMC7145843 32273497

[11] J. Feng , E. J. Taffet , A.‐P. M. Reponen , et al., “Carbene–Metal–Amide Polycrystalline Materials Feature Blue Shifted Energy yet Unchanged Kinetics of Emission,” Chemistry of Materials 32 (2020): 4743–4753, 10.1021/acs.chemmater.0c01363.

[12] J. Feng , L. Yang , A. S. Romanov , et al., “Environmental Control of Triplet Emission in Donor–Bridge–Acceptor Organometallics,” Advanced Functional Materials 30 (2020): 1908715, 10.1002/adfm.201908715.

[13] M. Gernert , L. Balles‐Wolf , F. Kerner , et al., “Cyclic (Amino)(aryl)carbenes Enter the Field of Chromophore Ligands: Expanded π System Leads to Unusually Deep Red Emitting Cu^I^ Compounds,” Journal of the American Chemical Society 142 (2020): 8897–8909, 10.1021/jacs.0c02234. 32302135

[14] R. Hamze , M. Idris , D. S. Muthiah Ravinson , et al., “Highly Efficient Deep Blue Luminescence of 2‐Coordinate Coinage Metal Complexes Bearing Bulky NHC Benzimidazolyl Carbene,” Frontiers in Chemistry 8 (2020): 401, 10.3389/fchem.2020.00401. 32457877 PMC7225363

[15] A. S. Romanov , S. T. E. Jones , Q. Gu , et al., “Carbene Metal Amide Photoemitters: Tailoring Conformationally Flexible Amides for Full Color Range Emissions Including White‐emitting OLED,” Chemical Science 11 (2020): 435, 10.1039/C9SC04589A. 32190264 PMC7067249

[16] H. H. Cho , A. S. Romanov , M. Bochmann , N. C. Greenham , and D. Credgington , “Matrix‐Free Hyperfluorescent Organic Light‐Emitting Diodes Based on Carbene–Metal–Amides,” Advanced Optical Materials 9 (2021): 2001965, 10.1002/adom.202001965.

[17] T. Y. Li , D. G. Shlian , P. I. Djurovich , and M. E. Thompson , “A Luminescent Two‐Coordinate Au^I^ Bimetallic Complex with a Tandem‐Carbene Structure: A Molecular Design for the Enhancement of TADF Radiative Decay Rate,” Chemistry—A European Journal 27 (2021): 6191–6197, 10.1002/chem.202100512. 33561304

[18] J. G. Yang , X. F. Song , J. Wang , et al., “Highly Efficient Thermally Activated Delayed Fluorescence from Pyrazine‐Fused Carbene Au(I) Emitters,” Chemistry—A European Journal 27 (2021): 17834–17842, 10.1002/chem.202102969. 34705307

[19] A. Ying , Y. H. Huang , C. H. Lu , et al., “High‐Efficiency Red Electroluminescence Based on a Carbene–Cu(I)–Acridine Complex,” ACS Applied Materials & Interfaces 13 (2021): 13478–13486, 10.1021/acsami.0c22109. 33689279

[20] T. Y. Li , J. Schaab , P. I. Djurovich , and M. E. Thompson , “Toward Rational Design of TADF Two‐coordinate Coinage Metal Complexes: Understanding the Relationship Between Natural Transition Orbital Overlap and Photophysical Properties,” Journal of Materials Chemistry C 10 (2022): 4674–4683, 10.1039/D2TC00163B.

[21] Q. Gu , F. Chotard , J. Eng , et al., “Excited‐State Lifetime Modulation by Twisted and Tilted Molecular Design in Carbene‐Metal‐Amide Photoemitters,” Chemistry of Materials 34 (2022): 7526–7542, 10.1021/acs.chemmater.2c01938. 36032551 PMC9404540

[22] J. Ma , S. C. Kapper , A. Ponnekanti , J. Schaab , P. I. Djurovich , and M. E. Thompson , “Dynamics of Rotation in Two‐Coordinate Thiazolyl Copper(I) Carbazolyl Complexes,” Applied Organometallic Chemistry 38 (2022): 6728, 10.1002/aoc.6728.

[23] T. Y. Li , S. J. Zheng , P. I. Djurovich , and M. E. Thompson , “Two‐Coordinate Thermally Activated Delayed Fluorescence Coinage Metal Complexes: Molecular Design, Photophysical Characters, and Device Application,” Chemical Reviews 124 (2024): 4332–4392, 10.1021/acs.chemrev.3c00761. 38546341

[24] A. C. Brannan , H. H. Cho , A.‐P. M. Reponen , et al., “Deep‐Blue and Fast Delayed Fluorescence From Carbene–Metal–Amides for Highly Efficient and Stable Organic Light‐Emitting Diodes,” Advanced Materials 36 (2024): 2404357, 10.1002/adma.202404357. 38727713

[25] R. Tang , S. Xu , T. L. Lam , et al., “Highly Robust Cu^I^‐TADF Emitters for Vacuum‐Deposited OLEDs with Luminance up to 222 200 cd m^−2^ and Device Lifetimes (LT_90_) up to 1300 hours at an Initial Luminance of 1000 cd m^−2^ ,” Angewandte Chemie International Edition 61 (2022): e202203982, 10.1002/anie.202203982. 35647660

[26] R. Tang , S. Xu , L. Du , et al., “Au(I)‐TADF Emitters for High Efficiency Full‐Color Vacuum‐Deposited OLEDs and TADF‐Sensitized Fluorescent OLEDs with Ultrahigh Brightness and Prolonged Operational Lifetime,” Advanced Optical Materials 11 (2023): 2300950, 10.1002/adom.202300950.

[27] R. Tang , S. Xu , G. Cheng , et al., “Highly Stable and Efficient Copper(I) Sensitizer for Narrowband Red Organic Light‐Emitting Diodes with an Operational Lifetime (LT_95_) of up to 3689 h at 1000 cd m^−2^ ,” Nature Communications 16 (2025): 7776, 10.1038/s41467-025-62867-8. PMC12368058 40835784

[28] J. Föller and C. M. Marian , “Rotationally Assisted Spin‐State Inversion in Carbene–Metal–Amides Is an Artifact,” The Journal of Physical Chemistry Letters 8 (2017): 5643–5647, 10.1021/acs.jpclett.7b02701. 29110478

[29] C. R. Hall , A. S. Romanov , M. Bochmann , and S. R. Meech , “Ultrafast Structure and Dynamics in the Thermally Activated Delayed Fluorescence of a Carbene–Metal–Amide,” The Journal of Physical Chemistry Letters 9 (2018): 5873–5876, 10.1021/acs.jpclett.8b02797. 30230847

[30] E. J. Taffet , Y. Olivier , F. Lam , D. Beljonne , and G. D. Scholes , “Carbene–Metal–Amide Bond Deformation, Rather Than Ligand Rotation, Drives Delayed Fluorescence,” The Journal of Physical Chemistry Letters 9 (2018): 1620–1626, 10.1021/acs.jpclett.8b00503. 29537849

[31] S. Thompson , J. Eng , and T. J. Penfold , “The Intersystem Crossing of a Cyclic (Alkyl)(amino) Carbene Gold (I) Complex,” The Journal of Chemical Physics 149 (2018): 014304, 10.1063/1.5032185. 29981553

[32] J. Eng , S. Thompson , H. Goodwin , D. Credgington , and T. J. Penfold , “Competition Between the Heavy Atom Effect and Vibronic Coupling in Donor–Bridge–Acceptor Organometallics,” Physical Chemistry Chemical Physics 22 (2020): 4659–4667, 10.1039/C9CP06999B. 32055809

[33] J. Feng , A.‐P. M. Reponen , A. S. Romanov , et al., “Influence of Heavy Atom Effect on the Photophysics of Coinage Metal Carbene‐Metal‐Amide Emitters,” Advanced Functional Materials 31 (2021): 2005438, 10.1002/adfm.202005438.

[34] P. Li , Z. Wang , S. Wang , et al., “Construction of High‐Performance Carbene–Metal–Amide‐Like TADF Materials: A Theoretical Study,” The Journal of Physical Chemistry C 125 (2021): 26770–26777, 10.1021/acs.jpcc.1c07192.

[35] S. Lin , Q. Ou , Y. Wang , Q. Peng , and Z. Shuai , “Aggregation‐Enhanced Thermally Activated Delayed Fluorescence Efficiency for Two‐Coordinate Carbene–Metal–Amide Complexes: A QM/MM Study,” The Journal of Physical Chemistry Letters 12 (2021): 2944–2953, 10.1021/acs.jpclett.1c00020. 33725452

[36] C. N. Muniz , J. Schaab , A. Razgoniaev , P. I. Djurovich , and M. E. Thompson , “π‐Extended Ligands in Two‐Coordinate Coinage Metal Complexes,” Journal of the American Chemical Society 144 (2022): 17916–17928, 10.1021/jacs.2c06948. 36126274

[37] X. F. Song , Z. W. Li , W. K. Chen , Y. J. Gao , and G. Cui , “Thermally Activated Delayed Fluorescence Mechanism of a Bicyclic “Carbene–Metal–Amide” Copper Compound: DFT/MRCI Studies and Roles of Excited‐State Structure Relaxation,” Inorganic Chemistry 61 (2022): 7673–7681, 10.1021/acs.inorgchem.1c03603. 35200011

[38] A.‐P. M. Reponen , F. Chotard , A. Lempelto , et al., “Donor *N*‐Substitution as Design Principle for Fast and Blue Luminescence in Carbene‐Metal‐Amides,” Advanced Optical Materials 10 (2022): 2200312, 10.1002/adom.202200312.

[39] N. L. Phuoc , A. C. Brannan , A. S. Romanov , and M. Linnolahti , “Tailoring Carbene–Metal–Amides for Thermally Activated Delayed Fluorescence: A Computationally Guided Study on the Effect of Cyclic (Alkyl)(amino)carbenes,” Molecules 28 (2023): 4398, https://www.mdpi.com/1420‐3049/28/11/4398. 37298874 10.3390/molecules28114398 PMC10254582

[40] J. P. Zobel , A. M. Wernbacher , and L. González , “Efficient Reverse Intersystem Crossing in Carbene‐Copper‐Amide TADF Emitters via an Intermediate Triplet State,” Angewandte Chemie International Edition 62 (2023): 202217620, 10.1002/anie.202217620. 36762599

[41] A.‐P. M. Reponen , G. Londi , C. S. B. Matthews , et al., “Understanding Spin‐Triplet Excited States in Carbene‐Metal‐Amides,” Angewandte Chemie International Edition 136 (2024): 202402052, 10.1002/anie.202402052. 38705856

[42] C. Murawski , K. Leo , and M. C. Gather , “Efficiency Roll‐Off in Organic Light‐Emitting Diodes,” Advanced Materials 25 (2013): 6801–6827, 10.1002/adma.201301603. 24019178

[43] S. Sudheendran Swayamprabha , D. K. Dubey , Shahnawaz , et al., “Approaches for Long Lifetime Organic Light Emitting Diodes,” Advanced Science 8 (2021): 2002254, 10.1002/advs.202002254. PMC7788592 33437576

[44] Y. Olivier , J. C. Sancho‐Garcia , L. Muccioli , G. D'Avino , and D. Beljonne , “Computational Design of Thermally Activated Delayed Fluorescence Materials: The Challenges Ahead,” The Journal of Physical Chemistry Letters 9 (2018): 6149–6163, 10.1021/acs.jpclett.8b02327. 30265539

[45] D. S. M. Ravinson and M. E. Thompson , “Thermally Assisted Delayed Fluorescence (TADF): Fluorescence Delayed Is Fluorescence Denied,” Materials Horizons 7 (2020): 1210–1217, 10.1039/D0MH00276C.

[46] J. G. Yang , X. F. Song , G. Cheng , et al., “Conformational Engineering of Two‐Coordinate Gold(I) Complexes: Regulation of Excited‐State Dynamics for Efficient Delayed Fluorescence,” ACS Applied Materials & Interfaces 14 (2022): 13539–13549, 10.1021/acsami.2c01776. 35286066

[47] H. J. Wang , Y. Liu , B. Yu , et al., “A Configurationally Confined Thermally Activated Delayed Fluorescent Two‐Coordinate Cu^I^ Complex for Efficient Blue Electroluminescence,” Angewandte Chemie International Edition 62 (2023): 202217195, 10.1002/anie.202217195. 36542446

[48] S. Avula , B. H. Jhun , U. Jo , S. Heo , J. Y. Lee , and Y. You , “Achieving Long‐Wavelength Electroluminescence Using Two‐Coordinate Gold(I) Complexes: Overcoming the Energy Gap Law,” Advanced Science 11 (2024): 2305745, 10.1002/advs.202305745. 37953418 PMC10767458

[49] J. P. Perdew , M. Ernzerhof , and K. Burke , “Rationale for Mixing Exact Exchange with Density Functional Approximations,” The Journal of Chemical Physics 105 (1996): 9982–9985, https://aip.scitation.org/doi/abs/10.1063/1.472933.

[50] C. Adamo and V. Barone , “Toward Reliable Density Functional Methods Without Adjustable Parameters: The PBE0 Model,” The Journal of Chemical Physics 110 (1999): 6158–6170, https://aip.scitation.org/doi/abs/10.1063/1.478522.

[51] S. Grimme , J. Antony , S. Ehrlich , and H. Krieg , “A Consistent and Accurate *Ab Initio* Parametrization of Density Functional Dispersion Correction (DFT‐D) for the 94 Elements H‐Pu,” The Journal of Chemical Physics 132 (2010): 154104, https://aip.scitation.org/doi/abs/10.1063/1.3382344. 20423165 10.1063/1.3382344

[52] S. Grimme , S. Ehrlich , and L. Goerigk , “Effect of the Damping Function in Dispersion Corrected Density Functional Theory,” Journal of Computational Chemistry 32 (2011): 1456–1465, 10.1002/jcc.21759. 21370243

[53] Q. Wan , J. Yang , W. P. To , and C. M. Che , “Strong metal–metal Pauli Repulsion Leads to Repulsive Metallophilicity in Closed‐Shell d^8^ and d^10^ Organometallic Complexes,” Proceedings of the National Academy of Sciences 118 (2021): 2019265118, 10.1073/pnas.2019265118. PMC7817198 33372160

[54] S. Xu , Q. Wan , J. Yang , and C. M. Che , “Anisotropic Metal–Metal Pauli Repulsion in Polynuclear d^10^ Metal Clusters,” The Journal of Physical Chemistry Letters 15 (2024): 2193–2201, 10.1021/acs.jpclett.3c03434. 38373151

[55] M. Menéndez , R. Álvarez Boto , E. Francisco , and Á. M. Pendás , “One‐Electron Images in Real Space: Natural Adaptive Orbitals,” Journal of Computational Chemistry 36 (2015): 833–843, 10.1002/jcc.23861. 25691432

[56] T. Lu and F. Chen , “Multiwfn: A Multifunctional Wavefunction Analyzer,” Journal of Computational Chemistry 33 (2012): 580–592, 10.1002/jcc.22885. 22162017

[57] M. Nooijen and R. J. Bartlett , “A New Method for Excited States: Similarity Transformed Equation‐of‐Motion Coupled‐Cluster Theory,” The Journal of Chemical Physics 106 (1997): 6441–6448, 10.1063/1.474000.

[58] M. Nooijen and R. J. Bartlett , “Similarity Transformed Equation‐of‐Motion Coupled‐Cluster Theory: Details, Examples, and Comparisons,” The Journal of Chemical Physics 107 (1997): 6812–6830, 10.1063/1.474922.

[59] C. Riplinger and F. Neese , “An Efficient and Near Linear Scaling Pair Natural Orbital Based Local Coupled Cluster Method,” The Journal of Chemical Physics 138 (2013): 034106, 10.1063/1.4773581. 23343267

[60] C. Riplinger , B. Sandhoefer , A. Hansen , and F. Neese , “Natural Triple Excitations in Local Coupled Cluster Calculations With Pair Natural Orbitals,” The Journal of Chemical Physics 139 (2013): 134101, 10.1063/1.4821834. 24116546

[61] A. K. Dutta , F. Neese , and R. Izsák , “Towards a Pair Natural Orbital Coupled Cluster Method for Excited States,” The Journal of Chemical Physics 145 (2016): 034102, 10.1063/1.4958734. 27448869

[62] C. Riplinger , P. Pinski , U. Becker , E. F. Valeev , and F. Neese , “Sparse Maps—A Systematic Infrastructure for Reduced‐Scaling Electronic Structure Methods. II. Linear Scaling Domain Based Pair Natural Orbital Coupled Cluster Theory,” The Journal of Chemical Physics 144 (2016): 024109, 10.1063/1.4939030. 26772556

[63] A. K. Dutta , M. Nooijen , F. Neese , and R. Izsák , “Automatic Active Space Selection for the Similarity Transformed Equations of Motion Coupled Cluster Method,” The Journal of Chemical Physics 146 (2017): 074103, 10.1063/1.4976130. 28228040

[64] R. Berraud‐Pache , F. Neese , G. Bistoni , and R. Izsák , “Unveiling the Photophysical Properties of Boron‐dipyrromethene Dyes Using a New Accurate Excited State Coupled Cluster Method,” Journal of Chemical Theory and Computation 16 (2020): 564–575, 10.1021/acs.jctc.9b00559. 31765141

[65] S. Grimme and M. Waletzke , “A Combination of Kohn–Sham Density Functional Theory and Multi‐Reference Configuration Interaction Methods,” The Journal of Chemical Physics 111 (1999): 5645–5655, 10.1063/1.479866.

[66] M. Kleinschmidt , C. M. Marian , M. Waletzke , and S. Grimme , “Parallel Multireference Configuration Interaction Calculations on Mini‐β‐carotenes and β‐carotene,” The Journal of Chemical Physics 130 (2009): 044708, 10.1063/1.3062842. 19191405

[67] I. Lyskov , M. Kleinschmidt , and C. M. Marian , “Redesign of the DFT/MRCI Hamiltonian,” The Journal of Chemical Physics 144 (2016): 034104, 10.1063/1.4940036. 26801017

[68] M. P. Mitoraj , A. Michalak , and T. Ziegler , “A Combined Charge and Energy Decomposition Scheme for Bond Analysis,” Journal of Chemical Theory and Computation 5 (2009): 962–975, 10.1021/ct800503d. 26609605

[69] C. Fonseca Guerra , J. G. Snijders , G. te Velde , and E. J. Baerends , “Towards an Order‐N DFT Method,” Theoretical Chemistry Accounts 99 (1998): 391–403, 10.1007/s002140050353.

[70] G. Te Velde , F. M. Bickelhaupt , E. J. Baerends , et al., “Chemistry with ADF,” Journal of Computational Chemistry 22 (2001): 931–967, 10.1002/jcc.1056.

[71] E. J. Baerends , T. Ziegler , A. J. Atkins , et al., "ADF 2019, SCM, Theoretical Chemistry, Vrije Universiteit, Amsterdam, The Netherlands," http://www.scm.com (2019).

[72] S. Dapprich and G. Frenking , “Investigation of Donor‐Acceptor Interactions: A Charge Decomposition Analysis Using Fragment Molecular Orbitals,” The Journal of Physical Chemistry 99 (1995): 9352–9362, 10.1021/j100023a009.

[73] M. Xiao and T. Lu , “Generalized Charge Decomposition Analysis (GCDA) Method,” Journal of Advances in Physical Chemistry 4 (2015): 111–124, 10.12677/japc.2015.44013.

[74] Y. Tsuchiya , S. Diesing , F. Bencheikh , et al., “Exact Solution of Kinetic Analysis for Thermally Activated Delayed Fluorescence Materials,” The Journal of Physical Chemistry A 125 (2021): 8074–8089, 10.1021/acs.jpca.1c04056. 34473511

[75] L. E. de Sousa and P. de Silva , “Unified Framework for Photophysical Rate Calculations in TADF Molecules,” Journal of Chemical Theory and Computation (2021): 5816–5824, 10.1021/acs.jctc.1c00476. 34383498

[76] R. A. Marcus , “Nonadiabatic Processes Involving Quantum‐Like and Classical‐Like Coordinates With Applications to Nonadiabatic Electron Transfers,” The Journal of Chemical Physics 81 (1984): 4494–4500, 10.1063/1.447418.

[77] S. Scholz , D. Kondakov , B. Lüssem , and K. Leo , “Degradation Mechanisms and Reactions in Organic Light‐Emitting Devices,” Chemical Reviews 115 (2015): 8449–8503, 10.1021/cr400704v. 26230864

[78] E. Tankelevičiūtė , I. D. W. Samuel , and E. Zysman‐Colman , “The Blue Problem: OLED Stability and Degradation Mechanisms,” The Journal of Physical Chemistry Letters 15 (2024): 1034–1047, 10.1021/acs.jpclett.3c03317. 38259039 PMC10839906

