# Supplementary material for: Metal (n+1)p‐nd Orbital Hybridization and Excited‐State Metal–Ligand π‐Interactions Enable d10 Carbene‐Metal‐Amide TADF OLEDs with High Efficiency and Long Operational Lifetime
Source: Adv Sci (Weinh). 2026 Jun 29:e00075. Online ahead of print. doi: 10.1002/advs.202600075 (PMC13337003; doi:10.1002/advs.202600075)
Supplement: Supplementary file 1 — Supporting File: advs76168‐sup‐0001‐SuppMat.pdf. [file ADVS-9999-e00075-s001.pdf]

Supporting Information

Metal (n+1)p-nd Orbital Hybridization and Excited-State Metal-Ligand π-Interactions Enable d^{10} Carbene-Metal-Amide TADF OLEDs with High Efficiency and Long Operational Lifetime

Shuo Xu, Rui Tang, Qingyun Wan, Gang Cheng, Jun Yang, Chi-Ming Che**

Table of Contents

S1. Experimental Section 7

S1.1. General experimental section 7

S1.1.1. Chemicals and instrumentations 7

S1.1.2. Crystal structure determination 7

S1.1.3. Photophysical measurements 8

S1.1.4. OLED device fabrication and characterization 8

S1.2. Molecular structures 11

S1.3. Synthesis and characterizations 12

S1.4. Crystal structure determination 20

S1.5. Electrochemical properties 22

S1.6. Photophysical measurements 24

S1.7. Electroluminescent properties 26

S2. Computational Section 29

S2.1. Computational details 29

S2.1.1. General computational details 29

S2.1.1.1. DFT calculations 29

S2.1.1.2. CCSD calculations 30

S2.1.1.3 DFT/MRCI calculations 31

S2.1.2. Functional validation 31

S2.1.3. Plotting of potential energy surfaces (PES) for excited states 31

S2.1.3.1. TDDFT method 31

S2.1.3.2. STEOM-DLPNO-CCSD method 32

S2.1.3.3. DFT/MRCI method 32

S2.1.4. NAdO analysis 33

S2.1.5. ETS-NOCV analysis 33

S2.1.6. CDA analysis 36

S2.1.7. Calculation of rate constants k_{S1}, k_{T1}, k_{ISC}, k_{rISC}, and k_{TADF} 37

S2.2. Additional results and discussion 40

S2.2.1. Excited state potential energy surfaces (PES) and dihedral angle rotation flexibility of emitters 40

S2.2.2. The metal relativistic effects on metal hybridization, metal-ligand interactions, and the rotational flexibility of emitters 42

S2.2.3. Charge decomposition analysis 42

S2.2.4. Further discussions on metal-ligand π-interaction and dihedral angle rotation 44
S2.2.5. Reasons for the differences in the calculated radiative decay rate constants of symmetric rotational isomers 44
S2.3. Calculation results 46
S2.3.1. Structural properties 46
S2.3.2. Excited state potential energy surfaces (PES) 51
S2.3.3. Molecular orbital interaction diagrams 55
S2.3.4. Electronic configuration analysis 56
S2.3.5. Canonical molecular orbital analysis 58
S2.3.6. NAdO analysis 61
S2.3.7. ETS-NOCV analysis 75
S2.3.8. CDA analysis 79
S2.3.9. Electron-hole analysis 81
S2.3.10. Calculation of excited state energies and radiative rate constants of emitters 82
S2.3.11. Topological analysis and buried volume calculations 94
S2.3.12. Coordinates 95
References 148

Figure S1. Molecular structures of CMA TADF emitters 11
Figure S2-S3. ^{1}H and ^{13}C NMR spectra of **PzIPrAgCl**. 13
Figure S4-S5. ^{1}H and ^{13}C NMR spectra of **Ag-1**. 15
Figure S6-S8. ^{1}H, ^{19}F, and ^{13}C NMR spectra of **Au-1^{2CF3}**. 16
Figure S9-S10. ^{1}H and ^{13}C NMR spectra of **Au-1^{2tBu}**. 18
Figure S11-S12. ^{1}H and ^{13}C NMR spectra of **Au-1^{2OMe}**. 19
Figure S13. Crystal structures of **Au-1^{2CF3}** and **Au-1^{2tBu}**. 20
Table S1. Key crystal structure parameters of CMA emitters. 20
Table S2. Selected crystallographic data of **Au-1^{2CF3}** and **Au-1^{2tBu}** 21
Table S3. Summary of electrochemical results of **Au-1^{2CF3}**, **Au-1^{2tBu}**, and **Au-1^{2OMe}**. 22
Figure S14. Cyclic voltammograms and differential pulse voltammograms of **Au-1^{2CF3}**, **Au-1^{2tBu}**, and **Au-1^{2OMe}** 23
Table S4. Photophysical properties of emitters **Au-1** and **Ag-1** 24
Figure S15. Absorption spectra and emission spectra of emitters. 25

Figure S16 (a) Variable temperature lifetime measurement and the corresponding Arrhenius plots of **Ag-1** thin film. 25
Figure S17. Characteristics of CMA(Au) OLEDs. 26
Figure S18. Characteristics of CMA(Cu) OLEDs. 27
Table S5. Key device parameters of CMA OLEDs. 28
Figure S19. Optimized geometries of emitters **M-2** and **M-3**. 46
Table S6. Comparison of X-ray crystal structures and DFT optimized structures of **M-1**, **M-2**, and **M-3**. 47
Figure S20. M-C and M-N bond lengths in X-ray crystal structures and optimized structures of **M-1**, **M-2**, and **M-3**. 48
Figure S21. Calculated Mayer bond order of M-C and M-N bonds in **M-1**, **M-2**, and **M-3**. 49
Figure S22. Top view of the optimized geometries of **Au-2** with $\theta_{C3\text{-}C1\text{-}N2\text{-}C3}$ fixed at 0º and 180º. 49
Figure S23. Resonance structures of the carbazole ligand. 50
Figure S24. TDDFT calculated excited-state PES of **M-1** and STEOM-DLPNO-CCSD calculated excited-state PES of **M-1'** 51
Figure S25. DFT/MRCI calculated excited-state PES of **M-1'** 51
Table S7. $\angle_{C1\text{-}Cu\text{-}N1}$ angle and $\theta_{N1\text{-}C1\text{-}N2\text{-}C3}$ dihedral angle in relaxed geometries of **Cu-1** with fixed $\theta_{N1\text{-}C1\text{-}N2\text{-}C2}$ dihedral angle. 52
Figure S26. TDDFT calculated excited-state PES of **Au-1'** in toluene solution and in vacuo 52
Figure S27. Potential energy surfaces of **M-2** and **M-3**. 53
Figure S28. Calculated potential energy surfaces of **M-1** without consideration of relativistic effects. 53
Figure S29. Schematic diagram of potential energy curves of S_0, S_1, and T_1 electronic states and reorganization energies λ_S and λ_T. 54
Figure S30. Interaction diagram of σ(M-L) bond foramtion and diagrams showing (n+1)s-nd hybridization. 55
Table S8. Relativistic and nonrelativistic calculations for electronic configurations of metal in **M-1**. 56
Table S9. Calculated metal electronic configurations in emitters with substituents on the carbazole ligand of **M-1**. 57
Table S10. Calculated composition of metal (n+1)p orbital in HOMO of emitters **M-1**, **M-2**, and **M-3**. 58
Table S11. Calculated composition of metal (n+1)p orbital in HOMO of emitters with substituents on the carbazole ligand of **Au-1**. 59

Figure S31. Frontier orbitals and HOMO-LUMO overlap integrals in semi-coplanar and orthogonal geometries of **M-1**, **M-2**, and **M-3**.............................60

Figure S32. Sum of NAdOs representing the π(M···N) interaction for **Au-1** and for **Au-1** with electron-withdrawing and electron-donating substituents on the carbazole ligand. 61

Figure S33. Sum of NAdOs representing π(M···C) interaction or the π(M···N) interaction for **M-1** in twist geometries with various dihedral angles in the T_1 state. 61

Figure S34-S36. Major NAdOs of M-C and M-N bonds for emitters **M-1**, **M-2**, and **M-3** in optimized geometries in different electronic states. 63

Figure S37-S39. Major NAdOs of M-C and M-N bonds for emitters with substituents on the carbazole ligand in optimized geometries in different electronic states 69

Table S12. Eigenvalues of NAdOs representing M-C and M-N σ- and π-interactions in CMA emitters in semi-coplanar and orthogonal geometries........73

Table S13. Eigenvalues of NAdOs representing M-C and M-N σ- and π-interactions in **M-1** in twisted geometries. 74

Figure S40. Major ETS-NOCV deformation density contributions to M-C/M-N bonds of **Ag-1**, **Cu-1**, **M-2** and **M-3**. 76

Figure S41. Calculated orbital energies of major ETS-NOCV deformation densities representing π(M···C) [$\Delta E_{\mathrm{Orb}}(2) + \Delta E_{\mathrm{Orb}}(6)$] and π(M···N) [$\Delta E_{\mathrm{Orb}}(3)$] interactions for **M-1** in twisted geometries with various dihedral angles in the T_1 excited state...76

Table S14. Orbital energies of major ETS-NOCV deformation densities of **M-1**, **M-2**, and **M-3**. 77

Table S15. Orbital energies of major ETS-NOCV deformation densities of **M-1** in twisted geometries with various dihedral angles. 78

Figure S42. Calculated electron transfer from metal to carbazole ligand for **M-1**, **M-2**, and **M-3**. 79

Table S16. CDA results of emitters **M-1**, **M-2**, and **M-3**..................................80

Figure S43. Electron-hole plots of **Au-1** and emitters with substituents on the carbazole ligand of **Au-1**. 81

Table S17. Electron-hole analysis results of **Au-1**, **Cu-1**, and emitters with substituents on the carbazole ligand. 81

Table S18. Calculated $E_{\mathrm{S1},\theta}$, $E_{\mathrm{T1},\theta}$, $\Delta E_{\mathrm{S1\text{-}T1},\theta}$, $f_{\mathrm{S1}\rightarrow\mathrm{S0},\theta}$, $\lambda_{\mathrm{T},\theta}$, $\lambda_{\mathrm{S},\theta}$, $|\langle\psi_{\mathrm{S1}}|\hat{H}_{\mathrm{SO}}|\psi_{\mathrm{T1}}\rangle|$, $|\langle\psi_{\mathrm{T1}}|\hat{H}_{\mathrm{SO}}|\psi_{\mathrm{T1}}\rangle|$, $k_{\mathrm{S1},\theta}$, $k_{\mathrm{T1},\theta}$, $k_{\mathrm{ISC},\theta}$, $k_{\mathrm{rISC},\theta}$, $k_{\mathrm{TADF},\theta}$, and k_{TADF} values for **M-1**, **M-2**, and **M-3**...82

Table S19 . STEOM-DLPNO-CCSD calculated $E_{\mathrm{S1},\theta}$, $E_{\mathrm{T1},\theta}$, $\Delta E_{\mathrm{S1\text{-}T1},\theta}$, $E_{\mathrm{S1(v)},\theta}$, $E_{\mathrm{T1(v)},\theta}$, $f_{\mathrm{S1}\rightarrow\mathrm{S0},\theta}$, and nature of transitions of **M-1'**...88

Table S20. DFT/MRCI calculated $E_{S1,\theta}$, $E_{T1,\theta}$, $\Delta E_{S1\text{-}T1,\theta}$ of emitter **M-1'**. 90

Table S21. Literature-reported TADF emission wavelength of CMA emitters...91

Table S22. Lowest excited states of CMA emitters..92

Table S23. Comparison between emission energies for the emitters **M-1**, **M-2**, and **M-3** measured experimentally and calculated theoretically 93

Figure S44. Calculated topological steric maps with buried volume (%V_{bur}) of **Au-1^{2CF3}** and **Au-1^{2CN}** 94

S1. Experimental Section

S1.1. General experimental section

S1.1.1. Chemicals and instrumentations

All chemicals were purchased unless otherwise specified. Reagent-grade solvents were used for synthesis. Synthesis of N-heterocyclic carbene (NHC) ligand precursors PzIPr-HCl, **Cu-1**, **Cu-1$^{2t\text{Bu}}$**, **Cu-1^{FLR}**, and **Au-1**, **Au-1^{CN}**, **Au-1$^{\text{2CN}}$** were based on previously reported methods (see Section S1.3 for chemical structure of precursors and emitters).[1-3] For photophysical measurements, HPLC-grade solvents were used. ^{1}H, ^{13}C, and ^{19}F NMR spectra were recorded on a Bruker Avance 400, DRX-500, or Avance 600 NMR spectrometer. All chemical shifts (δ) were reported in ppm; the chemical shifts were calibrated with the corresponding solvent residual peaks. High-resolution electrospray ionization (HR-ESI) mass spectra were recorded on Waters Micromass Q-Tof Premier or Bruker maXis II high resolution Q-Tof mass spectrometer.

S1.1.2. Crystal structure determination

A Bruker D8 VENTURE Duo FIXED-CHI X-ray diffractometer was used for crystal screening, unit cell determination, and data collection for the X-ray crystal structures. Crystals suitable for X-ray diffraction were mounted on a MiTeGen dual-thickness micro-mounts and placed under a cold nitrogen stream (Oxford). IµS (Mo Kα = 0.71073 Å, 50 kV, 0.8 mA) X-ray source was used. Bruker AXS APEX3 (v2018.7-2) software suite was used for data collection and reduction. Absorption corrections were applied using the program SADABS. Structure solutions were obtained using XT and refined by XL in APEX3. Hydrogen atoms were placed in idealized positions and were set riding on the respective parent atoms. All non-hydrogen atoms were refined with anisotropic thermal parameters. The structure was refined by weighted least squares refinement on F^2 to convergence. The X-ray crystallographic coordinates for structures

reported in this article have been deposited at Cambridge Crystallographic Data Centre (CCDC), under deposition number CCDC 2477707 for **Au-1^{2CF3}**, CCDC 2477706 for **Au-1^{2tBu}**. These data can be obtained free of charge from CCDC via https://www.ccdc.cam.ac.uk/structures/.

S1.1.3. Photophysical measurements

Ultraviolet-visible light (UV-vis) absorption spectra were recorded on a Hewelett-Packard 8453 diode array spectrophotometer. The spectra were generally obtained with 2×10^{-5} M solutions unless specified. Steady-state emission and excitation spectra of samples in solution, or glassy state were recorded on a SPEX Fluorolog 3 spectrofluorometer. For measurements in solution at room temperature, samples were loaded into two-compartment cells consisting of a 10 mL Pyrex bulb and a quartz cuvette with a 1-cm path length. The cells were sealed from the atmosphere with Rotaflo stopcocks. Solutions were degassed five times with a high-vacuum line by freeze-thaw-pump cycles. Absolute emission quantum yields of solutions and thin-film samples were recorded on a Hamamatsu Quantaurus-QY Absolute PL quantum yields measurement system C11347. Thin-film samples were prepared by drop-cast of a complex with 1,3-bis(N-carbazolyl)benzene (mCP) in toluene onto clean quartz plates, and then evaporated to dryness at 60 °C. Emission lifetimes (τ) were measured with a Quanta Ray GCR 150-10 pulsed Nd:YAG laser system (pulse λ_{exc} = 355 nm).

S1.1.4. OLED device fabrication and characterization

Indium-tin-oxide (ITO) coated glass with a sheet resistance of 10 Ω/sq was used as the anode substrate. Before film deposition, patterned ITO substrates were cleaned with detergent, rinsed in de-ionized water, acetone, and isopropanol, and then dried in an oven for 1 h in a cleanroom. The slides were then treated in an ultraviolet-ozone chamber for 5 min. The organic light-emitting diodes (OLEDs) were fabricated in a Kurt J. Lesker SPECTROS vacuum deposition system with a base pressure of 10^{-7} mbar.

In the vacuum chamber, organic materials were thermally deposited in sequence at a rate of 0.5 Å s^{-1}. The doping process in the emissive layers (EMLs) was performed using co-deposition. Afterward, Yb (1.0 nm)/Ag (100 nm) were thermally deposited at rates of 0.01 and 0.2 nm s^{-1}, respectively. The film thicknesses were determined in situ with calibrated oscillating quartz-crystal sensors. Current density-brightness-voltage characteristics, electroluminescence (EL) spectra, and external quantum efficiency (EQE) of EL devices were obtained by using a Keithley 2400 source-meter and an absolute external quantum efficiency measurement system (C9920-12, Hamamatsu Photonics). All devices were encapsulated in a 200-nm-thick Al_2O_3 thin film deposited by atomic layer deposition (ALD) in a Kurt J. Lesker SPECTROS ALD system before measurements.

Device structure for Au complexes

ITO/BCFN: NDP-9 (3 wt%, 10 nm)/BCFN (80 nm)/SiCzCz (5 nm)/Au emitter: SiCzCz: $SiTrzCz_2$ (2 wt%, 40 nm)/mSiTrz (10 nm)/mSiTrz: Liq (35 nm)/ Yb (1 nm)/Ag (100 nm)

Device structure for Cu complexes

ITO/FSFA: NDP-9 (3 wt%,10 nm)/FSFA (120 nm)/NBP-BC (5 nm)/Cu emitter: NBP-BC: PCPF-Trz (4 wt%, 40 nm)/ANT-Biz: Liq (30 nm)/Yb (1 nm)/Ag (100 nm)

Chemical structures of auxiliary organic materials used in this work:

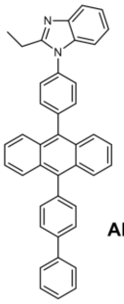

ANT-Biz

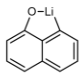

Liq

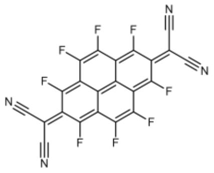

NDP-9

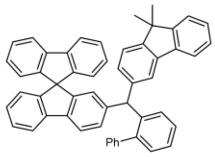

FSFA

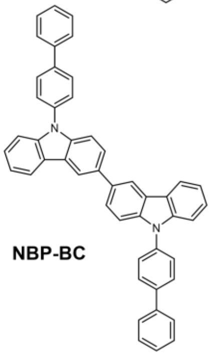

NBP-BC

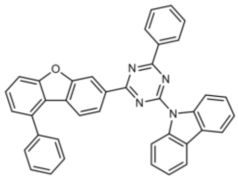

PCPF-Trz

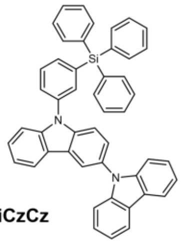

SiCzCz

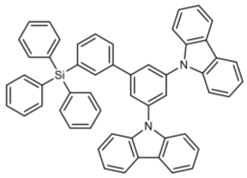

$SiTrzCz_2$

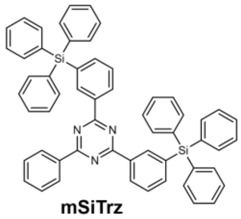

mSiTrz

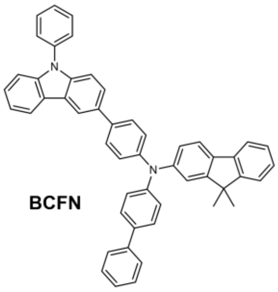

BCFN

S1.2. Molecular structures

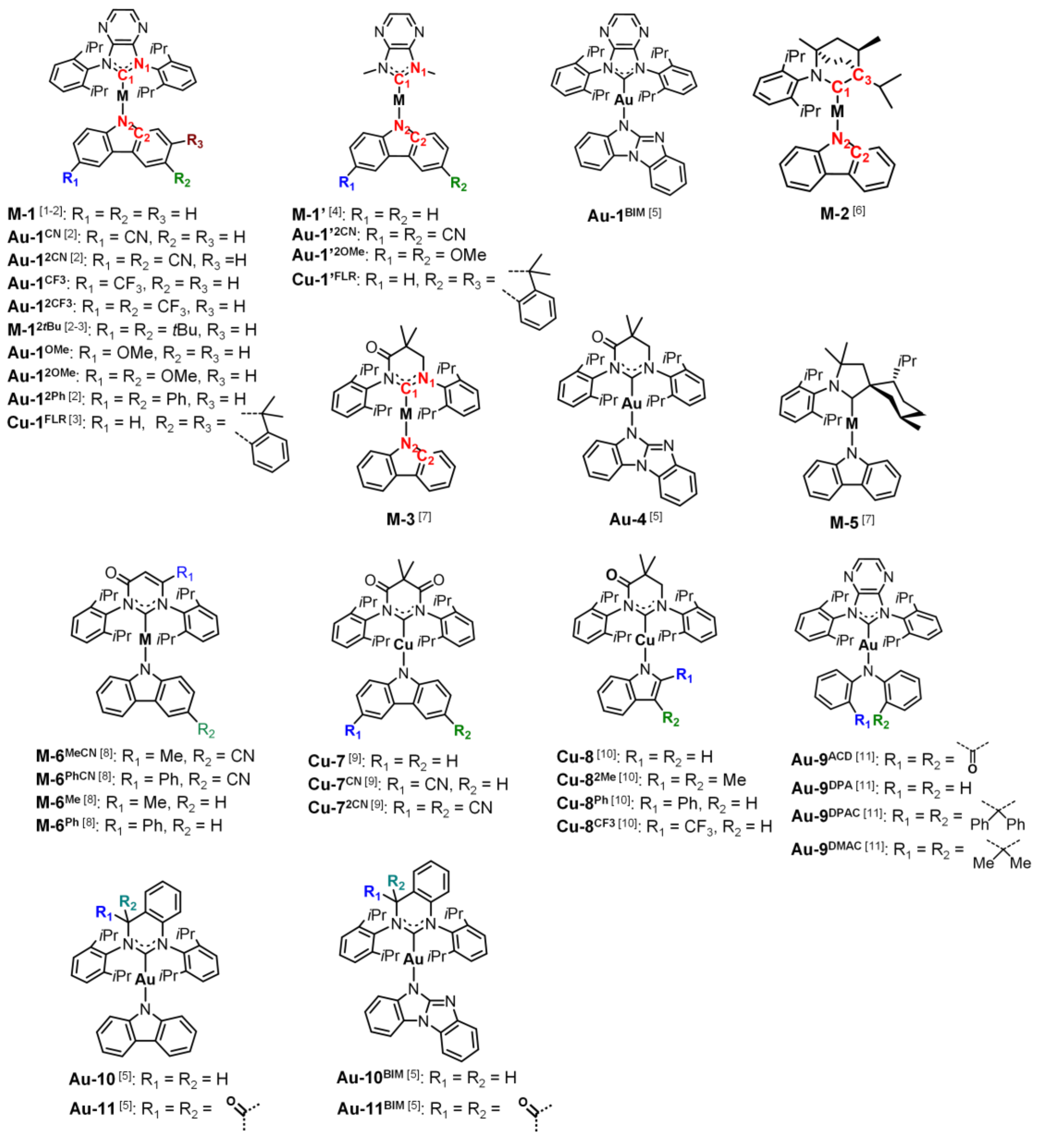

Figure S1. Molecular structures of carbene-metal-amide (CMA) thermally activated delayed fluorescence (TADF) emitters. M = Ag(I), Au(I), and Cu(I).

S1.3. Synthesis and characterizations

Synthesis of PzIPrAgCl:

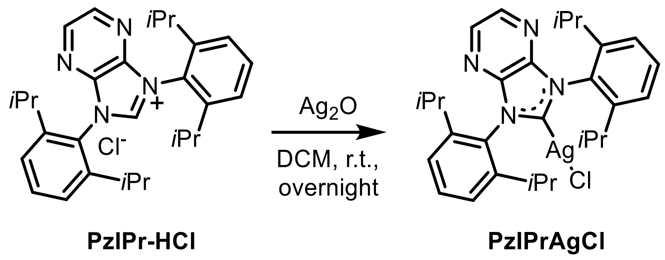

To a solution of PzIPr-HCl (100 mg, 0.21 mmol, 1.0 eq.) in CH_2Cl_2 (10 mL) was added Ag_2O (49 mg, 1.0 eq.). The resulting suspension was stirred in the dark at room temperature overnight. After reaction, the reaction mixture was filtered through a pad of Celite, and the filtrate was evaporated to dryness. The residue was recrystallized from CH_2Cl_2/EtOH to give a pale yellow crystalline solid as product. Yield: 56 mg, 46%. ^1H NMR (500 MHz, $CDCl_3$) δ/ppm 8.56 (s, 1H), 7.63 (t, J = 7.7 Hz, 1H), 7.42 (d, J = 7.8 Hz, 2H), 2.31 (dt, J = 13.5, 6.8 Hz, 3H), 1.28 (d, J = 6.8 Hz, 7H), 1.11 (d, J = 6.7 Hz, 8H). ^{13}C NMR (126 MHz, $CDCl_3$) δ/ppm 146.32, 141.47, 140.12, 131.97, 130.65, 125.06, 29.60, 24.86, 23.96. HR ESI-MS: $[M - Cl + MeCN]^+$ for $[C_{31}H_{39}N_5Ag]^+$, cal. m/z 588.2251, found at m/z 588.2231.

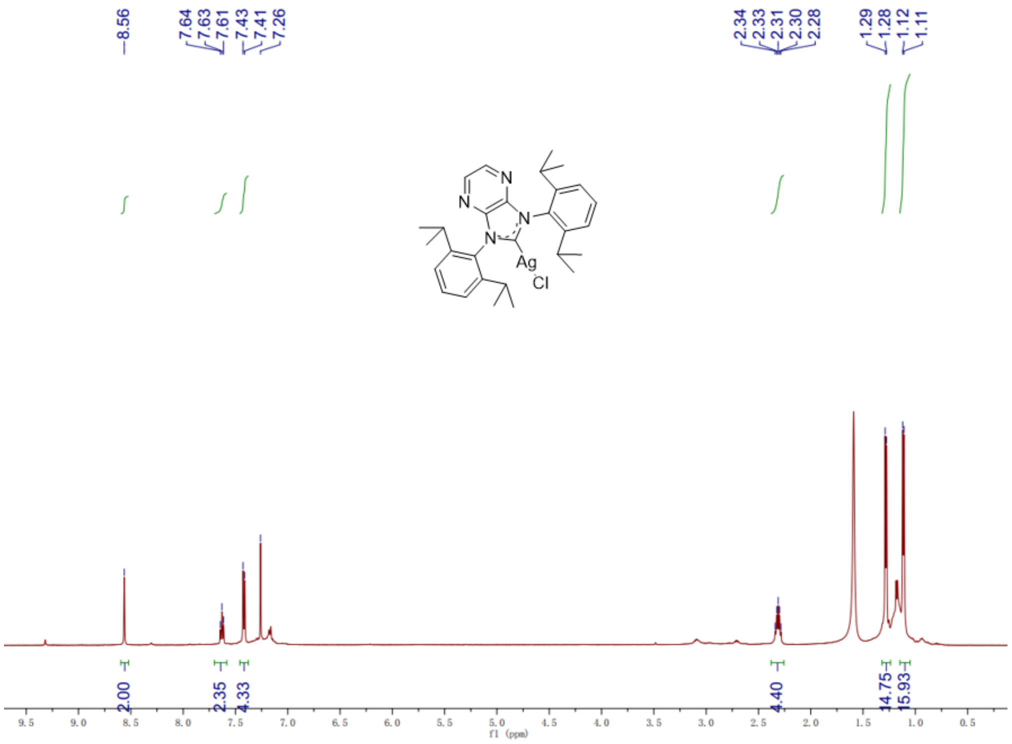

Figure S2. ^{1}H NMR spectrum of **PzIPrAgCl** (500 MHz, $CDCl_3$).

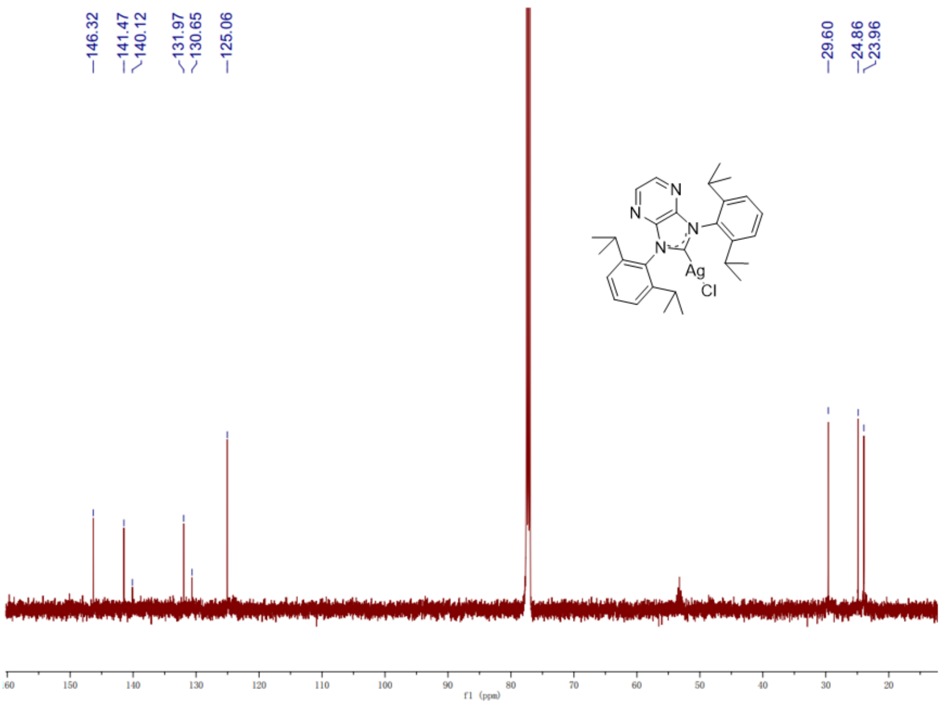

Figure S3. ^{13}C NMR spectrum of **PzIPrAgCl** (126 MHz, $CDCl_3$).

General procedure for synthesis of CMA emitters:

To a solution of amide (1.2 eq.) in tetrahydrofuran (THF) was added NaO*t*Bu (1.5 eq.) and the mixture was stirred for 30 min at room temperature under argon. Then NHC-M-Cl (1.0 eq.; M = Ag or Au) was added and the reaction mixture was stirred overnight. After reaction, the mixture was passed through a pad of celite. The filtrate was evaporated to dryness and the residue was recrystallized with CH_2Cl_2/MeOH to give the desired product.

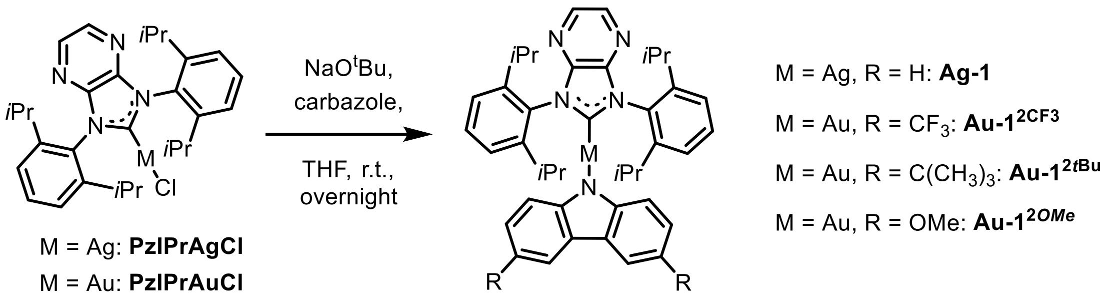

Ag-1: Yield: 36 mg, 59%. ^{1}H NMR (500 MHz, CD_2Cl_2) δ/ppm 8.61 (s, 2H), 7.87 (d, *J* = 7.5 Hz, 2H), 7.75 (t, *J* = 7.7 Hz, 2H), 7.53 (d, *J* = 7.7 Hz, 4H), 7.02 (t, *J* = 7.4 Hz, 2H), 6.84 (t, *J* = 7.2 Hz, 2H), 6.57 (d, *J* = 8.0 Hz, 2H), 2.46 (dt, *J* = 13.1, 6.5 Hz, 4H), 1.30 (d, *J* = 6.6 Hz, 12H), 1.15 (d, *J* = 6.6 Hz, 12H). ^{13}C NMR (126 MHz, CD_2Cl_2) δ/ppm 150.27, 146.80, 141.55, 140.18, 131.69, 131.15, 124.93, 123.82, 123.41, 119.25, 115.08, 114.10, 29.57, 24.48, 23.71. Note: the ^{13}C signal for $C_{carbene}$ was not observed.

Au-1^{2CF3}: Yield: 92 mg, 66%. ^{1}H NMR (600 MHz, Acetone-d_6) δ = 8.79 (s, 2H), 8.46 (s, 2H), 7.92 (t, *J*=7.9, 2H), 7.71 (d, *J*=7.9, 4H), 7.39 (dd, *J*=8.5, 1.4, 2H), 6.79 (d, *J*=8.5, 2H), 2.74 – 2.65 (m, 4H), 1.37 (d, *J*=6.9, 12H), 1.18 (d, *J*=6.8, 12H). ^{19}F NMR (565 MHz, Acetone-d_6) δ = -59.89. ^{13}C NMR (151 MHz, Acetone-d_6) δ = 190.17, 152.67, 148.17, 143.12, 141.46, 132.61, 131.92, 129.62, 127.83, 126.04, 125.78, 124.24, 122.09, 122.07, 119.98, 119.77, 119.56, 119.35, 118.69, 118.66, 114.83, 24.74, 24.36. HR ESI-MS: $[M + H]^+$ for $[C_{43}H_{42}N_5F_6Au]^+$, cal. m/z 940.3083, found at *m/z* 940.3068.

Au-1^{2tBu}: Yield: 76 mg, 56%. ^{1}H NMR (400 MHz, Acetone-d$_6$) δ = 8.73 (s, 2H), 7.93 (d, *J*=1.8, 2H), 7.86 (t, *J*=7.8, 2H), 7.66 (d, *J*=7.8, 4H), 7.08 (dd, *J*=8.5, 1.9, 2H), 6.63 (d, *J*=8.5, 2H), 2.68 (dt, *J*=13.6, 6.8, 4H), 1.38 (d, *J*=6.8, 12H), 1.34 (s, 18H), 1.16 (d, *J*=6.8, 12H). ^{13}C NMR (151 MHz, Acetone-d$_6$) δ = 192.23, 148.90, 148.04, 142.67, 141.59, 139.28, 132.35, 132.00, 125.62, 124.85, 122.05, 115.89, 113.74, 35.02, 32.67, 24.72, 24.32. HR ESI-MS: $[M + H]^+$ for $[C_{49}H_{60}N_5Au]^+$, cal. m/z 916.4587, found at *m/z* 916.4578.

Au-1^{2OMe}: Yield: 43 mg, 33%. ^{1}H NMR (400 MHz, CD_2Cl_2) δ = 8.59 (s, 2H), 7.80 (t, *J*=7.8, 2H), 7.56 (d, *J*=7.8, 4H), 7.33 (s, 2H), 6.69 (d, *J*=9.0, 2H), 6.49 (d, *J*=8.7, 2H), 3.82 (s, 6H), 2.50 (dt, *J*=13.7, 6.8, 4H), 1.34 (d, *J*=6.8, 12H), 1.16 (d, *J*=6.8, 12H). ^{13}C NMR (151 MHz, CD_2Cl_2) δ = 192.36, 152.21, 147.50, 145.47, 141.86, 140.98, 132.05, 131.15, 125.28, 123.95, 114.63, 113.96, 102.08, 56.48, 30.14, 24.58, 24.31. HR ESI-MS: $[M + H]^+$ for $[C_{43}H_{48}N_5O_2Au]^+$, cal. m/z 864.3546, found at *m/z* 864.3523.

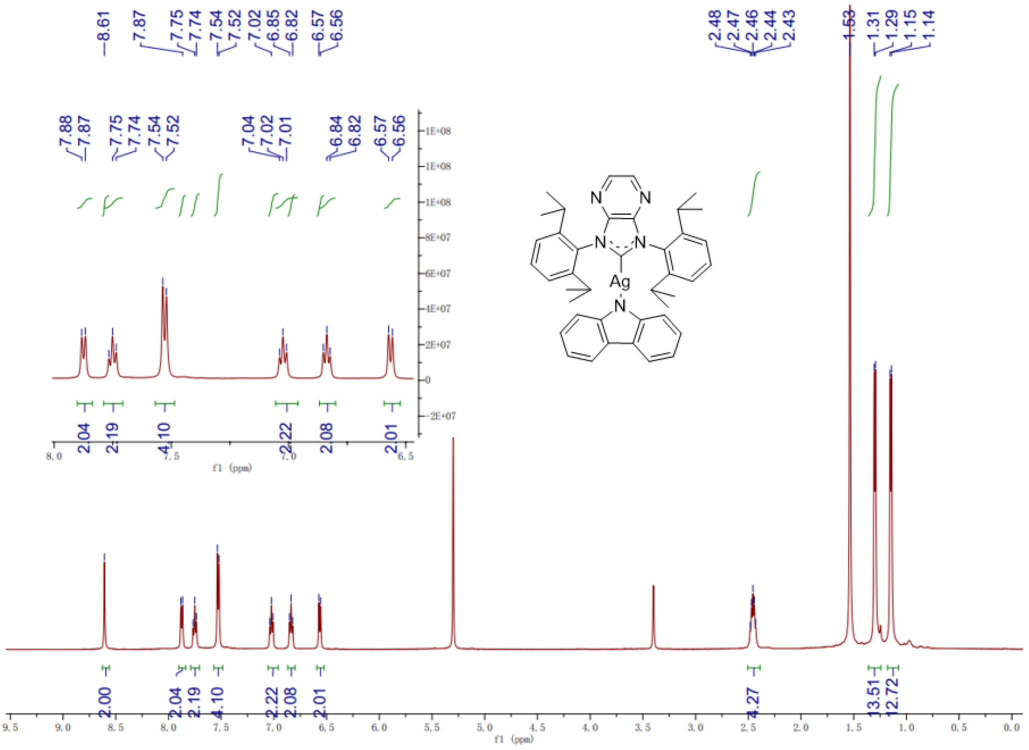

Figure S4. ^{1}H NMR spectrum of **Ag-1** (500 MHz, CD_2Cl_2).

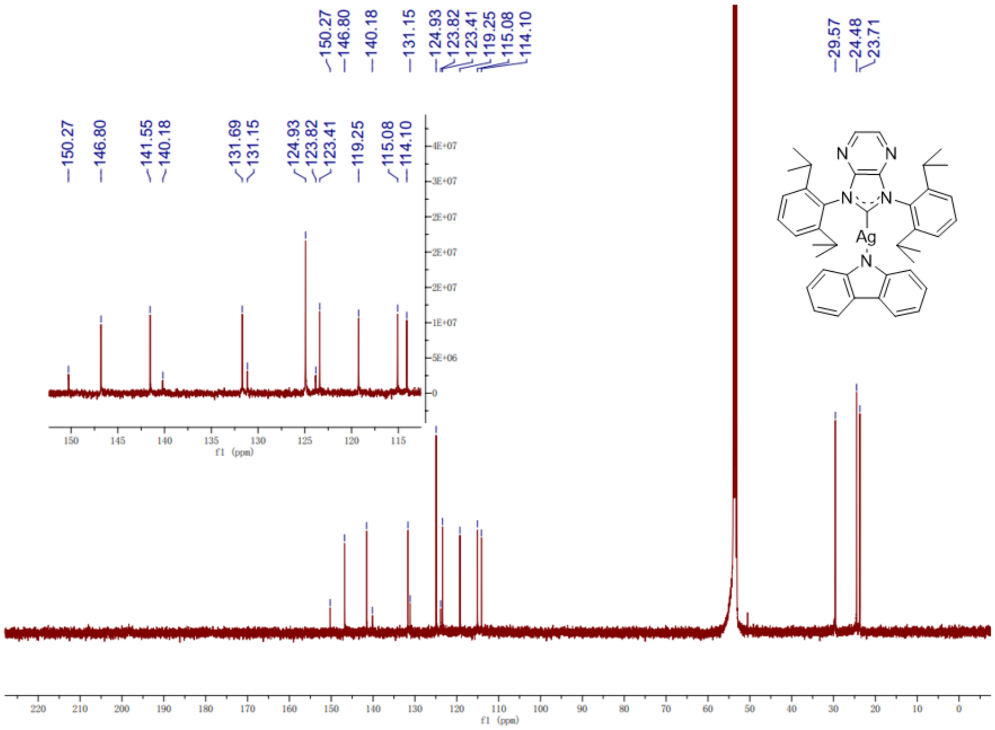

Figure S5. ^{13}C NMR spectrum of **Ag-1** (126 MHz, CD_2Cl_2).

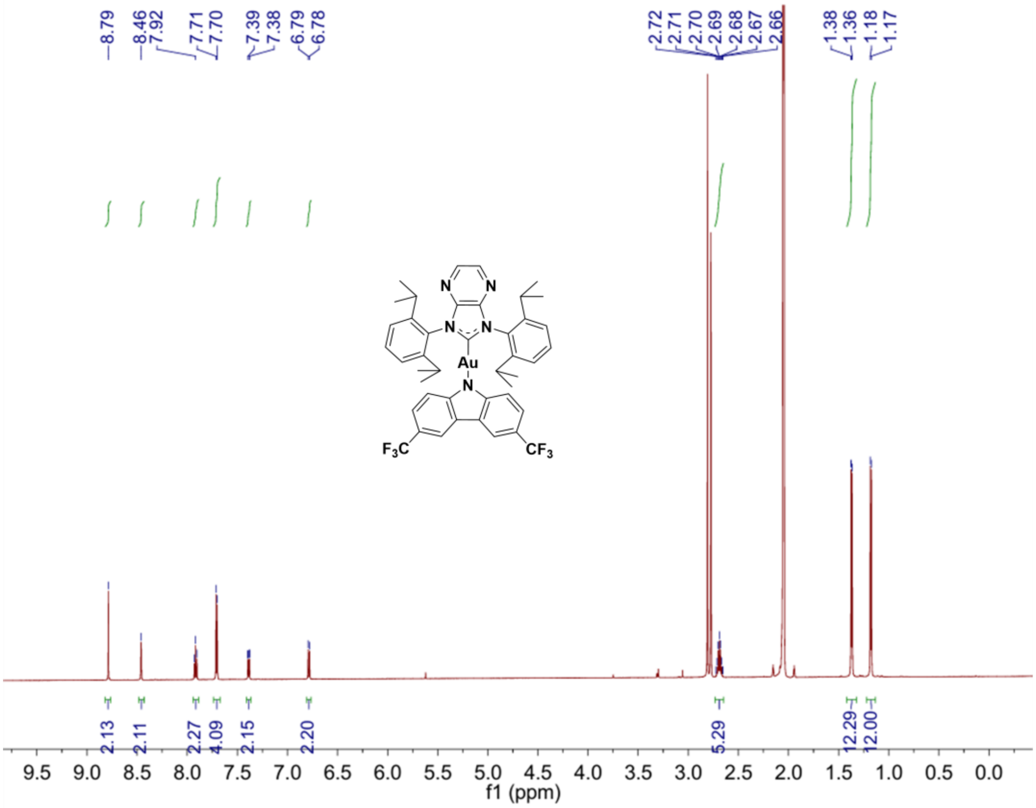

Figure S6. ^{1}H NMR spectrum of **Au-1^{2CF3}** (600 MHz, acetone-d_6).

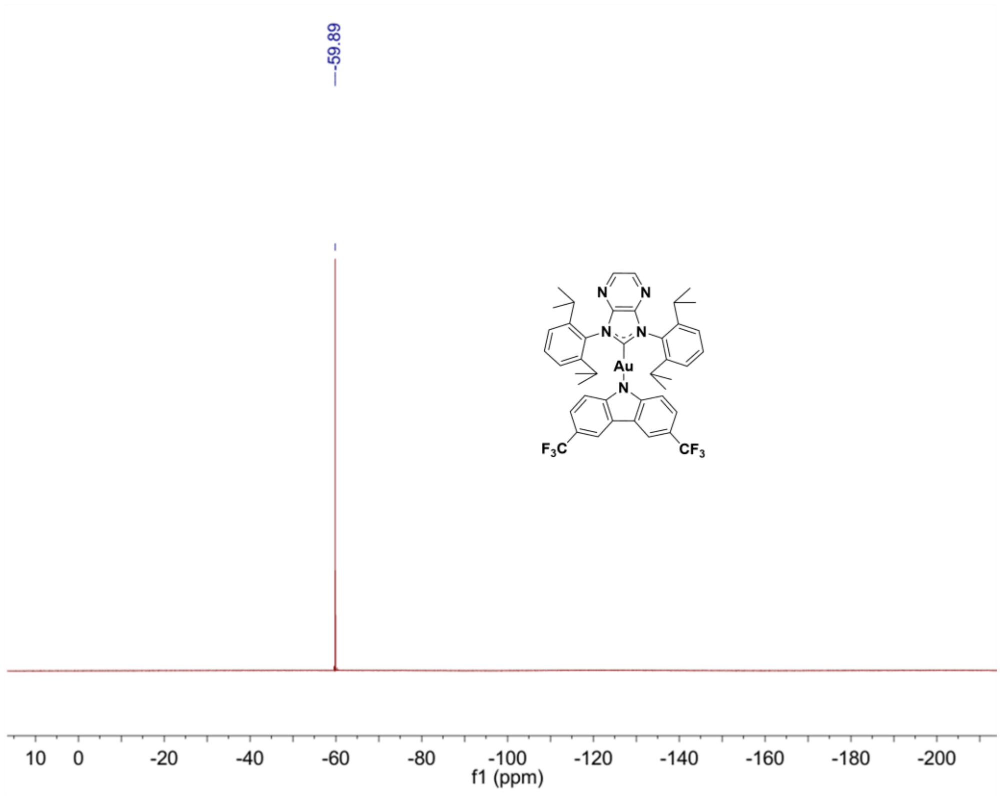

Figure S7. ^{19}F NMR spectrum of **Au-1^{2CF3}** (565 MHz, acetone-d$_6$).

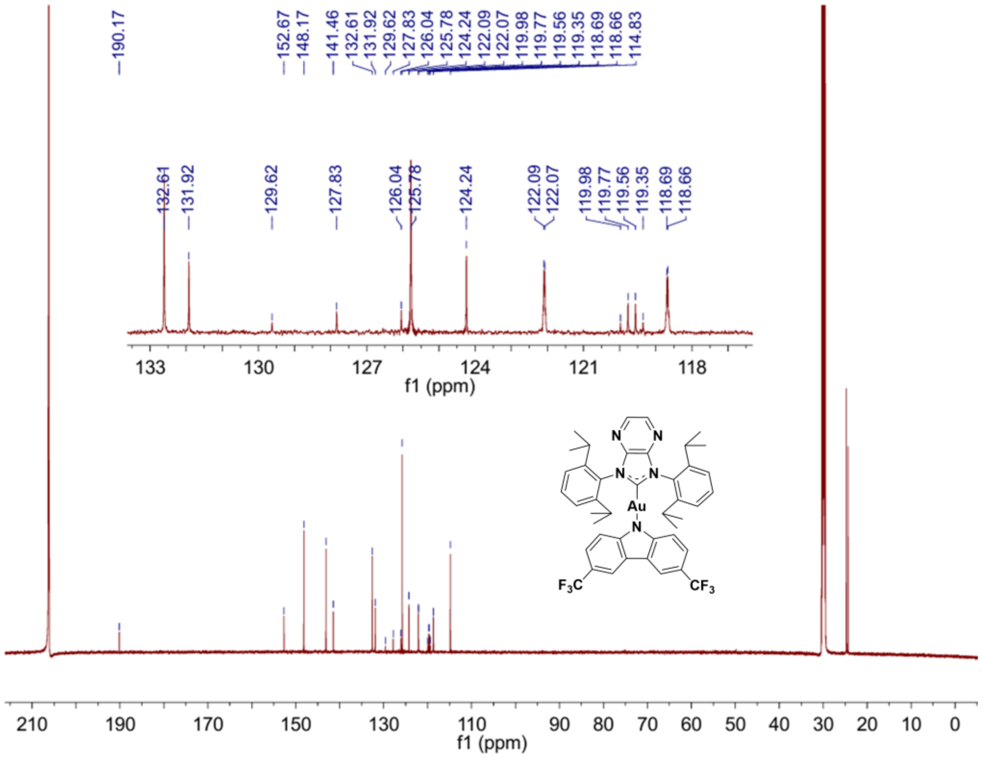

Figure S8. ^{13}C NMR spectrum of **Au-1^{2CF3}** (151 MHz, acetone-d$_6$).

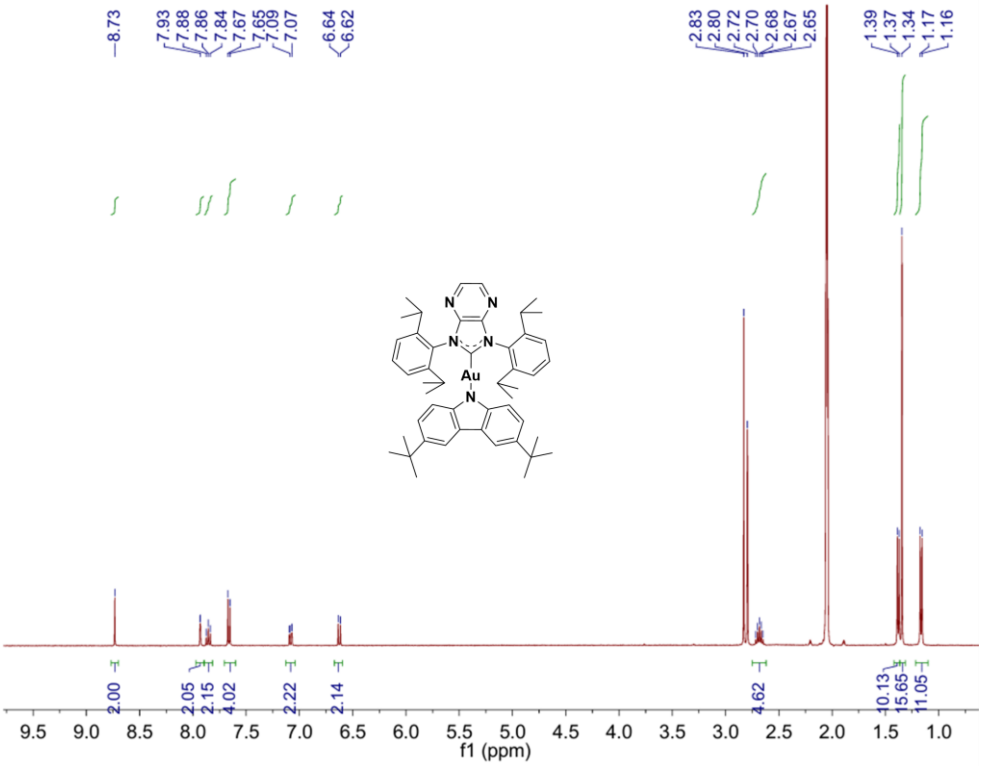

Figure S9. ^{1}H NMR spectrum of **Au-1^{2tBu}** (400 MHz, acetone-d_6).

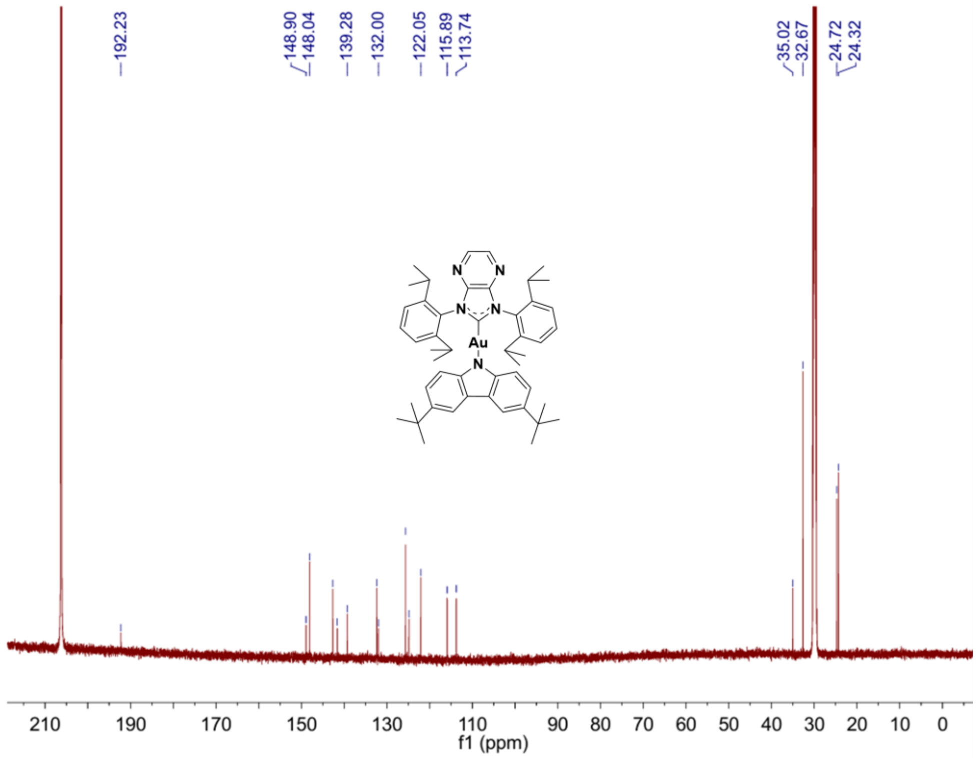

Figure S10. ^{13}C NMR spectrum of **Au-1^{2tBu}** (151 MHz, acetone-d_6).

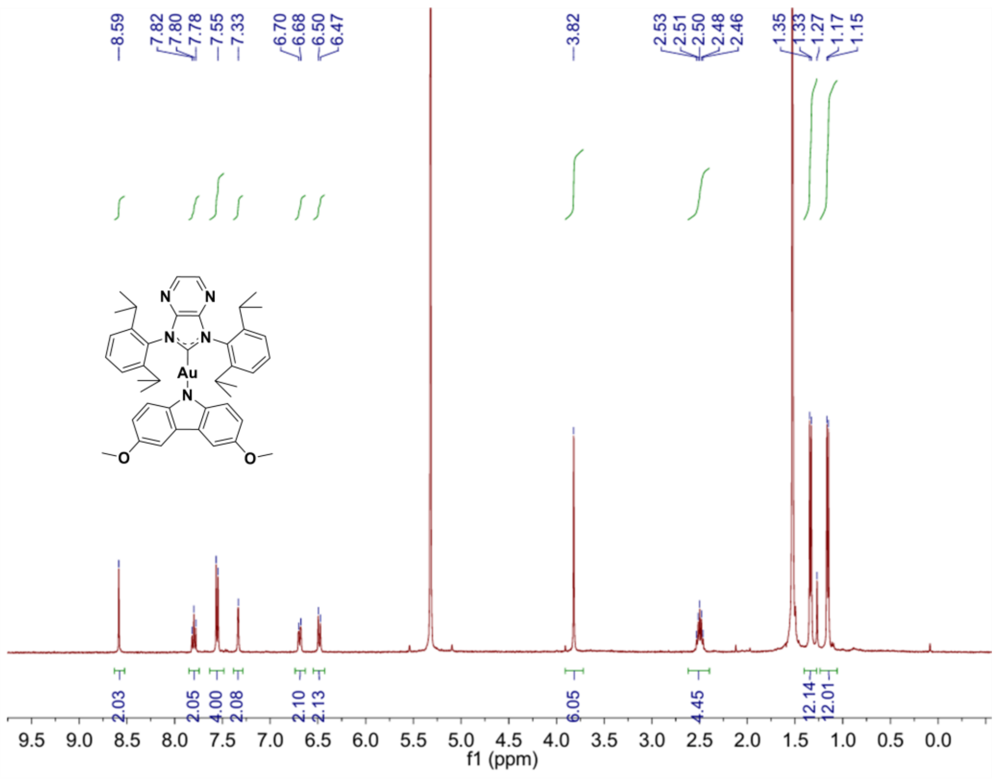

Figure S11. ^{1}H NMR spectrum of **Au-1$^{\mathbf{2OMe}}$** (400 MHz, CD_2Cl_2).

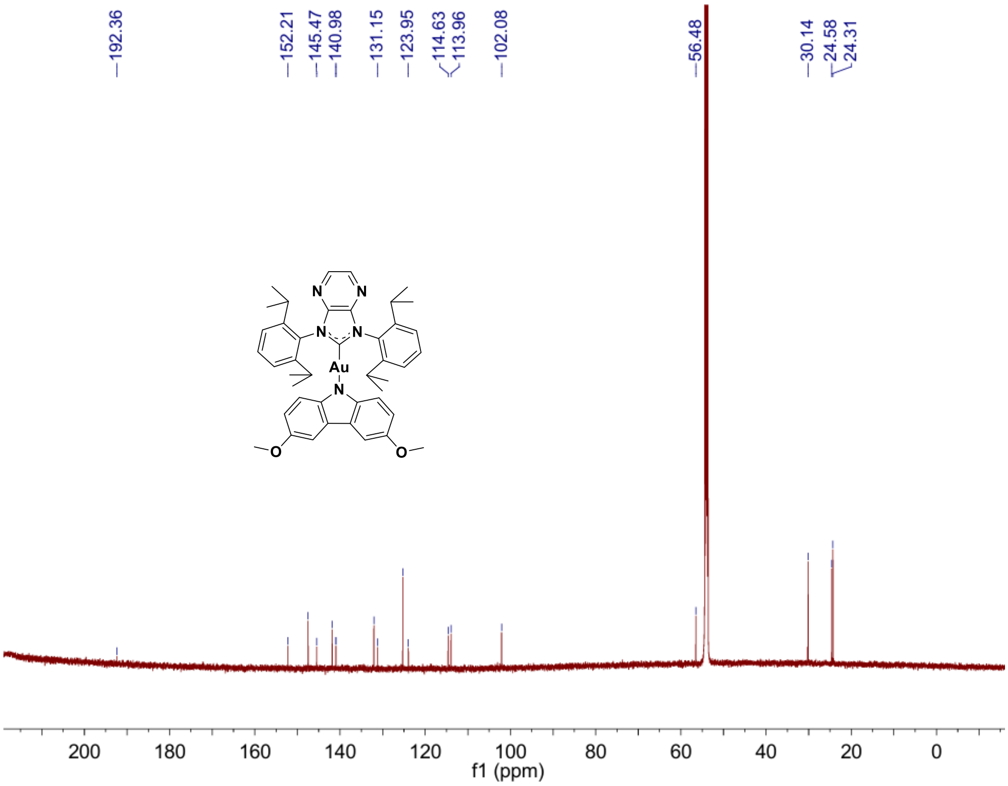

Figure S12. ^{13}C NMR spectrum of **Au-1$^{\mathbf{2OMe}}$** (151 MHz, CD_2Cl_2).

S1.4. Crystal structure determination

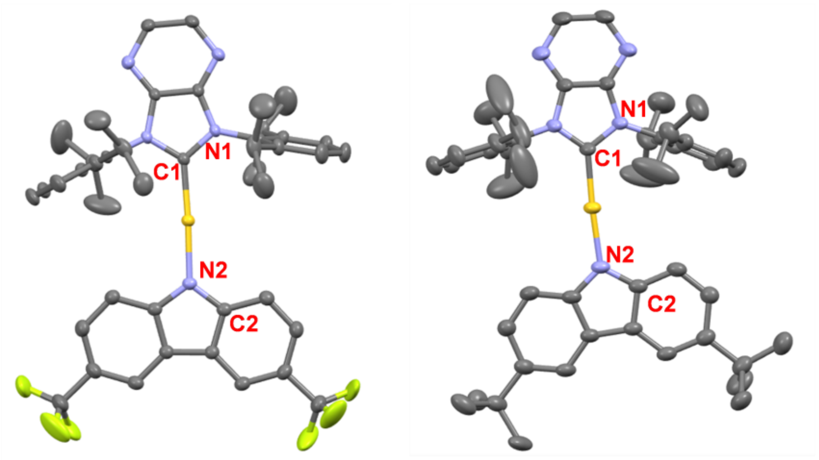

Figure S13. Crystal structures of **Au-1^{2CF3}** and **Au-1^{2tBu}**. Hydrogen atoms were omitted for clarity.

Table S1. Key crystal structure parameters of CMA emitters selected in literature and obtained in this work.

Compound	$d_{M\text{-}C1}$	$d_{M\text{-}N2}$	$d_{C1\text{-}N2}$	$\angle_{C1\text{-}M\text{-}N2}$	$\theta_{N1\text{-}C1\text{-}N2\text{-}C2}$	Ref
**Au-1^{2CF3}**	1.967	2.010	3.975	170.48	6.75	This work
**Au-1^{2tBu}**	1.961	1.991	3.949	176.38	6.02	
**Au-1**	1.973	2.021	3.992	176.5	6.42	[2]
**Au-1^{PYL}**	1.967	2.003	3.967	175.2	3.33	[2]
**Cu-1^{FLR}**	1.864	1.854	3.715	174.88	11.77	[3]

The units of bond length and bond angle are Å and ° respectively.

Table S2. Selected crystallographic data of **Au-1^{2CF3}** and **Au-1^{2tBu}**.

Complex	**Au-1^{2CF3}**	**Au-1^{2tBu}**
CCDC	2477707	2477706
Formula	$C_{43}H_{42}N_5AuF_6$	$C_{49}H_{60}N_5Au$
Temperature, K	100	100
Formula weight	939.78	915.98
Color	Colorless	Orange
Crystal system	Monoclinic	Triclinic
Space group	*P 21/n*	*-P 2yn*
a, Å	16.4109	14.8808
b, Å	12.8194	12.7996
c, Å	19.3177	24.5555
α, deg	90	90
β, deg	95.344	103.689
γ, deg	90	90
Cell volume, Å^3	4046.4(7)	4544.2(6)
Z	4	4
Density, calculated, g cm^{-3}	1.543	1.339
μ, mm^{-1}	5.082	4.386
F(000)	1872.0	1872.0
R_1	0.0186	0.0257
wR_2	0.0478	0.0695
GoF	1.040	1.042

S1.5. Electrochemical properties

Electrochemical studies were conducted with cyclic voltammetry (CV) and differential pulse voltammetry (DPV) in MeCN with 0.1 M ($^{n}Bu_4N)PF_6$ as electrolyte, saturated calomel electrode (SCE) as reference electrode, glassy carbon as working electrode, Pt wire as counter electrode. Fc^+/Fc was used as internal reference, and the oxidation potential (E_{ox}) was measured in the range of 0.400 – 0.454 V vs SCE.

Table S3. Summary of electrochemical results of **Au-1^{2CF3}**, **Au-1^{2tBu}**, and **Au-1^{2OMe}**.

Complex	E_{ox} / V vs Fc	E_{red} / V vs Fc	HOMO[a)] / eV	LUMO[a)] / eV
**Au-1^{2CF3}**	0.75	−1.92	−5.65	−2.56
**Au-1^{2tBu}**	0.22	−1.97	−5.05	−2.51
**Au-1^{2OMe}**	0.04	−1.96	−4.84	−2.52

[a)] where HOMO represents highest occupied molecular orbital and LUMO represents lowest unoccupied molecular orbital. E_{red} represents reduction potential. HOMO = 1.15×E_{ox} + 4.79, LUMO = 1.18 × E_{red} + 4.83, E_{ox} and E_{red} were estimated with DPV.

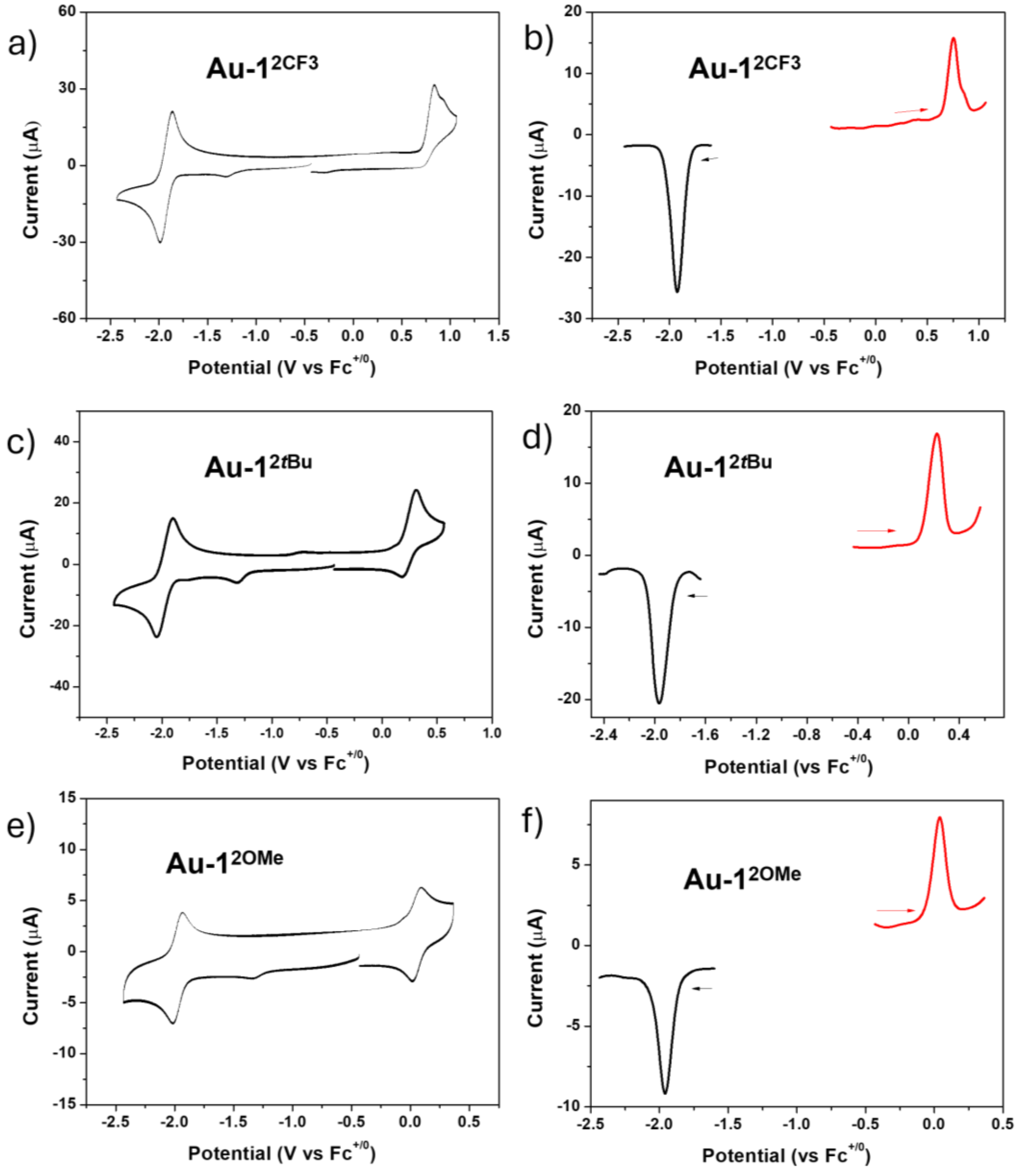

Figure S14. Cyclic voltammograms (a, c, and e) and differential pulse voltammograms (b, d and f) of **Au-1^{2CF3}**, **Au-1^{2tBu}**, and **Au-1^{2OMe}**.

S1.6. Photophysical measurements

Table S4. Photophysical properties of CMA emitters **Au-1** and **Ag-1**.

	Media	Absorption λ_{max} / nm (ε / 10^4 $M^{-1}cm^{-1}$)[a)]	Emission λ_{max} / nm (τ / μs)	Φ_{em}	k_{TADF}[b)] (10^5) / s^{-1}
**Au-1**	2wt% mCP[c)]	N/A	573 (0.34)	0.82	24.1
**Ag-1**	Toluene	309 (1.7), 356 (0.2), 373 (0.3), 378 (0.2)	676 (< 0.1)	0.06	–
	2wt% mCP	N/A	565 (0.23)	0.72	31.3
**Au-1^{2CF3}**	Toluene	296 (2.7), 314 (1.3), 342 (0.4), 360 (0.5), 409 (1.1)	509 (0.41)	0.83	20.0
	2wt% mCP	N/A	479 (0.33)	0.71	22.0
**Au-1^{2tBu}**	Toluene	310 (2.8), 318 (1.8), 339 (0.6), 370 (0.3), 503 (1.0)	650 (<0.1)	0.13	N/A
	2wt% mCP	N/A	599 (0.40)	0.81	20.0
**Au-1^{2OMe}**	Toluene	311 (2.2), 318 (2.3), 349 (0.4), 374 (0.3), 395 (0.3), 519 (0.6)	727	0.007	N/A
	2wt% mCP	N/A	602 (0.32)	0.35	11.0

[a)] Measured with [CMA] = 1 × 10^{-4} M. [b)] k_{TADF} represent the TADF radiative decay rate. $k_{TADF} = \Phi_{em}/\tau_{em}$.

[c)] 1,3-bis(N-carbazolyl)benzene (mCP)

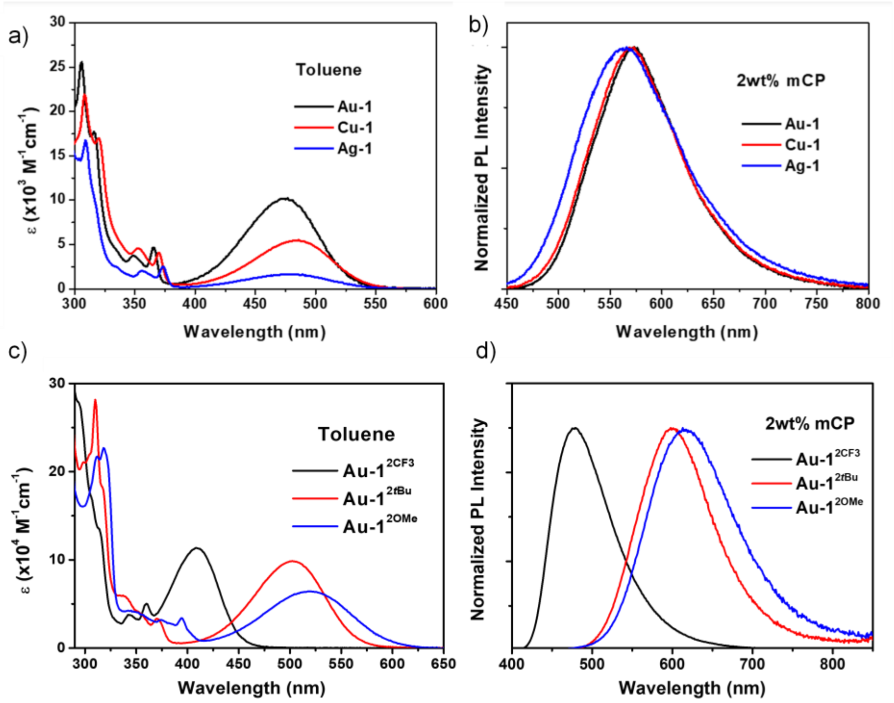

Figure S15. Absorption spectra of (a) **Au-1**, **Ag-1**, and **Cu-1**, and (c) **Au-1^{2CF3}**, **Au-1^{2tBu}**, and **Au-1^{OMe}**; and emission spectra of (b) **Au-1**, **Ag-1**, and **Cu-1**, and (d) **Au-1^{2CF3}**, **Au-1^{2tBu}**, and **Au-1^{OMe}**.

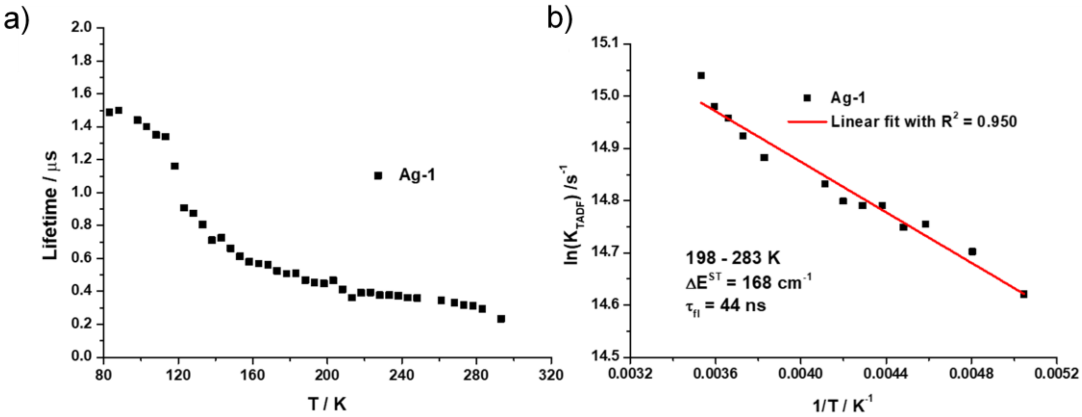

Figure S16. (a) Variable temperature lifetime measurement and (b) the corresponding Arrhenius plots of **Ag-1** doped in 2wt% mCP thin film.

S1.7. Electroluminescent properties

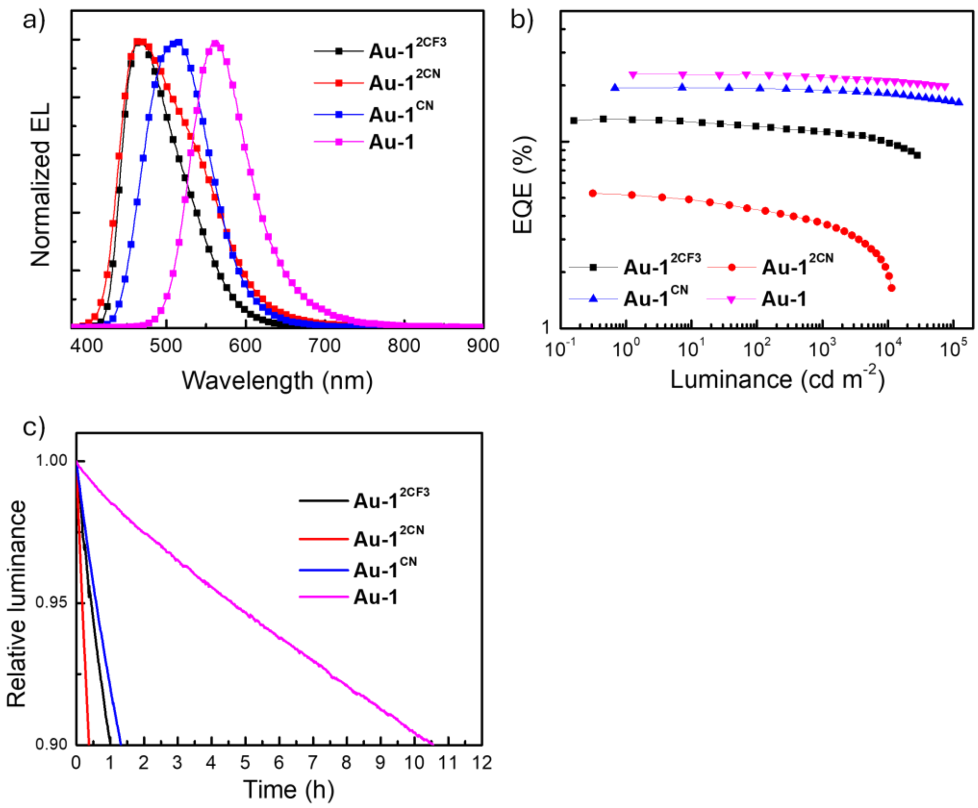

Figure S17. Characteristics of CMA(Au) OLEDs: a) EL spectra, b) EQE-luminance, and c) device operational lifetime.

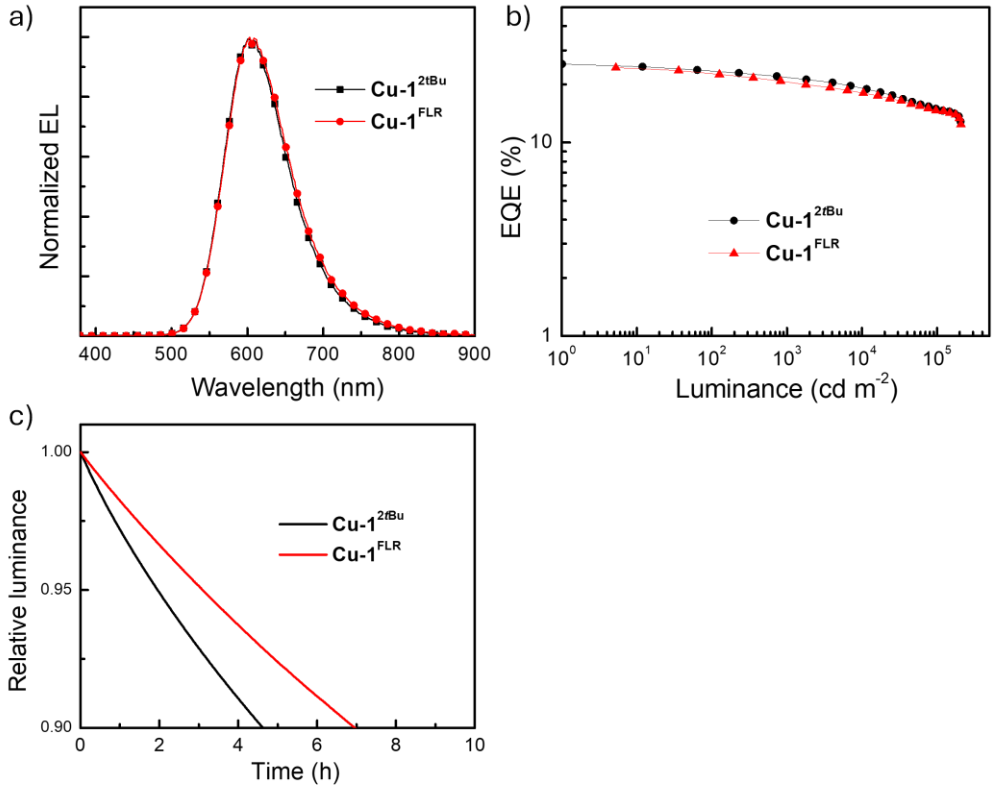

Figure S18. Characteristics of CMA(Cu) OLEDs: a) EL spectra, b) EQE-luminance, and c) device operational lifetime.

Table S5. Key device parameters of CMA OLEDs.

Emitter (2 wt%)	L_{max} [cd m^{-2}]	CE [a] [cd A^{-1}]		PE[b] [lm W^{-1}]		EQE [%]		CIE coordinates [(x, y)]	λ_{max}[c] [nm]	LT_{90} (h)	
		Max	at 1000 cd m^{-2}	Max	at 1000 cd m^{-2}	Max	at 1000 cd m^{-2}			at L_0[c]	at 1000 cd m^{-2} [d]
**Au-1^{2CF3}**	28400	25.1	21.2	19.4	6.52	13.2	11.3	0.17, 0.26	469	1.01	1.01
**Au-1^{2CN}**	11500	12.5	7.87	11.2	2.71	5.29	3.68	0.21, 0.31	467	0.37	0.37
**Au-1^{CN}**	121000	55.9	53.2	58.6	28.7	19.4	18.9	0.22, 0.48	511	1.31	131
**Au-1**	74000	73.4	72.3	76.9	34.9	23.0	22.1	0.44, 0.54	563	10.5	1709
**Cu-1^{2tBu}**	20700	46.5	41.4	58.4	28.2	25.5	21.8	0.57, 0.42	603	4.61	755
**Cu-1^{FLR}**	20900	41.4	37.4	52.1	25.4	24.4	20.6	0.58, 0.42	603	6.96	1125

[a] Current efficiency; [b] Power efficacy; [c] Initial luminance (cd m^{-2}): 1000 for **Au-1^{2CF3}** and **Au-1^{2CN}**, 15000 for **Au-1^{CN}**, 20000 for **Au-1**, **Cu-1^{2tBu}**, **Cu-1^{FLR}**; [d] Estimated by an acceleration coefficient of 1.7 using equation $LT_{1000}=LT_0(L_0/1000)^n$.

S2. Computational Section

S2.1. Computational details

S2.1.1. General computational details

S2.1.1.1. DFT calculations

The density functional theory (DFT) and time-dependent density functional theory (TDDFT) calculations including geometry optimization, adiabatic excited state energy calculations, determination of oscillator strength *f*, and estimation of electronic configurations were performed using the Gaussian 16 program package.[12] DFT calculations were carried out for ground state S_0 and TDDFT calculations were performed for the first singlet excited state S_1 and the first triplet excited state T_1. The hybrid functional PBE0[13-14] with D3 version of dispersion Becke-Johnson damping (D3BJ) [15-16] was applied for calculations. For all atoms, with the exception of gold, silver, and copper, the 6-31G* basis set[17-18] was applied. Gold, silver, and copper were described by Stuttgart/Dresden (SDD) pseudopotential and basis sets (ECPMWB60 for Au, ECPMWB28 for Ag, and ECPMDF10 for Cu). Non-relativistic calculations were performed using non-relativistic SDD pseudopotentials and basis sets for gold, silver, and copper (ECPMHF60 for Au, ECPMHF28 for Ag, and ECPMHF10 for Cu). Solvent effects were taken into account by means of the polarizable continuum model (PCM) with toluene as solvent.[19] The natural electronic configuration, which illustrates the degree of (n+1)s/(n+1)p-nd hybridization of gold, silver, and copper, was calculated using the NBO 6.0 program[20-21] implemented in the Gaussian 16 program package.

The triplet radiative decay rate constant k_{T1}, matrix element of the spin-orbit coupling (SOCME) of the excited state S_1 ($V_{SOC,S1}$) and T_1 ($V_{SOC,T1}$), and the analysis based on extended transition-state method with natural orbitals for chemical valence (ETS-NOCV)[22] were performed using the Amsterdam Density Functional (ADF) 2019

package.[23-25] Zeroth Order Regular Approximation (ZORA)[26-28] was applied to describe scalar relativistic effects. The all electron Slater-type orbital (STO) basis set of TZP (triple-ζ polarization function) was applied for all atoms. Solvent effects were considered by means of the Conductor like Screening Model (COSMO) with toluene as the solvent.[29-30] For ETS-NOCV analysis, the molecular structures and orbitals for ETS-NOCV analysis were observed using the ADF view. Theory and details for ETS-NOCV analysis in this work are described in Section S2.3.7.

Natural adaptive orbital (NAdO) analysis,[31] charge decomposition analysis (CDA)[32-33], determination of the overlap integral between the highest occupied molecular orbital (HOMO) and the lowest unoccupied molecular orbital (LUMO), molecular orbital composition analysis based on the Stout-Politzer parition,[34] and calculation of the Mayer bond order were performed using the Multiwfn 3.8(dev).[35] The electronic wavefunctions used for these analyses were generated by the Gaussian16 program package[12] at the computational level same as the geometry optimization. Isosurface maps of NAdOs and HOMO/LUMO were rendered by VMD 1.9.3 software.[36] The topological steric maps and buried volumes with spherical shape were obtained using the SambVca 2 Web tool.[37] The buried volume were calculated using the optimized coplanar structure in the ground state. Theory and details for NAdO and CDA analysis in this work are described in Section S2.3.6 and S2.3.8.

S2.1.1.2. CCSD calculations

The similarity transformed equation of motion domain-based local pair natural orbital coupled cluster singles and doubles (STEOM-DLPNO-CCSD) calculation[38-45] was performed with the def2-TZVP basis set[46] using the ORCA 5.0.3 program package.[47] Density fitting approximation RI-JK[48] was employed with the corresponding auxiliary basis set to speed up calculations. Tight thresholds were employed for the self-consistent field (SCF). Five roots were requested for each calculation with the $T_{\mathrm{CutPNOSingles}}$ keyword set to 5.0×10^{-12} and the active space selection keywords

“Othresh” and “Vthresh” set to 1.0×10^{-3}.

S2.1.1.3 DFT/MRCI calculations

The combined density functional theory and multireference configuration interaction (DFT/MRCI) calculations were performed using the parallelized version of DFT/MRCI program[49] based on geometries optimized at the TDDFT level. DFT/MRCI is semi-empirical multi-reference configuration interaction method that is based on Kohn-Sham orbitals and orbital energies derived from a closed-shell BH-LYP functional [50-51] anchor determinant representing the electronic ground state.[52-53] Such molecular orbitals were generated using the def2-TZVP basis set was used for all atoms from a converged DFT run with BH-LYP functional performed with ORCA 5.0.3 program package. The redesigned Hamiltonian for multi-chromophore systems[49] was employed and the configuration selection threshold was tightly set to 0.8 E_h. All orbitals with energies of below -3.0 E_h or above +3.0 E_h were frozen. At all geometries, 10 singlet and 10 triplet roots were calculated.

S2.1.2. Functional validation

In the literature, the PBE0[13-14] functional has been extensively used for calculating excited state properties of carbene-metal-amide (CMA) type emitters.[4, 54-57] The optimized geometries in the S_1 excited state were used to calculate the emission energies of complexes in toluene solution. The calculated results were compared with the corresponding experimental results. The mean error between the theoretical and experimental emission energy was ~0.19 eV (Table S23), which is typically reasonable for the TDDFT approach,[58] validating the level of theory.

S2.1.3. Plotting of potential energy surfaces (PES) for excited states

S2.1.3.1. TDDFT method

The S_1 and T_1 excited state potential energy surfaces (PES) of emitters **M-1** to **M-3**

were plotted by a geometric scan on torsional angles ($\theta_{C3\text{-}C1\text{-}N2\text{-}C2}$ for **M-2** and $\theta_{N1\text{-}C1\text{-}N2\text{-}C2}$ for **M-1 and M-3**) with an increment of 15° in the range of 0° to 180° using the TDDFT method. For each geometry, excited state geometry optimization was performed with the torsional angle being the only fixed parameter and the adiabatic energy was used to plot the excited state PES. The adiabatic energies of optimized rotamers are shown in Table S18. In the PES plots, the lowest energy, which is the energy of the semi-coplanar T_1 structure (θ = 0°), was set as 0 eV for comparison.

The fully relaxed S_1 excited state geometry optimization for **Au-2** resulted in only a perpendicular structure (Figure S19). To keep consistency, we used the relaxed **Au-2** geometry in the S_1 state with torsional angle $\theta_{C2\text{-}C1\text{-}N2\text{-}C3}$ of 30°, which represents a local minimum on the PES of the S_1 state, as the semi-coplanar structure of **Au-2** in the S_1 state for subsequent calculations.

S2.1.3.2. STEOM-DLPNO-CCSD method

The S_1 and T_1 excited state PES of emitters **M-1'** were plotted by a geometric scan on torsional angles (θ) with an increment of 30° in the range of 0° to 180°. For each geometry, excited state geometry optimization was performed with the torsional angle being the only fixed parameter using the TDDFT method and the adiabatic energy was calculated by the STEOM-DLPNO-CCSD method. The adiabatic energies are listed in Table S19 and were used to plot the excited state PES. In the PES plots, the lowest energy was set as 0 eV for comparison.

S2.1.3.3. DFT/MRCI method

The S_1 and T_1 excited state PES of emitters **M-1'** were plotted by a geometric scan on torsional angles (θ) with an increment of 15° in the range of 0° to 90°. For each geometry, excited state geometry optimization was performed with the torsional angle being the only fixed parameter using the TDDFT method and the adiabatic energy was calculated by the DFT/MRCI method. The adiabatic energies are listed in Table S20

and were used to plot the excited state PES. In the PES plots, the lowest energy was set as 0 eV for comparison.

S2.1.4. NAdO analysis

The delocalization index (DI) can be used to quantitatively estimate the degree of electron pair sharing between two distinct regions, serving as an indicator of covalent bond strength. The Mayer bond order is related to the DI calculated across the atomic spaces. The bond order density (BOD),[59] which is a real space function, reveals the impact of electron distribution through three-dimensional (3D) space on the DI. The natural adaptive orbital (NAdO),[31] derived from the computation of BOD, offers insight into the nature and degree of contribution by various orbitals to the DI by the NAdO graphical plots. Eigenvalues of NAdOs indicate the magnitude of the contributions. The sum of eigenvalues of all NAdOs is equivalent to the DI in the specified region.

In this work, we calculated the bonding interactions of the metal-carbon (M-C) and metal-nitrogen (M-N) bonds in CMA emitters using NAdO analysis. Calculations were performed on the structures of the CMA emitters that were optimized in the S_1 (semi-coplanar and orthogonal) and T_1 (semi-coplanar) excited states. The atomic space was partitioned using Becke's method[60] with Pyykkö's covalent atomic radius[61] for NAdO analysis.

S2.1.5. ETS-NOCV analysis

The ETS-NOCV[22] scheme reveals the bonding nature between fragments in a complex. The total bonding energy between the interacting fragments (ΔE_{Tol}) is divided into five components as shown in equation (1):

$$\Delta E_{\mathrm{Tot}} = \Delta E_{\mathrm{Prep}} + \Delta E_{\mathrm{Orb}} + \Delta E_{\mathrm{Elst}} + \Delta E_{\mathrm{Pauli}} + \Delta E_{\mathrm{Disp}} \quad (1)$$

Here, ΔE_{Prep} represents the preparation energy required to distort the isolated fragments from their equilibrium geometries to the structure of complex formation. ΔE_{Orb} accounts for the orbital interaction energy due to mix of fragment orbitals. This includes the effect of both charge-transfer (interaction between occupied orbital of one fragment and unoccupied orbital of the other fragment) and polarization (interaction between occupied and unoccupied orbitals within a single fragment). ΔE_{Elst} corresponds to the classical electrostatic interaction between the unperturbed charge distributions of the prepared fragments (geometric deformed fragments) when brought together at their final positions. $\Delta E_{\mathrm{Pauli}}$ represents the Pauli repulsion interaction arising from overlap between occupied orbitals of the fragments. ΔE_{Disp} corresponds to the London dispersion interaction energy which describes the long-ranged dispersive interaction between fragments.[62]

The deformation density, which is the change in electron density upon orbital interaction between fragments A and B, is given by:

$$\Delta\rho(r) = \rho_{\mathrm{AB}}(r) - [\rho_{\mathrm{A}}(r) + \rho_{\mathrm{B}}(r)] \tag{2}$$

The deformation density matrix is expressed as the difference between the molecular and fragmental matrices:

$$\Delta P = P_{\mathrm{AB}} - P_{\mathrm{A}} - P_{\mathrm{B}} \tag{3}$$

The change in electron density that gives rise to ΔE_{Orb} can be expressed as:

$$\Delta\rho(r) = \sum_{\alpha}^{M}\sum_{\beta}^{M} \Delta P_{\alpha\beta}\chi_{\alpha}(r)\chi_{\beta}(r) \tag{4}$$

where α and β represent occupied and unoccupied orbitals on the two fragments, χ refers to the basis sets, and *M* represents the number of basis functions.

According to extended transition-state (ETS) scheme,[62] ΔE_{Orb} is expressed as:

$$\Delta E_{\text{Orb}} = \sum_{\alpha}^{M}\sum_{\beta}^{M} \Delta P_{\alpha\beta} F_{\alpha\beta}^{TS} \tag{5}$$

where $F_{\alpha\beta}^{TS}$ is the extended transition-state Kohn-Sham Fock matrix element constructed using the average of molecule and the sum of the fragments.

Following Nalewajski−Mrozek valence theory,[63-66] the eigenvectors derived from diagonalizing the deformation density matrix (ΔP) are defined as natural orbitals for chemical valence (NOCV).[67-68] The NOCV pairs (Ψ_{-k} and Ψ_k) allow for decomposition of the deformation density ($\Delta\rho$(r)) into the NOCV contributions ($\Delta\rho_k$) as:

$$\Delta\rho(r) = \sum_{k=1}^{M/2} \nu_k[-\psi_{-k}^2(r) + \psi_k^2] = \sum_{k=1}^{M/2} \Delta\rho_k(r) \tag{6}$$

where ν_k corresponds to eigenvalues of basis functions. Deformation density plots of NOCV channels ($\Delta\rho_k$) allow visualization of symmetry and direction of charge flow, where charge depletion and charge accumulation are reflected by negative and positive values of $\Delta\rho_k$, respectively.

In the combined scheme of ETS-NOCV,[67] ΔE_{Orb} is expressed in terms of eigenvalues of NOCV paris (ν_k) as:

$$\Delta E_{\text{Orb}} = \sum_{k} \Delta E_{Orb}^{k} = \sum_{k=1}^{M/2} \nu_k\left[-F_{-k,-k}^{TS} + F_{k,k}^{TS}\right] \tag{7}$$

where $F_{-k,-k}^{TS}$ and $F_{k,k}^{TS}$ are diagonal Kohn-Sham Fock matrix elements established regarding to the NOCVs concerning the transition state (TS). The ETS-NOCV scheme allows decomposition of inter-fragment orbital interaction into limited number of contributions from major NOCV pairs. For every NOCV pair representing a charge delocalization channel ($\Delta\rho_k$), the energy contribution (ΔE_{Orb}^{k}) to the bond energy can be estimated and the electron flow channel can be visualized. In an ETS-NOCV plot, red

color and blue color of $\Delta\rho_k$ show charge depletion and charge accumulation, respectively, due to bond formation.

Here, CMA emitters were separated into two fragments, namely the metal ion (Au^+, Ag^+, and Cu^+) and the ligands (carbene and carbazole), for ETS-NOCV analysis. The calculations were performed on the structures of the CMA emitters that were optimized in the S_1 (semi-coplanar and orthogonal) and T_1 (semi-coplanar) excited states.

S2.1.6. CDA analysis

The charge decomposition analysis (CDA) proposed by Dapprich and Frenking[32] is widely used in the literature to provide a quantitative description of electron donation and back-donation within the Dewar-Chatt-Duncanson (DCD) bonding model. In CDA, molecular orbitals (MOs) are expressed as linear combination of fragment orbitals (FOs) of donors and acceptors. For the i-th MO of the complex, charge donation (d_i) represents the amount of electron donation from donor to acceptor, back-donation (b_i) represents the amount of electron back-donation from acceptor to donor, and charge polarization (r_i) represents the interaction between occupied orbitals of both fragments. The equations used for in these calculations are:

$$d_i = \sum_{m\epsilon A}^{\mathrm{occ}} \sum_{n\epsilon B}^{\mathrm{vir}} \eta_i C_{m,i} C_{n,i} S_{m,n} \quad (8)$$

$$b_i = \sum_{m\epsilon A}^{\mathrm{occ}} \sum_{n\epsilon B}^{\mathrm{vir}} \eta_i C_{m,i} C_{n,i} S_{m,n} \quad (9)$$

$$r_i = \sum_{m\epsilon A}^{\mathrm{occ}} \sum_{n\epsilon B}^{\mathrm{vir}} \eta_i C_{m,i} C_{n,i} S_{m,n} \quad (10)$$

where η donates the occupation number of the MO(i), $C_{m,i}$ and $C_{n,i}$ are the coefficients of donor FO(m) and acceptor FO(n) in the complex MO(i), and $S_{m,n}$ is the overlap

integral between donor FO(m) and acceptor FO(n). The terms "occ" and "vir" represent occupied orbitals and virtual (i.e. unoccupied) orbitals, respectively.

In this work, using the CDA method, we analyzed the number of electrons donated from the carbene or carbazole ligands to the metal ion (Au^+, Ag^+, and Cu^+) for the formation of C/N→M σ-bonds, as well as the number of electrons back-donated from the metal ions to the ligands for the formation of M→C/N π-interactions. The metal ion and the corresponding carbene or carbazole ligands are defined as separate fragments for this analysis. Calculations were performed on structures of CMA emitters optimized in the S_1 (semi-coplanar and orthogonal) and T_1 (semi-coplanar) excited states.

S2.1.7. Calculation of rate constants k_{S1}, k_{T1}, k_{ISC}, k_{rISC}, and k_{TADF}

The radiative rate constant (k_{S1}) for the S_1→S_0 transition was calculated using the Einstein spontaneous emission rate formula:[69]

$$k_{\mathrm{S1}} = \frac{1}{\tau} = \frac{f_{\mathrm{S1-S0}}\, {E_{\mathrm{S1,min-S0,min}}}^2}{1.5} \tag{11}$$

Here, τ represents the fluorescence lifetime, $f_{S1\text{-}S0}$ donates the S_1→S_0 transition oscillator strength, and $E_{S1,min\text{-}S0,min}$ is the adiabatic energy difference between the global minimum of the potential energy surfaces of S_1 and S_0.

The radiative rate constant for the T_1→S_0 transition (k_{T1}) was calculated using the Amsterdam Density Functional (ADF) 2019 package.[23-25]

The intersystem crossing (ISC) rate constant (k_{ISC}) and the reverse intersystem crossing (rISC) rate constant (k_{rISC}) were calculated using the simplified expression of semiclassical Marcus theory and the classical Fermi-golden rule:[70-71]

$$k_{\mathrm{ISC}} = \frac{2\pi}{\hbar}\left|\langle\psi_{\mathrm{S1}}|\widehat{\mathcal{H}}_{SO}|\psi_{\mathrm{T1}}\rangle\right|^2 \sqrt{\frac{1}{4\pi k_{\mathrm{B}} T \lambda_{\mathrm{T}}}} \exp\left[-\frac{(\Delta E_{\mathrm{T1-S1}} + \lambda_{\mathrm{T}})^2}{4 k_{\mathrm{B}} T \lambda_{\mathrm{T}}}\right] \tag{12}$$

$$k_{\mathrm{rISC}} = \frac{2\pi}{\hbar} \left| \langle \psi_{\mathrm{T1}} | \hat{\mathcal{H}}_{SO} | \psi_{\mathrm{S1}} \rangle \right|^2 \sqrt{\frac{1}{4\pi k_{\mathrm{B}} T \lambda_{\mathrm{S}}}} \exp\left[-\frac{(\Delta E_{\mathrm{S1-T1}} + \lambda_{\mathrm{S}})^2}{4 k_{\mathrm{B}} T \lambda_{\mathrm{S}}} \right] \quad (13)$$

In these equations, $\hbar$ is the reduced Planck constant, k_{B} is the Boltzmann constant, T is the temperature set at 298 K, and $\Delta E_{\mathrm{S1\text{-}T1}}$ is the adiabatic energy difference between the S_1 and T_1 states. The SOC matrix elements calculated using restrictively relaxed S_1 and T_1 geometries, are represented by $|\langle \psi_{\mathrm{S1}} | \hat{\mathcal{H}}_{SO} | \psi_{\mathrm{T1}} \rangle|$ and $|\langle \psi_{\mathrm{T1}} | \hat{\mathcal{H}}_{SO} | \psi_{\mathrm{S1}} \rangle|$, respectively. The Marcus reorganization energies λ_{T} and λ_{S} are formulated as:

$$\lambda_{\mathrm{T}} = E_{\mathrm{T1/S1}} - E_{\mathrm{T1}} \quad (14)$$

$$\lambda_{\mathrm{S}} = E_{\mathrm{S1/T1}} - E_{\mathrm{S1}} \quad (15)$$

Here, $E_{\mathrm{T1/S1}}$ is the T_1 energy in the S_1 restrictively relaxed geometry, $E_{\mathrm{S1/T1}}$ is the S_1 energy in the T_1 restrictively relaxed geometry. E_{T1} and E_{S1} are the adiabatic T_1 or S_1 energies, respectively (Figure S29).

The overall thermally activated delayed fluorescence (TADF) emission rate constant (k_{TADF}) of emitters was estimated by assuming a fast thermal equilibrium between all thermally accessible rotamers in restrictively relaxed geometries on the potential energy surfaces ($\theta = 0°, 15°, 30°, ..., 180°$):

$$k_{\mathrm{TADF}} = \frac{\sum_{\theta} \left[k_{\mathrm{TADF},\theta} \exp\left(-\frac{\Delta E_{\mathrm{S1},\theta-\mathrm{S1,min}}}{k_B T} \right) \right]}{\sum_{\theta} \left[\exp\left(-\frac{\Delta E_{\mathrm{S1},\theta-\mathrm{S1,min}}}{k_{\mathrm{B}} T} \right) \right]} \quad (16)$$

Here, $\Delta E_{\mathrm{S1},\theta-\mathrm{S1,min}}$ represents the difference between the energy of the specific S_1 state in the twisted structure and the minimum energy of the S_1 state on the potential energy surface. $k_{\mathrm{TADF},\theta}$ donates the TADF emission rate constant of specific rotamers. The method used to calculate $k_{\mathrm{TADF},\theta}$ is presented in the main text.

The calculated values of $E_{\mathrm{S1},\theta}$, $E_{\mathrm{T1},\theta}$, $\Delta E_{\mathrm{S1\text{-}T1},\theta}$, $f_{\mathrm{S1}\rightarrow\mathrm{S0},\theta}$, $\lambda_{\mathrm{T},\theta}$, $\lambda_{\mathrm{S},\theta}$, $|\langle \psi_{\mathrm{S1}} | \hat{\mathcal{H}}_{SO} | \psi_{\mathrm{T1}} \rangle|$,

$|\langle\psi_{\mathrm{T1}}|\hat{\mathcal{H}}_{SO}|\psi_{\mathrm{S1}}\rangle|$, k_{S1}, $k_{\mathrm{T1},\theta}$, $k_{\mathrm{ISC},\theta}$, $k_{\mathrm{rISC},\theta}$, and $k_{\mathrm{TADF},\theta}$, for the restrictively relaxed rotamers of emitters, as well as the overall k_{TADF} values for the emitters, are presented in Table S18.

S2.2. Additional results and discussion

S2.2.1. Excited state potential energy surfaces (PES) and dihedral angle rotation flexibility of emitters

To study the changes in the torsional structures of the emitter in the excited state, we plotted the adiabatic potential energy surfaces (PES) of the S_1 and T_1 excited states using the energies of the relaxed structures at fixed torsional angles θ (0°–180°, step = 15°) calculated by TDDFT (see Figure S24a for **M-1** and Figure S27 for **M-2** and **M-3**; see Section S2.1.3 for details of the PES calculations). The calculated excited-state PES show differences between emitters with different metal atoms, and the flatness of the PES follows the order of Ag(I) emitter > Au(I) emitter > Cu(I) emitter. For example, the $\Delta E_{S1\text{-}T1}$ values of all rotamers are in the range of 0.002-0.096 eV for **Ag-1**, 0.003-0.141 eV for **Au-1**, and 0.004-0.164 eV for **Cu-1**, respectively. Similar trends are observed for **M-2** and **M-3**.

Taking into account the inherent errors of the TDDFT method, the excited-state PES of the simplified structures **M-1'** (M = Au, Ag, Cu) is analyzed at the def2-TZVP basis set level[46] within the framework of STEOM-DLPNO-CCSD[38-45] (Figure S24b and Table S19; see Section S2.1.1.2 and S2.1.3.2 for details of the STEOM-DLPNO-CCSD and PES calculations). In the simplified structures, the diisopropylphenyl (Dipp) group is replaced by the methyl group. Since the Dipp group does not participate in the frontier molecular orbitals of the S_1 and T_1 excited states, this structural simplification is not expected to significantly affect the accuracy of excited-state PES calculations. Considering the potential error introduced by combining STEOM-DLPNO-CCSD with polarizable continuum model (PCM) for solvent effects,[72] the calculations were performed in vacuo. As a result, the excited-state PES barriers are systematically higher compared to the TDDFT results (see Figure S26 for the solvent effect on PES energy barriers). Nonetheless, the PES trends of simplified emitters **M-1'** with different metal

atoms closely follow those obtained from TDDFT calculations of **M-1**.

The high-level DFT/MRCI method developed by Marian group surpasses the linear response TDDFT and introduces higher-order excited states through multireference configuration interaction (MRCI) expansion, which is expected to provide high precision excited-state analysis for d^{10} CMA complexes.[49, 73] We further applied DFT/MRCI to calculate the excited-state PES of the simplified **M-1'** structure (Figure S25 and Table S20). The resulting PES trend of different metal atoms is consistent with the results of TDDFT and STEOM-DLPNO-CCSD (Figure S24). (see Section S2.1.1.3 and S2.1.3.3 for details of the DFT/MRCI and PES calculations).

This means that the excited-state dihedral angle rotations of carbene and carbazole ligands are most flexible for Ag(I) emitters and most hindered for Cu(I) emitters. The trend of PES flatness in the excited state is consistent with the trend of k_{TADF} in these CMA emitters: Ag > Au > Cu. A flatter PES indicates more flexibility in dihedral angle rotations, making it easier for the complex to transform into an orthogonal conformation and reduce $\Delta E_{\mathrm{S1\text{-}T1}}$. This is expected to increase k_{TADF} values of CMA emitters.

As shown in Figure S24a-b, the excited state PES of **Cu-1** and **Cu-1'** are asymmetric. This can be explained by the following reasons: 1) In the excited state, structural distortion causes bending of the ligand-metal-ligand (L-M-L) backbone. As shown in Table S7, the $\angle_{\mathrm{C1\text{-}Cu\text{-}N1}}$ angle of the rotamer differs from 180° (167°-179°), which disrupts molecular symmetry and leads to an asymmetric PES. 2) During rotamer optimization, the dihedral angle $\theta_{\mathrm{N1\text{-}C1\text{-}N2\text{-}C2}}$ is fixed, while all other atoms relax. Since the carbene and carbazole ligands are not perfectly planar, this relaxation disrupts mirror symmetry. Distortions within the ligands cause the rotation of $\theta_{\mathrm{N1\text{-}C1\text{-}N2\text{-}C2}}$ to have different effects on the other internal dihedral angles. Therefore, the sum of the selected dihedral angle and another dihedral angle of ligands may not be equal to 180° (Table S7). 3) Relaxed dihedral angle scan explores a multi-dimensional PES with multiple

symmetrically equivalent local minima at each dihedral angle. As the optimized rotamer moves along this potential energy surface, it may fall into different branches of these minima on both sides of 90º, resulting in an asymmetric PES even if the underlying surface is symmetric.

S2.2.2. The metal relativistic effects on metal hybridization, metal-ligand interactions, and the rotational flexibility of emitters

As demonstrated in our previous report, metal (n+1)s-nd and (n+1)p-nd hybridization is influenced by relativistic effects.[74-75] To further study the impact of relativistic effects on excited state rotational dynamics of emitters, we performed single point calculations on **Au-1**, **Ag-1**, and **Cu-1** using basis sets excluding the relativistic effect (please see Section 2.1.1 "General computational details" for detailed calculation methods). Our results show that when the relativistic effects are not considered, the degree of metal (n+1)p-nd hybridization is reduced for **Au-1**, while remaining almost unchanged for **Ag-1** and **Cu-1** (Table S8). Without consideration of the relativistic effects, the calculated PES of **Au-1** is remarkably flattened, while the calculated PES of **Ag-1** and **Cu-1** are moderately changed (Figure S28). Without consideration of relativistic effects, the PES flatness follows the trend: **Au-1** > **Ag-1** > **Cu-1**. That is, the significant relativistic effect of Au leads to a higher degree of (n+1)p-nd hybridization and restricts the dihedral rotation of Au(I) emitters. This restriction enables Ag(I) emitters to exceed Au(I) emitters in terms of ligand rotational flexibility. Therefore, we observed a trend in rotational flexibility: Ag(I) emitters > Au(I) emitters > Cu(I) emitters.

S2.2.3. Charge decomposition analysis

We applied charge decomposition analysis (CDA)[32-33, 35] in Multiwfn 3.8(dev)[35] to quantify the charge (electron) donation and back donation interaction between the metal atom and the ligand in the emitter. The CDA results show that the electron flow is

related to the formation of C/N→M σ-bonds and M→C/N π-interactions. Specifically, in the S_1 state-optimized semi-coplanar **Au-1**, the carbene and carbazole ligands contribute 0.367 e^- and 0.220 e^- to the Au atom, respectively, to form Au-C and Au-N σ-bonds. On the other hand, there are 0.064 e^- and 0.007 e^- flow from Au atoms to carbene and carbazole ligands, respectively, which is related to the Au-C and Au-N π-interactions (Table S16). The amount of electrons/charge transferred from Au atoms to the carbazole ligands (0.007 e^-) is smaller than that transferred to the carbene ligand (0.064 e^-). This shows that the Au-N π-interaction is weaker, which is beneficial to the flexibility of dihedral angle rotation. For different metal atoms, the order of the extent of π-electron back donation from metal to carbazole is **Ag-1** (0.001 e^-) < **Au-1** (0.007 e^-) < **Cu-1** (0.009 e^-) (Table S16). Similar results were observed for various optimized structures and excited states for all sets of emitters (Figure S42 and Table S16). The CDA analysis results support those of the NAdO and ETS-NOCV analyses, elucidating the excited-state dihedral angle rotational flexibility of the emitters (Ag(I) emitter > Au(I) emitter > Cu(I) emitter) by providing additional quantification of the electron density transfer.

It is noteworthy that the π-interactions in these d^{10} complexes originate from orbital mixing and electron sharing between the (n+1)p orbitals and nd orbitals of the metal atom and the ligand π/π* system. The occupancy of the metal p orbitals in the ground and excited states is due to the hybridization of the metal d orbitals and their mixing with the ligand π/π* orbitals (see Figure 2a of the Main Text for the orbital interaction diagram). This mixing effect will transfer electron density from the ligand π orbitals to the metal p orbitals, and vice versa (that is, electron density transfers from the metal p orbitals to the ligand π* orbitals). In other words, the p orbital mediates a bidirectional electron sharing effect, rather than a unidirectional back-donation effect. CDA results only show the net electron transfer and cannot reflect the extent of orbital mixing. ETS-NOCV and NAdO analyses can more directly and quantitatively assess metal-ligand

(M-L) π-interactions.

S2.2.4. Further discussions on metal-ligand π-interaction and dihedral angle rotation

Excited state potential energy surfaces calculated at different levels (i.e. TDDFT, STEOM-DLPNO-CCSD, and DFT/MRCI) consistently show the same trend in rotational barriers: Ag(I) emitters < Au(I) emitters < Cu emitters. This means that the excited state dihedral rotation is most flexible for Ag(I) emitters and most hindered for Cu(I) emitters. Although a new π-interaction forms as the ligand rotates, the rotation is not instantaneous and requires passing through geometries where the π-overlap is partially or fully disrupted. From a kinetic perspective, the molecule must still overcome this temporary loss of stabilization, which produces a rotational barrier on the PES. Thus, differences in the intrinsic strength of the M-L π-interaction directly influence how steeply the stabilization is lost along the rotation coordinate and therefore affect the height of the rotational barrier, even if the final conformations at 0° and 90° both regain some π-character. The PES therefore can meaningfully reflect how π-interaction strength modulates rotational difficulty.

S2.2.5. Reasons for the differences in the calculated radiative decay rate constants of symmetric rotational isomers

As shown in Table S18, the calculated rate constants ($k_{S1,\theta}$, $k_{T1,\theta}$, $k_{ISC,\theta}$, $k_{rISC,\theta}$, and $k_{TADF,\theta}$) differ in the symmetric rotational isomers with dihedral angles on either side of 90°. For example, there is a significant difference, exceeding one order of magnitude, between $k_{ISC,0^{\circ}}/k_{rISC,0^{\circ}}$ and $k_{ISC,180^{\circ}}/k_{rISC,180^{\circ}}$ for **Au-2**. This can be explained by the fact that although the $\theta = 0^{\circ}$ and 180° conformations appear chemically equivalent at first glance, there are non-negligible geometric differences between the optimized rotamers. During the rotamer optimization process, the dihedral angle $\theta_{C3\text{-}C1\text{-}N2\text{-}C3}$ is fixed, while all other internal coordinates are allowed to relax. In the relaxed structure, the carbene and

carbazole ligands are not perfectly planar, so rotation around $\theta_{C3\text{-}C1\text{-}N2\text{-}C2}$ alters the other internal dihedral angles in different ways. Therefore, the sum of the fixed dihedral angle and the dihedral angle of the other ligand may not equal 180º (Figure S22), leading to subtle structural differences between the 0º and 180º rotamers. Furthermore, the relaxed dihedral angle scan explores a multi-dimensional potential energy surface, where multiple symmetry-equivalent local minima exist at each dihedral angle. Therefore, the optimized rotamers at 0° and 180° may converge to different local minima, resulting in slightly different rotamer geometries. Since the ISC and *r*ISC rate constants are highly sensitive to small geometric changes, these deviations lead to observed variations in k_{ISC} and k_{rISC}. Notably, the differences between the corresponding $k_{TADF,0^{\circ}}$ and $k_{TADF,180^{\circ}}$ values remain within one order of magnitude, supporting the overall reliability of our calculations. Therefore, the observed rate constant differences stem from small but unavoidable geometric deviations introduced during constrained optimization process, rather than from chemically meaningful in-equivalences of symmetric rotational isomers.

S2.3. Calculation results

S2.3.1. Structural properties

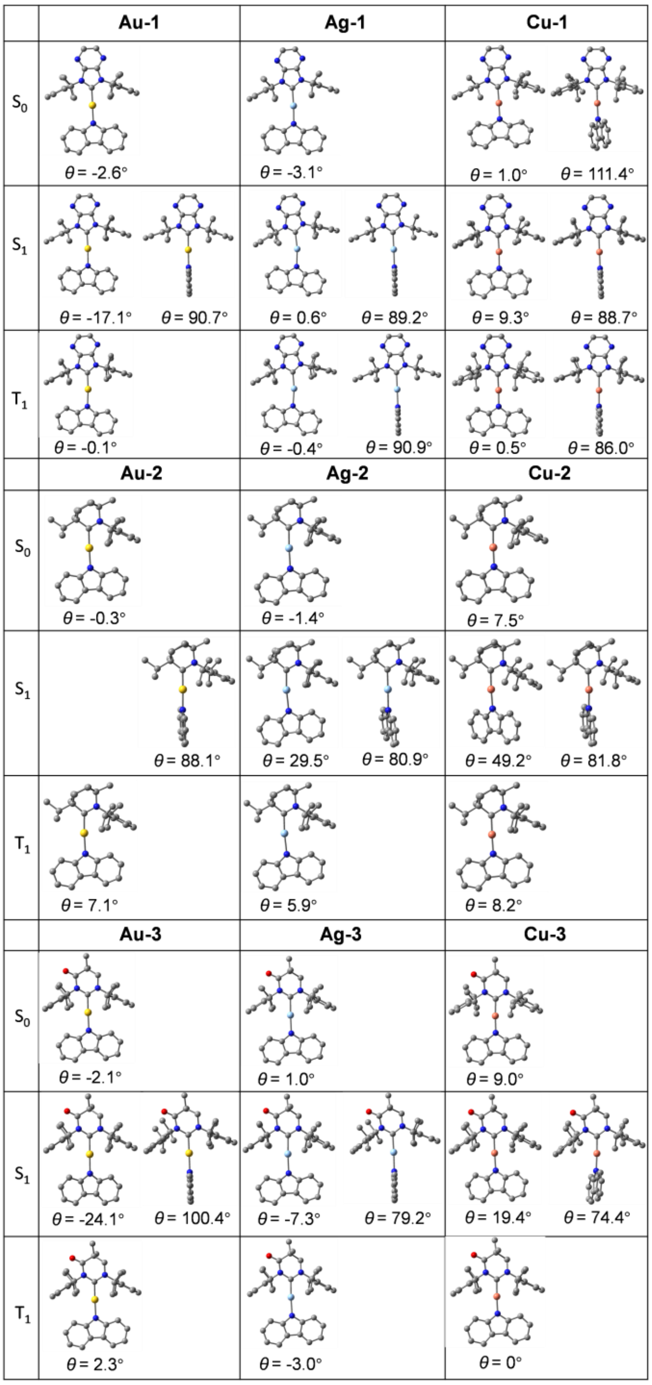

Figure S19. Optimized geometries of emitters **M-1**, **M-2** and **M-3** in the ground state S_0, and excited states S_1 and T_1. The torsional angle θ is labeled in each optimized geometry.

Table S6. Summary of the structural properties of X-ray crystal structures and DFT optimized structures of CMA emitters **M-1**, **M-2**, and **M-3** in the ground state (optimized semi-coplanar structures were used for comparison).

	**Au-1**	**Au-2**	**Ag-2**	**Cu-2**	**Au-3**	**Ag-3**	**Cu-3**
$d_{M\text{-}C1}$ (Å) Expt./Cal.	1.973/ 1.973	1.987/ 1.991	2.078/ 2.066	1.885/ 1.874	1.983/ 1.993	2.079/ 2.068	1.882/ 1.876
$d_{M\text{-}N2}$ (Å) Expt./Cal.	2.021/ 2.013	2.024/ 2.026	2.066/ 2.060	1.867/ 1.856	2.009 /2.102	2.067/ 2.051	1.853/ 1.848
$\angle_{C1\text{-}M\text{-}N2}$ (°) Expt./Cal.	176.5/ 178.0	176.2/ 177.6	175.6/ 176.8	176.0/ 177.9	180.0/ 177.2	180.0/ 177.2	178.2/ 177.3
θ (°) Expt./Cal.	2.2/ -2.6	19.5/ -0.3	22.0/ -1.4	19.6/ 7.5	-5.0/ -2.1	-0.5/ 1.0	-3.2/ 7.2

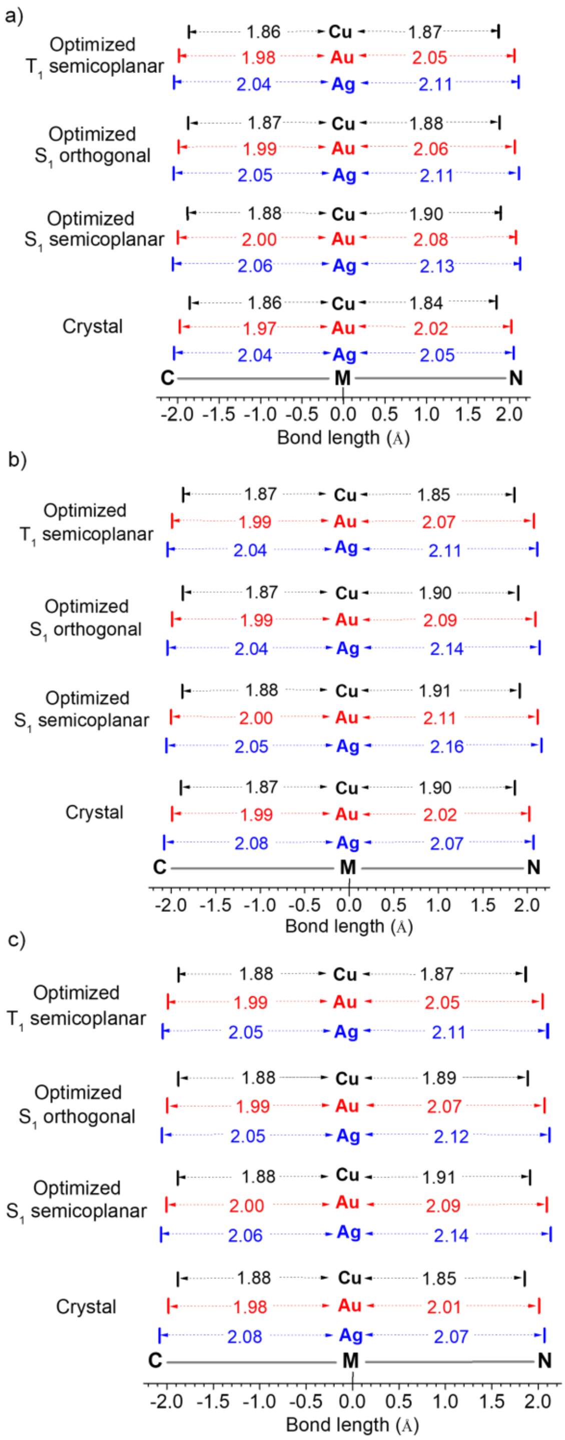

Figure S20. Schematic diagram showing M-C and M-N bond lengths in X-ray crystal structures, and optimized structures in the S_1 and T_1 excited states of emitters a) **M-1**, b) **M-2**, and c) **M-3**.

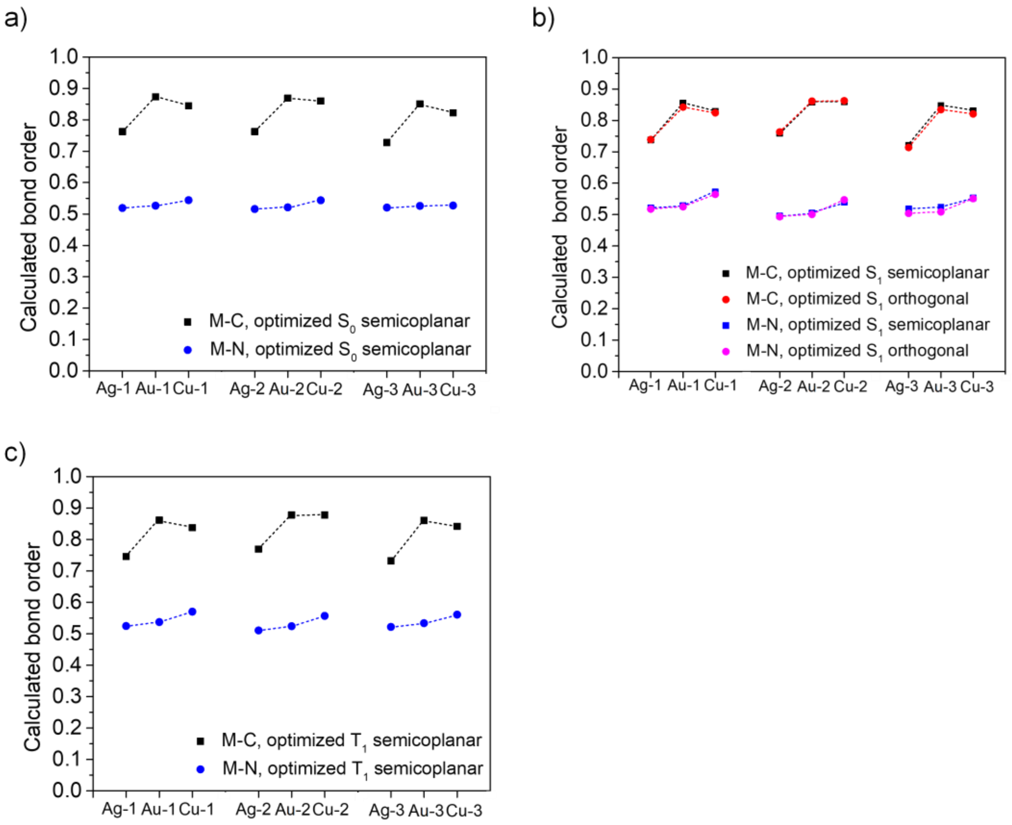

Figure S21. Calculated Mayer bond order of M-C and M-N bonds in emitters **M-1**, **M-2**, and **M-3** using a) optimized semi-coplanar structures in the ground state S_0, b) optimized semi-coplanar and orthogonal structures in the S_1 excited state, and c) optimized semi-coplanar structures in the T_1 excited state.

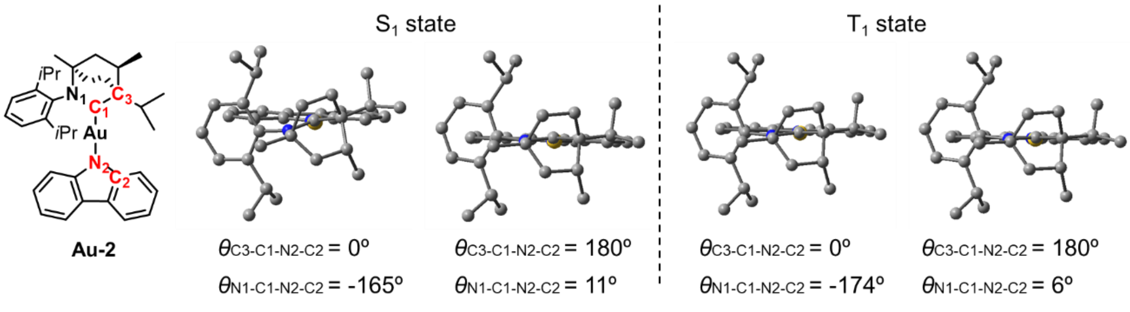

Figure S22. Top view of the optimized geometries of **Au-2** with $\theta_{C3\text{-}C1\text{-}N2\text{-}C3}$ fixed at 0º and 180º.

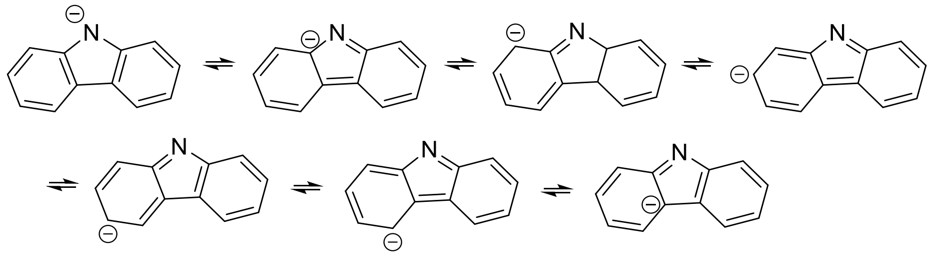

Figure S23. Resonance structures of the carbazole ligand.

S2.3.2. Excited state potential energy surfaces (PES)

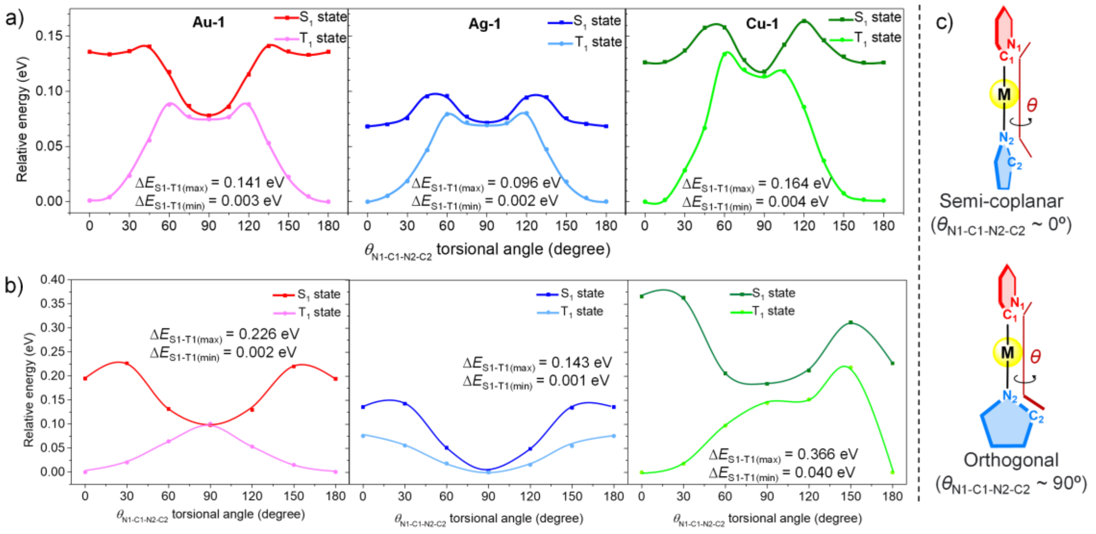

Figure S24. a) TDDFT calculated excited-state potential energy surfaces of **Au-1**, **Ag-1**, and **Cu-1**. b) STEOM-DLPNO-CCSD calculated potential energy surfaces of **Au-1'**, **Ag-1'**, and **Cu-1'**. c) Schematic diagrams showing the semi-coplanar and orthogonal geometries of CMA emitters.

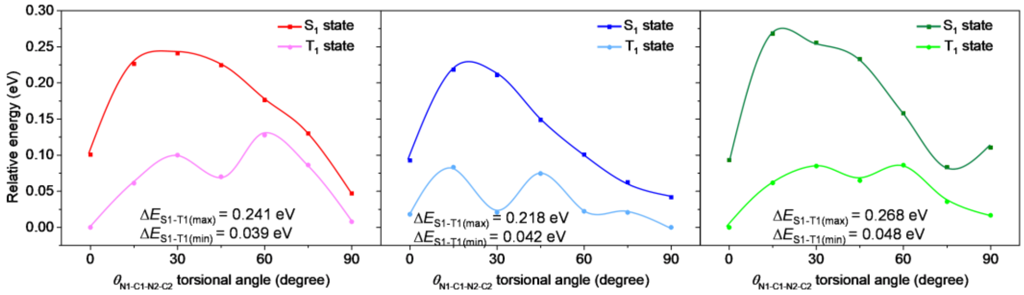

Figure S25. DFT/MRCI calculated potential energy surfaces of **Au-1'**, **Ag-1'**, and **Cu-1'**.

Table S7. $\angle_{C1\text{-}Cu\text{-}N1}$ angle and $\theta_{N1\text{-}C1\text{-}N2\text{-}C3}$ dihedral angle in relaxed geometries of **Cu-1** with fixed $\theta_{N1\text{-}C1\text{-}N2\text{-}C2}$ dihedral angle.

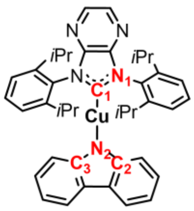

Cu-3

$\theta_{N1\text{-}C1\text{-}N2\text{-}C2}$ (º)	0	15	30	45	60	75	90	105	120	135	150	165	180
	$\angle_{C1\text{-}Cu\text{-}N1}$ angle (º)												
S_1	177	177	177	173	178	178	179	177	175	170	174	176	177
T_1	177	176	177	173	176	179	179	178	167	169	172	176	176
	$\theta_{N1\text{-}C1\text{-}N2\text{-}C3}$ dihedral angle (º)												
S_1	179	-168	-162	-151	-111	-99	-111	-86	-76	-25	-17	-11	3
T_1	177	-163	-162	-156	-127	-100	-92	-81	-28	-19	-12	-7	-3

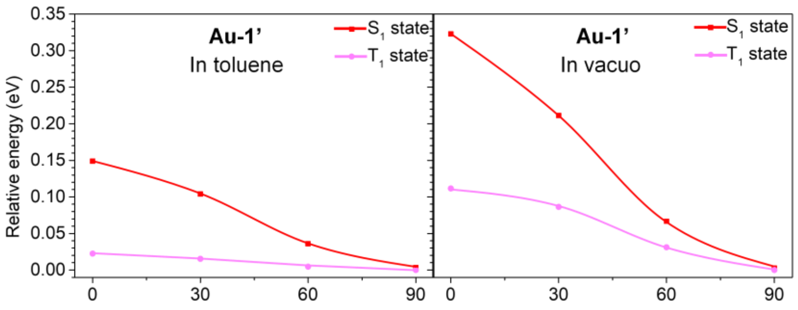

Figure S26. TDDFT calculated excited-state potential energy surfaces of **Au-1'** in toluene solution and in vacuo.

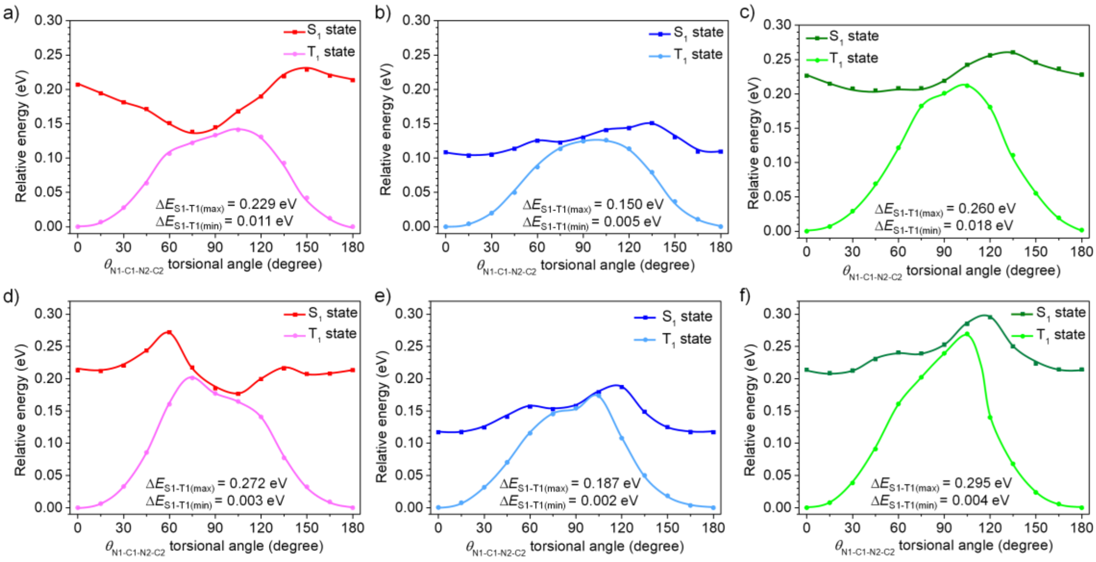

Figure S27. TDDFT calculated excited state potential energy surfaces of a) **Au-2**, b) **Ag-2**, c) **Cu-2**, d) **Au-3**, e) **Ag-3**, and f) **Cu-3**.

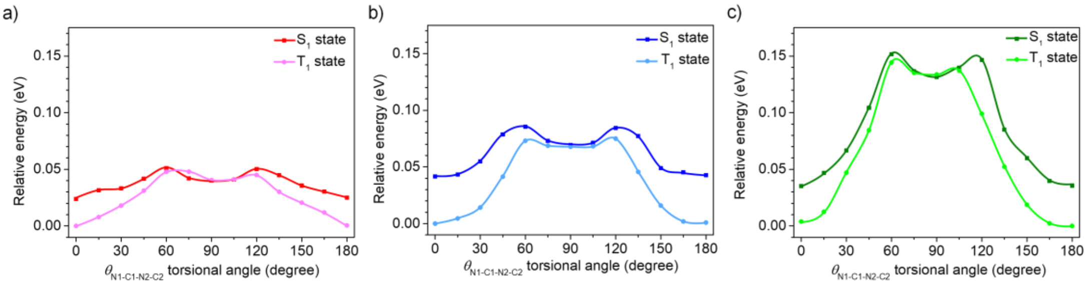

Figure S28. TDDFT calculated excited state potential energy surfaces of a) **Au-1**, b) **Ag-1**, and c) **Cu-1** without consideration of relativistic effects.

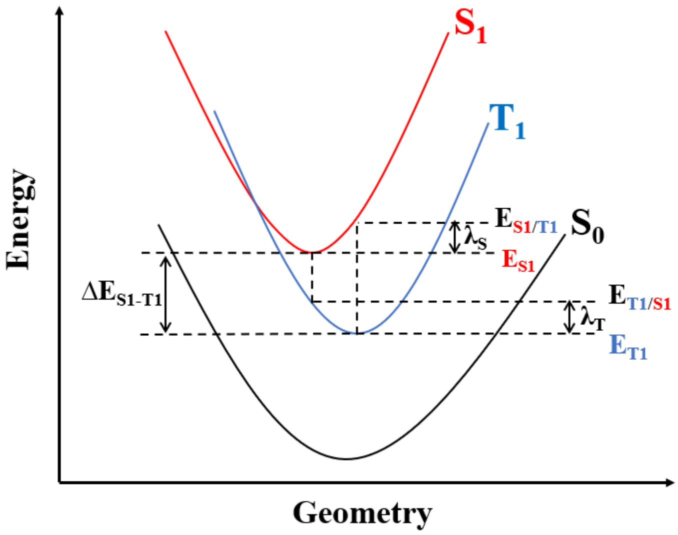

Figure S29. Schematic diagram of the potential energy curves of the S_0, S_1, and T_1 electronic states and reorganization energies λ_S and λ_T.

S2.3.3. Molecular orbital interaction diagrams

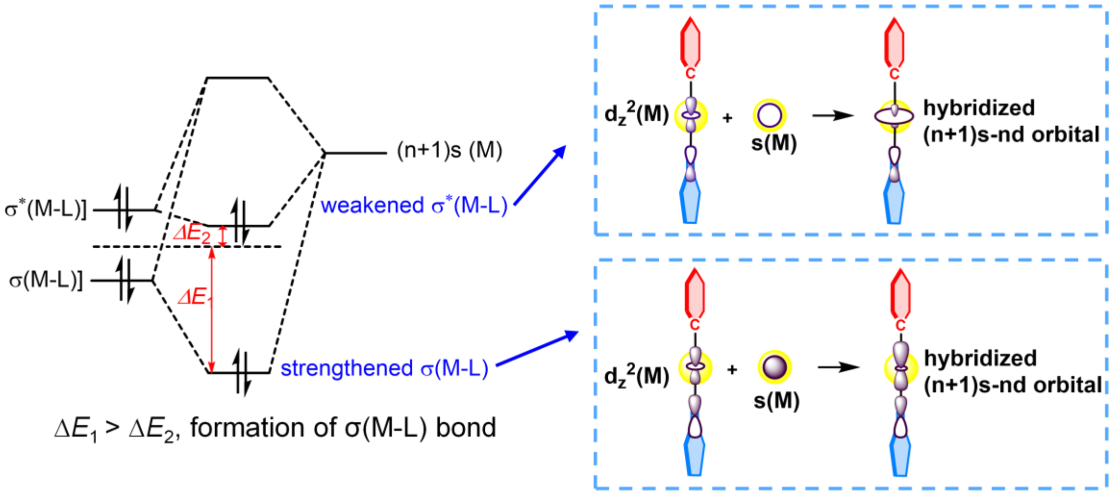

Figure S30. Interaction diagram of the σ(M-L) bond by including (n+1)s-nd orbital hybridization in d^{10} metal complexes (left), and schematic diagrams showing the metal (n+1)s-nd orbital hybridization for the formation of a strengthened σ(M-L) bond and a weakened σ^*(M-L) bond (right).

S2.3.4. Electronic configuration analysis

Table S8. Relativistic and nonrelativistic calculation for electronic configuration of metal in emitters **Au-1**, **Ag-1**, and **Cu-1**.

	Optimized structures	Calculated metal electronic configuration		
		**Au-1**	**Ag-1**	**Cu-1**
**Relativistic**	S_0 semi-coplanar	[Xe] $5d^{9.572}$ $6s^{0.956}$ $6p^{0.100}$	[Kr] $4d^{9.760}$ $5s^{0.639}$ $5p^{0.093}$	[Ar] $3d^{9.748}$ $4s^{0.595}$ $4p^{0.116}$
	S_1 semi-coplanar	[Xe] $5d^{9.591}$ $6s^{0.943}$ $5p^{0.091}$	[Kr] $4d^{9.779}$ $5s^{0.609}$ $5p^{0.079}$	[Ar] $3d^{9.759}$ $4s^{0.586}$ $4p^{0.103}$
	S_1 orthogonal	[Xe] $5d^{9.590}$ $6s^{0.948}$ $6p^{0.079}$	[Kr] $4d^{9.774}$ $5s^{0.620}$ $5p^{0.074}$	[Ar] $3d^{9.758}$ $4s^{0.592}$ $4p^{0.085}$
	T_1 semi-coplanar	[Xe] $5d^{9.583}$ $6s^{0.945}$ $6p^{0.087}$	[Kr] $4d^{9.772}$ $5s^{0.617}$ $5p^{0.080}$	[Ar] $3d^{9.759}$ $4s^{0.586}$ $4p^{0.103}$
**Non relativistic**	S_0 semi-coplanar	[Xe] $5d^{9.692}$ $6s^{0.709}$ $6p^{0.098}$	[Kr] $4d^{9.795}$ $5s^{0.558}$ $5p^{0.093}$	[Ar] $3d^{9.765}$ $4s^{0.565}$ $4p^{0.116}$
	S_1 semi-coplanar	[Xe] $5d^{9.716}$ $6s^{0.679}$ $6p^{0.088}$	[Kr] $4d^{9.813}$ $5s^{0.527}$ $5p^{0.080}$	[Ar] $3d^{9.776}$ $4s^{0.554}$ $4p^{0.103}$
	S_1 orthogonal	[Xe] $5d^{9.712}$ $6s^{0.790}$ $6p^{0.077}$	[Kr] $4d^{9.808}$ $5s^{0.539}$ $5p^{0.074}$	[Ar] $3d^{9.775}$ $4s^{0.561}$ $4p^{0.085}$
	T_1 semi-coplanar	[Xe] $5d^{9.705}$ $6s^{0.690}$ $6p^{0.084}$	[Kr] $4d^{9.806}$ $5s^{0.536}$ $5p^{0.080}$	[Ar] $3d^{9.776}$ $4s^{0.554}$ $4p^{0.103}$

Table S9. Calculated metal electronic configurations in CMA emitters **Au-1**, **Au-1^{CN}**, **Au-1^{2CN}**, **Au-1^{CF3}**, **Au-1^{2CF3}**, **Au-1^{2tBu}**, **Au-1^{OMe}**, **Au-1^{2OMe}**, and **Cu-1^{FLR}**.

Optimized structures	Calculated metal electronic configuration		
	**Au-1**	**Au-1^{CN}**	**Au-1^{2CN}**
S_0 semi-coplanar	[Xe]$5d^{9.5722}6s^{0.9564}6p^{0.0999}$	[Xe]$5d^{9.5795}6s^{0.9508}6p^{0.0987}$	[Xe]$5d^{9.5854}6s^{0.9471}6p^{0.0977}$
S_1 semi-coplanar	[Xe]$5d^{9.5912}6s^{0.9426}5p^{0.0909}$	[Xe]$5d^{9.5940}6s^{0.9367}5p^{0.0909}$	[Xe]$5d^{9.5981}6s^{0.9331}5p^{0.0871}$
S_1 orthogonal	[Xe]$5d^{9.5900}6s^{0.9477}6p^{0.0790}$	[Xe]$5d^{9.5938}6s^{0.9426}6p^{0.0772}$	[Xe]$5d^{9.5971}6s^{0.9389}6p^{0.0768}$
	**Au-1^{CF3}**	**Au-1^{2CF3}**	**Au-1^{2tBu}**
S_0 semi-coplanar	[Xe]$5d^{9.5768}6s^{0.9525}6p^{0.0991}$	[Xe]$5d^{9.5807}6s^{0.9505}6p^{0.0986}$	[Xe]$5d^{9.5703}6s^{0.9576}6p^{0.1001}$
S_1 semi-coplanar	[Xe]$5d^{9.5927}6s^{0.9402}5p^{0.0884}$	[Xe]$5d^{9.5942}6s^{0.9373}p^{0.0880}$	[Xe]$5d^{9.5901}6s^{0.9437}5p^{0.0917}$
S_1 orthogonal	[Xe]$5d^{9.5923}6s^{0.9448}6p^{0.0781}$	[Xe]$5d^{9.5941}6s^{0.9428}6p^{0.0777}$	[Xe]$5d^{9.5887}6s^{0.9494}6p^{0.080}$
	**Au-1^{OMe}**	**Au-1^{2OMe}**	**Cu-1^{FLR}**
S_0 semi-coplanar	[Xe]$5d^{9.5707}6s^{0.9569}6p^{0.0999}$	[Xe]$5d^{9.5693}6s^{0.9578}6p^{0.0999}$	[Ar]$3d^{9.7486}4s^{0.5958}4p^{0.1180}$
S_1 semi-coplanar	[Xe]$5d^{9.5912}6s^{0.9417}5p^{0.0915}$	[Xe]$5d^{9.5911}6s^{0.9439}5p^{0.0921}$	[Ar]$3d^{9.7581}4s^{0.5873}4p^{0.1071}$
S_1 orthogonal	[Xe]$5d^{9.5893}6s^{0.9483}6p^{0.0784}$	[Xe]$5d^{9.5890}6s^{0.9493}6p^{0.0819}$	[Ar] $3d^{9.7616}4s^{0.5793}4p^{0.1082}$

S2.3.5. Canonical molecular orbital analysis

Table S10. Calculated composition of metal (n+1)p orbitals in HOMO of emitters **M-1**, **M-2**, and **M-3**.

Optimized structures	Calculated metal (n+1)p orbital component in HOMO		
	**Au-1**	**Ag-1**	**Cu-1**
S_1 semi-coplanar	0.61%	0.49%	0.69%
S_1 orthogonal	0.27%	0.26%	0.43%
T_1 semi-coplanar	0.73%	0.52%	0.86%
	**Au-2**	**Ag-2**	**Cu-2**
S_1 semi-coplanar	0.36%	0.34%	0.44%
S_1 orthogonal	0.32%	0.25%	0.41%
T_1 semi-coplanar	0.73%	0.50%	0.90%
	**Au-3**	**Ag-3**	**Cu-3**
S_1 semi-coplanar	0.63%	0.58%	0.74%
S_1 orthogonal	0.17%	0.18%	0.31%
T_1 semi-coplanar	0.93%	0.66%	1.08%

Table S11. Calculated composition of metal (n+1)p orbital in HOMO of emitters **Au-1**, **Au-1^{CN}**, **Au-1^{2CN}**, **Au-1^{CF3}**, **Au-1^{2CF3}**, **Au-1^{2tBu}**, **Au-1^{OMe}**, and **Au-1^{2OMe}**.

Optimized structures	Calculated metal (n+1)p orbital component in HOMO		
	**Au-1**	**Au-1^{CN}**	**Au-1^{2CN}**
S_1 semi-coplanar	0.61%	0.52%	0.39%
S_1 orthogonal	0.27%	0.24%	0.16%
T_1 semi-coplanar	0.73%	0.58%	0.42%
	**Au-1^{CF3}**	**Au-1^{2CF3}**	**Au-1^{2tBu}**
S_1 semi-coplanar	0.56%	0.51%	0.67%
S_1 orthogonal	0.27%	0.23%	0.29%
T_1 semi-coplanar	0.65%	0.57%	0.78%
	**Au-1^{OMe}**	**Au-1^{2OMe}**	
S_1 semi-coplanar	0.66%	0.65%	
S_1 orthogonal	0.37%	0.31%	
T_1 semi-coplanar	0.82%	0.83%	

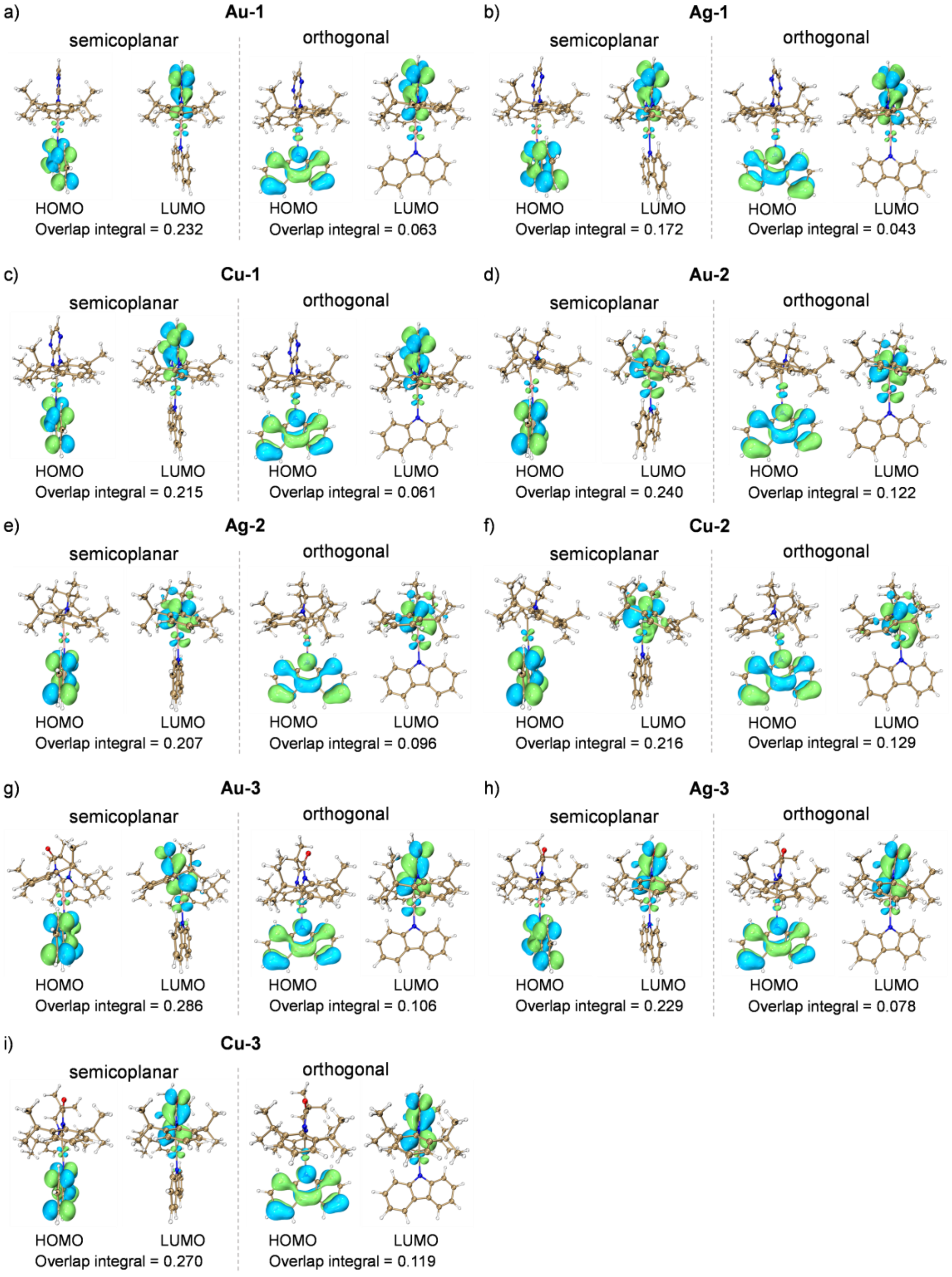

Figure S31. Frontier orbitals and HOMO-LUMO overlap integrals for emitters **M-1**, **M-2**, and **M-3** in optimized semi-coplanar and orthogonal geometries in the S_1 excited state.

S2.3.6. NAdO analysis

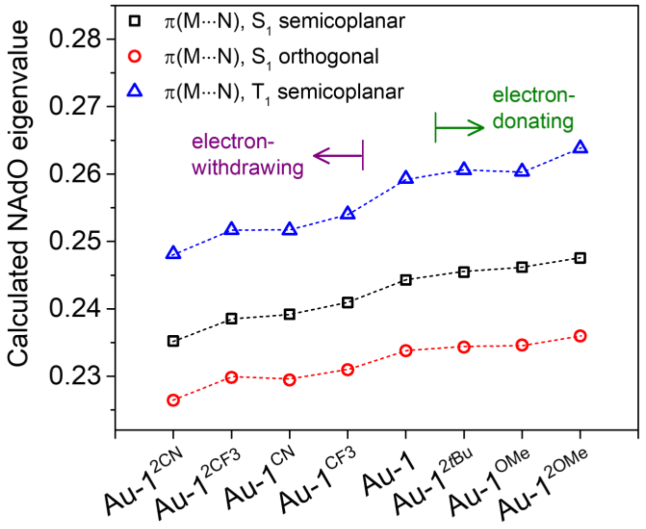

Figure S32. Sum of the NAdOs representing the π(M···N) interaction for **Au-1** and for **Au-1** with electron-withdrawing and electron-donating substituents on the carbazole ligand calculated using optimized semi-coplanar and orthogonal geometries in the S_1 excited state, and optimized semi-coplanar geometries in the T_1 excited state.

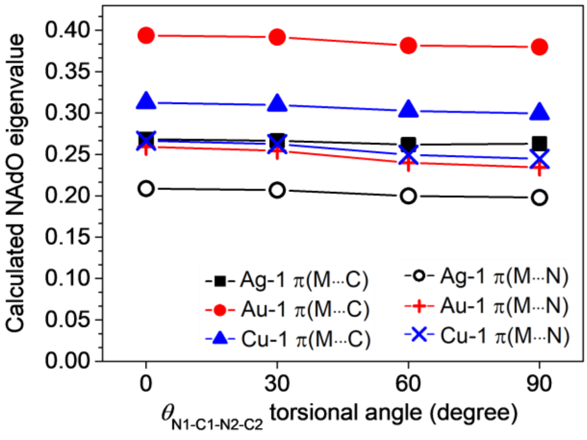

Figure S33. Sum of eigenvalues of NAdOs representing the π(M···C) interaction or the π(M···N) interaction for **M-1** in twist geometires with various dihedral angles in the T_1 excited state.

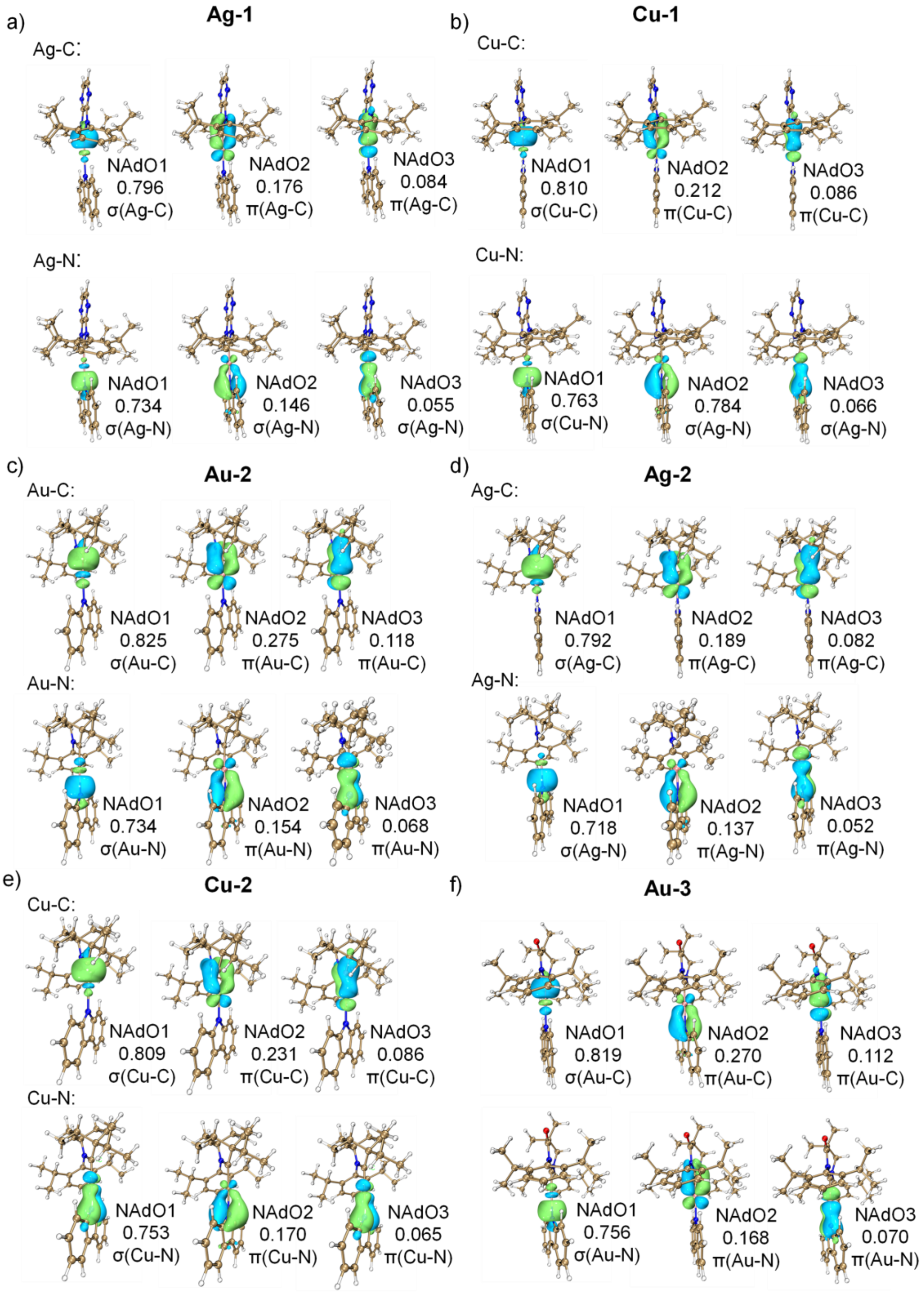

a)
Ag-1
Ag-C:
NAdO1
0.796
σ(Ag-C)
NAdO2
0.176
π(Ag-C)
NAdO3
0.084
π(Ag-C)
Ag-N:
NAdO1
0.734
σ(Ag-N)
NAdO2
0.146
σ(Ag-N)
NAdO3
0.055
σ(Ag-N)
b)
Cu-1
Cu-C:
NAdO1
0.810
σ(Cu-C)
NAdO2
0.212
π(Cu-C)
NAdO3
0.086
π(Cu-C)
Cu-N:
NAdO1
0.763
σ(Cu-N)
NAdO2
0.784
σ(Ag-N)
NAdO3
0.066
σ(Ag-N)
c)
Au-2
Au-C:
NAdO1
0.825
σ(Au-C)
NAdO2
0.275
π(Au-C)
NAdO3
0.118
π(Au-C)
Au-N:
NAdO1
0.734
σ(Au-N)
NAdO2
0.154
π(Au-N)
NAdO3
0.068
π(Au-N)
d)
Ag-2
Ag-C:
NAdO1
0.792
σ(Ag-C)
NAdO2
0.189
π(Ag-C)
NAdO3
0.082
π(Ag-C)
Ag-N:
NAdO1
0.718
σ(Ag-N)
NAdO2
0.137
π(Ag-N)
NAdO3
0.052
π(Ag-N)
e)
Cu-2
Cu-C:
NAdO1
0.809
σ(Cu-C)
NAdO2
0.231
π(Cu-C)
NAdO3
0.086
π(Cu-C)
Cu-N:
NAdO1
0.753
σ(Cu-N)
NAdO2
0.170
π(Cu-N)
NAdO3
0.065
π(Cu-N)
f)
Au-3
NAdO1
0.819
σ(Au-C)
NAdO2
0.270
π(Au-C)
NAdO3
0.112
π(Au-C)
NAdO1
0.756
σ(Au-N)
NAdO2
0.168
π(Au-N)
NAdO3
0.070
π(Au-N)

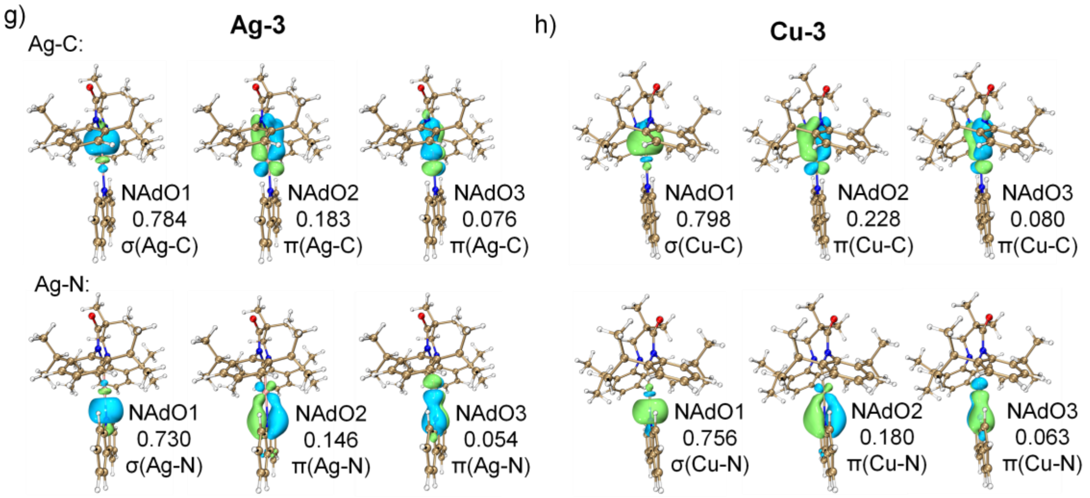

Figure S34. Three major NAdOs of the M-C and M-N bonds for a) **Ag-1**, b) **Cu-1**, c) **Au-2**, d) **Ag-2**, and e) **Cu-2**, f) **Au-3**, g) **Ag-3**, and h) **Cu-3** in optimized semi-coplanar geometries in the S_1 excited state.

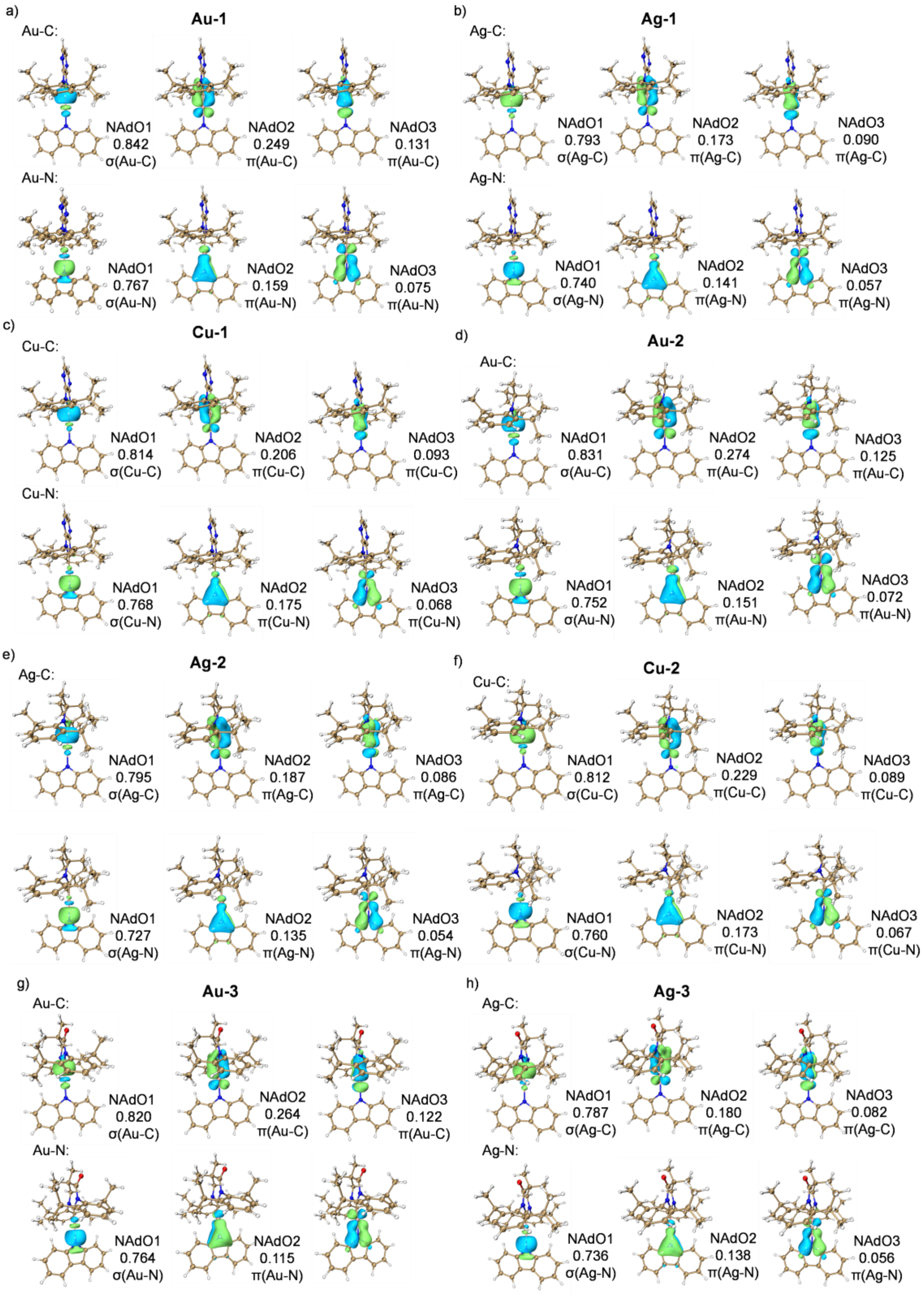
a)
Au-1
Au-C:
NAdO1
0.842
σ(Au-C)
NAdO2
0.249
π(Au-C)
NAdO3
0.131
π(Au-C)
Au-N:
NAdO1
0.767
σ(Au-N)
NAdO2
0.159
π(Au-N)
NAdO3
0.075
π(Au-N)
b)
Ag-1
Ag-C:
NAdO1
0.793
σ(Ag-C)
NAdO2
0.173
π(Ag-C)
NAdO3
0.090
π(Ag-C)
Ag-N:
NAdO1
0.740
σ(Ag-N)
NAdO2
0.141
π(Ag-N)
NAdO3
0.057
π(Ag-N)
c)
Cu-1
Cu-C:
NAdO1
0.814
σ(Cu-C)
NAdO2
0.206
π(Cu-C)
NAdO3
0.093
π(Cu-C)
Cu-N:
NAdO1
0.768
σ(Cu-N)
NAdO2
0.175
π(Cu-N)
NAdO3
0.068
π(Cu-N)
d)
Au-2
Au-C:
NAdO1
0.831
σ(Au-C)
NAdO2
0.274
π(Au-C)
NAdO3
0.125
π(Au-C)
NAdO1
0.752
σ(Au-N)
NAdO2
0.151
π(Au-N)
NAdO3
0.072
π(Au-N)
e)
Ag-2
Ag-C:
NAdO1
0.795
σ(Ag-C)
NAdO2
0.187
π(Ag-C)
NAdO3
0.086
π(Ag-C)
NAdO1
0.727
σ(Ag-N)
NAdO2
0.135
π(Ag-N)
NAdO3
0.054
π(Ag-N)
f)
Cu-2
Cu-C:
NAdO1
0.812
σ(Cu-C)
NAdO2
0.229
π(Cu-C)
NAdO3
0.089
π(Cu-C)
NAdO1
0.760
σ(Cu-N)
NAdO2
0.173
π(Cu-N)
NAdO3
0.067
π(Cu-N)
g)
Au-3
Au-C:
NAdO1
0.820
σ(Au-C)
NAdO2
0.264
π(Au-C)
NAdO3
0.122
π(Au-C)
Au-N:
NAdO1
0.764
σ(Au-N)
NAdO2
0.115
π(Au-N)
h)
Ag-3
Ag-C:
NAdO1
0.787
σ(Ag-C)
NAdO2
0.180
π(Ag-C)
NAdO3
0.082
π(Ag-C)
Ag-N:
NAdO1
0.736
σ(Ag-N)
NAdO2
0.138
π(Ag-N)
NAdO3
0.056
π(Ag-N)

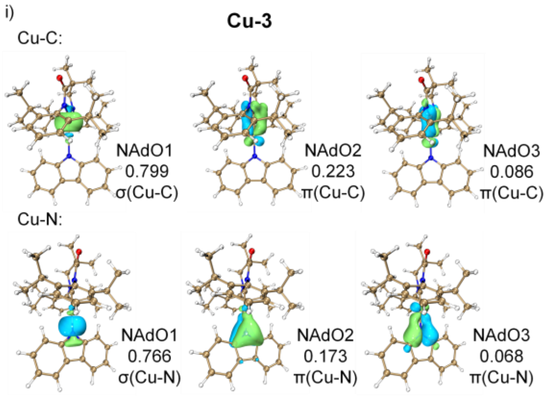

Figure S35. Three major NAdOs of the M-C and M-N bonds for a) **Au-1**, b) **Ag-1**, and c) **Cu-1**, d) **Au-2**, e) **Ag-2**, f) **Cu-2**, g) **Au-3**, h) **Ag-3**, and i) **Cu-3** in optimized orthogonal geometries in the S_1 excited state.

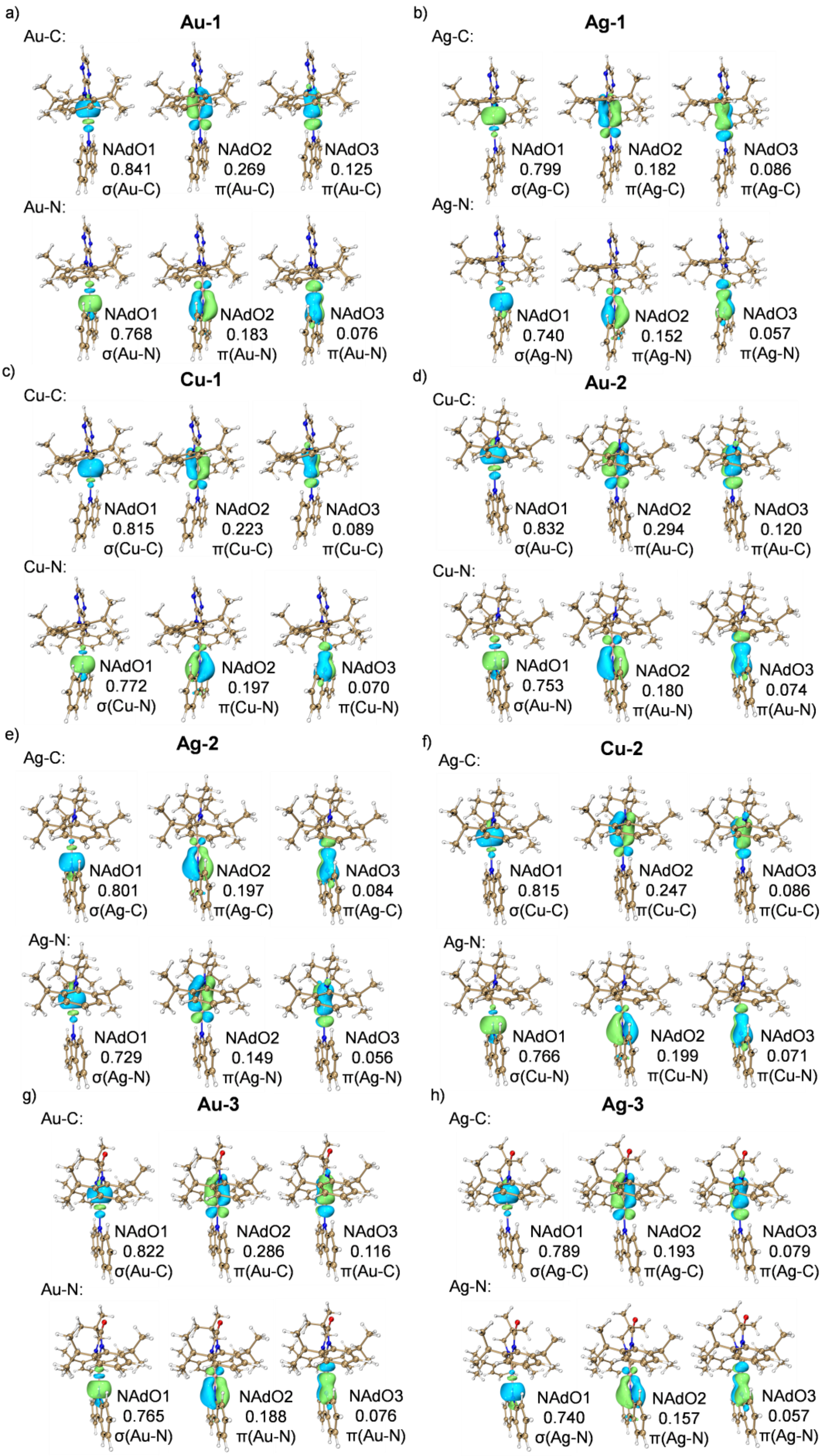
a)
Au-1
Au-C:
NAdO1
0.841
σ(Au-C)
NAdO2
0.269
π(Au-C)
NAdO3
0.125
π(Au-C)
Au-N:
NAdO1
0.768
σ(Au-N)
NAdO2
0.183
π(Au-N)
NAdO3
0.076
π(Au-N)
b)
Ag-1
Ag-C:
NAdO1
0.799
σ(Ag-C)
NAdO2
0.182
π(Ag-C)
NAdO3
0.086
π(Ag-C)
Ag-N:
NAdO1
0.740
σ(Ag-N)
NAdO2
0.152
π(Ag-N)
NAdO3
0.057
π(Ag-N)
c)
Cu-1
Cu-C:
NAdO1
0.815
σ(Cu-C)
NAdO2
0.223
π(Cu-C)
NAdO3
0.089
π(Cu-C)
Cu-N:
NAdO1
0.772
σ(Cu-N)
NAdO2
0.197
π(Cu-N)
NAdO3
0.070
π(Cu-N)
d)
Au-2
Cu-C:
NAdO1
0.832
σ(Au-C)
NAdO2
0.294
π(Au-C)
NAdO3
0.120
π(Au-C)
Cu-N:
NAdO1
0.753
σ(Au-N)
NAdO2
0.180
π(Au-N)
NAdO3
0.074
π(Au-N)
e)
Ag-2
Ag-C:
NAdO1
0.801
σ(Ag-C)
NAdO2
0.197
π(Ag-C)
NAdO3
0.084
π(Ag-C)
Ag-N:
NAdO1
0.729
σ(Ag-N)
NAdO2
0.149
π(Ag-N)
NAdO3
0.056
π(Ag-N)
f)
Cu-2
Ag-C:
NAdO1
0.815
σ(Cu-C)
NAdO2
0.247
π(Cu-C)
NAdO3
0.086
π(Cu-C)
Ag-N:
NAdO1
0.766
σ(Cu-N)
NAdO2
0.199
π(Cu-N)
NAdO3
0.071
π(Cu-N)
g)
Au-3
Au-C:
NAdO1
0.822
σ(Au-C)
NAdO2
0.286
π(Au-C)
NAdO3
0.116
π(Au-C)
Au-N:
NAdO1
0.765
σ(Au-N)
NAdO2
0.188
π(Au-N)
NAdO3
0.076
π(Au-N)
h)
Ag-3
Ag-C:
NAdO1
0.789
σ(Ag-C)
NAdO2
0.193
π(Ag-C)
NAdO3
0.079
π(Ag-C)
Ag-N:
NAdO1
0.740
σ(Ag-N)
NAdO2
0.157
π(Ag-N)
NAdO3
0.057
π(Ag-N)

i) **Cu-3**

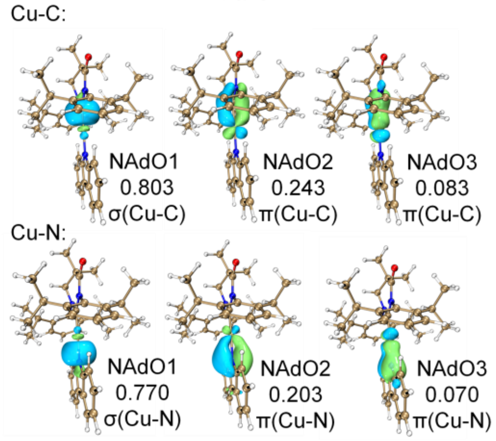

Figure S36. Three major NAdOs of the M-C and M-N bonds for a) **Au-1**, b) **Ag-1**, and c) **Cu-1**, d) **Au-2**, e) **Ag-2**, f) **Cu-2**, g) **Au-3**, h) **Ag-3**, and i) **Cu-3** in optimized semi-coplanar geometries in the T_1 excited state.

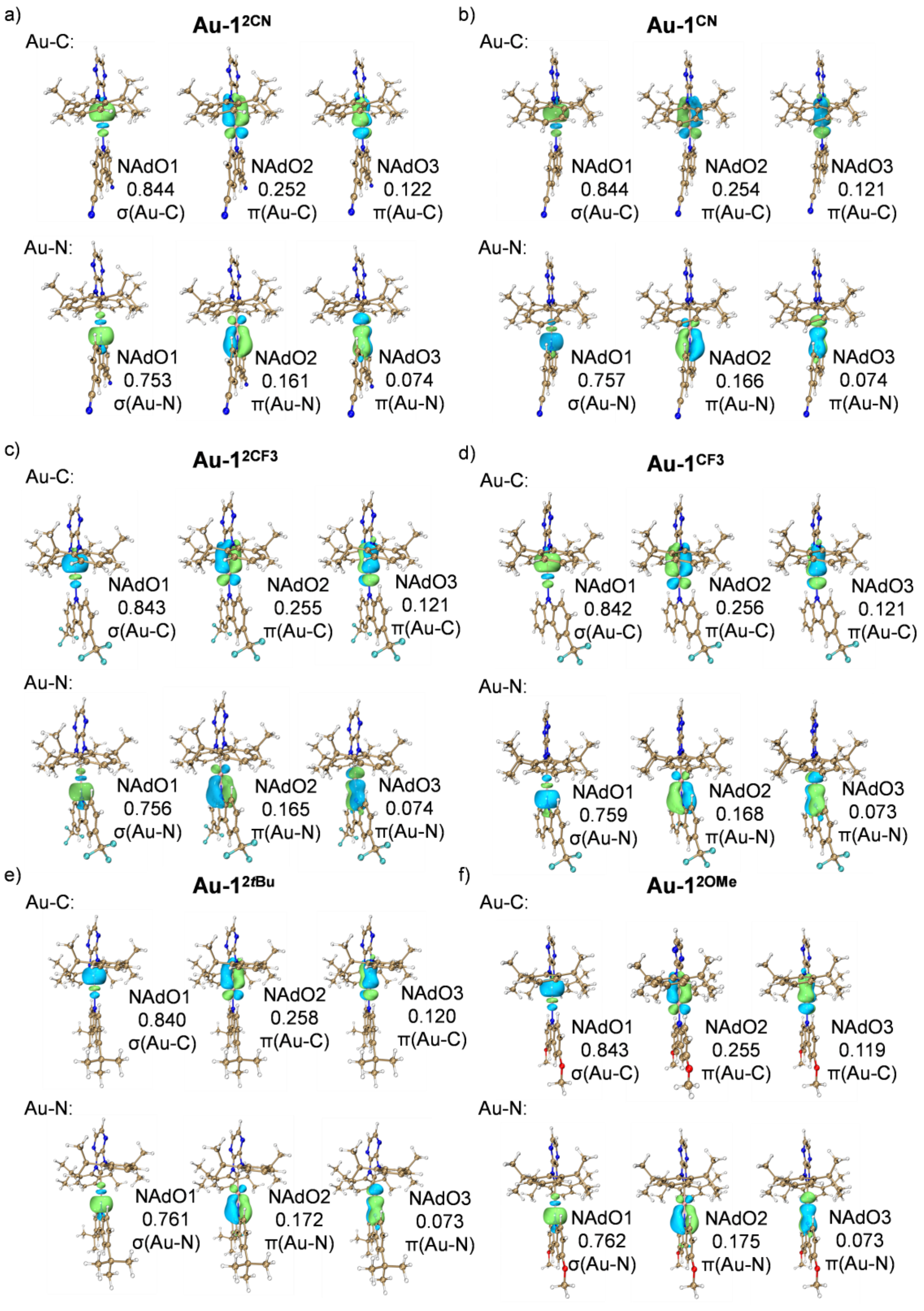
a)
Au-1^{2CN}
Au-C:
NAdO1
0.844
σ(Au-C)
NAdO2
0.252
π(Au-C)
NAdO3
0.122
π(Au-C)
Au-N:
NAdO1
0.753
σ(Au-N)
NAdO2
0.161
π(Au-N)
NAdO3
0.074
π(Au-N)
b)
Au-1^{CN}
Au-C:
NAdO1
0.844
σ(Au-C)
NAdO2
0.254
π(Au-C)
NAdO3
0.121
π(Au-C)
Au-N:
NAdO1
0.757
σ(Au-N)
NAdO2
0.166
π(Au-N)
NAdO3
0.074
π(Au-N)
c)
Au-1^{2CF3}
Au-C:
NAdO1
0.843
σ(Au-C)
NAdO2
0.255
π(Au-C)
NAdO3
0.121
π(Au-C)
Au-N:
NAdO1
0.756
σ(Au-N)
NAdO2
0.165
π(Au-N)
NAdO3
0.074
π(Au-N)
d)
Au-1^{CF3}
Au-C:
NAdO1
0.842
σ(Au-C)
NAdO2
0.256
π(Au-C)
NAdO3
0.121
π(Au-C)
Au-N:
NAdO1
0.759
σ(Au-N)
NAdO2
0.168
π(Au-N)
NAdO3
0.073
π(Au-N)
e)
Au-1^{2tBu}
Au-C:
NAdO1
0.840
σ(Au-C)
NAdO2
0.258
π(Au-C)
NAdO3
0.120
π(Au-C)
Au-N:
NAdO1
0.761
σ(Au-N)
NAdO2
0.172
π(Au-N)
NAdO3
0.073
π(Au-N)
f)
Au-1^{2OMe}
Au-C:
NAdO1
0.843
σ(Au-C)
NAdO2
0.255
π(Au-C)
NAdO3
0.119
π(Au-C)
Au-N:
NAdO1
0.762
σ(Au-N)
NAdO2
0.175
π(Au-N)
NAdO3
0.073
π(Au-N)

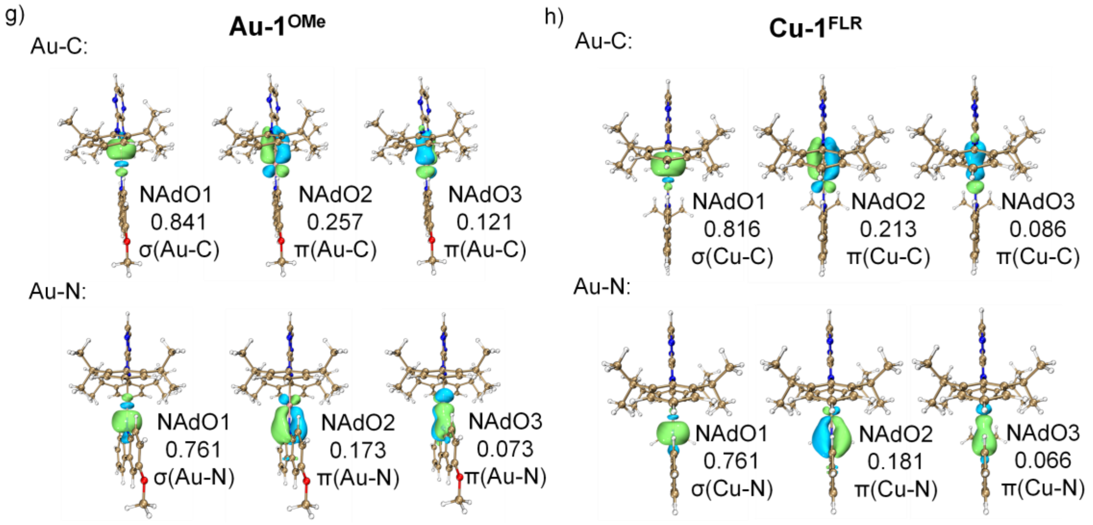

Figure S37. Three major NAdOs of the M-C and M-N bonds for **Au-1$^{\mathbf{2CN}}$**, **Au-1$^{\mathbf{CN}}$**, **Au-1$^{\mathbf{2CF3}}$**, **Au-1$^{\mathbf{CF3}}$**, **Au-1$^{\mathbf{2}t\mathbf{Bu}}$**, **Au-1$^{\mathbf{2OMe}}$**, **Au-1$^{\mathbf{OMe}}$**, and **Cu-1$^{\mathbf{FLR}}$** in optimized semi-coplanar geometries in the S_1 excited state.

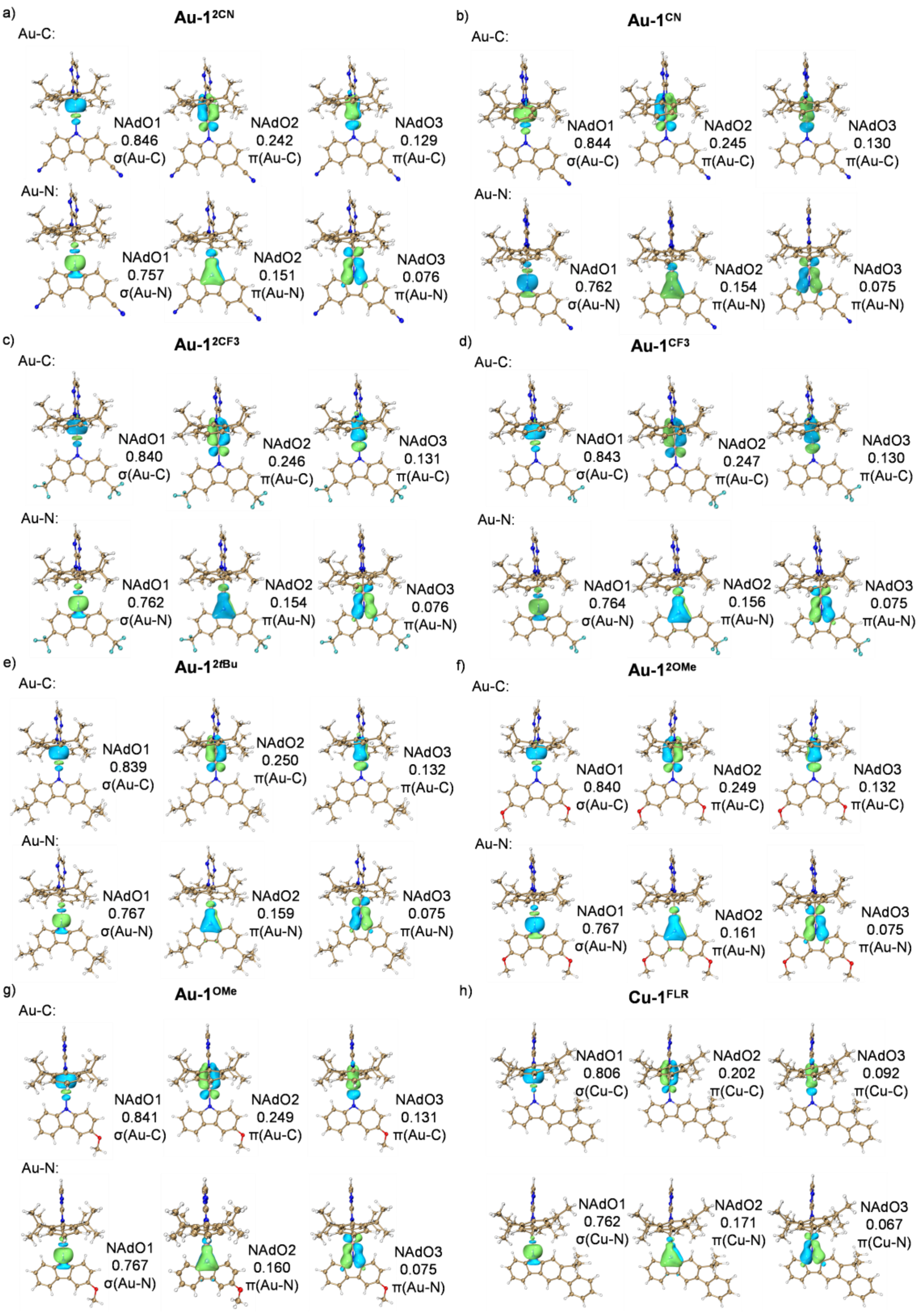

Figure S38. Three major NAdOs of the M-C and M-N bonds for **Au-1^{2CN}**, **Au-1^{CN}**, **Au-1^{2CF3}**, **Au-1^{CF3}**, **Au-1^{2tBu}**, **Au-1^{2OMe}**, **Au-1^{OMe}**, and **Cu-1^{FLR}** in optimized orthogonal geometries in the S_1 excited state.

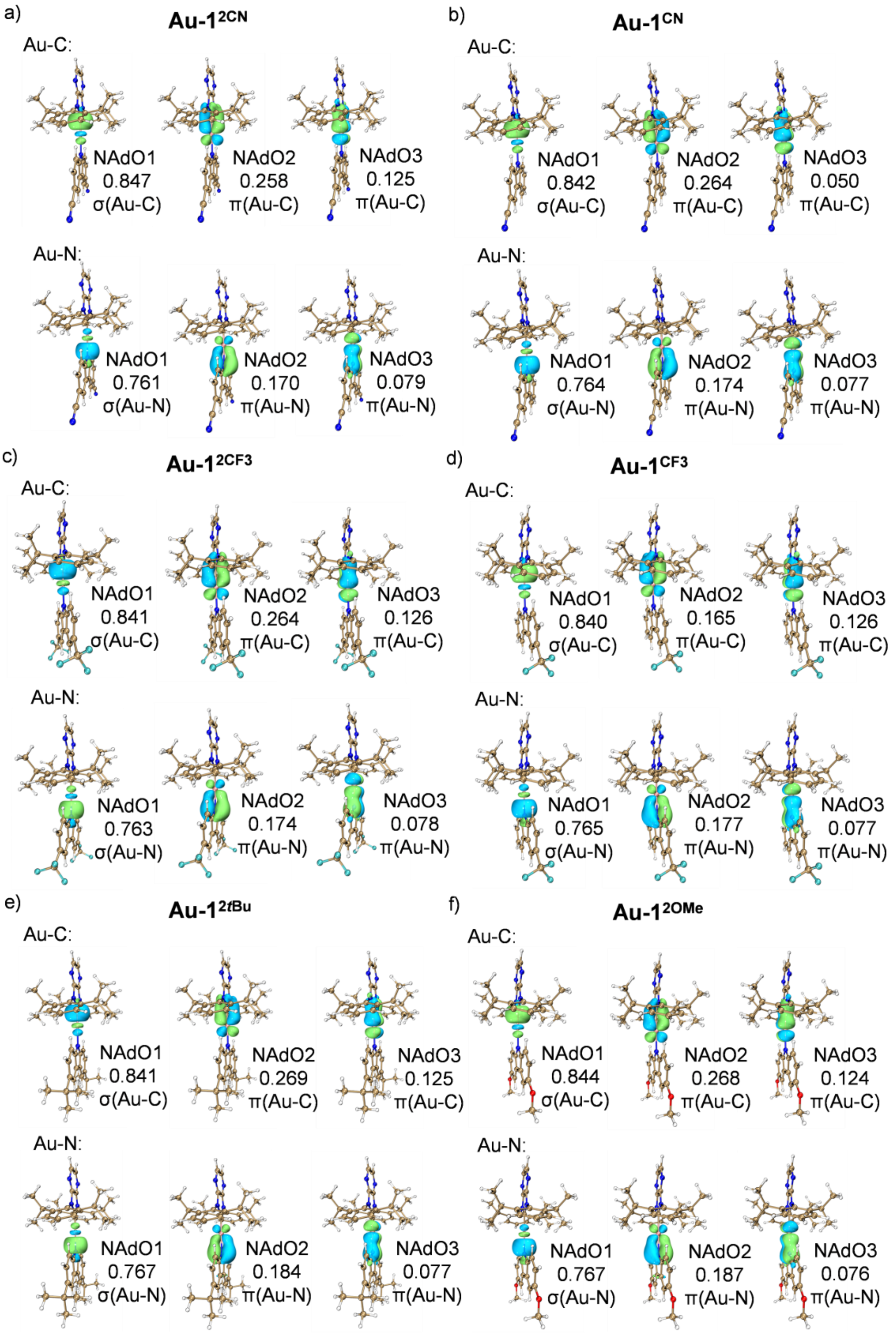
a)
Au-1^{2CN}
Au-C:
NAdO1 0.847 σ(Au-C)
NAdO2 0.258 π(Au-C)
NAdO3 0.125 π(Au-C)
Au-N:
NAdO1 0.761 σ(Au-N)
NAdO2 0.170 π(Au-N)
NAdO3 0.079 π(Au-N)
b)
Au-1^{CN}
Au-C:
NAdO1 0.842 σ(Au-C)
NAdO2 0.264 π(Au-C)
NAdO3 0.050 π(Au-C)
Au-N:
NAdO1 0.764 σ(Au-N)
NAdO2 0.174 π(Au-N)
NAdO3 0.077 π(Au-N)
c)
Au-1^{2CF3}
Au-C:
NAdO1 0.841 σ(Au-C)
NAdO2 0.264 π(Au-C)
NAdO3 0.126 π(Au-C)
Au-N:
NAdO1 0.763 σ(Au-N)
NAdO2 0.174 π(Au-N)
NAdO3 0.078 π(Au-N)
d)
Au-1^{CF3}
Au-C:
NAdO1 0.840 σ(Au-C)
NAdO2 0.165 π(Au-C)
NAdO3 0.126 π(Au-C)
Au-N:
NAdO1 0.765 σ(Au-N)
NAdO2 0.177 π(Au-N)
NAdO3 0.077 π(Au-N)
e)
Au-1^{2tBu}
Au-C:
NAdO1 0.841 σ(Au-C)
NAdO2 0.269 π(Au-C)
NAdO3 0.125 π(Au-C)
Au-N:
NAdO1 0.767 σ(Au-N)
NAdO2 0.184 π(Au-N)
NAdO3 0.077 π(Au-N)
f)
Au-1^{2OMe}
Au-C:
NAdO1 0.844 σ(Au-C)
NAdO2 0.268 π(Au-C)
NAdO3 0.124 π(Au-C)
Au-N:
NAdO1 0.767 σ(Au-N)
NAdO2 0.187 π(Au-N)
NAdO3 0.076 π(Au-N)

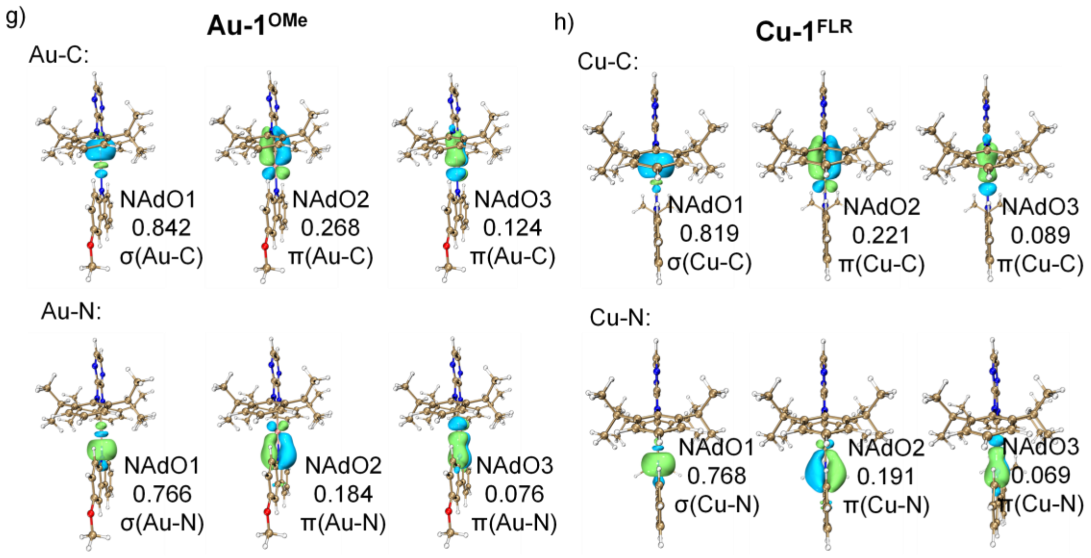

Figure S39. Three major NAdOs of the M-C and M-N bonds for **Au-1^{2CN}**, **Au-1^{CN}**, **Au-1^{2CF3}**, **Au-1^{CF3}**, **Au-1^{2tBu}**, **Au-1^{2OMe}** **Au-1^{OMe}**, and **Cu-1^{FLR}** in optimized orthogonal geometries in the T_1 excited state.

Table S12. Eigenvalues of NAdOs representing M-C and M-N σ- and π-interactions in CMA emitters in semi-coplanar and orthogonal geometries.

NAdOs	**Au-1**	**Ag-1**	**Cu-1**	**Au-2**	**Ag-2**	**Cu-2**	**Au-3**	**Ag-3**	**Cu-3**
S_1 semi-coplanar									
σ(M-C)	0.844	0.796	0.810	0.825	0.792	0.809	0.819	0.784	0.798
π(M···C)	0.376	0.260	0.298	0.393	0.271	0.317	0.382	0.258	0.308
σ(M-N)	0.761	0.734	0.763	0.743	0.718	0.753	0.756	0.730	0.756
π(M···N)	0.244	0.201	0.250	0.223	0.188	0.235	0.239	0.200	0.243
S_1 orthogonal									
σ(M-C)	0.842	0.793	0.814	0.831	0.795	0.812	0.820	0.787	0.799
π(M···C)	0.380	0.263	0.299	0.399	0.273	0.318	0.386	0.261	0.309
σ(M-N)	0.767	0.741	0.768	0.752	0.727	0.760	0.764	0.736	0.766
π(M···N)	0.234	0.198	0.244	0.223	0.190	0.240	0.229	0.194	0.240
T_1 semi-coplanar									
σ(M-C)	0.841	0.799	0.815	0.832	0.801	0.815	0.822	0.789	0.803
π(M···C)	0.394	0.268	0.313	0.414	0.281	0.333	0.402	0.272	0.326
σ(M-N)	0.768	0.740	0.772	0.753	0.729	0.766	0.765	0.740	0.770
π(M···N)	0.259	0.209	0.267	0.254	0.205	0.269	0.264	0.214	0.273

NAdOs	**Au-1^{2CN}**	**Au-1^{CN}**	**Au-1^{2CF3}**	**Au-1^{CF3}**	**Au-1^{2tBu}**	**Au-1^{2OMe}**	**Au-1^{OMe}**	**Cu-1^{FLR}**
S_1 semi-coplanar								
σ(M-C)	0.844	0.844	0.843	0.842	0.840	0.843	0.841	0.816
π(M···C)	0.347	0.375	0.376	0.378	0.378	0.374	0.378	0.299
σ(M-N)	0.753	0.757	0.756	0.759	0.761	0.762	0.761	0.761
π(M···N)	0.235	0.239	0.239	0.241	0.245	0.248	0.246	0.247
S_1 orthogonal								
σ(M-C)	0.846	0.844	0.840	0.843	0.839	0.840	0.842	0.806
π(M···C)	0.370	0.375	0.376	0.377	0.382	0.381	0.234	0.294
σ(M-N)	0.757	0.762	0.762	0.764	0.767	0.767	0.767	0.762
π(M···N)	0.226	0.229	0.230	0.231	0.234	0.236	0.381	0.238
T_1 semi-coplanar								

σ(M-C)	0.847	0.842	0.841	0.840	0.841	0.844	0.842	0.819
π(M···C)	0.383	0.314	0.390	0.391	0.395	0.392	0.392	0.310
σ(M-N)	0.761	0.764	0.763	0.765	0.767	0.767	0.766	0.768
π(M···N)	0.248	0.252	0.252	0.254	0.261	0.264	0.260	0.260

Table S13. Eigenvalues of NAdOs representing M-C and M-N σ- and π-interactions in **M-1** in twisted geometries with various dihedral angles in S_1 and T_1 excited states.

NAdOs	**Au-1** (S_1)	**Ag-1** (S_1)	**Cu-1** (S_1)	**Au-1** (T_1)	**Ag-1** (T_1)	**Cu-1** (T_1)
$\theta_{N1\text{-}C1\text{-}N2\text{-}C2}$ = 0°						
σ(M-C)	0.840	0.799	0.811	0.394	0.268	0.313
π(M···C)	0.379	0.259	0.298	0.841	0.799	0.815
σ(M-N)	0.761	0.734	0.763	0.259	0.209	0.267
π(M···N)	0.245	0.201	0.250	0.768	0.740	0.772
$\theta_{N1\text{-}C1\text{-}N2\text{-}C2}$ = 30°						
σ(M-C)	0.842	0.800	0.812	0.840	0.800	0.813
π(M···C)	0.377	0.258	0.298	0.392	0.267	0.310
σ(M-N)	0.761	0.735	0.763	0.767	0.739	0.769
π(M···N)	0.241	0.200	0.249	0.254	0.207	0.263
$\theta_{N1\text{-}C1\text{-}N2\text{-}C2}$ = 60°						
σ(M-C)	0.836	0.795	0.813	0.840	0.798	0.809
π(M···C)	0.380	0.259	0.297	0.382	0.262	0.303
σ(M-N)	0.764	0.737	0.765	0.766	0.740	0.767
π(M···N)	0.233	0.197	0.242	0.240	0.200	0.250
$\theta_{N1\text{-}C1\text{-}N2\text{-}C2}$ = 90°						
σ(M-C)	0.842	0.793	0.813	0.380	0.263	0.299
π(M···C)	0.380	0.263	0.299	0.842	0.793	0.814
σ(M-N)	0.767	0.741	0.768	0.234	0.198	0.245
π(M···N)	0.234	0.198	0.244	0.768	0.741	0.769

S2.3.7. ETS-NOCV analysis

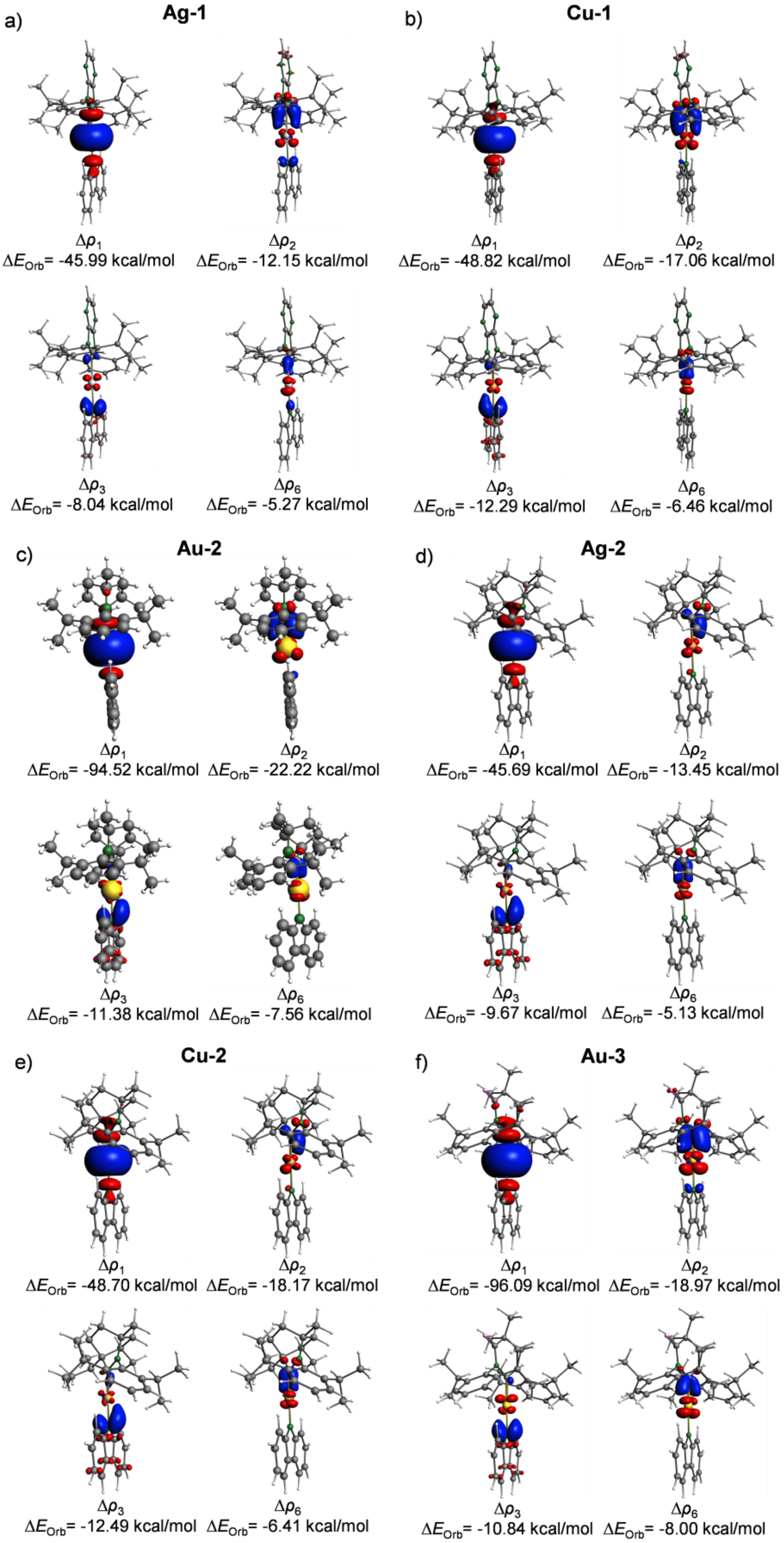

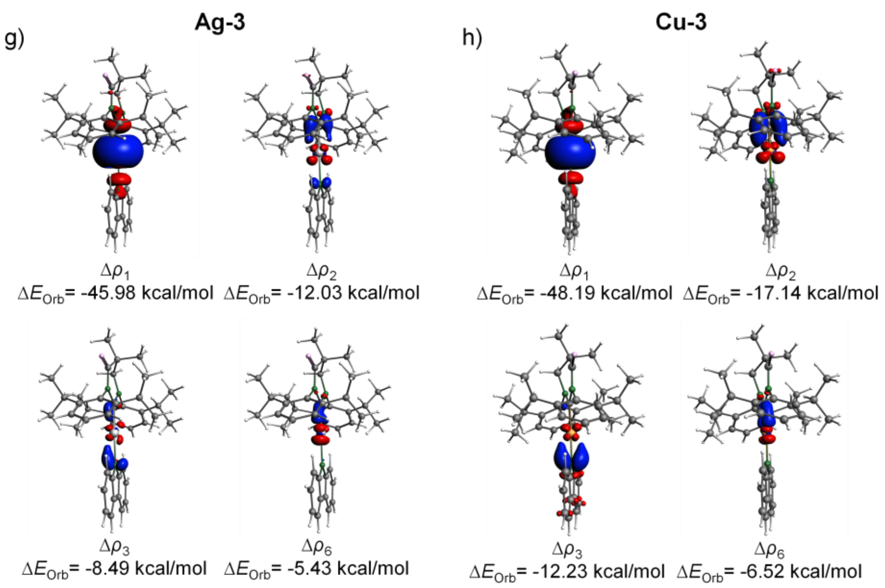

Figure S40. Major ETS-NOCV deformation density contributions to the M-C/M-N σ- and π-interactions in a) **Ag-1**, b) **Cu-1**, c) **Au-2**, d) **Ag-2**, e) **Cu-2**, f) **Au-3**, g) **Ag-3**, h) **Cu-3** in optimized semi-coplanar geometries in the S_1 excited state. Isovalue = 0.002. Electron density transfer from red to blue.

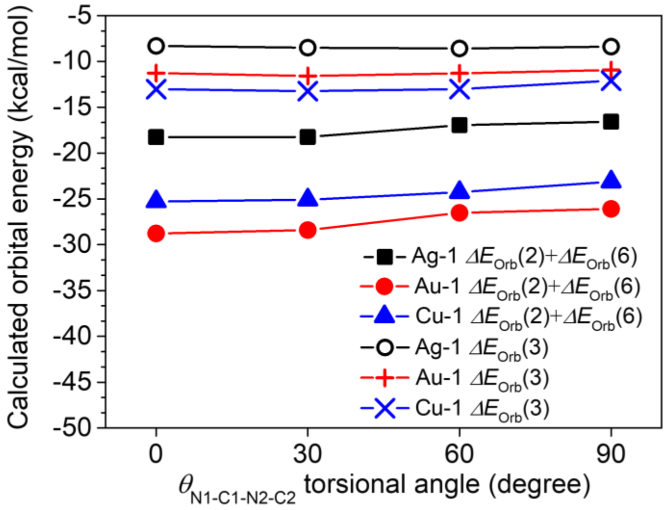

Figure S41. Calculated orbital energies $\Delta E_{\mathrm{Orb}}(3)$ of deformation density $\Delta\rho_3$ contributing to the π(M···N) interactions and calculated orbital energies [$\Delta E_{\mathrm{Orb}}(2)$ + $\Delta E_{\mathrm{Orb}}(6)$] of deformation densities ($\Delta\rho_2$+$\Delta\rho_6$) contributing to π(M···C) interactions for **M-1** in twisted geometires with various dihedral angles in the T_1 excited state.

Table S14. Orbital energies of major ETS-NOCV deformation densities representing the M-C/M-N σ- and π-interactions in **M-1**, **M-2**, and **M-3** in optimized semi-coplananr and orthogonal geometries in the S_1 excited state, and in optimized semi-coplanar geometries in the T_1 excited state. $\Delta\rho_1$: σ-bonding (C-p_z and N-p_z → metal-d_{z^2}), $\Delta\rho_2$: π-interaction (metal's (n+1)p hybridized nd_{xz} → C-p_x), $\Delta\rho_3$: π-interaction (metal's (n+1)p hybridized nd_{xz} → N-p_x), $\Delta\rho_6$: π-interaction (metal's (n+1)p hybridized nd_{yz} → C-p_y).

ΔE_{orb} (kcal/mol)	**Au-1**	**Ag-1**	**Cu-1**	**Au-2**	**Ag-2**	**Cu-2**	**Au-3**	**Ag-3**	**Cu-3**
S_1 semi-coplanar									
$\Delta\rho_1$	-97.08	-45.99	-48.82	-94.52	-45.69	-48.70	-96.09	-45.98	-48.19
$\Delta\rho_2$	-18.96	-12.15	-17.06	-22.22	-13.45	-18.17	-18.97	-12.03	-17.14
$\Delta\rho_3$	-10.85	-8.04	-12.29	-11.38	-9.67	-12.49	-10.84	-8.49	-12.23
$\Delta\rho_6$	-7.98	-5.27	-6.46	-7.56	-5.13	-6.41	-8.00	-5.34	-6.54
Total	-181.90	-107.26	-129.40	-181.90	-108.17	-130.47	-183.90	-110.71	-132.25
S_1 orthogonal									
$\Delta\rho_1$	-98.00	-47.13	-49.26	-97.27	-47.17	-49.44	-97.41	-46.44	-48.72
$\Delta\rho_2$	-17.18	-10.99	-15.88	-19.77	-12.84	-17.56	-17.17	-10.60	-16.10
$\Delta\rho_3$	-10.91	-8.38	-12.08	-10.58	-8.63	-12.28	-11.55	-8.93	-12.56
$\Delta\rho_6$	-8.87[a)]	-5.57	-7.16	-8.35	-5.43	-6.80	-9.10[a)]	-5.95[a)]	-[b)]
Total	-181.21	-107.39	-128.24	-182.44	-110.14	-131.50	-184.77	-110.71	-132.66
T_1 semi-coplanar									
$\Delta\rho_1$	-98.48	-46.93	-49.76	-97.61	-47.33	-49.91	-98.42	-47.36	-49.59
$\Delta\rho_2$	-20.33	-12.75	-18.38	-21.60	-13.35	-19.31	-21.21	-13.17	-19.37
$\Delta\rho_3$	-11.27	-8.31	-13.03	-12.50	-10.22	-13.53	-11.37	-8.60	-13.27
$\Delta\rho_6$	-8.45	-5.52	-6.89	-7.81	-5.35	-6.63	-8.36	-5.75	-6.87
Total	-185.98	-109.72	-133.23	-186.05	-111.78	-135.23	-190.44	-114.90	-137.95

[a)] the orbital energy of ETS-NOCV deformation density $\Delta\rho_5$ representing electron transfer from the metal's (n+1)p-nd_{yz} hybridized orbital to the C-p_y orbital were counted. [b]no corresponding ETS-NOCV deformation density was found.

Table S15. Orbital energies of major ETS-NOCV deformation densities representing the M-C/M-N σ- and π-interactions in **M-1** in twisted geometries with various dihedral angles in S_1 and T_1 excited states. $\Delta\rho_1$: σ-bonding (C-p_z and N-p_z → metal-d_{z^2}), $\Delta\rho_2$: π-interaction (metal's (n+1)p hybridized nd_{xz} → C-p_x), $\Delta\rho_3$: π-interaction (metal's (n+1)p hybridized nd_{xz} → N-p_x), $\Delta\rho_6$: π-interaction (metal's (n+1)p hybridized nd_{yz} → C-p_y).

ΔE_{orb} (kcal/mol)	**Au-1** (S_1)	**Ag-1** (S_1)	**Cu-1** (S_1)	**Au-1** (T_1)	**Ag-1** (T_1)	**Cu-1** (T_1)
$\theta_{N1-C1-N2-C2}$ = 0°						
$\Delta\rho_1$	-96.74	-45.97	-48.85	-98.48	-46.93	-49.77
$\Delta\rho_2$	-19.06	-12.12	-17.12	-20.33	-12.75	-18.39
$\Delta\rho_3$	-10.71	-8.04	-12.27	-11.27	-8.31	-13.03
$\Delta\rho_6$	-7.98	-5.26	-6.44	-8.45	-5.52	-6.89
Total	-181.44	-107.07	-129.59	-185.98	-109.73	-133.26
$\theta_{N1-C1-N2-C2}$ = 30°						
$\Delta\rho_1$	-97.15	-46.06	-48.89	-98.53	-46.61	-49.45
$\Delta\rho_2$	-18.67	-11.98	-16.99	-19.98	-12.70	-18.22
$\Delta\rho_3$	-10.98	-8.51	-12.34	-11.58	-8.50	-13.25
$\Delta\rho_6$	-8.06	-5.34	-6.48	-8.42	-5.55	-6.87
Total	-181.69	-107.55	-129.56	-185.74	-109.06	-132.23
$\theta_{N1-C1-N2-C2}$ = 60°						
$\Delta\rho_1$	-97.42	-46.72	-49.00	-97.90	-46.92	-48.86
$\Delta\rho_2$	-17.71	-11.22	-16.25	-17.89	-11.50	-17.03
$\Delta\rho_3$	-11.33	-8.57	-12.63	-11.29	-8.59	-13.02
$\Delta\rho_6$	-9.59[a]	-5.56	-7.29	-8.63	-5.45	-7.25
Total	-180.72	-107.20	-128.05	-182.63	-108.20	-129.80
$\theta_{N1-C1-N2-C2}$ = 90°						
$\Delta\rho_1$	-98.02	-47.17	-49.23	-98.08	-47.16	-49.32
$\Delta\rho_2$	-17.19	-11.00	-15.87	-17.21	-11.00	-15.92
$\Delta\rho_3$	-10.92	-8.46	-12.06	-10.94	-8.39	-12.11
$\Delta\rho_6$	-8.88[a]	-5.56	-7.17	-8.89[a]	-5.58	-7.20
Total	-181.25	-107.61	-128.17	-181.39	-107.47	-128.45

[a)] the orbital energy of ETS-NOCV deformation density $\Delta\rho_5$ representing electron transfer from the metal's (n+1)p-nd_{yz} hybridized orbital to the C-p_y orbital were counted.

S2.3.8. CDA analysis

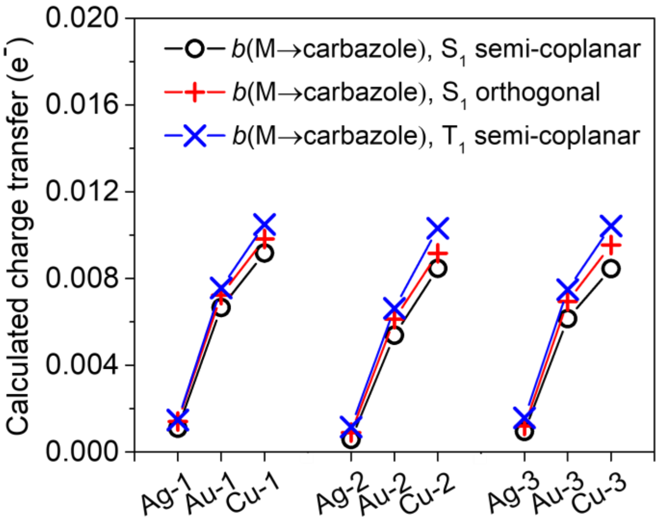

Figure S42. Calculated electron transfer from metal to carbazole ligand for **M-1**, **M-2**, and **M-3** in optimized semi-coplanar and orthogonal geometries in the S_1 excited state and in optimized semi-coplanar geometries in the T_1 excited state.

Table S16. CDA results of **M-1**, **M-2**, and **M-3** in the optimized semi-coplanar and orthogonal geometries in the S_1 excited state, and in optimized semi-coplanar geometries in the T_1 excited state. Donation (*d*) from carbene (Cb) or carbazole (Cz) ligands to metal ions, and back-donation (*b*) from metal ion to ligands are listed.

Charge transfer (e^-)	**Au-1**	**Ag-1**	**Cu-1**	**Au-2**	**Ag-2**	**Cu-2**	**Au-3**	**Ag-3**	**Cu-3**
S_1 semi-coplanar									
d(Cb→M)	0.367	0.375	0.408	0.223	0.233	0.269	0.372	0.398	0.427
b(M→Cb)	0.064	0.033	0.037	0.070	0.038	0.041	0.069	0.037	0.039
d(Cz→M)	0.220	0.234	0.268	0.223	0.233	0.269	0.221	0.233	0.268
b(M→Cz)	0.007	0.001	0.009	0.005	0.001	0.008	0.006	0.001	0.008
S_1 orthogonal									
d(Cb→M)	0.343	0.360	0.381	0.360	0.399	0.432	0.354	0.383	0.405
b(M→Cb)	0.064	0.034	0.037	0.072	0.040	0.042	0.071	0.038	0.040
d(Cz→M)	0.220	0.234	0.269	0.222	0.234	0.269	0.220	0.234	0.269
b(M→Cz)	0.007	0.001	0.010	0.006	0.001	0.009	0.007	0.001	0.010
T_1 semi-coplanar									
d(Cb→M)	0.354	0.374	0.398	0.364	0.405	0.440	0.374	0.402	0.423
b(M→Cb)	0.065	0.035	0.038	0.072	0.040	0.043	0.073	0.040	0.041
d(Cz→M)	0.219	0.234	0.268	0.221	0.234	0.269	0.219	0.234	0.269
b(M→Cz)	0.008	0.001	0.010	0.007	0.001	0.010	0.007	0.002	0.010

S2.3.9. Electron-hole analysis

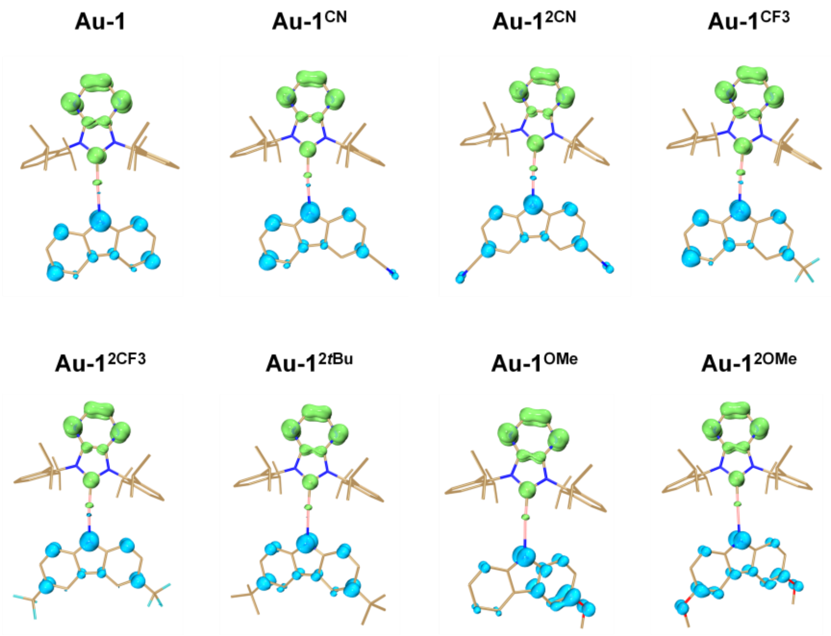

Figure S43. Calculated electron-hole plot of $S_0 \rightarrow S_1$ transition in the semi-coplanar geometries of emitters **Au-1**, **Au-1^{CN}**, **Au-1^{2CN}**, **Au-1^{CF3}**, **Au-1^{2CF3}**, **Au-1^{2tBu}**, **Au-1^{OMe}**, and **Au-1^{2OMe}**. Electron and hole distributions are represented by green and blue isosurfaces, respectively.

Table S17. Calculated electron-hole wavefunction overlap ($O_{h,e}$) and electron-hole centroid distance (Δr) of $S_0 \rightarrow S_1$ transition in the semi-coplanar geometries of emitters **Au-1**, **Au-1^{CN}**, **Au-1^{2CN}**, **Au-1^{CF3}**, **Au-1^{2CF3}**, **Au-1^{2tBu}**, **Au-1^{OMe}**, **Au-1^{2OMe}**, **Cu-1**, and **Cu-1^{FLR}**.

	**Au-1**	**Au-1^{CN}**	**Au-1^{2CN}**	**Au-1^{CF3}**	**Au-1^{2CF3}**
$O_{h,e}$	0.246	0.238	0.240	0.236	0.237
Δr (Å)	7.587	7.601	7.636	7.564	7.508
	**Au-1^{2tBu}**	**Au-1^{OMe}**	**Au-1^{2OMe}**	**Cu-1**	**Cu-1^{FLR}**
$O_{h,e}$	0.252	0.244	0.255	0.233	0.223
Δr (Å)	7.755	7.974	8.082	7.271	7.908

S2.3.10. Calculation of excited state energies and radiative rate constants of emitters

Table S18. Adiabatic energies of the S_1 and T_1 states ($E_{S1,\theta}$ and $E_{T1,\theta}$), adiabatic S_1 and T_1 energy gap ($\Delta E_{S1\text{-}T1,\theta}$), oscillator strength for the $S_1 \rightarrow S_0$ transition ($f_{S1\rightarrow S0,\theta}$), Marcus reorganization energies ($\lambda_{T,\theta}$ and $\lambda_{S,\theta}$), spin-orbit coupling (SOC) matrix elements $|\langle\psi_{S1}|\hat{\mathcal{H}}_{SO}|\psi_{T1}\rangle|$ and $|\langle\psi_{T1}|\hat{\mathcal{H}}_{SO}|\psi_{S1}\rangle|$, radiative rate constants ($k_{S1,\theta}$ and $k_{T1,\theta}$), intersystem crossing rate constants ($k_{ISC,\theta}$), reverse intersystem crossing rate constants ($k_{rISC,\theta}$), and delayed fluorescence rate constants ($k_{TADF,\theta}$) calculated for rotamers of emitters **M-1**, **M-2**, and **M-3**, and the calculated overall delayed fluorescence rate constants (k_{TADF}) of the emitters.

θ (°)	$E_{S1,\theta}$ (a.u.)	$E_{T1,\theta}$ (a.u.)	$\Delta E_{S1\text{-}T1,\theta}$ (eV)	$f_{S1\rightarrow S0,\theta}$	$\lambda_{T,\theta}$ (eV)	$\lambda_{S,\theta}$ (eV)	$\lvert\langle\psi_{S1}\lvert\hat{\mathcal{H}}_{SO}\rvert\psi_{T1}\rangle\rvert$ (cm^{-1})	$\lvert\langle\psi_{T1}\lvert\hat{\mathcal{H}}_{SO}\rvert\psi_{S1}\rangle\rvert$ (cm^{-1})	$k_{S1,\theta}$ (s^{-1})	$k_{T1,\theta}$ (s^{-1})	$k_{ISC,\theta}$ (s^{-1})	$k_{rISC,\theta}$ (s^{-1})	$k_{TADF,\theta}$ (s^{-1})
						**Au-1** (k_{TADF} = 3.83×10^{5})							
0	-1996.328253	-1996.333214	0.135	0.1225	0.0112	0.0157	1.43	1.73	1.94×10^{7}	36.7	9.36×10^{3}	4.73×10^{3}	4.77×10^{3}
15	-1996.328343	-1996.333102	0.129	0.1161	0.0123	0.0142	2.73	1.86	1.84×10^{7}	39.9	3.34×10^{5}	5.23×10^{3}	5.18×10^{3}
30	-1996.328238	-1996.332388	0.113	0.0976	0.0126	0.0137	1.93	2.46	1.55×10^{7}	37.1	3.49×10^{6}	1.50×10^{5}	1.22×10^{5}
45	-1996.328081	-1996.33121	0.085	0.0697	0.0114	0.0124	2.37	2.76	1.11×10^{7}	26.8	1.30×10^{8}	1.03×10^{7}	7.59×10^{5}
60	-1996.328933	-1996.330016	0.029	0.0161	0.0088	0.0164	2.58	2.51	2.56×10^{6}	32.3	1.14×10^{10}	3.64×10^{9}	6.18×10^{5}
75	-1996.330057	-1996.330419	0.010	0.0046	0.0017	0.0021	1.13	1.34	7.30×10^{5}	37.8	5.47×10^{9}	5.22×10^{9}	3.57×10^{5}
90	-1996.330385	-1996.330502	0.003	0	0.00002	0.00005	0.05	0.07	0	40.3	7.75×10^{5}	2.15×10^{7}	1.40
105	-1996.330091	-1996.330433	0.009	0.0046	0.0011	0.0022	1.19	1.32	7.30×10^{5}	39.4	6.09×10^{9}	5.34×10^{9}	3.41×10^{5}
120	-1996.329016	-1996.330009	0.027	0.0175	0.0050	0.0218	2.97	2.59	2.78×10^{6}	27.8	1.26×10^{10}	4.05×10^{9}	6.76×10^{5}
135	-1996.328066	-1996.331301	0.088	0.0689	0.0152	0.0179	2.40	5.01	1.09×10^{7}	97.0	4.07×10^{8}	1.08×10^{8}	2.26×10^{6}
150	-1996.328244	-1996.332429	0.114	0.0970	0.0145	0.0193	1.92	5.12	1.54×10^{7}	111	1.04×10^{7}	6.31×10^{6}	3.39×10^{6}
165	-1996.328358	-1996.333076	0.128	0.1154	0.0114	0.0152	1.45	1.92	1.83×10^{7}	43.4	4.35×10^{4}	1.45×10^{4}	1.45×10^{4}

180	-1996.328254	-1996.333254	0.136	0.1223	0.0126	0.0139	1.37	1.72	1.94×10^{7}	37.3	3.39×10^{4}	9.72×10^{2}	1.01×10^{3}
**Ag-1** (k_{TADF} = 4.75×10^{5})													
0	-2007.538791	-2007.541298	0.068	0.0598	0.0046	0.0058	0.55	0.60	7.87×10^{6}	3.69	2.01×10^{5}	1.27×10^{5}	1.24×10^{5}
15	-2007.538720	-2007.541103	0.065	0.0555	0.0049	0.0071	1.06	1.00	7.31×10^{6}	3.45	3.53×10^{6}	2.58×10^{6}	1.59×10^{6}
30	-2007.538517	-2007.540612	0.057	0.0523	0.0089	0.0038	1.46	1.53	6.89×10^{6}	4.99	2.80×10^{8}	1.57×10^{5}	3.76×10^{3}
45	-2007.537785	-2007.539578	0.049	0.0354	0.0104	0.0033	1.84	1.97	4.66×10^{6}	9.41	2.15×10^{9}	5.33×10^{6}	1.15×10^{4}
60	-2007.537772	-2007.538380	0.017	0.0100	0.0045	0.0058	1.50	1.89	1.32×10^{6}	16.7	6.34×10^{9}	5.25×10^{9}	5.96×10^{5}
75	-2007.538463	-2007.538661	0.005	0.0024	0.0014	0.0005	0.80	0.82	3.16×10^{5}	17.2	3.96×10^{9}	5.49×10^{9}	1.84×10^{5}
90	-2007.538667	-2007.538744	0.002	0	0.0007	0.0002	0	0.05	0	3.52	0	3.75×10^{7}	0
105	-2007.538505	-2007.538680	0.005	0.0020	0.0013	0.0002	0.72	0.92	2.63×10^{5}	16.9	3.42×10^{9}	3.97×10^{9}	1.42×10^{5}
120	-2007.537828	-2007.538349	0.014	0.0096	0.0025	0.0072	1.46	1.83	1.26×10^{6}	17.7	2.63×10^{9}	5.50×10^{9}	5.82×10^{5}
135	-2007.537812	-2007.539555	0.047	0.0368	0.0075	0.0094	1.84	1.81	4.84×10^{6}	7.43	1.26×10^{9}	3.10×10^{8}	9.54×10^{5}
150	-2007.538517	-2007.540632	0.058	0.0523	0.0051	0.0054	1.38	1.38	6.89×10^{6}	4.84	1.41×10^{7}	1.78×10^{6}	5.51×10^{5}
165	-2007.538705	-2007.541169	0.067	0.0569	0.0064	0.0062	0.90	0.87	7.49×10^{6}	4.10	9.60×10^{6}	5.67×10^{5}	2.44×10^{5}
180	-2007.538785	-2007.541290	0.068	0.0599	0.0045	0.0084	0.53	0.59	7.89×10^{6}	3.73	1.81×10^{5}	1.11×10^{6}	1.08×10^{6}
**Cu-1** (k_{TADF} = 1.33×10^{5})													
0	-2057.873717	-2057.878361	0.126	0.0876	0.0138	0.0156	1.97	1.93	1.29×10^{7}	24.5	1.14×10^{6}	2.69×10^{4}	2.48×10^{4}
15	-2057.873700	-2057.878304	0.125	0.0808	0.0199	0.0116	3.15	3.68	1.19×10^{7}	27.1	8.04×10^{7}	4.67×10^{3}	6.29×10^{2}
30	-2057.873323	-2057.877314	0.109	0.0717	0.0136	0.0142	4.65	4.9	1.06×10^{7}	22.8	7.31×10^{7}	1.70×10^{6}	2.11×10^{5}
45	-2057.872567	-2057.875906	0.091	0.0579	0.0117	0.0112	5.95	6.66	8.52×10^{6}	83.8	4.69×10^{8}	1.27×10^{7}	2.21×10^{5}
60	-2057.872553	-2057.873447	0.024	0.0102	0.0055	0.0230	4.48	7.23	1.50×10^{6}	1.54×10^{2}	3.74×10^{10}	2.99×10^{10}	6.68×10^{5}
75	-2057.873637	-2057.873962	0.009	0.0026	0.00009	0.0024	2.38	3.06	3.83×10^{5}	1.04×10^{2}	3.00×10^{7}	2.96×10^{10}	3.82×10^{5}
90	-2057.874023	-2057.874179	0.004	0	0.00009	0.0012	0.07	0.31	0	12.1	1.98×10^{7}	5.70×10^{8}	0.406
105	-2057.873128	-2057.874017	0.024	0.0032	0.0148	0.0012	2.52	2.67	4.71×10^{5}	78.4	1.27×10^{10}	2.48×10^{8}	9.09×10^{3}
120	-2057.872337	-2057.875204	0.078	0.0107	0.0588	0.0280	4.35	7.62	1.58×106	88.8	1.90×10^{10}	1.81×10^{9}	1.37×10^{5}
135	-2057.872977	-2057.876994	0.109	0.0687	0.0154	0.0191	5.79	6.60	1.01×10^{7}	48.7	2.64×10^{8}	1.83×10^{7}	6.35×10^{5}

150	-2057.873521	-2057.878081	0.124	0.0797	0.0188	0.0146	4.14	5.35	1.17×10^{7}	48.8	1.06×10^{8}	1.68×10^{5}	1.68×10^{4}
165	-2057.873726	-2057.878292	0.124	0.0852	0.0138	0.0162	2.90	3.17	1.25×10^{7}	35.8	3.46×10^{6}	1.47×10^{5}	1.15×10^{5}
180	-2057.873721	-2057.878317	0.125	0.0872	0.0131	0.0161	2.07	2.03	1.28×10^{7}	25.1	8.71×10^{5}	4.82×10^{4}	4.52×10^{4}
**Au-2** (k_{TADF} = 5.10×10^{5})													
0	-1642.480842	-1642.488447	0.207	0.1087	0.0353	0.0355	5.43	5.44	2.88×10^{7}	3.11×10^{2}	1.19×10^{7}	2.13×10^{3}	1.82×10^{3}
15	-1642.481314	-1642.488194	0.187	0.0872	0.0436	0.0392	2.81	2.80	2.31×10^{7}	1.90×10^{2}	9.78×10^{7}	3.07×10^{4}	6.06×10^{3}
30	-1642.481792	-1642.487428	0.153	0.0637	0.0426	0.0445	1.72	5.03	1.69×10^{7}	2.23×10^{2}	2.24×10^{8}	5.96×10^{6}	4.08×10^{5}
45	-1642.482154	-1642.486117	0.108	0.0352	0.0390	0.0469	6.33	7.45	9.32×10^{6}	4.25×10^{2}	1.61×10^{10}	4.61×10^{8}	2.60×10^{5}
60	-1642.482897	-1642.484534	0.045	0.0105	0.0147	0.0452	8.05	8.81	2.78×10^{6}	6.78×10^{2}	7.67×10^{10}	1.66×10^{10}	4.96×10^{5}
75	-1642.483370	-1642.483962	0.016	0.0018	0.0016	0.0011	7.16	7.34	4.77×10^{5}	1.02×10^{3}	8.97×10^{10}	3.27×10^{10}	1.28×10^{5}
90	-1642.483130	-1642.483545	0.011	0.0002	0.0112	0.0002	4.87	5.55	5.30×10^{4}	2.93×10^{2}	5.77×10^{10}	1.53×10^{9}	1.66×10^{3}
105	-1642.482278	-1642.483260	0.027	0.0054	0.0090	0.0136	2.19	4.79	1.43×10^{6}	4.84×10^{2}	9.30×10^{9}	1.59×10^{10}	9.02×10^{5}
120	-1642.481477	-1642.483641	0.059	0.0240	0.0140	0.0240	1.35	2.28	6.35×10^{6}	69.3	9.86×10^{8}	5.35×10^{8}	2.23×10^{6}
135	-1642.480393	-1642.485044	0.127	0.0477	0.0462	0.0657	4.17	8.59	1.26×10^{7}	4.22×10^{2}	5.37×10^{9}	3.12×10^{8}	6.92×10^{5}
150	-1642.480034	-1642.486904	0.187	0.1028	0.0326	0.0444	3.36	6.68	2.72×10^{7}	4.02×10^{2}	1.30×10^{7}	9.06×10^{7}	2.28×10^{7}
165	-1642.480362	-1642.487994	0.208	0.1231	0.0252	0.0294	4.69	4.26	3.26×10^{7}	3.23×10^{2}	9.52×10^{4}	2.25×10^{2}	5.48×10^{2}
180	-1642.480611	-1642.488443	0.213	0.1261	0.0249	0.0279	4.59	5.23	3.34×10^{7}	3.19×10^{2}	3.38×10^{4}	67.7	3.87×10^{2}
**Ag-2** (k_{TADF} = 9.36×10^{5})													
0	-1653.687266	-1653.691255	0.109	0.0660	0.0125	0.0123	2.42	2.22	1.62×10^{7}	64.1	1.02×10^{7}	1.08×10^{5}	6.63×10^{4}
15	-1653.687452	-1653.691098	0.099	0.0578	0.0139	0.0154	2.54	2.23	1.42×10^{7}	42.5	8.64×10^{7}	2.57×10^{6}	3.56×10^{5}
30	-1653.687401	-1653.690529	0.085	0.0468	0.0142	0.0139	2.85	2.89	1.15×10^{7}	39.7	5.66×10^{8}	1.92×10^{7}	3.71×10^{5}
45	-1653.687088	-1653.689426	0.064	0.0332	0.0104	0.0159	3.16	3.62	8.16×10^{6}	52.8	1.80×10^{9}	5.61×10^{8}	1.94×10^{6}
60	-1653.686660	-1653.688061	0.038	0.0096	0.0186	0.0151	2.67	3.76	2.23×10^{6}	66.9	1.11×10^{10}	4.79×10^{9}	7.13×10^{5}
75	-1653.686757	-1653.687089	0.009	0.0020	0.0006	0.0008	1.63	1.74	4.92×10^{5}	52.4	8.95×10^{9}	8.27×10^{9}	2.36×10^{5}
90	-1653.686485	-1653.686680	0.005	0.0001	0.00006	0.0002	0.97	1.06	2.46×10^{4}	25.4	1.15×10^{6}	4.65×10^{9}	2.46×10^{4}
105	-1653.686094	-1653.686628	0.015	0.0058	0.0021	0.0047	1.85	2.29	1.43×10^{6}	57.1	9.36×10^{9}	9.19×10^{9}	7.06×10^{5}

120	-1653.685975	-1653.687085	0.030	0.0156	0.0043	0.0057	2.69	3.07	3.83×10^{6}	83.5	6.32×10^{9}	3.55×10^{9}	1.38×10^{6}
135	-1653.685713	-1653.688341	0.071	0.0272	0.0289	0.0221	3.22	3.06	6.68×10^{6}	55.0	8.55×10^{9}	3.45×10^{8}	2.59×10^{5}
150	-1653.686471	-1653.689905	0.093	0.0560	0.0112	0.0150	2.63	2.23	1.38×10^{7}	62.6	4.68×10^{7}	5.12×10^{6}	1.09×10^{6}
165	-1653.687229	-1653.690859	0.099	0.0674	0.0013	0.0176	2.33	1.98	1.66×10^{7}	67.7	0	4.25×10^{6}	4.25×10^{6}
180	-1653.687242	-1653.691249	0.109	0.0685	0.0103	0.0118	2.29	2.14	1.68×10^{7}	65.0	1.35×10^{6}	6.63×10^{4}	6.14×10^{4}
**Cu-2** (k_{TADF} = 1.11×10^{5})													
0	-1704.023054	-1704.031375	0.226	0.0981	0.0307	0.0355	8.55	12.27	2.13×10^{7}	5.70×10^{2}	5.71×10^{5}	1.38×10^{3}	1.91×10^{3}
15	-1704.023480	-1704.031130	0.208	0.0817	0.0373	0.0413	12.45	10.83	1.78×10^{7}	3.36×10^{2}	1.03×10^{8}	6.37×10^{4}	9.68×10^{3}
30	-1704.023745	-1704.030301	0.178	0.0585	0.0455	0.0466	16.33	15.53	1.27×10^{7}	4.40×10^{2}	7.36×10^{9}	7.46×10^{6}	1.33×10^{4}
45	-1704.023843	-1704.028836	0.136	0.0363	0.0435	0.0474	17.48	21.19	7.89×10^{6}	7.42×10^{2}	5.63×10^{10}	5.41×10^{8}	7.58×10^{4}
60	-1704.023719	-1704.026912	0.087	0.0138	0.0399	0.0379	13.56	22.46	3.00×10^{6}	1.01×10^{3}	1.39×10^{11}	1.23×10^{10}	2.44×10^{5}
75	-1704.023726	-1704.024677	0.026	0.0020	0.0035	0.0074	6.23	10.61	4.35×10^{5}	5.54×10^{2}	4.28×10^{10}	7.86×10^{10}	2.82×10^{5}
90	-1704.023333	-1704.023994	0.018	0.0001	0.0005	0.0004	1.62	2.22	2.17×10^{4}	1.83×10^{2}	6.43×10^{7}	1.99×10^{7}	5.27×10^{3}
105	-1704.022476	-1704.023592	0.030	0.0024	0.0087	0.0181	5.13	13.03	5.22×10^{5}	8.73×10^{2}	4.31×10^{10}	9.22×10^{10}	3.56×10^{5}
120	-1704.021967	-1704.024731	0.075	0.0178	0.0243	0.0686	13.52	21.35	3.87×10^{6}	7.42×10^{2}	1.07×10^{11}	2.39×10^{10}	7.07×10^{5}
135	-1704.021801	-1704.027312	0.150	0.0331	0.0662	0.0595	17.10	16.76	7.19×10^{6}	4.89×10^{2}	1.05×10^{11}	2.28×10^{8}	1.61×10^{4}
150	-1704.022346	-1704.029348	0.191	0.0682	0.0429	0.0466	14.80	10.95	1.48×10^{7}	3.24×10^{2}	1.96×10^{9}	1.15×10^{6}	8.94×10^{3}
165	-1704.022684	-1704.030659	0.217	0.0925	0.0321	0.0380	8.86	4.63	2.01×10^{7}	3.51×10^{2}	3.60×10^{6}	1.69×10^{3}	1.78×10^{3}
180	-1704.022995	-1704.031316	0.226	0.1004	0.0289	0.0347	6.57	8.49	2.18×10^{7}	4.47×10^{2}	1.30×10^{5}	4.86×10^{2}	9.30×10^{2}
**Au-3** (k_{TADF} = 3.48×10^{5})													
0	-2003.756115	-2003.763949	0.213	0.1412	0.0238	0.0291	0.88	0.69	3.27×10^{7}	77.8	5.69×10^{2}	2.13	8.00
15	-2003.756168	-2003.763718	0.205	0.1324	0.0250	0.0340	1.93	0.91	3.06×10^{7}	71.1	1.93×10^{4}	8.6×10^{2}	1.57×10^{2}
30	-2003.755858	-2003.762739	0.187	0.1161	0.0245	0.0316	0.69	0.69	2.69×10^{7}	72.0	2.17×10^{4}	2.74×10^{2}	3.46×10^{2}
45	-2003.755000	-2003.760803	0.158	0.0914	0.0230	0.0298	1.23	3.87	2.12×10^{7}	93.5	1.16×10^{6}	2.26×10^{5}	2.15×10^{5}
60	-2003.753949	-2003.758055	0.112	0.0155	0.0800	0.0303	2.66	4.51	3.59×10^{6}	86.4	5.72×10^{9}	4.65×10^{7}	2.90×10^{4}
75	-2003.755968	-2003.756555	0.016	0.0036	0.0023	0.0030	1.56	1.19	8.33×10^{5}	3.09×10^{2}	5.93×10^{9}	2.07×10^{9}	2.16×10^{5}

90	-2003.757149	-2003.757438	0.008	0	0.0004	-0.0003	0.25	0.26	0	1.65×10^{2}	1.99×10^{8}	-[a)]	1.65×10^{2}
105	-2003.757465	-2003.757892	0.012	0.0025	0.0006	0.0024	1.53	1.25	5.79×10^{5}	1.68×10^{2}	3.08×10^{9}	3.71×10^{9}	3.17×10^{5}
120	-2003.756616	-2003.758779	0.059	0.0127	0.0319	0.0771	3.09	11.79	2.94×10^{6}	5.37×10^{2}	1.11×10^{10}	1.25×10^{10}	1.56×10^{6}
135	-2003.756008	-2003.761107	0.139	0.0680	0.0349	0.0408	3.41	12.68	1.57×10^{7}	6.08×10^{2}	7.93×10^{8}	9.42×10^{7}	1.65×10^{6}
150	-2003.756322	-2003.762758	0.175	0.1046	0.0260	0.0325	4.05	9.53	2.42×10^{7}	3.56×10^{2}	6.48×10^{6}	3.23×10^{5}	2.55×10^{5}
165	-2003.756315	-2003.763614	0.199	0.1254	0.0260	0.0317	0.47	5.00	2.90×10^{7}	1.52×10^{2}	5.00×10^{3}	3.04×10^{3}	3.19×10^{3}
180	-2003.756113	-2003.763949	0.213	0.1411	0.0239	0.0290	1.23	0.97	3.27×10^{7}	78.7	1.18×10^{3}	4.12	8.29
**Ag-3** (k_{TADF} = 3.81 $\times10^{5}$)													
0	-2014.965856	-2014.970139	0.117	0.0772	0.0115	0.0150	0.45	0.51	1.54×10^{7}	17.0	4.50×10^{4}	7.35×10^{3}	7.34×10^{3}
15	-2014.965841	-2014.969870	0.110	0.0702	0.0127	0.0142	1.50	1.51	1.40×10^{7}	17.8	3.83×10^{6}	1.35×10^{5}	1.06×10^{5}
30	-2014.965584	-2014.968989	0.093	0.0574	0.0103	0.0123	2.31	2.44	1.15×10^{7}	19.2	2.31×10^{7}	2.32×10^{6}	7.35×10^{5}
45	-2014.964981	-2014.967572	0.070	0.0413	0.0081	0.0095	2.81	3.10	8.25×10^{6}	20.4	2.05×10^{8}	3.62×10^{7}	1.20×10^{6}
60	-2014.964391	-2014.965905	0.041	0.0193	0.0096	0.0075	2.55	3.20	3.85×10^{6}	43.0	6.27×10^{9}	1.41×10^{9}	7.08×10^{5}
75	-2014.964543	-2014.964813	0.007	0.0020	0.0012	0.0015	0.99	1.33	3.99×10^{5}	36.9	5.38×10^{9}	7.07×10^{9}	2.27×10^{5}
90	-2014.964359	-2014.964483	0.003	0.0000	0.0001	-0.00002	0.16	0.16	0	21.2	1.36×10^{8}	-[a)]	21.2
105	-2014.963566	-2014.963767	0.005	0.0013	0.0002	0.0006	0.72	0.79	2.60×10^{5}	23.5	1.00×10^{8}	3.61×10^{9}	2.53×10^{5}
120	-2014.963279	-2014.966188	0.079	0.0422	0.0128	0.0240	2.67	2.97	8.43×10^{6}	26.0	5.81×10^{8}	1.96×10^{8}	2.11×10^{6}
135	-2014.964693	-2014.968315	0.099	0.0558	0.0159	0.0182	2.36	2.82	1.11×10^{7}	29.8	1.76×10^{8}	1.04×10^{7}	5.89×10^{5}
150	-2014.965561	-2014.969474	0.106	0.0653	0.0141	0.0191	1.74	2.21	1.30×10^{7}	25.7	1.79×10^{7}	2.93×10^{6}	1.17×10^{6}
165	-2014.965846	-2014.970023	0.114	0.0732	0.0130	0.0168	1.12	1.11	1.46×10^{7}	19.1	1.42×10^{6}	1.29×10^{5}	1.18×10^{5}
180	-2014.965858	-2014.970154	0.117	0.0773	0.0118	0.0150	0.40	0.39	1.54×10^{7}	16.6	4.12×10^{4}	4.01×10^{3}	4.01×10^{3}
**Cu-3** (k_{TADF} = 7.44 $\times10^{4}$)													
0	-2065.302453	-2065.310319	0.214	0.1046	0.0272	0.0356	1.74	2.02	2.23×10^{7}	86.8	1.78×10^{4}	2.26×10^{2}	3.13×10^{2}
15	-2065.302652	-2065.310029	0.201	0.0924	0.0300	0.0345-	7.38	6.88	1.97×10^{7}	95.1	6.27×10^{6}	1.08×10^{4}	8.32×10^{3}
30	-2065.302505	-2065.308909	0.174	0.0748	0.0276	0.0329	10.32	10.40	1.59×10^{7}	1.61×10^{2}	8.43×10^{7}	4.72×10^{5}	7.48×10^{4}
45	-2065.301845	-2065.306978	0.140	0.0544	0.0238	0.0251	12.19	13.54	1.16×10^{7}	2.78×10^{2}	1.01×10^{9}	8.09×10^{6}	9.10×10^{4}

60	-2065.301482	-2065.304407	0.080	0.0181	0.0312	0.0333	10.42	15.24	3.86×10^{6}	4.72×10^{2}	7.64×10^{10}	7.93×10^{9}	3.63×10^{5}
75	-2065.301526	-2065.302887	0.037	0.0067	0.0109	0.0093	6.97	10.60	1.43×10^{6}	4.23×10^{2}	6.51×10^{10}	3.17×10^{10}	4.86×10^{5}
90	-2065.301028	-2065.301525	0.014	0.0001	0.0005	0.0016	1.31	2.91	2.13×10^{4}	78.7	9.38×10^{8}	1.38×10^{10}	2.00×10^{4}
105	-2065.299841	-2065.300417	0.016	0.0014	0.0001	0.0031	3.02	4.46	2.98×10^{5}	1.16×10^{2}	6.51×10^{10}	3.05×10^{10}	6.98×10^{4}
120	-2065.299464	-2065.305173	0.155	0.0540	0.0348	0.0427	11.90	14.64	1.15×10^{7}	2.21×10^{2}	3.37×10^{9}	3.53×10^{7}	1.19×10^{5}
135	-2065.301126	-2065.307818	0.182	0.0734	0.0332	0.0453	9.93	12.51	1.56×10^{7}	2.16×10^{2}	2.13×10^{8}	2.83×10^{6}	1.92×10^{5}
150	-2065.302101	-2065.309446	0.200	0.0882	0.0324	0.0432	7.72	9.67	1.88×10^{7}	1.89×10^{2}	1.90×10^{7}	1.93×10^{5}	9.61×10^{4}
165	-2065.302439	-2065.310109	0.209	0.0985	0.0293	0.0394	4.97	5.44	2.10×10^{7}	1.26×10^{2}	8.51×10^{5}	9.75×10^{3}	9.50×10^{3}
180	-2065.302446	-2065.310318	0.214	0.1048	0.0271	0.0356	1.59	1.88	2.23×10^{7}	84.4	1.38×10^{4}	1.86×10^{2}	2.70×10^{2}

[a)] $k_{rISC,\theta}$ was not calculated due to the negative value of λ_T. Here, $k_{S1,\theta}$ is equal to 0 s^{-1} and $k_{T1,\theta}$ is very small, resulting in a small calculated $k_{TADF,\theta}$ that is not significantly influenced by $k_{rISC,\theta}$. Therefore,F the impact of absence of calculated k_{rISC} value on the calculated overall k_{TADF} is negligible.

Table S19. STEOM-DLPNO-CCSD calculated adiabatic energies of S_1 and T_1 states ($E_{S1,\theta}$ and $E_{T1,\theta}$), adiabatic S_1 and T_1 energy gap ($\Delta E_{S1\text{-}T1,\theta}$), oscillator strength for $S_1 \rightarrow S_0$ transition ($f_{S1\rightarrow S0,\theta}$), and nature of the $S_0 \rightarrow S_1$ and $S_0 \rightarrow T_1$ transitions of emitters **M-1'**. For the nature of transitions, orbital transitions with contributions larger than 5% are considered. H and L represent HOMO and LUMO, respectively.

θ (°)	$E_{S1,\theta}$ (a.u.)	$E_{T1,\theta}$ (a.u.)	$\Delta E_{S1\text{-}T1,\theta}$ (eV)	$f_{S1\rightarrow S0,\theta}$	$S_0 \rightarrow S_1$ transition nature	$S_0 \rightarrow T_1$ transition nature
				**Au-1'**		
0	-1140.529908	-1140.537068	0.195	0.1116	H→L (83.9%), H→L+1 (5.4%)	H→L (76.2%), H-1→L (5.9%), H-4→L (5.7%)
30	-1140.528748	-1140.536325	0.206	0.0427	H→L (87.2%)	H→L (77.8%), H→L+1 (6.2%)
60	-1140.532236	-1140.534715	0.067	0.0090	H→L (85.9%)	H→L (82.3%), H→L+1 (6.8%)
90	-1140.533456	-1140.533377	-0.002	0	H→L (87.7%)	H→L (83.2%), H→L+1 (8.1%)
120	-1140.53231	-1140.535117	0.076	0.0140	H→L (85.3%), H→L+1 (5.9%)	H→L (79.8%), H→L+1 (7.8%)
150	-1140.528988	-1140.536521	0.205	0.0548	H→L (87.4%)	H→L (77.5%), H→L+1 (6.7%)
180	-1140.529927	-1140.537057	0.194	0.1117	H→L (83.8%), H→L+1 (5.4%)	H→L (76.2%), H→L+1 (5.9%), H-4→L (5.7%)
				**Ag-1'**		
0	-1151.885761	-1151.887976	0.060	0.037	H→L (91.0%)	H→L (81.9%), H→L+1 (7.2%)
30	-1151.885502	-1151.888701	0.087	0.011	H→L (93.9%)	H→L (83.7%), H→L+1 (6.8%)
60	-1151.888873	-1151.890074	0.033	0.002	H→L (92.9%)	H→L (86.3%), H→L+1 (5.7%)
90	-1151.890722	-1151.890753	0.001	0	H→L (88.5%), H→L+1 (5.5%)	H→L (85.9%), H→L+1 (6.3%)
120	-1151.888955	-1151.890173	0.033	0.003	H→L (93.0%)	H→L (86.0%), H→L+1 (5.7%)
150	-1151.885807	-1151.888728	0.079	0.015	H→L (93.7%)	H→L (83.9%), H→L+1 (6.8%)
180	-1151.885761	-1151.887976	0.060	0.037	H→L (90.9%)	H→L (81.9%), H→L+1 (7.3%)
				**Cu-1'**		
0	-2644.942939	-2644.942600	0.366	0.0367	H→L (93.7%)	H→L (70.6%), H-4→L (8.9%), H-14→L (6.8%), H-6→L (6.0%)
30	-2644.941210	-2644.942860	0.345	0.0044	H→L (83.2%)	H→L (75.7%), H-4→L (5.8%), H→L+1 (5.2%), H-6→L (5.1%)

60	-2644.942136	-2644.942853	0.109	0.0033	H→L (91.6%)	H→L (81.4%), H→L+1 (6.5%)
90	-2644.942855	-2644.941889	0.040	0	H→L (91.8%)	H→L (84.3%), H→L+1 (9.1%)
120	-2644.851025	-2644.853248	0.060	0.0033	H→L (91.1%)	H→L (83.8%), H→L+1 (8.9%)
150	-2644.84736	-2644.850804	0.094	0.0175	H→L (91.5%)	H→L (84.3%), H→L+1 (8.7%)
180	-2644.850479	-2644.858812	0.227	0.0728	H→L (84.4%)	H→L (70.6%), H-4→L (9.0%), H-14→L (6.8%), H-6→L (6.0%)

Table S20. DFT/MRCI calculated adiabatic energies of S_1 and T_1 states ($E_{S1,\theta}$ and $E_{T1,\theta}$), and adiabatic S_1 and T_1 energy gap ($\Delta E_{S1\text{-}T1,\theta}$), of emitters **M-1'**.

θ (°)	$E_{S1,\theta}$ (a.u.)	$E_{T1,\theta}$ (a.u.)	$\Delta E_{S1\text{-}T1,\theta}$ (eV)
		**Au-1'**	
0	-1142.777846	-1142.781546	0.101
15	-1142.773228	-1142.779296	0.165
30	-1142.772696	-1142.777873	0.141
45	-1142.773292	-1142.778971	0.155
60	-1142.775064	-1142.77685	0.049
75	-1142.776775	-1142.778374	0.044
90	-1142.779815	-1142.78126	0.039
		**Ag-1'**	
0	-1154.056785	-1154.059522	0.074
15	-1154.052165	-1154.057127	0.135
30	-1154.05243	-1154.059424	0.190
45	-1154.054714	-1154.057448	0.074
60	-1154.056479	-1154.059367	0.079
75	-1154.057882	-1154.059424	0.042
90	-1154.05866	-1154.060185	0.041
		**Cu-1'**	
0	-2647.640539	-2647.643959	0.093
15	-2647.634100	-2647.641693	0.207
30	-2647.634563	-2647.640840	0.171
45	-2647.635401	-2647.641578	0.168
60	-2647.638153	-2647.640792	0.072
75	-2647.640899	-2647.642652	0.048
90	-2647.639893	-2647.643345	0.094

Table S21. Literature-reported TADF emission wavelengths of CMA emitters.

	**Au-1**	**Au-1^{CN}**	**Au-1^{CN2}**	**Au-1^{2tBu}**	**Ag-1**	**Cu-1**
λ_{max} (nm)$^{a)}$ [1-2]	617	550	485	768	676	624
	**Au-2**	**Ag-2**	**Cu-2**			
λ_{max} (nm)$^{a)}$ [6]	512	526	502			
	**Au-3**	**Ag-3**	**Cu-3**			
λ_{max} (nm)$^{a)}$ [7]	544	568	542			
	**Au-4**	**Ag-4**	**Cu-4**			
λ_{max} (nm)$^{b)}$ [7]	502	512	474			
	**Au-5^{MeCN}**	**Ag-5^{MeCN}**	**Cu-5^{MeCN}**	**Au-5^{PhCN}**	**Ag-5^{PhCN}**	**Cu-5^{PhCN}**
λ_{max} (nm)$^{b)}$ [8]	484	476	482	504	498	500
	**Au-5^{Me}**	**Ag-5^{Me}**	**Cu-5^{Me}**	**Au-5^{Ph}**	**Ag-5^{Ph}**	**Cu-5^{Ph}**
λ_{max} (nm)$^{b)}$ [8]	528	530	534	554	558	556
	**Cu-6**	**Cu-6^{CN}**	**Cu-6^{2CN}**			
λ_{max} (nm)[9]	704^{c}	666^{b}	602^{b}			
	**Cu-7**	**Cu-7^{2Me}**	**Cu-7^{Ph}**	**Cu-7^{CF3}**		
λ_{max} (nm)$^{c)}$ [76]	505	560	555	458		
	**Au-8^{ACD}**	**Au-8^{DPA}**	**Au-8^{DPAC}**	**Au-8^{DMAC}**		
λ_{max} (nm)$^{d)}$ [11]	533	663	666	705		

$^{a)}$ Measured in toluene solution. $^{b)}$ Measured in 2-MeTHF solution. $^{c)}$ Measured in 1 wt% polystyrene (PS) film. $^{d)}$ Measured in 5 wt% Zeonex film.

Table S22. Lowest excited states of CMA emitters calculated using optimized semi-coplanar structures in the S_0 state.

State	Nature	E_{vert}	State	Nature	E_{vert}
**Au-1**			**Au-1^{CN}**		
T_1	$^{3}LLCT_{cz\to cb}$	2.03	T_1	$^{3}LLCT_{cz\to cb}$	2.34
S_1	$^{1}LLCT_{cz\to cb}$	2.18	S_1	$^{1}LLCT_{cz\to cb}$	2.47
T_2	$^{3}LLCT_{cz\to cb}$	2.72	T_2	$^{3}LLCT_{cz\to cb}$ / $^{3}IL_{cz}$	2.99
S_2	$^{3}LLCT_{cz\to cb}$	2.75	S_2	$^{1}LLCT_{cz\to cb}$	3.07
T_3	$^{3}IL_{cz}$ / $^{3}LLCT_{cz\to cb}$	3.10	T_3	$^{3}LLCT_{cz\to cb}$ / $^{3}IL_{cz}$	3.10
**Au-1^{CN2}**			**Au-1^{CF3}**		
T_1	$^{3}LLCT_{cz\to cb}$ / $^{3}IL_{cz}$	2.64	T_1	$^{3}LLCT_{cz\to cb}$	2.26
S_1	$^{1}LLCT_{cz\to cb}$	2.76	S_1	$^{1}LLCT_{cz\to cb}$	2.39
T_2	$^{3}IL_{cz}$ / $^{3}LLCT_{cz\to cb}$	2.99	T_2	$^{3}LLCT_{cz\to cb}$	2.92
T_3	$^{3}LLCT_{cz\to cb}$ / $^{3}IL_{cz}$	3.12	S_2	$^{1}LLCT_{cz\to cb}$	2.95
T_4	$^{3}LLCT_{cz\to cb}$	3.39	T_3	$^{3}LLCT_{cz\to cb}$ / $^{3}IL_{cz}$	3.32
**Au-1^{2CF3}**			**Au-1^{2tBu}**		
T_1	$^{3}LLCT_{cz\to cb}$	2.49	T_1	$^{3}LLCT_{cz\to cb}$	1.92
S_1	$^{1}LLCT_{cz\to cb}$	2.61	S_1	$^{1}LLCT_{cz\to cb}$	2.08
T_2	$^{3}IL_{cz}$ / $^{3}LLCT_{cz\to cb}$	3.01	S_2	$^{1}LLCT_{cz\to cb}$	2.61
S_2	$^{1}LLCT_{cz\to cb}$	3.17	T_2	$^{3}IL_{cz}$ / $^{3}LLCT_{cz\to cb}$	3.10
T_3	$^{3}IL_{cz}$ / $^{3}LLCT_{cz\to cb}$	3.19	T_3	$^{3}IL_{cz}$ / $^{3}LLCT_{cz\to cb}$	3.19
**Au-1^{OMe}**			**Au-1^{2OMe}**		
T_1	$^{3}LLCT_{cz\to cb}$	1.85	T_1	$^{3}LLCT_{cz\to cb}$	1.73
S_1	$^{1}LLCT_{cz\to cb}$	2.00	S_1	$^{1}LLCT_{cz\to cb}$	1.90
T_2	$^{3}LLCT_{cz\to cb}$	2.62	T_2	$^{3}LLCT_{cz\to cb}$	2.46
S_2	$^{1}LLCT_{cz\to cb}$	2.66	S_2	$^{1}LLCT_{cz\to cb}$	2.48
T_3	$^{3}LLCT_{cz\to cb}$ / $^{3}IL_{cz}$	2.89	T_3	$^{3}LLCT_{cz\to cb}$ / $^{3}IL_{cz}$	2.78
**Ag-1**			**Cu-1**		
T_1	$^{3}LLCT_{cz\to cb}$	1.90	T_1	$^{3}LLCT_{cz\to cb}$	1.92
S_1	$^{1}LLCT_{cz\to cb}$	1.97	S_1	$^{1}LLCT_{cz\to cb}$	2.06
T_2	$^{3}LLCT_{cz\to cb}$	2.57	T_2	$^{3}LLCT_{cz\to cb}$	2.66
S_2	$^{1}LLCT_{cz\to cb}$	2.58	S_2	$^{1}LLCT_{cz\to cb}$	2.67
T_3	$^{3}IL_{cz}$ / $^{3}LLCT_{cz\to cb}$	3.03	T_3	$^{3}IL_{cz}$ / $^{3}LLCT_{cz\to cb}$	3.07
**Cu-1^{FLR}**			**Au-2**		
T_1	$^{3}LLCT_{cz\to cb}$	1.84	T_1	$^{3}LLCT_{cz\to cb}$	2.57
S_1	$^{1}LLCT_{cz\to cb}$	1.95	S_1	$^{1}LLCT_{cz\to cb}$	2.80
T_2	$^{3}LLCT_{cz\to cb}$	2.53	T_2	$^{3}IL_{cz}$ / $^{3}LLCT_{cz\to cb}$	3.11
S_2	$^{1}LLCT_{cz\to cb}$	2.56	T_3	$^{3}IL_{cz}$ / $^{3}LLCT_{cz\to cb}$	3.11
T_3	$^{3}LLCT_{cz\to cb}$ / $^{3}IL_{cz}$	2.75	T_4	$^{3}IL_{cz}$ / $^{3}LLCT_{cz\to cb}$	3.39

**Ag-2**			**Cu-2**		
T_1	$^3LLCT_{cz\rightarrow cb}$	2.57	T_2	$^3LLCT_{cz\rightarrow cb}$	2.48
S_1	$^1LLCT_{cz\rightarrow cb}$	2.68	S_1	$^1LLCT_{cz\rightarrow cb}$	2.71
T_2	$^3IL_{cz}$	3.04	T_2	$^3LLCT_{cz\rightarrow cb}$	2.92
T_3	$^3MLCT_{Ag\rightarrow cb}$ / $^3LLCT_{cz\rightarrow cb}$	3.07	T_3	$^3IL_{cz}$	3.07
T_4	$^3LLCT_{cz\rightarrow cb}$	3.12	T_4	$^3IL_{cz}$ / $^3LLCT_{cz\rightarrow cb}$	3.12
**Au-3**			**Ag-3**		
T_1	$^3LLCT_{cz\rightarrow cb}$	2.39	T_1	$^3LLCT_{cz\rightarrow cb}$	2.33
S_1	$^1LLCT_{cz\rightarrow cb}$	2.56	S_1	$^1LLCT_{cz\rightarrow cb}$	2.42
T_2	$^3IL_{cz}$ / $^3LLCT_{cz\rightarrow cb}$	3.05	T_2	$^3LLCT_{cz\rightarrow cb}$ / $^3IL_{cz}$	3.02
T_3	$^3IL_{cz}$ / $^3LLCT_{cz\rightarrow cb}$	3.11	T_3	$^3IL_{cz}$ / $^3LLCT_{cz\rightarrow cb}$	3.04
S_2	$^1LLCT_{cz\rightarrow cb}$	3.16	S_2	$^1LLCT_{cz\rightarrow cb}$	3.06
**Cu-3**					
T_1	$^3LLCT_{cz\rightarrow cb}$	2.29			
S_1	$^1LLCT_{cz\rightarrow cb}$	2.46			
T_2	$^3MLCT_{Cu\rightarrow cb}$	2.98			
T_3	$^3LLCT_{cz\rightarrow cb}$ / $^3IL_{cz}$	3.04			
T_4	$^3IL_{cz}$	3.07			

Table S23. Comparison between $S_1 \rightarrow S_0$ emission energies of CMA emitters **M-1**, **M-2**, and **M-3** (M=Ag, Au, or Cu) measured experimentally in the literature and calculated at the level of PBE0-D3(BJ)/6-31G*/PCM(toluene) using optimized semi-coplanar structures for the emitters.

	**Au-1**	**Ag-1**	**Cu-1**	**Au-2**	**Ag-2**	**Cu-2**	**Au-3**	**Ag-3**	**Cu-3**
Expt. (eV)	2.00[2]	1.83[a)]	1.99[1]	2.42[6]	2.36[7]	2.47[6]	2.26[7]	2.17[7]	2.29[7]
Calc. (eV)	2.17	1.93	2.06	2.08	2.03	2.08	2.32	2.37	2.27
Error (eV)	0.17	0.10	0.07	-0.34	-0.32	-0.39	0.05	0.20	-0.02
Mean error (eV)					~0.19				

[a)] Photophysical results are shown in Table S4.

S2.3.11. Topological analysis and buried volume calculations

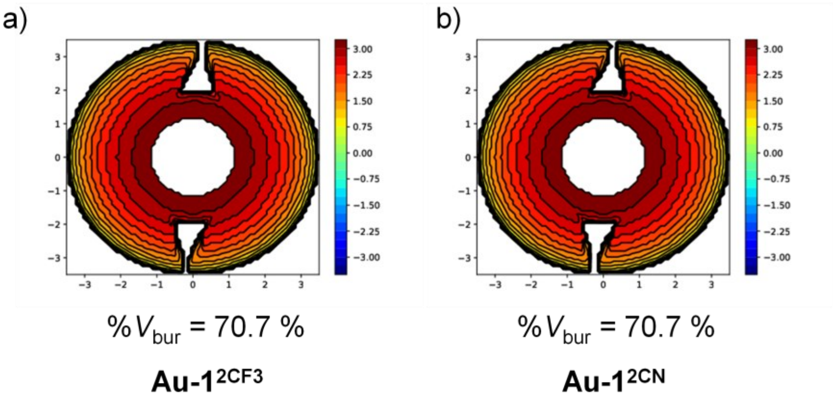

Figure S44. Calculated topological steric maps with buried volume (%V_{bur}) of a) **Au-1^{2CF3}** and b) **Au-1^{2CN}**.

S2.3.12. Coordinates

Optimized **Au-1** S_0 semi-coplanar:

Au	-0.70595000	-0.00176200	-0.00069300
N	2.07682900	-1.09107400	0.01553100
N	2.07297300	1.09606600	-0.01005000
N	4.49547200	-1.43067700	0.02170100
N	4.49036900	1.44435500	-0.01075100
N	-2.71846600	-0.00339400	-0.00222300
C	1.26685800	0.00106200	0.00166700
C	3.40095500	-0.69804900	0.01214700
C	3.39847400	0.70779800	-0.00355800
C	5.61157100	0.70984500	-0.00121600
H	6.55095500	1.25474300	-0.00631600
C	5.61405800	-0.69216300	0.01461300
H	6.55537000	-1.23370900	0.02170800
C	1.56590400	-2.42922300	0.03520500
C	1.32241500	-3.02530900	1.27876900
C	0.75772100	-4.30155000	1.27313700
H	0.54452600	-4.79478100	2.21725000
C	0.45231800	-4.94167400	0.07763000
H	0.00728000	-5.93257200	0.09447900
C	0.70714700	-4.31977200	-1.13909100
H	0.45638300	-4.82770700	-2.06603300
C	1.27031300	-3.04352600	-1.18803700
C	1.61436900	-2.31884700	2.58587300
H	2.09941000	-1.36285700	2.35866800
C	2.58473800	-3.12437300	3.45114400
H	2.15358600	-4.08805800	3.74483000
H	2.82109300	-2.57313200	4.36806500
H	3.51941800	-3.32072200	2.91531400
C	0.31593300	-2.00248100	3.33217400
H	-0.21645900	-2.92029500	3.60646400
H	-0.35472000	-1.39610100	2.71326100
H	0.53082400	-1.44821100	4.25294300
C	1.51577800	-2.36288300	-2.51817100
H	1.96668400	-1.38307000	-2.32462500
C	2.50563900	-3.15863600	-3.37102100
H	3.45450800	-3.30060600	-2.84289000
H	2.70770900	-2.63055900	-4.30958300
H	2.10802000	-4.14802000	-3.62414200
C	0.19892100	-2.11652200	-3.25790200
H	-0.30121900	-3.05975900	-3.50515000
H	0.38395400	-1.57766300	-4.19404500
H	-0.48820200	-1.51996700	-2.64765800
C	1.55728200	2.43232800	-0.03349900
C	1.31636000	3.02559600	-1.27892400
C	0.74730700	4.29991100	-1.27744600
H	0.53598500	4.79096400	-2.22312400
C	0.43511000	4.94080400	-0.08410000
H	-0.01330100	5.93012000	-0.10418900
C	0.68736800	4.32163500	1.13454600
H	0.43122400	4.83007400	2.05973500
C	1.25486100	3.04746100	1.18763400
C	1.61564100	2.31805900	-2.58378900
H	2.10150500	1.36328900	-2.35324300
C	0.32126900	1.99796600	-3.33550700
H	0.54149000	1.44274100	-4.25443700
H	-0.21180500	2.91421800	-3.61365800
H	-0.35086400	1.39111300	-2.71867800
C	2.58838200	3.12447300	-3.44555400
H	2.82969300	2.57286700	-4.36096600

Optimized **Au-1** S_1 semi-coplanar:

Au	-0.74894300	-0.10985700	-0.00643000
N	2.16352500	-0.92974600	0.08024300
N	1.93714300	1.21265700	-0.13872800
N	4.61245000	-1.07026300	0.11491500
N	4.30166400	1.86373100	-0.17818200
N	-2.82531500	-0.20161100	0.01714200
C	1.23973600	0.05524900	-0.02660300
C	3.44954900	-0.40640000	0.03729400
C	3.30388300	0.97090800	-0.10113200
C	5.49875100	1.19929100	-0.09842300
H	6.38822600	1.82224600	-0.14917200
C	5.64361900	-0.16969600	0.03675200
H	6.64386400	-0.59240900	0.08866700
C	1.80555000	-2.30297300	0.21603100
C	1.63003700	-2.82144000	1.50462700
C	1.22762400	-4.15392600	1.61324100
H	1.08320600	-4.59001000	2.59821900
C	1.01094600	-4.92789700	0.47869800
H	0.69985100	-5.96405400	0.58252400
C	1.19795000	-4.38518300	-0.78796900
H	1.03413500	-5.00202200	-1.66775600
C	1.60141400	-3.05773300	-0.94567700
C	1.86806800	-1.97960500	2.73990500
H	2.12801400	-0.96805200	2.41213700
C	3.05721600	-2.51648400	3.53854100
H	2.86743700	-3.53362000	3.90150400
H	3.25182300	-1.87893800	4.40856900
H	3.95770400	-2.53649800	2.91622900
C	0.60339000	-1.86878500	3.59240300
H	0.28670200	-2.84633400	3.97463600
H	-0.22292100	-1.44808200	3.00762400
H	0.78151000	-1.21453600	4.45367300
C	1.82213900	-2.47261800	-2.32420400
H	2.06541500	-1.41206400	-2.20448700
C	3.02008700	-3.13999000	-3.00305200
H	3.91951100	-3.02709300	-2.38901400
H	3.20883800	-2.68306300	-3.98126800
H	2.84339400	-4.21094700	-3.15864600
C	0.55770500	-2.55292800	-3.18017800
H	0.25461800	-3.59126900	-3.35921000
H	0.72866200	-2.08261600	-4.15532300
H	-0.27545300	-2.03335900	-2.69257100
C	1.29173100	2.48031100	-0.23908700
C	0.97185300	2.96887300	-1.51137300
C	0.27913600	4.17929900	-1.58155500
H	0.01343200	4.58742500	-2.55313800
C	-0.07399400	4.86669000	-0.42584100
H	-0.61097400	5.80871400	-0.49988300
C	0.26353800	4.35870400	0.82431200
H	-0.00934500	4.90910000	1.72089800
C	0.95468200	3.15107600	0.94379300
C	1.35190500	2.21862100	-2.76977000
H	1.86506100	1.29917000	-2.47039200
C	0.11229900	1.80866300	-3.56661800
H	0.40196700	1.22546900	-4.44840000
H	-0.45144700	2.68299400	-3.91284800
H	-0.55679200	1.19369200	-2.95341900
C	2.33442400	3.03305400	-3.61303000
H	2.63691400	2.46496700	-4.50008900

H	3.52036300	3.32285500	-2.90578200	H	3.23202900	3.27122200	-3.03324700
H	2.15688100	4.08719000	-3.74186600	H	1.88580100	3.97396400	-3.95300400
C	1.49760100	2.36969900	2.51971600	C	1.33399800	2.60522400	2.30397400
H	1.95355900	1.39160600	2.32931400	H	1.77122500	1.61242400	2.15756700
C	0.17867100	2.11875400	3.25422000	C	0.11205000	2.43159000	3.20624400
H	0.36206200	1.58213700	4.19197900	H	0.40829400	1.98820400	4.16375200
H	-0.50335700	1.51832300	2.64203800	H	-0.62641600	1.76991000	2.73869500
H	-0.32655800	3.06021100	3.49787200	H	-0.37546100	3.39000300	3.42018700
C	2.48037800	3.17086200	3.37569300	C	2.40742700	3.48329400	2.95091000
H	2.68089200	2.64480700	4.31572100	H	2.71179600	3.06497700	3.91719800
H	2.07732400	4.15876800	3.62599500	H	2.03653100	4.50062200	3.12411000
H	3.43079700	3.31642500	2.85133800	H	3.29067200	3.54481000	2.30688700
C	-3.53699300	-1.11202800	-0.04052100	C	-3.60534500	-1.30425000	-0.17327000
C	-3.17232900	-2.46249500	-0.08787900	C	-3.17936900	-2.61807600	-0.41243000
H	-2.12318400	-2.75241200	-0.09723700	H	-2.12001800	-2.85421100	-0.46132600
C	-4.18304300	-3.41339200	-0.12316300	C	-4.15790900	-3.59317000	-0.57600800
H	-3.91938100	-4.46763500	-0.16044500	H	-3.86392900	-4.62081500	-0.76312200
C	-5.53869500	-3.04181800	-0.11154000	C	-5.51600800	-3.26769900	-0.49934600
H	-6.30570000	-3.81105400	-0.14022500	H	-6.25866000	-4.04865700	-0.62880200
C	-5.90401700	-1.70240900	-0.06388400	C	-5.94350500	-1.95062900	-0.25498800
H	-6.95392500	-1.41826800	-0.05482100	H	-7.00403300	-1.72400100	-0.19734000
C	-4.90591900	-0.72495300	-0.02755000	C	-4.98942400	-0.96542400	-0.09123400
C	-4.90649700	0.71644500	0.02357700	C	-5.01891500	0.46570500	0.16925800
C	-5.90536600	1.69310700	0.06021500	C	-6.01198900	1.40872300	0.34905100
H	-6.95505100	1.40813800	0.05131700	H	-7.06256600	1.13559500	0.31359600
C	-5.54109300	3.03279900	0.10799800	C	-5.63815200	2.74478500	0.57956500
H	-6.30870200	3.80142300	0.13694600	H	-6.41220200	3.49242200	0.72126600
C	-4.18573200	3.40544000	0.11944600	C	-4.29458100	3.13051600	0.62549600
H	-3.92286400	4.45987900	0.15685000	H	-4.04117800	4.17111800	0.80027500
C	-3.17427500	2.45534900	0.08383100	C	-3.27724800	2.19903700	0.44569700
H	-2.12537600	2.74614400	0.09307200	H	-2.22912600	2.48495000	0.46919500
C	-3.53788800	1.10460300	0.03632700	C	-3.65024400	0.86595200	0.22187400
Optimized **Au-1** S_1 orthogonal:				Optimized **Au-1** T_1 semi-coplanar:			
Au	0.80959000	-0.03226100	0.00020500	Au	-0.75295000	0.22162800	-0.00808800
N	-2.03441700	-1.04800000	0.00027600	N	2.23551700	0.78411800	-0.00300500
N	-1.97323400	1.11889900	-0.00049600	N	1.78626400	-1.33712300	0.00119500
N	-4.45996700	-1.37488900	0.00000200	N	4.68141400	0.66779700	0.00478800
N	-4.37528100	1.58518700	-0.00114700	N	4.06408700	-2.22963200	0.00960800
N	2.87347100	-0.06090600	0.00027300	N	-2.80178800	0.39960000	-0.01313900
C	-1.17656000	0.01206600	0.00005200	C	1.20430100	-0.10409600	-0.00257500
C	-3.35113500	-0.62415600	-0.00011000	C	3.45502500	0.12989700	0.00106500
C	-3.31157100	0.77151100	-0.00064000	C	3.16491600	-1.23764900	0.00379500
C	-5.52226900	0.82646300	-0.00104900	C	5.32836400	-1.68992100	0.01242700
H	-6.45500900	1.38440000	-0.00143400	H	6.14571900	-2.40633900	0.01707200
C	-5.56161100	-0.55170500	-0.00051300	C	5.61565700	-0.34046100	0.01017100
H	-6.52477000	-1.05517600	-0.00047800	H	6.65404900	-0.01911100	0.01293000
C	-1.59730900	-2.40405300	0.00097600	C	2.02819400	2.19383800	0.00438900
C	-1.38770900	-3.03925700	-1.22949300	C	1.91947300	2.86000600	-1.22329800
C	-0.95886900	-4.36758500	-1.20423800	C	1.68521900	4.23580800	-1.18985800
H	-0.79417400	-4.89501000	-2.13992200	H	1.60386800	4.78814500	-2.12191500
C	-0.74762000	-5.02531300	0.00233800	C	1.56059900	4.90881500	0.02102200
H	-0.41811300	-6.06097400	0.00287300	H	1.38194200	5.98090900	0.02724600
C	-0.95932500	-4.36647900	1.20822600	C	1.67001900	4.21896600	1.22296500
H	-0.79498300	-4.89303900	2.14446000	H	1.57584100	4.75632200	2.16298000
C	-1.38816700	-3.03812300	1.23209900	C	1.90652600	2.84275400	1.23893100
C	-1.65213300	-2.32799400	-2.53939300	C	2.09120100	2.12376100	-2.53483000
H	-1.78388300	-1.26334600	-2.31941200	H	2.02258900	1.05121900	-2.32475800
C	-2.95672500	-2.83376200	-3.16084100	C	3.48656500	2.39147600	-3.10582200
H	-2.88660000	-3.90149100	-3.40174700	H	3.61671700	3.45632500	-3.33437600
H	-3.17691000	-2.29005900	-4.08707400	H	3.63763000	1.82379500	-4.03147000
H	-3.79195500	-2.69231300	-2.46727000	H	4.25797300	2.09714800	-2.38662700
C	-0.47564500	-2.44490000	-3.50771400	C	0.98828900	2.45424600	-3.53888600
H	-0.30776700	-3.48032300	-3.82581000	H	1.01440400	3.50563700	-3.84770600
H	0.44874900	-2.07946400	-3.04557800	H	-0.00154100	2.24691200	-3.11601400

H	-0.66900500	-1.85094900	-4.40847900	H	1.10835900	1.84431400	-4.44140900
C	-1.65308000	-2.32567900	2.54126200	C	2.04576100	2.09372500	2.54697600
H	-1.78505300	-1.26127300	2.32024100	H	2.13025000	1.02721400	2.31535800
C	-2.95771000	-2.83118600	3.16285100	C	3.33233600	2.50788500	3.26441100
H	-3.79276300	-2.69064900	2.46887800	H	4.20202700	2.32679200	2.62441300
H	-3.17832400	-2.28660500	4.08846600	H	3.45684600	1.93337300	4.18958400
H	-2.88737700	-3.89865100	3.40485800	H	3.31471800	3.57248300	3.52697100
C	-0.47677900	-2.44135600	3.50995100	C	0.81149900	2.26580600	3.43246500
H	-0.30856000	-3.47645900	3.82890500	H	0.66550100	3.31127200	3.72849100
H	-0.67057300	-1.84675800	4.41019600	H	0.91634900	1.67230400	4.34801100
H	0.44757200	-2.07591800	3.04772700	H	-0.09133700	1.92979100	2.90922900
C	-1.45870500	2.44704000	-0.00112700	C	1.01626900	-2.53620700	0.00606800
C	-1.21229400	3.06887700	1.22935900	C	0.62392700	-3.07361100	1.23884500
C	-0.70271500	4.36833300	1.20418300	C	-0.17274900	-4.22050800	1.21880600
H	-0.50709900	4.88500800	2.13993800	H	-0.49121000	-4.66642600	2.15729100
C	-0.45030200	5.01146700	-0.00238900	C	-0.55870600	-4.79986300	0.01471700
H	-0.05778700	6.02492000	-0.00288400	H	-1.17464000	-5.69550600	0.01820400
C	-0.70215200	4.36693100	-1.20832300	C	-0.15505200	-4.24204700	-1.19368200
H	-0.50609600	4.88249600	-2.14460200	H	-0.45985900	-4.70449200	-2.12870700
C	-1.21172600	3.06744500	-1.23221200	C	0.64178200	-3.09543200	-1.22235600
C	-1.52103700	2.37590400	2.53937200	C	1.05614200	-2.45082200	2.54878300
H	-1.73166100	1.32428200	2.31850100	H	1.57065300	-1.51238900	2.31890300
C	-0.33344900	2.40524400	3.50079100	C	-0.14113800	-2.10408000	3.43381500
H	-0.56495200	1.82792500	4.40340200	H	0.19696700	-1.60409100	4.34867500
H	-0.08693300	3.42566900	3.81620700	H	-0.70067500	-2.99870700	3.73126500
H	0.55814800	1.97152100	3.03331400	H	-0.82880300	-1.42965700	2.91027000
C	-2.78195500	2.97370100	3.16892000	C	2.05899600	-3.35824200	3.26468700
H	-3.03698300	2.44440200	4.09453100	H	2.40638900	-2.88780700	4.19173000
H	-3.62834100	2.89553300	2.47897200	H	2.92775100	-3.54737500	2.62576300
H	-2.63351800	4.03271400	3.41267600	H	1.60609500	-4.32289600	3.52350200
C	-1.51968100	2.37297300	-2.54161200	C	1.09098300	-2.49421700	-2.53653300
H	-1.73193800	1.32194900	-2.31951600	H	1.60560400	-1.55382400	-2.31508500
C	-0.33070600	2.39946100	-3.50141500	C	-0.09579100	-2.15837300	-3.43981200
H	-0.56171400	1.82136200	-4.40365700	H	0.25346200	-1.67495000	-4.35937100
H	0.55960000	1.96506000	-3.03210700	H	-0.78670100	-1.47323100	-2.93467100
H	-0.08230800	3.41917300	-3.81765900	H	-0.65522700	-3.05608200	-3.72800300
C	-2.77899700	2.97140200	-3.17373100	C	2.09985100	-3.41456400	-3.22689600
H	-3.03350900	2.44098800	-4.09884200	H	2.45814400	-2.95948200	-4.15743600
H	-2.62897000	4.02985100	-3.41894400	H	1.64829400	-4.38258900	-3.47529000
H	-3.62636200	2.89534700	-2.48475700	H	2.96113100	-3.59421600	-2.57529600
C	3.68350600	-0.06251300	-1.10460500	C	-3.55935200	1.53889800	-0.01620500
C	3.28786500	-0.06250900	-2.44683800	C	-3.10137300	2.86134500	-0.01622100
H	2.23465200	-0.06265400	-2.70869200	H	-2.03661700	3.07563400	-0.01381300
C	4.28655100	-0.06245800	-3.41576900	C	-4.05694100	3.87318000	-0.01924900
H	4.01476400	-0.06223900	-4.46597400	H	-3.73912900	4.91049500	-0.01930600
C	5.63569500	-0.06248700	-3.05182300	C	-5.42146400	3.56972900	-0.02228000
H	6.39595000	-0.06213700	-3.82630500	H	-6.14601000	4.37788300	-0.02463000
C	6.03409300	-0.06282700	-1.70280000	C	-5.88060000	2.24033000	-0.02234400
H	7.08910100	-0.06253400	-1.44652000	H	-6.94605200	2.03132200	-0.02471600
C	5.05708200	-0.06299100	-0.72827700	C	-4.94865300	1.22214500	-0.01931300
C	5.05706900	-0.06201300	0.72888900	C	-5.01291300	-0.23409000	-0.01792000
C	6.03408100	-0.06057900	1.70341500	C	-6.02985100	-1.16770800	-0.01936900
H	7.08908800	-0.06061600	1.44713400	H	-7.07330100	-0.86711400	-0.02246400
C	5.63567700	-0.05843800	3.05243600	C	-5.68769100	-2.53243100	-0.01673400
H	6.39592700	-0.05709200	3.82692100	H	-6.48001700	-3.27429600	-0.01786600
C	4.28654000	-0.05785100	3.41636000	C	-4.35483000	-2.95265800	-0.01263700
H	4.01473000	-0.05619300	4.46655800	H	-4.12443900	-4.01291700	-0.01054700
C	3.28784900	-0.05913900	2.44740800	C	-3.31465700	-2.02795900	-0.01117000
H	2.23463400	-0.05879700	2.70926700	H	-2.27445500	-2.34088300	-0.00780300
C	3.68350800	-0.06101300	1.10520500	C	-3.65758300	-0.67048400	-0.01397800
Optimized **Ag-1** S_0 semi-coplanar:				Optimized **Ag-1** S_1 semi-coplanar:			
C	-1.23129500	-0.06907900	0.00444600	C	-1.17030100	-0.10361500	-0.01670500
N	2.84477300	0.27032800	-0.02595600	N	2.94685300	0.58830200	-0.04616600
C	3.63414500	1.39476700	-0.06485200	C	3.71943400	1.71336100	-0.03849300

N	-2.17925000	0.90190900	0.03568200
Ag	0.79895700	0.15480400	-0.01977700
N	-1.88862300	-1.25819200	0.00772100
N	-4.62351000	0.92328700	0.08837100
N	-4.24051900	-1.92652200	0.05479200
C	-3.44156800	0.34050600	0.05733900
C	-3.25425200	-1.05190900	0.04082400
C	-5.44687100	-1.34635200	0.08672300
H	-6.30693500	-2.00938100	0.10012300
C	-5.63388700	0.04471200	0.10290500
H	-6.63837800	0.45676800	0.12849000
C	-1.85319400	2.29660800	0.04125500
C	-1.66758600	2.92784000	1.27741800
C	-1.28776800	4.27070900	1.25586900
H	-1.12676600	4.79490000	2.19366900
C	-1.10367900	4.94114000	0.05222700
H	-0.80343200	5.98516300	0.05650800
C	-1.29693400	4.28327100	-1.15691200
H	-1.14451800	4.81771400	-2.09040700
C	-1.67592700	2.94023700	-1.18988900
C	-1.83862200	2.19720400	2.59271800
H	-2.17554300	1.17715200	2.37722500
C	-2.91487300	2.85623300	3.45725200
H	-2.63309900	3.87783100	3.73601600
H	-3.05956600	2.28668800	4.38201500
H	-3.87141700	2.90175300	2.92597600
C	-0.50404400	2.08650000	3.33387000
H	-0.11404000	3.07551200	3.59999100
H	0.24964600	1.58325400	2.71801100
H	-0.62902200	1.51315400	4.25944900
C	-1.86420600	2.22714100	-2.51250800
H	-2.15777000	1.19181300	-2.30627000
C	-2.99213900	2.86574900	-3.32548600
H	-3.93097600	2.86576000	-2.76153300
H	-3.14864000	2.31213100	-4.25802800
H	-2.75536500	3.90290000	-3.58847100
C	-0.55430400	2.17687200	-3.30196700
H	-0.20928300	3.18273700	-3.56665700
H	-0.69204400	1.61297700	-4.23140600
H	0.23837100	1.69190100	-2.72158700
C	-1.20125400	-2.51515900	-0.02787000
C	-0.91772000	-3.07816200	-1.27893100
C	-0.18499200	-4.26590800	-1.29176200
H	0.06532400	-4.72842100	-2.24217700
C	0.23913800	-4.85613000	-0.10687000
H	0.81366800	-5.77758700	-0.13839600
C	-0.06041000	-4.27058000	1.11747900
H	0.28418000	-4.73774200	2.03565200
C	-0.78777800	-3.08084000	1.18497400
C	-1.34292000	-2.42250500	-2.57617100
H	-1.96173500	-1.55117800	-2.33407300
C	-0.12403100	-1.91355400	-3.34983400
H	-0.43989300	-1.40035100	-4.26528000
H	0.53670800	-2.73992400	-3.63536800
H	0.46139600	-1.21084900	-2.74651100
C	-2.19987000	-3.36282000	-3.42497400
H	-2.54025100	-2.84924400	-4.33093700
H	-3.08005900	-3.70202200	-2.86872300
H	-1.63465600	-4.24772800	-3.73811200
C	-1.08507100	-2.43891900	2.52381900
H	-1.64837000	-1.51647100	2.34461800
C	0.20653200	-2.04758800	3.24516500
H	-0.02414900	-1.53353800	4.18508000
H	0.81694600	-1.37821600	2.62895900
H	0.81342000	-2.92830900	3.48328700

N	-2.27590700	0.68545800	-0.00662000
Ag	0.82810600	0.38611300	-0.03551400
N	-1.64474700	-1.37913600	0.00922400
N	-4.70803100	0.36198800	0.05118900
N	-3.83964700	-2.47341700	0.06888100
C	-3.43813600	-0.07164100	0.02783900
C	-3.03087900	-1.40337100	0.03694100
C	-5.14408200	-2.04433300	0.09224000
H	-5.89743700	-2.82757000	0.11930600
C	-5.54822000	-0.72346600	0.08434800
H	-6.61095900	-0.49579200	0.10606800
C	-2.20058500	2.10792300	-0.00896200
C	-2.10075100	2.77312300	1.22006400
C	-1.99596100	4.16521200	1.19383300
H	-1.92403900	4.71398100	2.12916100
C	-1.99237300	4.85733300	-0.01218300
H	-1.91528600	5.94143900	-0.01339600
C	-2.09620600	4.16974500	-1.21614700
H	-2.10147800	4.72198500	-2.15219700
C	-2.20228800	2.77755400	-1.23900800
C	-2.14239900	2.02140900	2.53360800
H	-2.09334800	0.95132500	2.30856700
C	-3.47236700	2.27811200	3.24657300
H	-3.58340900	3.33833600	3.50416000
H	-3.52910700	1.69605500	4.17372800
H	-4.31165500	1.99144100	2.60463200
C	-0.94293300	2.34973200	3.42227300
H	-0.93123000	3.40474700	3.71971000
H	-0.00094200	2.13252500	2.90491700
H	-0.97487800	1.74778300	4.33766000
C	-2.34559800	2.03057000	-2.54786000
H	-2.28704100	0.95933300	-2.33004400
C	-3.72231300	2.29731100	-3.16115200
H	-4.51344800	2.00837200	-2.46186000
H	-3.84812800	1.72198400	-4.08569400
H	-3.84730100	3.35966600	-3.40310800
C	-1.21194600	2.35718700	-3.51993100
H	-1.21659700	3.41375500	-3.81196700
H	-1.31368300	1.76057300	-4.43366800
H	-0.23600500	2.13336300	-3.07328300
C	-0.78129800	-2.51276800	0.02938900
C	-0.38328800	-3.07701600	-1.19042600
C	0.48773500	-4.16788200	-1.14463300
H	0.81069600	-4.63365400	-2.07162600
C	0.94112900	-4.66870900	0.07118500
H	1.61416100	-5.52215400	0.08771800
C	0.53371700	-4.08493500	1.26558100
H	0.89372600	-4.48397100	2.21015200
C	-0.33863500	-2.99407500	1.26886800
C	-0.90024700	-2.54781000	-2.51087800
H	-1.40618800	-1.59721900	-2.31299100
C	0.23025300	-2.26091400	-3.49859000
H	-0.17407700	-1.81454900	-4.41409200
H	0.76537900	-3.17353400	-3.78537800
H	0.95832100	-1.56162200	-3.07082700
C	-1.94021500	-3.50940700	-3.09207400
H	-2.35388100	-3.10808100	-4.02450200
H	-2.76112000	-3.65917700	-2.38339200
H	-1.49186300	-4.48598200	-3.31171700
C	-0.79204300	-2.37122200	2.57198900
H	-1.41095100	-1.50152300	2.33048700
C	0.39343400	-1.86974600	3.39756800
H	0.03932700	-1.37815000	4.31095900
H	0.98703700	-1.14438800	2.82850300
H	1.05650000	-2.69042800	3.69549600

C	-1.96264900	-3.34789800	3.38673000
H	-2.20728900	-2.85257200	4.33299800
H	-1.44995300	-4.28688800	3.62419000
H	-2.89813500	-3.59404900	2.87317400
C	3.23415300	2.73662100	-0.11693300
H	2.17649000	2.99665100	-0.12930600
C	4.21488600	3.71785500	-0.15331800
H	3.92046300	4.76395900	-0.19458800
C	5.58189900	3.38763300	-0.13795800
H	6.32533800	4.17977200	-0.16782400
C	5.98632900	2.05988200	-0.08494600
H	7.04461500	1.80755200	-0.07286300
C	5.01827400	1.05235100	-0.04743100
C	5.05986500	-0.38667900	0.00800900
C	6.08385500	-1.33740300	0.05029000
H	7.12609000	-1.02519400	0.04351900
C	5.75569000	-2.68618800	0.10096700
H	6.54328500	-3.43433600	0.13437900
C	4.40962600	-3.09384400	0.10961000
H	4.17360400	-4.15485900	0.14926900
C	3.37460400	-2.17055300	0.06831600
H	2.33479800	-2.49398700	0.07529700
C	3.69815500	-0.80786900	0.01805100

C	-1.66755900	-3.34858400	3.35893700
H	-2.03120200	-2.87868700	4.27993000
H	-1.10768300	-4.24936500	3.63757100
H	-2.53270100	-3.65356600	2.76150100
C	3.27963300	3.04314700	-0.02777200
H	2.21784100	3.27291300	-0.02474700
C	4.24471100	4.04571700	-0.02170800
H	3.93729900	5.08629600	-0.01352500
C	5.60658100	3.72926700	-0.02610800
H	6.33911200	4.53021000	-0.02131900
C	6.05104400	2.39541900	-0.03658300
H	7.11457400	2.17580200	-0.03957700
C	5.10903900	1.38574900	-0.04267800
C	5.15608800	-0.06928900	-0.05321300
C	6.15940600	-1.01773500	-0.05957600
H	7.20727300	-0.73219500	-0.05797700
C	5.79915000	-2.37783400	-0.06818100
H	6.58130900	-3.13042800	-0.07308700
C	4.46000000	-2.77943100	-0.07027000
H	4.21393600	-3.83625300	-0.07654800
C	3.43352100	-1.84068600	-0.06385300
H	2.39079000	-2.14552200	-0.06484600
C	3.79036600	-0.48469000	-0.05512800

Optimized **Ag-1** S_1 orthogonal:

C	-1.13265840	-0.01023003	0.00366676
N	3.02488446	0.07704607	0.00222155
C	3.83820107	0.08137370	-1.09809427
N	-1.92970970	-1.11884481	0.00117460
Ag	0.91235850	0.04393824	0.00237979
N	-2.00463640	1.04020141	0.00505697
N	-4.33067345	-1.60548588	-0.00283276
N	-4.43402530	1.35702380	0.00290085
C	-3.27131198	-0.78338563	0.00032498
C	-3.31982360	0.61129695	0.00294575
C	-5.53005984	0.52526433	-0.00011066
H	-6.49693621	1.02176257	-0.00039030
C	-5.48214861	-0.85223121	-0.00277731
H	-6.41194066	-1.41522667	-0.00510274
C	-1.40290421	-2.44152840	-0.00550658
C	-1.14576714	-3.05354767	-1.23937770
C	-0.61533481	-4.34501090	-1.22207728
H	-0.41199581	-4.85333092	-2.16072233
C	-0.35310692	-4.99149828	-0.01948210
H	0.05547711	-5.99859396	-0.02491142
C	-0.61719667	-4.35884821	1.19017173
H	-0.41516757	-4.87815635	2.12299137
C	-1.14772829	-3.06774800	1.22174623
C	-1.47372519	-2.36034529	-2.54478392
H	-1.67623907	-1.30793809	-2.32008412
C	-2.74905483	-2.95464162	-3.14877694
H	-2.60735828	-4.01402307	-3.39525508
H	-3.02136768	-2.42459609	-4.06907475
H	-3.58086188	-2.87444886	-2.44159932
C	-0.30709739	-2.39542402	-3.53116646
H	-0.07378917	-3.41637607	-3.85473114
H	0.59862224	-1.96862765	-3.08439712
H	-0.55451038	-1.81487452	-4.42739895
C	-1.48114374	-2.38984687	2.53381017
H	-1.66474865	-1.33097928	2.32285306
C	-2.77347170	-2.97453023	3.11090870
H	-3.59376528	-2.87301040	2.39304521
H	-3.05156883	-2.45406837	4.03496493
H	-2.64921829	-4.03919275	3.34380651
C	-0.32948423	-2.45787116	3.53576483

Optimized **Ag-1** T_1 semi-coplanar:

C	-1.15808071	-0.10291870	0.01745869
N	2.92973128	0.58299128	0.04988815
C	3.69953412	1.71281045	0.04441953
N	-2.26713982	0.68833429	0.00446031
Ag	0.82944698	0.37675326	0.03891124
N	-1.64162273	-1.37950762	-0.00974360
N	-4.69674376	0.37168968	-0.05834169
N	-3.83704443	-2.46564779	-0.07357133
C	-3.42900179	-0.06364224	-0.03240609
C	-3.02461767	-1.40001659	-0.04049038
C	-5.14253519	-2.03252088	-0.09965562
H	-5.89709803	-2.81448047	-0.12793845
C	-5.54209561	-0.71282280	-0.09287934
H	-6.60371677	-0.48054473	-0.11671132
C	-2.18691387	2.10987937	0.00634859
C	-2.18886453	2.78068182	1.23586858
C	-2.07875664	4.17261978	1.21272001
H	-2.08538423	4.72546058	2.14841683
C	-1.96989109	4.85934820	0.00868918
H	-1.89032078	5.94332182	0.00950133
C	-1.97177760	4.16646648	-1.19693854
H	-1.89601339	4.71457954	-2.13236045
C	-2.08044129	2.77462224	-1.22251441
C	-2.33954609	2.03391713	2.54399953
H	-2.26796488	0.96326266	2.32724355
C	-3.72602956	2.28851226	3.14040451
H	-3.86351677	3.35023899	3.37862277
H	-3.85747757	1.71371835	4.06456152
H	-4.50583354	1.99076671	2.43214447
C	-1.22110707	2.37150127	3.52963599
H	-1.23990959	3.42750110	3.82324914
H	-0.23772749	2.15776280	3.09444831
H	-1.32775179	1.77281803	4.44147613
C	-2.12194325	2.02130866	-2.53513021
H	-2.06084018	0.95222355	-2.30835932
C	-3.45867141	2.26512643	-3.24009861
H	-4.29134067	1.97207140	-2.59246462
H	-3.51618403	1.68146058	-4.16625622
H	-3.58018727	3.32418515	-3.49804761
C	-0.93140648	2.35915864	-3.43194740

H	-0.11807189	-3.48665685	3.84934635
H	-0.58058423	-1.88548861	4.43617084
H	0.58985808	-2.04039840	3.10846309
C	-1.57518559	2.39783807	0.00519630
C	-1.36716481	3.03484634	1.23557055
C	-0.94019708	4.36400126	1.21103422
H	-0.77791069	4.89182659	2.14693159
C	-0.72903883	5.02260835	0.00493630
H	-0.40158195	6.05893964	0.00485576
C	-0.93925266	4.36350131	-1.20105362
H	-0.77625288	4.89089545	-2.13708222
C	-1.36621141	3.03435424	-1.22532183
C	-1.63539196	2.32424707	2.54526137
H	-1.75548088	1.25812383	2.32579493
C	-0.46997720	2.45475729	3.52528089
H	-0.66703418	1.86128897	4.42552157
H	-0.31507453	3.49234013	3.84288127
H	0.46399923	2.09742497	3.07556771
C	-2.95014242	2.81965601	3.15383516
H	-3.17390293	2.27592967	4.07923186
H	-3.77753663	2.66917219	2.45290334
H	-2.89134577	3.88852296	3.39294282
C	-1.63307328	2.32325961	-2.53502651
H	-1.75541979	1.25755009	-2.31507736
C	-0.46523602	2.45081770	-3.51254484
H	-0.66109744	1.85656389	-4.41254377
H	0.46703737	2.09246544	-3.06012645
H	-0.30784947	3.48780506	-3.83088473
C	-2.94568926	2.82025039	-3.14682507
H	-3.16817373	2.27626003	-4.07237407
H	-2.88493635	3.88888540	-3.38645378
H	-3.77482123	2.67126223	-2.44762925
C	3.44217257	0.08476879	-2.44049128
H	2.38890206	0.08538062	-2.70386076
C	4.43888157	0.08681611	-3.41172410
H	4.16572718	0.08908624	-4.46156390
C	5.78852213	0.08581933	-3.04982457
H	6.54733944	0.08721018	-3.82568451
C	6.18953021	0.08288776	-1.70139060
H	7.24511764	0.08199243	-1.44731867
C	5.21411235	0.08082716	-0.72556374
C	5.21346517	0.07623726	0.73196143
C	6.18797713	0.07274157	1.70868094
H	7.24379835	0.07368464	1.45560133
C	5.78570638	0.06717130	3.05673869
H	6.54381470	0.06425573	3.83328816
C	4.43573561	0.06500690	3.41740755
H	4.16164693	0.06044771	4.46699593
C	3.43989809	0.06828519	2.44527235
H	2.38633358	0.06637411	2.70751788
C	3.83723272	0.07402930	1.10325323

H	-0.93181544	3.41310485	-3.73347241
H	-0.96294856	1.75381747	-4.34513849
H	0.01567373	2.15262151	-2.91949969
C	-0.78026451	-2.51380193	-0.02864142
C	-0.33186527	-2.99314357	-1.26695374
C	0.53949157	-4.08495794	-1.26226404
H	0.90212345	-4.48317860	-2.20621445
C	0.94182882	-4.67119365	-0.06727845
H	1.61366345	-5.52566889	-0.08264393
C	0.48436021	-4.17164466	1.14759664
H	0.80305898	-4.63934166	2.07513294
C	-0.38629893	-3.08027245	1.19154725
C	-0.78090311	-2.36811951	-2.57052160
H	-1.39007237	-1.49169239	-2.32875981
C	0.40744456	-1.88020255	-3.39988649
H	0.05578647	-1.38554661	-4.31258911
H	1.06103209	-2.70785897	-3.69963808
H	1.00976607	-1.16033943	-2.83294390
C	-1.66753099	-3.33864182	-3.35362009
H	-2.02846094	-2.86743154	-4.27505063
H	-2.53438869	-3.63392240	-2.75384276
H	-1.11665053	-4.24542071	-3.63099382
C	-0.90925743	-2.55279232	2.51029179
H	-1.40902489	-1.59924836	2.31117224
C	0.21557797	-2.27401001	3.50660257
H	-0.19320602	-1.82894270	4.42077985
H	0.94892373	-1.57583669	3.08594537
H	0.74515791	-3.18962764	3.79432184
C	-1.95755363	-3.51151394	3.08123568
H	-2.37611257	-3.11116747	4.01193637
H	-1.51498672	-4.49075235	3.30102507
H	-2.77401444	-3.65520377	2.36620675
C	3.25268576	3.03917087	0.03635315
H	2.19004087	3.26390387	0.03369001
C	4.21360604	4.04622496	0.03209013
H	3.90199206	5.08537910	0.02597598
C	5.57605069	3.73485320	0.03557951
H	6.30526932	4.53872173	0.03218502
C	6.02709841	2.40263320	0.04322190
H	7.09137022	2.18756739	0.04546464
C	5.08885110	1.39059662	0.04750645
C	5.14246166	-0.06606554	0.05482612
C	6.15099320	-1.00799310	0.05913894
H	7.19723573	-0.71717799	0.05806395
C	5.79657848	-2.37005476	0.06486782
H	6.58239024	-3.11873922	0.06814209
C	4.46027042	-2.77840998	0.06627769
H	4.21918474	-3.83620999	0.07037544
C	3.42901309	-1.84398453	0.06210858
H	2.38784086	-2.15349884	0.06291309
C	3.78086873	-0.48822111	0.05614752

Optimized **Ag-1** T_1 orthogonal:

C	-1.13255182	0.01004594	-0.00106291
N	3.02435113	-0.07593769	-0.00085577
C	3.83724394	-0.07590468	-1.10153699
N	-2.00415252	-1.04087064	-0.00116380
Ag	0.91223732	-0.04334990	-0.00093244
N	-1.93030687	1.11828745	-0.00016571
N	-4.43333547	-1.35869755	0.00003941
N	-4.33138615	1.60364206	0.00156384
C	-3.31946815	-0.61263776	-0.00023666
C	-3.27164326	0.78219032	0.00043903
C	-5.48255147	0.84995879	0.00184799

Optimized **Cu-1** S_0 semi-coplanar:

Cu	0.72597600	0.05481900	-0.32510000
N	2.56552600	0.10469400	-0.42264200
N	-1.83952600	-1.15118000	0.15385700
N	-1.94232500	1.02874500	0.18230400
N	-4.16458500	-1.61634500	0.74651200
N	-4.29793800	1.25573900	0.79426400
C	3.40677700	-0.98511400	-0.35430800
C	3.06521900	-2.34335700	-0.35984700
H	2.02423500	-2.65368700	-0.42816100
C	4.08606100	-3.27931900	-0.27397500
H	3.83717400	-4.33800900	-0.27540500

H	-6.41257408	1.41257148	0.00272744	C	5.77802300	-1.54054000	-0.17980100
C	-5.52981827	-0.52755327	0.00113365	H	6.82139800	-1.24066100	-0.10973500
H	-6.49642328	-1.02457074	0.00144445	C	4.76791400	-0.57778300	-0.26202400
C	-1.57390190	-2.39824610	-0.00112020	C	4.74297700	0.86382200	-0.27075900
C	-1.36500940	-3.03499256	-1.23150423	C	5.71979300	1.86092300	-0.19880500
C	-0.93717121	-4.36386047	-1.20697374	H	6.77263400	1.59720000	-0.12447600
H	-0.77422051	-4.89147163	-2.14287755	C	3.97023600	3.54023000	-0.31331700
C	-0.72597823	-5.02244262	-0.00086685	H	3.68643800	4.58997100	-0.32656600
H	-0.39782331	-6.05855339	-0.00077456	C	2.98140100	2.56940700	-0.38887600
C	-0.93699733	-4.36358704	1.20512077	H	1.93014700	2.84325100	-0.45890700
H	-0.77389123	-4.89096269	2.14113539	C	3.36831600	1.22338100	-0.36805000
C	-1.36482913	-3.03471688	1.22939358	C	-1.10410400	-0.02160600	-0.02850100
C	-1.63322650	-2.32441241	-2.54121133	C	-3.14065500	-0.82896300	0.48594100
H	-1.75395567	-1.25839260	-2.32166036	C	-3.20599500	0.57581400	0.50857800
C	-2.94748648	-2.82047517	-3.15029626	C	-5.28137900	-0.93558900	1.03845800
H	-2.88798053	-3.88925522	-3.38961629	H	-6.16568600	-1.52426600	1.26421900
H	-3.17128984	-2.27667991	-4.07564260	C	-5.34618400	0.46511900	1.06263400
H	-3.77519384	-2.67064786	-2.44959280	H	-6.28054800	0.96128700	1.30829200
C	-0.46741274	-2.45403319	-3.52086100	C	-1.26768900	-2.45996200	0.04015200
H	-0.31192953	-3.49141433	-3.83884194	C	-1.26322200	-3.07512700	-1.21818000
H	0.46621875	-2.09647158	-3.07062154	C	-0.64677900	-4.32361300	-1.31630500
H	-0.66438311	-1.86023974	-4.42091187	H	-0.61557700	-4.82926400	-2.27725000
C	-1.63260731	-2.32384855	2.53903266	C	-0.06184400	-4.92041500	-0.20565500
H	-1.75450249	-1.25804167	2.31918493	H	0.41854500	-5.88984600	-0.30371000
C	-2.94587360	-2.82077716	3.14952823	C	-0.07768200	-4.27892700	1.02710900
H	-3.77434580	-2.67173783	2.44956332	H	0.39706600	-4.74852800	1.88380900
H	-3.16920992	-2.27686481	4.07491782	C	-0.68078600	-3.02910300	1.17808800
H	-2.88535520	-3.88943733	3.38912039	C	-1.85850000	-2.41385300	-2.44313300
C	-0.46574451	-2.45197463	3.51763765	H	-2.31839000	-1.46882300	-2.13379100
H	-0.30895529	-3.48908983	3.83585177	C	-0.76296400	-2.07566200	-3.45732900
H	-0.66238819	-1.85797992	4.41763151	H	-0.27290100	-2.98297100	-3.82836500
H	0.46709104	-2.09370774	3.06631290	H	0.00755200	-1.43928100	-3.00780700
C	-1.40439195	2.44129829	0.00117050	H	-1.18814300	-1.54529800	-4.31683100
C	-1.14772361	3.05887694	1.23240674	C	-2.95996200	-3.27439800	-3.06398700
C	-0.61835401	4.35066228	1.20940074	H	-3.40903400	-2.75645700	-3.91873900
H	-0.41534046	4.86327920	2.14575405	H	-3.74937600	-3.48742800	-2.33549900
C	-0.35676496	4.99219617	0.00396077	H	-2.56512900	-4.23081300	-3.42485300
H	0.05093538	5.99966348	0.00504687	C	-0.65184400	-2.32248200	2.51696300
C	-0.62032544	4.35407853	-1.20288353	H	-1.21223200	-1.38564200	2.42287100
H	-0.41880085	4.86938821	-2.13806571	C	-1.33991800	-3.15357500	3.60111900
C	-1.14970220	3.06235682	-1.22874036	H	-2.37325600	-3.38567800	3.32230400
C	-1.47602746	2.37107369	2.54059428	H	-1.35248900	-2.60320600	4.54844000
H	-1.67199419	1.31629737	2.32097144	H	-0.81466800	-4.09969600	3.77357300
C	-0.31335143	2.41793686	3.53109496	C	0.78317300	-1.95828900	2.90690000
H	-0.56087029	1.84036613	4.42920877	H	1.25768200	-1.33110100	2.14387100
H	-0.08739064	3.44176480	3.85074473	H	1.40110900	-2.85479700	3.02994400
H	0.59667145	1.99435145	3.09006152	H	0.79108100	-1.40929200	3.85539800
C	-2.75671794	2.96190592	3.13672914	C	-1.49479800	2.38803400	0.10702300
H	-3.02971863	2.43532245	4.05881757	C	-1.67828700	3.07840000	-1.09903200
H	-3.58547230	2.87393806	2.42688600	C	-2.32708900	2.41998800	-2.29919700
H	-2.62109909	4.02325929	3.37813374	C	-1.24842400	1.90637900	-3.25863100
C	-1.48061937	2.37825684	-2.53821214	C	-3.31648600	3.33929200	-3.01342500
H	-1.67265878	1.32209945	-2.32161133	H	-2.89487900	1.55173600	-1.94581900
C	-0.32198103	2.43207670	-3.53306021	H	-0.56546300	1.21294600	-2.75503900
H	-0.57132838	1.85676031	-4.43210345	H	-1.70432700	1.38359700	-4.10737300
H	0.59114986	2.00987133	-3.09716674	H	-0.65049400	2.73762400	-3.64983300
H	-0.10053480	3.45763252	-3.85030649	H	-4.07424200	3.72012700	-2.32090900
C	-2.76529510	2.96756817	-3.12731838	H	-2.81538000	4.19637200	-3.47658000
H	-3.04043375	2.44340759	-4.05015625	H	-3.82568900	2.78970300	-3.81250000
H	-2.63336053	4.03009995	-3.36559052	C	-0.83769900	2.93447200	1.21705000
H	-3.59101622	2.87498079	-2.41452295	C	-0.64434700	2.17117500	2.51087900
C	3.44054680	-0.07396410	-2.44372618	C	-1.44924400	2.81504400	3.64237500
H	2.38708941	-0.07313772	-2.70644832	C	0.83730100	2.04432800	2.87115500
C	4.43682661	-0.07285672	-3.41541702	H	-1.03071600	1.15549400	2.37262100
H	4.16321794	-0.07113672	-4.46514091	H	-2.51343600	2.86734800	3.38833000

C	5.78662815	-0.07366855	-3.05412078
H	6.54509303	-0.07250266	-3.83032859
C	6.18826066	-0.07571703	-1.70587814
H	7.24396377	-0.07590070	-1.45229397
C	5.21328179	-0.07693747	-0.72960066
C	5.21326855	-0.07773067	0.72794644
C	6.18825516	-0.07748929	1.70422160
H	7.24395525	-0.07734090	1.45062487
C	5.78663550	-0.07690050	3.05246557
H	6.54510070	-0.07651486	3.82867380
C	4.43683734	-0.07657423	3.41375296
H	4.16321218	-0.07599480	4.46347412
C	3.44055775	-0.07673434	2.44205609
H	2.38711834	-0.07624478	2.70483636
C	3.83722999	-0.07716056	1.09986922

H	-1.34144000	2.23328100	4.56471100
H	-1.10008500	3.83387300	3.84568400
H	1.40485200	1.56183300	2.06787200
H	1.29103500	3.02390900	3.05790400
H	0.95318300	1.44325700	3.77990800
C	-1.17225900	4.37614500	-1.17416800
C	-0.51693300	4.95042600	-0.09013300
C	-0.35346200	4.23855600	1.09123800
H	0.16551400	4.69688600	1.92814800
H	-0.12614700	5.96094200	-0.16988000
H	-1.28260900	4.94187900	-2.09423600
C	5.43336000	-2.88636600	-0.18547800
H	6.20983600	-3.64398100	-0.12089900
C	5.32988100	3.19418300	-0.21995800
H	6.08011800	3.97840600	-0.16364900

Optimized **Cu-1** S_1 semi-coplanar:

Cu	-0.80989500	-0.13385400	-0.09298600
N	-2.70467400	-0.21828000	-0.09814600
N	1.75919900	1.19056500	0.10648900
N	1.99749200	-0.95156600	-0.04719700
N	4.11246000	1.84021700	0.35224900
N	4.43790800	-1.09852200	0.15947300
C	-3.53634400	0.85624400	-0.23361900
C	-3.16416600	2.19912900	-0.38770400
H	-2.11731500	2.48985700	-0.41014900
C	-4.18178400	3.14031100	-0.50534800
H	-3.92751700	4.18847900	-0.62522200
C	-5.89901700	1.40732600	-0.30619300
H	-6.94959800	1.13367500	-0.27466600
C	-4.90503900	0.45542100	-0.18843900
C	-4.87283200	-0.98876700	-0.00767100
C	-5.82485100	-1.98185300	0.11784600
H	-6.88619700	-1.75345800	0.08710500
C	-4.03460500	-3.63649800	0.33067600
H	-3.73714400	-4.67185500	0.46156700
C	-3.05859800	-2.65280000	0.20464000
H	-1.99929700	-2.89464500	0.23237000
C	-3.48769800	-1.32896300	0.03842400
C	1.06099400	0.03301700	-0.03857600
C	3.12153000	0.94974600	0.19814900
C	3.27511200	-0.43311000	0.10520600
C	5.30985100	1.17234000	0.40599000
H	6.19208400	1.79554800	0.52887800
C	5.46118600	-0.19879500	0.31804300
H	6.45945500	-0.62546500	0.37594500
C	1.09564100	2.45073800	0.16924300
C	0.81776800	3.12240400	-1.03040500
C	0.11206200	4.32453000	-0.94691800
H	-0.11668500	4.87550700	-1.85454300
C	-0.30111400	4.82616100	0.28371000
H	-0.84904900	5.76375900	0.32865200
C	-0.01440800	4.13563900	1.45511800
H	-0.34335400	4.53561600	2.41061700
C	0.69207000	2.93099200	1.42114600
C	1.30182900	2.58025800	-2.35864800
H	1.50768300	1.51288600	-2.22311600
C	0.25501100	2.69836000	-3.46455100
H	0.05071400	3.74230700	-3.72901400
H	-0.69120400	2.23113000	-3.16766900
H	0.61256600	2.19719400	-4.37083200
C	2.61848400	3.25977700	-2.74845100
H	3.00704100	2.83530200	-3.68158700
H	3.37138100	3.12671900	-1.96453900
H	2.46749100	4.33559300	-2.90016000

Optimized **Cu-1** S_1 orthogonal:

Cu	0.89795845	-0.00043807	0.04421940
N	2.78030843	-0.00078821	0.06395714
N	-1.80820492	1.08208083	-0.05994205
N	-1.80894304	-1.08132880	-0.05956313
N	-4.22110516	1.47994961	-0.17188211
N	-4.22211667	-1.47758967	-0.17132142
C	3.59476282	-0.00052279	1.16658746
C	3.19913855	-0.00015724	2.50853961
H	2.14629994	-0.00002142	2.77291263
C	4.19680613	0.00001983	3.47886040
H	3.92422186	0.00029221	4.52887466
C	5.94560808	-0.00046771	1.76766639
H	7.00084916	-0.00054320	1.51215054
C	4.96889972	-0.00066733	0.79235101
C	4.96893057	-0.00102559	-0.66529657
C	5.94538799	-0.00130025	-1.64106770
H	7.00071898	-0.00128671	-1.38591237
C	4.19615063	-0.00168057	-3.35171527
H	3.92298794	-0.00193494	-4.40158656
C	3.19878841	-0.00142364	-2.38097750
H	2.14586914	-0.00147781	-2.64490361
C	3.59505187	-0.00108679	-1.03913888
C	-0.97208808	0.00010177	-0.01774718
C	-3.13389339	0.70004095	-0.12264705
C	-3.13436764	-0.69840661	-0.12238516
C	-5.34580921	0.69071591	-0.22494086
H	-6.29225839	1.22311049	-0.26990089
C	-5.34627918	-0.68760444	-0.22468149
H	-6.29309105	-1.21936917	-0.26944815
C	-1.32031212	2.41963916	-0.01418951
C	-1.16916308	3.03329156	1.23608577
C	-0.67623674	4.33964610	1.26031306
H	-0.55494223	4.84868521	2.21265457
C	-0.34419711	4.99736824	0.08199764
H	0.03610001	6.01481716	0.11914384
C	-0.49857424	4.36151661	-1.14505363
H	-0.23815319	4.88890930	-2.05823791
C	-0.99013688	3.05707619	-1.21860606
C	-1.56331590	2.32474316	2.51464564
H	-1.71868726	1.26785623	2.27421894
C	-0.46600347	2.38915056	3.57638033
H	-0.28843046	3.41436511	3.92111986
H	0.48110349	1.99638559	3.18844412
H	-0.75284740	1.79344181	4.45060442
C	-2.89212272	2.87922925	3.03520311
H	-3.20924872	2.33668076	3.93360040
H	-3.67276187	2.77977224	2.27422563
H	-2.79690706	3.94073708	3.29456512

C	0.99929500	2.17814000	2.69798500	C	-1.21284789	2.37174272	-2.55028302
H	1.53743700	1.26445500	2.42749700	H	-1.27719005	1.29476687	-2.35869396
C	1.92219600	2.99568100	3.60329300	C	-2.55160490	2.81767265	-3.14718585
H	2.85200400	3.24068300	3.07995800	H	-3.37191473	2.62388599	-2.44838298
H	2.17354200	2.42588400	4.50510700	H	-2.75347414	2.28107564	-4.08184618
H	1.44771500	3.93261000	3.91834400	H	-2.53541007	3.89217211	-3.36743325
C	-0.28339800	1.75519200	3.41588800	C	-0.06157017	2.58897018	-3.53056086
H	-0.90978500	1.13577500	2.76296400	H	0.89912397	2.30097529	-3.08815837
H	-0.87616800	2.62282200	3.72876800	H	0.01511737	3.63489096	-3.84923129
H	-0.04399300	1.17147700	4.31224500	H	-0.21996254	1.98617967	-4.43211091
C	1.62509100	-2.32388700	-0.15332000	C	-1.32199499	-2.41921357	-0.01344011
C	1.57101600	-2.90585500	-1.42645500	C	-1.17115619	-3.03257394	1.23700061
C	1.93816000	-2.11620900	-2.66574800	C	-1.56462921	-2.32331863	2.51538330
C	0.67364700	-1.63860900	-3.38610100	C	-0.46704542	-2.38788766	3.57683220
C	2.85470700	-2.89921200	-3.60456800	C	-2.89357260	-2.87692472	3.03650314
H	2.49014000	-1.22782200	-2.34212600	H	-1.71952764	-1.26645348	2.27457900
H	0.05340700	-1.02562500	-2.72129800	H	0.48014712	-1.99576972	3.18845984
H	0.93382100	-1.03432600	-4.26326900	H	-0.75330584	-1.79170630	4.45092575
H	0.07023000	-2.48958400	-3.72488000	H	-0.28991001	-3.41307456	3.92187294
H	3.75386400	-3.23773900	-3.08003000	H	-3.67436995	-2.77730438	2.27570480
H	2.35392500	-3.77717700	-4.02874600	H	-2.79886311	-3.93840131	3.29617970
H	3.16496000	-2.26297500	-4.44082300	H	-3.21016425	-2.33391819	3.93481098
C	1.26144000	-3.00880200	1.01428700	C	-0.99240048	-3.05726598	-1.21768791
C	1.37997900	-2.36235600	2.37856900	C	-1.21480178	-2.37218572	-2.54954413
C	2.63694900	-2.87212200	3.08930400	C	-2.55397806	-2.81734835	-3.14611378
C	0.12512900	-2.55462600	3.22869600	C	-0.06383612	-2.59053938	-3.52992794
H	1.50294200	-1.28500600	2.22761300	H	-1.27833730	-1.29510619	-2.35826339
H	3.52738500	-2.67453000	2.48343300	H	-3.37402357	-2.62291966	-2.44718142
H	2.75824800	-2.37423700	4.05839000	H	-2.75567434	-2.28079577	-4.08083618
H	2.57494900	-3.95266600	3.26673800	H	-2.53850299	-3.89189807	-3.36616154
H	-0.76506500	-2.18071000	2.70933700	H	0.89714240	-2.30311406	-3.08776627
H	-0.04530300	-3.60814000	3.47877300	H	0.01203061	-3.63661396	-3.84829559
H	0.22333300	-2.00535700	4.17177900	H	-0.22192831	-1.98790788	-4.43163582
C	1.11852900	-4.22369400	-1.51503400	C	-0.67912659	-4.33926416	1.26160268
C	0.74632000	-4.92749800	-0.37510100	C	-0.34767824	-4.99759729	0.08346455
C	0.82203700	-4.32769100	0.87725200	C	-0.50174477	-4.36202232	-1.14377597
H	0.53994700	-4.89270100	1.76139200	H	-0.24181393	-4.88989480	-2.05682198
H	0.39994600	-5.95392500	-0.46286900	H	0.03191370	-6.01529908	0.12089086
H	1.05444500	-4.70440700	-2.48710000	H	-0.55807310	-4.84806753	2.21410235
C	-5.52521600	2.75355700	-0.46653600	C	5.54619509	-0.00012932	3.11587832
H	-6.29959100	3.50868300	-0.55875500	H	6.30564631	0.00002503	3.89118332
C	-5.39349200	-3.30899100	0.28811400	C	5.54564385	-0.00162287	-2.98913827
H	-6.13399300	-4.09649400	0.38730300	H	6.30487694	-0.00183654	-3.76466039
Optimized **Cu-1** T_1 semi-coplanar:				Optimized **Cu-1** T_1 orthogonal:			
Cu	0.82247981	0.10436914	-0.12780205	Cu	0.89855400	-0.01992100	0.02963300
N	2.68997209	0.17308032	-0.14877766	N	2.77885800	-0.03686200	0.03969000
N	-1.77035329	-1.17547154	0.05370866	N	-1.79180300	1.09759700	-0.07426400
N	-1.95279207	0.98091776	0.04296157	N	-1.82694100	-1.06392800	0.02170000
N	-4.13070585	-1.77187441	0.31278393	N	-4.19922400	1.53053600	-0.15739500
N	-4.37809159	1.17676456	0.30167113	N	-4.24820300	-1.42235700	-0.02005200
C	3.52277196	-0.91555837	-0.17342330	C	3.59639300	0.08292600	1.13417200
C	3.14551848	-2.26293332	-0.20871415	C	3.20456200	0.22976500	2.46915400
H	2.09802190	-2.55135492	-0.22116512	H	2.15256200	0.25773400	2.73499200
C	4.16125087	-3.21448450	-0.22333051	C	4.20502200	0.33797800	3.43064900
H	3.90412017	-4.26830246	-0.24909614	H	3.93534400	0.45354400	4.47505600
C	5.88200388	-1.47482955	-0.16845699	C	5.94907000	0.15069500	1.72531300
H	6.93296265	-1.20194834	-0.15332476	H	7.00336700	0.12215600	1.46734100
C	4.88904463	-0.51507775	-0.15251394	C	4.96933700	0.04166400	0.75886100
C	4.86127804	0.94246242	-0.11102997	C	4.96598700	-0.11903200	-0.68963100
C	5.81795232	1.93785274	-0.07527515	C	5.94138800	-0.22998200	-1.66025800
H	6.87822413	1.70363003	-0.07609522	H	6.99675400	-0.20257300	-1.40671600
C	4.03392456	3.61233945	-0.03000733	C	4.18972800	-0.41700800	-3.35747500
H	3.73979288	4.65617640	0.00347295	H	3.91480800	-0.53335100	-4.40044200
C	3.05415329	2.62412219	-0.06585387	C	3.19363800	-0.30635000	-2.39166700

H	1.99581407	2.87102374	-0.05810593
C	3.48058338	1.29192560	-0.10872136
C	-1.03479995	-0.02710376	-0.03789344
C	-3.11742811	-0.90607611	0.19344430
C	-3.23537210	0.49036317	0.18832377
C	-5.31195610	-1.07761538	0.42741211
H	-6.20704180	-1.68591038	0.52868311
C	-5.42687049	0.29651630	0.42296224
H	-6.41092554	0.74756617	0.52188621
C	-1.13589868	-2.45107962	0.04773610
C	-0.91118828	-3.08511345	-1.18287872
C	-0.23304269	-4.30570714	-1.16581888
H	-0.04605027	-4.82789948	-2.09967890
C	0.20347436	-4.86324021	0.03194333
H	0.72608324	-5.81632240	0.02556494
C	-0.02823093	-4.20888519	1.23633143
H	0.31664090	-4.65368428	2.16596513
C	-0.70148555	-2.98518080	1.26800926
C	-1.42465657	-2.48441637	-2.47402607
H	-1.61369638	-1.42148620	-2.28896636
C	-0.40893795	-2.56907142	-3.61153000
H	-0.21955124	-3.60396428	-3.91920652
H	0.54814604	-2.11922724	-3.32275650
H	-0.78640909	-2.03253399	-4.48912120
C	-2.75900557	-3.13404543	-2.85408484
H	-3.16832450	-2.66786966	-3.75802516
H	-3.48806205	-3.02429268	-2.04442205
H	-2.62486414	-4.20437251	-3.05339095
C	-0.96470741	-2.27859343	2.58059559
H	-1.37879211	-1.29177984	2.35122475
C	-2.01739737	-3.03864543	3.39076475
H	-2.94488282	-3.13452199	2.81698234
H	-2.24010821	-2.50726557	4.32324185
H	-1.66679975	-4.04514685	3.64921992
C	0.32179095	-2.05491839	3.37549722
H	1.05273023	-1.48845981	2.78646319
H	0.78746812	-3.00135980	3.67400763
H	0.10812473	-1.48797755	4.28882512
C	-1.54660013	2.34504886	-0.00672997
C	-1.51498609	2.98869692	-1.24987797
C	-1.96552650	2.29034028	-2.51503434
C	-0.79514950	2.09713575	-3.48120161
C	-3.13257775	3.03162032	-3.16832538
H	-2.32772235	1.29585181	-2.23731057
H	0.00746246	1.51951203	-3.00754504
H	-1.12284997	1.55552623	-4.37614317
H	-0.37766932	3.05851959	-3.80359517
H	-3.97153675	3.11540464	-2.47020147
H	-2.84412210	4.04186318	-3.48197805
H	-3.47683030	2.49048991	-4.05699118
C	-1.12506946	2.96189517	1.17959385
C	-1.20811418	2.24869215	2.51247830
C	-2.39264840	2.78550564	3.32011701
C	0.10185673	2.32537409	3.29545985
H	-1.40090780	1.19015207	2.31167546
H	-3.32433152	2.66810079	2.75688896
H	-2.48896758	2.24263623	4.26760449
H	-2.26172393	3.84999283	3.54942272
H	0.93613524	1.92715326	2.70587592
H	0.34976260	3.35387357	3.58241895
H	0.02490751	1.73609024	4.21622546
C	-1.03493807	4.29964041	-1.28849349
C	-0.60807168	4.93661333	-0.12885081
C	-0.65632722	4.27467433	1.09371002
H	-0.32877017	4.78589569	1.99489833

H	2.14047900	-0.33272500	-2.65341900
C	3.59150500	-0.15866900	-1.05865000
C	-0.97175700	0.00423600	-0.00317900
C	-3.12402700	0.73573200	-0.09306400
C	-3.14706200	-0.66145400	-0.02860600
C	-5.33761300	0.75974100	-0.15028000
H	-6.27593300	1.30601800	-0.19986400
C	-5.36036900	-0.61688200	-0.08606500
H	-6.31629700	-1.13389600	-0.08706500
C	-1.27906600	2.42531200	-0.13477400
C	-1.03158600	3.10526800	1.06587800
C	-0.51445500	4.39915900	0.98094400
H	-0.31686300	4.95839800	1.89102800
C	-0.25356100	4.98301200	-0.25370200
H	0.14622300	5.99256300	-0.30045000
C	-0.50419900	4.28337500	-1.42824100
H	-0.30022000	4.75255500	-2.38677700
C	-1.02109800	2.98673700	-1.39246100
C	-1.36438400	2.47651600	2.40213900
H	-1.44752500	1.39555200	2.24521800
C	-0.27464100	2.69807200	3.44935300
H	-0.18643400	3.75269500	3.73469100
H	0.70327800	2.36677600	3.08152500
H	-0.50978300	2.13366600	4.35894700
C	-2.72586300	2.98142100	2.88978600
H	-3.00715300	2.48535900	3.82629100
H	-3.50189900	2.78492900	2.14286600
H	-2.69253600	4.06245300	3.07306700
C	-1.33634800	2.23485600	-2.66802400
H	-1.50013400	1.18513600	-2.40202100
C	-2.63766700	2.76412300	-3.27805800
H	-3.45773300	2.68883200	-2.55669000
H	-2.90467900	2.18926200	-4.17268100
H	-2.53000700	3.81629600	-3.56884700
C	-0.18372300	2.27332300	-3.67023500
H	0.74544600	1.90573600	-3.21936900
H	0.00216300	3.28693700	-4.04365400
H	-0.41850500	1.64387200	-4.53631900
C	-1.36032200	-2.40622800	0.12057200
C	-1.21959200	-2.97274100	1.39355100
C	-1.59897300	-2.20887600	2.64427100
C	-0.47775400	-2.21035900	3.68273500
C	-2.90917100	-2.75390700	3.21879400
H	-1.77434000	-1.16681100	2.35795800
H	0.45205700	-1.81447200	3.25772100
H	-0.75686200	-1.58680100	4.53995200
H	-0.27200100	-3.21821900	4.06148000
H	-3.70604300	-2.69878300	2.47020700
H	-2.79637200	-3.80059300	3.52668100
H	-3.21469300	-2.17265900	4.09673800
C	-1.04031300	-3.09567700	-1.05801000
C	-1.25809000	-2.45922200	-2.41468200
C	-2.62783800	-2.86822100	-2.96597800
C	-0.14043800	-2.77187100	-3.40777400
H	-1.26882800	-1.37293800	-2.26931400
H	-3.42433600	-2.61709700	-2.25789600
H	-2.82844400	-2.35710100	-3.91511700
H	-2.66087100	-3.94960600	-3.14717100
H	0.84366800	-2.52268500	-2.99423400
H	-0.12698300	-3.83031300	-3.69223100
H	-0.28504000	-2.19197800	-4.32646500
C	-0.74607000	-4.28441200	1.46985000
C	-0.42252600	-4.99227600	0.31884400
C	-0.56707400	-4.40269700	-0.93263700
H	-0.31324900	-4.96930900	-1.82378600

H	-0.24044282	5.95834913	-0.17650723
H	-0.99431057	4.82635460	-2.23839922
C	5.50459985	-2.82892964	-0.20423457
H	6.27715662	-3.59130401	-0.21641391
C	5.39074610	3.27657072	-0.03555117
H	6.13424405	4.06689279	-0.00740951

H	-0.05683700	-6.01281800	0.39600400
H	-0.63271100	-4.75685700	2.44190600
C	5.55356300	0.29934400	3.06626900
H	6.31503800	0.38551000	3.83476300
C	5.53995500	-0.37967800	-2.99932800
H	6.29783500	-0.46785300	-3.77113200

Optimized **Au-2** S_0 semi-coplanar:

Au	-0.46855304	0.54496003	0.04846001
N	2.31575518	-0.22021193	-0.01008099
N	-2.46456817	0.19671493	0.02173101
C	1.50371409	0.81770712	0.02814501
C	2.24488410	2.13472824	0.00208101
C	3.24807517	2.10528127	1.18481610
H	3.81842017	3.04007736	1.17664610
H	2.68942013	2.08470625	2.12452117
C	4.17831128	0.89098822	1.09454309
H	5.22230334	1.19296128	0.95797408
H	4.14096530	0.28608817	2.00571016
C	3.78438328	0.03600614	-0.11559300
C	1.79731319	-1.56533204	0.11033702
C	1.52426820	-2.30948711	-1.04941606
C	1.06723621	-3.61916922	-0.89084105
H	0.84011622	-4.21101027	-1.77292712
C	0.87903922	-4.16811026	0.37050004
H	0.52365323	-5.18952135	0.47271505
C	1.11570021	-3.39754020	1.50060112
H	0.93164321	-3.81882124	2.48486919
C	1.57120119	-2.08129009	1.39846811
C	1.75097418	-1.26677702	2.66363320
H	2.07625717	-0.26197794	2.38169218
C	2.82180628	-1.86696403	3.57674827
H	2.53540529	-2.86706311	3.92104329
H	2.96271126	-1.23785798	4.46283133
H	3.78682635	-1.95658200	3.06507923
C	0.41810208	-1.10865706	3.40015025
H	-0.33489399	-0.65198305	2.74838321
H	0.54660607	-0.46995801	4.28158332
H	0.02957708	-2.07530314	3.73935528
C	1.62606118	-1.72565106	-2.44255616
H	2.07160118	-0.73191498	-2.35849816
C	0.22895208	-1.54327510	-3.04331121
H	-0.28256392	-2.50609919	-3.15313821
H	0.29823307	-1.07979806	-4.03429328
H	-0.39286999	-0.90568907	-2.40509516
C	2.51520028	-2.55820509	-3.36723023
H	3.52198736	-2.69135206	-2.95632820
H	2.60801427	-2.06632005	-4.34181630
H	2.09246829	-3.55397617	-3.54062924
C	4.58672038	-1.24769592	-0.18197700
H	4.43993039	-1.86509197	0.70883106
H	5.64833344	-0.98910387	-0.24753701
H	4.32803739	-1.84634397	-1.05911006
C	3.98249626	0.90346121	-1.36487208
H	3.78445827	0.30517916	-2.26059415
H	5.03940833	1.18818527	-1.40841109
C	3.07150415	2.14751027	-1.32367609
H	3.70463917	3.04113436	-1.27631608
C	2.20354309	2.23548024	-2.57756518
H	1.50044607	1.39701016	-2.63731018
H	2.83572613	2.20763726	-3.47219924
H	1.61649201	3.15736929	-2.61443818
C	1.28017698	3.34233329	0.11052902
H	0.48611693	3.16422125	-0.62812903
C	1.93858498	4.68310841	-0.21722400

Optimized **Au-2** S_1 orthogonal:

Au	-0.49945152	-0.34732300	-0.35778131
N	2.35337283	0.13905894	0.26876795
N	-2.57189532	-0.12303808	-0.19700914
C	1.46986445	-0.56861722	-0.54469348
C	2.20258411	-1.62859556	-1.34465721
C	3.33984889	-0.92969708	-2.12813553
H	3.88219365	-1.66456718	-2.73672904
H	2.89363024	-0.20858455	-2.81997937
C	4.30250730	-0.21259482	-1.16910002
H	5.29781696	-0.67402913	-1.18460596
H	4.44067943	0.83700140	-1.44944281
C	3.76017675	-0.30227117	0.26469917
C	1.90964578	1.27814468	0.99630247
C	1.47261162	1.13894490	2.32941198
C	1.11053299	2.28599104	3.04077534
H	0.78964268	2.19095646	4.07551295
C	1.14570324	3.54084994	2.44784505
H	0.86867875	4.42330473	3.01897717
C	1.52014277	3.65993683	1.11542021
H	1.52511083	4.64029370	0.64449899
C	1.89491965	2.54026270	0.36963281
C	2.25567321	2.71039615	-1.09088525
H	2.47356454	1.71697942	-1.48867496
C	3.48956896	3.59466933	-1.27860182
H	3.30882918	4.61390395	-0.91672798
H	3.75789103	3.66052364	-2.33978472
H	4.35405464	3.19872142	-0.73448214
C	1.06688934	3.25403652	-1.88495979
H	0.20572246	2.58857133	-1.76705217
H	1.31328887	3.31555156	-2.95185865
H	0.77582034	4.25594261	-1.54734556
C	1.33175729	-0.21528371	2.99383152
H	1.59328969	-0.96830085	2.24595137
C	-0.12199056	-0.46551701	3.40365950
H	-0.44188181	0.22150794	4.19646785
H	-0.24220018	-1.48903563	3.77986211
H	-0.78914031	-0.33120541	2.54631257
C	2.25481478	-0.38222354	4.20239128
H	3.30942025	-0.26844900	3.93253040
H	2.12695456	-1.37710614	4.64542547
H	2.02873161	0.35898382	4.97833571
C	4.62264847	0.51256782	1.21109387
H	4.61854711	1.57396980	0.94438764
H	5.65435485	0.14952366	1.15507427
H	4.28580337	0.42688691	2.24724834
C	3.76044226	-1.78846394	0.64268579
H	3.38399404	-1.89098529	1.66739233
H	4.79475040	-2.15430722	0.64874842
C	2.88987106	-2.60970095	-0.33874457
H	3.55277567	-3.26405536	-0.92153778
C	1.90832262	-3.48671185	0.43123351
H	1.16780221	-2.85810544	0.94093180
H	2.43754509	-4.08106740	1.18594018
H	1.36393703	-4.18025202	-0.21780346
C	1.25268221	-2.37498440	-2.31389310
H	0.39569724	-2.69717767	-1.70359887
C	1.86643784	-3.61893500	-2.95842356

H	2.77450103	4.89818345	0.45929604
H	1.20712790	5.48864246	-0.09098500
H	2.31216601	4.74067943	-1.24366508
C	0.60662893	3.44869628	1.48081512
H	0.19942593	2.48904119	1.81885314
H	-0.22640715	4.15810830	1.42997712
H	1.30277897	3.81666733	2.24359117
C	-3.49112828	1.11487996	-0.00461399
C	-3.41354033	2.51179207	0.02586801
H	-2.44566828	3.00608913	0.07826902
C	-4.59520943	3.23910107	-0.01168699
H	-4.55302147	4.32539416	0.01162301
C	-5.84544049	2.60015999	-0.07870099
H	-6.75243658	3.19800600	-0.10692400
C	-5.92871245	1.21390189	-0.10816300
H	-6.89747052	0.72175181	-0.15938000
C	-4.75332135	0.45914487	-0.07086799
C	-4.46161928	-0.95253422	-0.08476599
C	-5.23906829	-2.11278833	-0.14133900
H	-6.32392340	-2.04735736	-0.18764000
C	-4.60934520	-3.35108440	-0.13718800
H	-5.20328722	-4.26017148	-0.18048100
C	-3.20746410	-3.43948935	-0.07571199
H	-2.73282003	-4.41809741	-0.07161899
C	-2.41329308	-2.30292625	-0.01872999
H	-1.32971800	-2.37476621	0.02989701
C	-3.04395717	-1.05369318	-0.02539999

H	2.74789746	-3.36528061	-3.56011466
H	1.13859548	-4.08394549	-3.63383814
H	2.16787932	-4.37695606	-2.22939081
C	0.69878442	-1.47684446	-3.42252144
H	0.33693148	-0.52505797	-3.02045995
H	-0.13614251	-1.97499474	-3.92942309
H	1.46103384	-1.26215581	-4.18172367
C	-3.40152852	-0.87267099	0.59773686
C	-3.04423484	-1.94827993	1.41785103
H	-2.01061932	-2.27401335	1.46804889
C	-4.05319584	-2.57121789	2.14752291
H	-3.80843576	-3.40751209	2.79422157
C	-5.37742316	-2.13517002	2.05605256
H	-6.14562321	-2.63804566	2.63525942
C	-5.73977564	-1.06003303	1.22581197
H	-6.77611049	-0.74079756	1.16630362
C	-4.75177753	-0.43059438	0.49584968
C	-4.72191469	0.67555837	-0.45083522
C	-5.66956017	1.52535817	-0.98438238
H	-6.71655186	1.45006294	-0.70580217
C	-5.25132935	2.50189728	-1.90537056
H	-5.98751595	3.17619740	-2.33175000
C	-3.91114663	2.62230251	-2.28181326
H	-3.62178280	3.38675336	-2.99549539
C	-2.94234359	1.77508817	-1.75139486
H	-1.89661796	1.85170134	-2.02811477
C	-3.35586190	0.80323943	-0.83460926

Optimized **Au-2** T_1 semi-coplanar:

Au	-0.46212600	0.61281100	-0.04016800
N	2.31396100	-0.31336700	-0.01378800
N	-2.51061100	0.26023300	-0.05214900
C	1.51132800	0.82358100	0.07160500
C	2.34967100	2.08352400	0.08044300
C	3.34195300	1.98079600	1.26458100
H	3.97987700	2.87371100	1.29442500
H	2.77210400	1.96302600	2.19841000
C	4.19995500	0.70968000	1.15002900
H	5.26396200	0.95500200	1.04509700
H	4.10905100	0.08393500	2.04398600
C	3.76997600	-0.09424300	-0.08594900
C	1.73444100	-1.60767800	0.10820300
C	1.42904600	-2.35023000	-1.05162400
C	0.92570900	-3.64696600	-0.90849800
H	0.70237100	-4.23149300	-1.79781400
C	0.69719100	-4.19422900	0.34722400
H	0.31825100	-5.20888800	0.44164400
C	0.93532900	-3.42831500	1.48247000
H	0.72819500	-3.84601400	2.46480600
C	1.44119900	-2.12963200	1.38522200
C	1.64222600	-1.31860900	2.64784900
H	1.98347100	-0.32577500	2.34495300
C	2.69673000	-1.94349300	3.56301000
H	2.38165700	-2.93178300	3.91876100
H	2.86497100	-1.31093200	4.44264900
H	3.65469300	-2.06762500	3.04580700
C	0.31662700	-1.12454800	3.38723500
H	-0.41521500	-0.63928900	2.73140400
H	0.46180300	-0.48929100	4.26932800
H	-0.10280800	-2.07919000	3.72698100
C	1.56971500	-1.76252700	-2.44017700
H	2.00230000	-0.76608700	-2.32489100
C	0.19299200	-1.58622200	-3.08809100
H	-0.31503900	-2.55004600	-3.21650400

Optimized **Ag-2** S_0 semi-coplanar:

Ag	-0.52260606	0.62825495	0.06054399
N	2.29784309	-0.21115128	-0.00475002
N	-2.55741322	0.30635304	0.03288698
C	1.53068710	0.85376584	0.02874298
C	2.32530323	2.13548389	-0.01011902
C	3.32269930	2.07915883	1.17699606
H	3.93267340	2.98856086	1.16103007
H	2.76200826	2.09193786	2.11580313
C	4.20062529	0.82478169	1.10099706
H	5.25732138	1.08110664	0.96900305
H	4.13249225	0.22864865	2.01621312
C	3.77911721	-0.02219335	-0.10624203
C	1.72214797	-1.53258834	0.12491499
C	1.42243591	-2.27318337	-1.03057710
C	0.90694179	-3.56002744	-0.86469908
H	0.65744975	-4.14807147	-1.74319215
C	0.68887975	-4.09017946	0.39985001
H	0.28785866	-5.09386151	0.50801302
C	0.95225281	-3.32151542	1.52516209
H	0.74402078	-3.72664844	2.51134616
C	1.46583993	-2.02717736	1.41591008
C	1.67575699	-1.21266731	2.67665118
H	2.04649508	-0.22509026	2.39024216
C	2.71490803	-1.85381242	3.59850324
H	2.38236594	-2.83741947	3.94862726
H	2.88077608	-1.22517039	4.48054630
H	3.67652809	-1.99042349	3.09101420
C	0.34851891	-0.99166622	3.40730423
H	-0.38447912	-0.50610214	2.75334318
H	0.50202196	-0.35499818	4.28610529
H	-0.08431218	-1.93817326	3.74931125
C	1.55681195	-1.70369334	-2.42694519
H	2.06399405	-0.73946930	-2.34977619
C	0.17148687	-1.43799724	-3.02437423
H	-0.40217523	-2.36658528	-3.12027724

H	0.29219300	-1.12208700	-4.07686000	H	0.26497890	-0.99023922	-4.02053831
H	-0.44118200	-0.94169000	-2.46893600	H	-0.40882813	-0.75580916	-2.39276219
C	2.48572800	-2.58873600	-3.34446300	C	2.39032796	-2.59342145	-3.34998226
H	3.48376800	-2.71273900	-2.91124000	H	3.38609702	-2.78955253	-2.93738823
H	2.59860800	-2.09956000	-4.31895800	H	2.51514300	-2.10986343	-4.32510233
H	2.07534800	-3.58967300	-3.52335600	H	1.90510887	-3.56027350	-3.52273027
C	4.54203600	-1.39766100	-0.17484600	C	4.52619719	-1.33924649	-0.16167403
H	4.35460800	-2.03527200	0.69507200	H	4.34945914	-1.94486452	0.73184803
H	5.61392700	-1.17665900	-0.21296800	H	5.59852225	-1.12802253	-0.22434503
H	4.27884400	-1.96534700	-1.07156400	H	4.24532413	-1.93132752	-1.03672909
C	4.03340500	0.79861100	-1.30463300	C	4.01932728	0.82980770	-1.35894211
H	3.78219000	0.24300200	-2.21583300	H	3.80403123	0.23557367	-2.25338518
H	5.10819400	1.01311900	-1.35569000	H	5.08713337	1.07124765	-1.39642912
C	3.21132200	2.10776600	-1.22394500	C	3.15914529	2.10996884	-1.33067612
H	3.91345200	2.94794900	-1.13733200	H	3.82745439	2.97790586	-1.28511611
C	2.38845100	2.29962600	-2.49348900	C	2.30458124	2.22589290	-2.59173020
H	1.61248100	1.52760000	-2.55905500	H	1.57216214	1.41316788	-2.65579421
H	3.02971600	2.22308100	-3.37988000	H	2.94233728	2.17196685	-3.48123227
H	1.88912100	3.27351700	-2.52710200	H	1.75294425	3.16914200	-2.63634421
C	1.46833600	3.35108100	0.20755300	C	1.39603424	3.37126003	0.08327499
H	0.68993700	3.24915700	-0.56337300	H	0.61160917	3.21673907	-0.67171107
C	2.21629500	4.66056100	-0.04709200	C	2.09644837	4.69374609	-0.22890303
H	3.03985000	4.79587400	0.66484600	H	2.92015944	4.88709905	0.46876201
H	1.53471200	5.50949300	0.08355700	H	1.38463736	5.51965817	-0.12377003
H	2.63311900	4.72748600	-1.05642400	H	2.49855040	4.73727207	-1.24534211
C	0.75258900	3.45444500	1.55661300	C	0.69857819	3.48883208	1.44011408
H	0.28665700	2.50419800	1.83704600	H	0.26523011	2.53671104	1.76854211
H	-0.03108400	4.22042600	1.51000200	H	-0.11745582	4.21678918	1.37877808
H	1.44504400	3.74684900	2.35558100	H	1.38845727	3.83537307	2.21840014
C	-3.53961000	1.16341500	-0.06239300	C	-3.57613624	1.22813417	0.00807498
C	-3.43858800	2.55835500	-0.07904100	C	-3.48687416	2.62569927	0.04985998
H	-2.46338700	3.03514100	-0.08057900	H	-2.51407906	3.11099024	0.11163099
C	-4.62213100	3.29209800	-0.09016900	C	-4.65911820	3.36741439	0.01193998
H	-4.58128100	4.37646000	-0.10267100	H	-4.60468013	4.45314246	0.04416998
C	-5.86316500	2.64954000	-0.08458200	C	-5.91705134	2.74359142	-0.06661602
H	-6.77094700	3.24486700	-0.09343700	H	-6.81691334	3.35232851	-0.09470803
C	-5.96491800	1.24688200	-0.06684200	C	-6.01563442	1.35865632	-0.10751903
H	-6.94077400	0.77032000	-0.06258500	H	-6.99022852	0.87861034	-0.16766603
C	-4.80224700	0.50288300	-0.05481200	C	-4.84945537	0.58916520	-0.07009902
C	-4.48994600	-0.92108700	-0.03292600	C	-3.15157634	-0.93251901	-0.02707702
C	-5.23207600	-2.08484800	-0.01653200	C	-2.53285937	-2.18921914	-0.02765602
H	-6.31794200	-2.06348500	-0.02014200	H	-1.44971230	-2.27069420	0.02691598
C	-4.54883200	-3.31513500	0.00593400	C	-3.33334049	-3.32004317	-0.09841703
H	-5.12283500	-4.23658600	0.01938700	H	-2.86554951	-4.30223727	-0.09985603
C	-3.15305500	-3.37577600	0.01324500	C	-4.73496959	-3.22174008	-0.16692103
H	-2.65452900	-4.33954900	0.03373900	H	-5.33487768	-4.12645211	-0.22108803
C	-2.38668700	-2.21331000	-0.00294300	C	-5.35565556	-1.97888296	-0.16401903
H	-1.30188200	-2.24479400	0.00605900	H	-6.44006262	-1.90621989	-0.21587203
C	-3.06783900	-0.99135100	-0.02842500	C	-4.57113444	-0.82371792	-0.09361003
Optimized **Ag-2** S_1 semi-coplanar:				Optimized **Ag-2** S_1 orthogonal:			
Ag	0.48661397	0.73993767	-0.08791991	Ag	-0.53649543	-0.39585102	-0.43213627
N	-2.28635079	-0.31085605	0.14664493	N	2.33006271	0.14429789	0.28230003
N	2.61886639	0.41211791	-0.07094149	N	-2.66069185	-0.20075740	-0.30499576
C	-1.56199028	0.81857076	-0.22055494	C	1.49321033	-0.59504517	-0.55810563
C	-2.48871918	1.99536205	-0.43054463	C	2.30084512	-1.63273492	-1.31380445
C	-3.53789518	1.58936906	-1.49472732	C	3.44593037	-0.90287275	-2.05695045
H	-4.22339185	2.42508114	-1.68717093	H	4.03293333	-1.61798604	-2.64819020
H	-3.01757504	1.37903358	-2.43432356	H	3.00418988	-0.19185113	-2.76229370
C	-4.32030121	0.34689988	-1.03772633	C	4.35206861	-0.16028520	-1.06150301
H	-5.38134865	0.57898197	-0.88269650	H	5.35560275	-0.60308642	-1.02978685
H	-4.28168663	-0.45027042	-1.78780326	H	4.48403719	0.88978201	-1.34418875
C	-3.74607813	-0.16439118	0.29228205	C	3.74778535	-0.25105222	0.34773400
C	-1.62397836	-1.56565191	0.24795242	C	1.80786835	1.26953473	0.97525998
C	-1.14971979	-2.01246979	1.49908523	C	1.28878638	1.12122953	2.27820241

C	-0.55963486	-3.27747347	1.58344012	C	0.82408089	2.25456956	2.95186325
H	-0.20823738	-3.63709107	2.54759704	H	0.44055569	2.15183559	3.96441134
C	-0.41279611	-4.07823983	0.45896911	C	0.83840914	3.50529008	2.34936955
H	0.03463723	-5.06525843	0.54517615	H	0.48161161	4.37751884	2.89128318
C	-0.82064025	-3.60073740	-0.78132482	C	1.29748323	3.63251316	1.04426849
H	-0.67820512	-4.21534006	-1.66729115	H	1.28862733	4.60871214	0.56458245
C	-1.41210763	-2.34082706	-0.91100312	C	1.77407557	2.52625224	0.33719304
C	-1.78102210	-1.83476361	-2.28953755	C	2.22337851	2.70088965	-1.09793679
H	-2.19178356	-0.83054705	-2.16636133	H	2.52573056	1.71708809	-1.46142071
C	-2.83365937	-2.71676428	-2.96274536	C	3.41366310	3.65410441	-1.21621581
H	-2.45507419	-3.73090419	-3.13803629	H	3.15125242	4.66587902	-0.88451926
H	-3.12152594	-2.29698766	-3.93384249	H	3.75004049	3.72192906	-2.25771098
H	-3.73699487	-2.80156529	-2.34850276	H	4.25983138	3.31521178	-0.60839510
C	-0.53480820	-1.69649456	-3.16704332	C	1.06273188	3.16039665	-1.98178322
H	0.18485060	-1.01314794	-2.70219664	H	0.24722218	2.43117492	-1.93179912
H	-0.80272959	-1.29181316	-4.15043002	H	1.38459548	3.24335276	-3.02693307
H	-0.03924912	-2.66219006	-3.32490348	H	0.67381831	4.13695662	-1.66909866
C	-1.22326045	-1.14717517	2.74017498	C	1.17835014	-0.23205505	2.95026394
H	-1.69385901	-0.20569337	2.44678045	H	1.52476421	-0.97604501	2.22874591
C	0.17938017	-0.81381428	3.25686244	C	-0.28065033	-0.55993972	3.27887074
H	0.71798126	-1.71624551	3.57067469	H	-0.68689528	0.11964725	4.03785001
H	0.11725947	-0.14104857	4.12072704	H	-0.36498617	-1.58275413	3.66629435
H	0.77205363	-0.31683450	2.48049378	H	-0.90624266	-0.47897590	2.38395170
C	-2.05796339	-1.78484483	3.85272771	C	2.03604320	-0.33508873	4.21308487
H	-3.07976292	-2.00045504	3.52429881	H	3.09588541	-0.16034728	4.00293103
H	-2.11632167	-1.11345789	4.71743544	H	1.94152837	-1.33238012	4.65913580
H	-1.61327330	-2.72737832	4.19378192	H	1.72171649	0.39758461	4.96612576
C	-4.41977433	-1.46017270	0.70458610	C	4.54086929	0.59759889	1.32455643
H	-4.25197680	-2.25159609	-0.03300822	H	4.51568739	1.65624968	1.04733577
H	-5.49903481	-1.29330677	0.78752964	H	5.58541644	0.26805195	1.32147532
H	-4.05490307	-1.81879280	1.67086927	H	4.15778168	0.50984082	2.34469304
C	-3.97766341	0.94536445	1.32515617	C	3.77725063	-1.73403281	0.73772986
H	-3.59665071	0.60898846	2.29692796	H	3.35422496	-1.84242815	1.74368136
H	-5.05749573	1.09704116	1.44670900	H	4.82085224	-2.06789632	0.79775540
C	-3.27982607	2.25794928	0.89355105	C	2.98147959	-2.58821248	-0.27919797
H	-4.05706650	3.00169906	0.66919776	H	3.69386889	-3.21870155	-0.82986500
C	-2.41822329	2.79932379	2.02970415	C	2.00392988	-3.50169228	0.45282022
H	-1.57344401	2.12488732	2.21554034	H	1.22265175	-2.89877689	0.93183457
H	-3.00726865	2.87803927	2.95155124	H	2.52420383	-4.07591861	1.22920033
H	-2.00902273	3.79124737	1.81129866	H	1.51133543	-4.21549091	-0.21561466
C	-1.69592269	3.24572499	-0.88234959	C	1.40967413	-2.41165650	-2.31213634
H	-0.88890436	3.36323752	-0.14249754	H	0.54616183	-2.76248185	-1.72567498
C	-2.51365752	4.53740859	-0.90130106	C	2.08250503	-3.63400458	-2.93802991
H	-3.35292448	4.46793574	-1.60435659	H	2.96894874	-3.34839841	-3.51779357
H	-1.88554024	5.37362727	-1.23089976	H	1.39048947	-4.12861799	-3.63013218
H	-2.92215013	4.80004361	0.07929702	H	2.39475709	-4.37743302	-2.19848638
C	-1.02605393	3.06671826	-2.24604746	C	0.85739245	-1.53115751	-3.43558397
H	-0.50692020	2.10460456	-2.30927164	H	0.45544569	-0.59121394	-3.04314981
H	-0.29617463	3.86664049	-2.41941315	H	0.05576303	-2.05525641	-3.96972414
H	-1.75902260	3.11211999	-3.06101152	H	1.63464961	-1.28781329	-4.17061434
C	3.64010593	1.29800968	0.11708452	C	-3.49498676	-1.02223087	0.40579688
C	3.53161173	2.67129047	0.36689296	C	-3.13787909	-2.16810172	1.12583188
H	2.55439768	3.14178743	0.42053299	H	-2.10336671	-2.49528037	1.15564218
C	4.70778728	3.39675093	0.53651502	C	-4.14753496	-2.86115207	1.78840493
H	4.65759785	4.46290983	0.73310932	H	-3.90250046	-3.75238515	2.35678895
C	5.95451207	2.77017213	0.45247830	C	-5.47335818	-2.42462625	1.72893756
H	6.85673028	3.35932611	0.58464861	H	-6.24221558	-2.98309310	2.25367624
C	6.06763915	1.39195707	0.19836044	C	-5.83696968	-1.27796219	1.00059205
H	7.04759117	0.92729804	0.13773224	H	-6.87458149	-0.95921820	0.96632706
C	4.91230196	0.65474200	0.03080857	C	-4.84828955	-0.57859244	0.33904579
C	3.18411944	-0.81083588	-0.28189694	C	-3.44361929	0.78006532	-0.85111202
C	2.51145528	-2.02140512	-0.49734688	C	-3.02322811	1.84338931	-1.65755404
H	1.42654449	-2.05971810	-0.51455453	H	-1.97481599	1.95596352	-1.91224809
C	3.28177047	-3.16654558	-0.67489970	C	-3.98798662	2.73987249	-2.10878920
H	2.78825817	-4.11885271	-0.84044277	H	-3.69282782	3.57463486	-2.73601892

C	4.67765697	-3.10583273	-0.63505318	C	-5.33164055	2.57999641	-1.76097168
H	5.25533118	-4.01435972	-0.77495502	H	-6.06446409	3.29401141	-2.12356395
C	5.35525222	-1.89294853	-0.41012187	C	-5.75803273	1.51438849	-0.94820606
H	6.44075272	-1.87383817	-0.37465520	H	-6.80758908	1.41208084	-0.68852372
C	4.60994753	-0.74484889	-0.23256950	C	-4.81463649	0.61516097	-0.49506213
Optimized **Ag-2** T_1 semi-coplanar:				Optimized **Cu-2** S_0 semi-coplanar:			
Ag	-0.50580900	0.71563000	-0.07612800	Cu	0.51530804	0.61256304	-0.10283401
N	2.29129700	-0.30021500	-0.00151200	N	-2.15998315	-0.13336501	0.02813800
N	-2.60510400	0.38223200	-0.06384300	N	2.33611517	0.29761902	-0.06099700
C	1.52357600	0.86506900	0.06153400	C	-1.33374910	0.88720807	-0.07405001
C	2.42167100	2.08440000	0.08170000	C	-2.06530115	2.20838916	-0.11137101
C	3.38500700	1.94947100	1.28629100	C	-3.07438322	2.12631415	-1.28548409
H	4.05555200	2.81802700	1.33199900	H	-3.62942326	3.06914822	-1.33749109
H	2.79531100	1.95177600	2.20819900	H	-2.52144818	2.03436715	-2.22473416
C	4.19857900	0.64723900	1.18883800	C	-4.02479429	0.93560607	-1.11132108
H	5.27226000	0.85384700	1.09860200	H	-5.06096337	1.26447509	-0.97448907
H	4.07273300	0.02908500	2.08405300	H	-4.01528929	0.28056502	-1.98827614
C	3.75599300	-0.14515900	-0.05010900	C	-3.62418526	0.13939001	0.13726501
C	1.65686800	-1.56666300	0.11285700	C	-1.65747312	-1.48694611	-0.02326800
C	1.33297300	-2.29587900	-1.05096900	C	-1.47508611	-2.08599615	-1.28170609
C	0.77840800	-3.57286800	-0.91652400	C	-1.07029408	-3.42213925	-1.31364709
H	0.54447600	-4.14805200	-1.80931800	H	-0.92009407	-3.90662928	-2.27426416
C	0.51526900	-4.11269500	0.33570000	C	-0.83537406	-4.13142630	-0.14394701
H	0.09898500	-5.11321500	0.42435600	H	-0.52095004	-5.17008537	-0.19090601
C	0.76618500	-3.35612900	1.47462600	C	-0.95910907	-3.49661425	1.08432008
H	0.53104100	-3.76639400	2.45386400	H	-0.71780305	-4.03757329	1.99471014
C	1.32286800	-2.07744200	1.38467200	C	-1.36523610	-2.16407115	1.17230708
C	1.54004300	-1.27435600	2.64959400	C	-1.38068310	-1.47984011	2.52238318
H	1.92723100	-0.29796500	2.34903700	H	-1.80134313	-0.48071803	2.38727517
C	2.55229700	-1.94233600	3.58186700	C	0.05327500	-1.30068409	3.03237822
H	2.19003000	-2.91568800	3.93403300	H	0.53974504	-2.26953716	3.19148323
H	2.73447100	-1.31602500	4.46324300	H	0.05344700	-0.75927605	3.98563729
H	3.51168700	-2.10793300	3.07924600	H	0.66541505	-0.74079905	2.31636616
C	0.21331000	-1.02290600	3.36945800	C	-2.24513516	-2.21280916	3.54843626
H	-0.48849500	-0.50575500	2.70512200	H	-3.27697024	-2.33428417	3.20015623
H	0.37216900	-0.39484500	4.25434700	H	-2.26799316	-1.65367912	4.49039332
H	-0.25341100	-1.95825600	3.70119100	H	-1.84967313	-3.21037123	3.76919027
C	1.51456200	-1.71059000	-2.43563300	C	-1.62544612	-1.33027710	-2.58613018
H	1.99015300	-0.73537900	-2.31075500	H	-1.95117114	-0.31239402	-2.35651717
C	0.15372100	-1.46860900	-3.09556700	C	-2.67606719	-1.96155214	-3.50056125
H	-0.39871300	-2.40659100	-3.23189000	H	-2.39061317	-2.97684422	-3.79758328
H	0.28205800	-1.00484000	-4.08115000	H	-2.79037820	-1.36957910	-4.41555932
H	-0.45662000	-0.79764700	-2.47994600	H	-3.65417726	-2.02333915	-3.00968822
C	2.40117200	-2.57344200	-3.33472600	C	-0.26869202	-1.21350509	-3.28762624
H	3.38787400	-2.74283600	-2.89093700	H	0.47255403	-0.73643905	-2.63639619
H	2.54650600	-2.08593500	-4.30581100	H	-0.36301003	-0.61491704	-4.20099830
H	1.94898100	-3.55459700	-3.52245400	H	0.12197501	-2.19793816	-3.56830826
C	4.47627000	-1.47920000	-0.12228200	C	-4.43862432	-1.13043708	0.28161102
H	4.24880500	-2.10606400	0.74600300	H	-4.30814031	-1.79539713	-0.57715804
H	5.55714900	-1.30350100	-0.14371000	H	-5.49766541	-0.86063006	0.34787302
H	4.20395100	-2.03889300	-1.02166100	H	-4.17148030	-1.68594712	1.18471709
C	4.07818400	0.73026000	-1.26725700	C	-3.79379827	1.07266107	1.34263210
H	3.81626900	0.18248300	-2.18047000	H	-3.56743026	0.52158704	2.26196716
H	5.16214800	0.89731500	-1.30349100	H	-4.85004435	1.35834110	1.40252110
C	3.31281500	2.07448400	-1.20257900	C	-2.88677921	2.31473817	1.21168109
H	4.04973400	2.88303800	-1.10036700	H	-3.52333526	3.20172023	1.10615208
C	2.53030600	2.30279300	-2.49162000	C	-2.01735315	2.49233418	2.45499618
H	1.72339100	1.56484700	-2.57507800	H	-1.30042410	1.67024412	2.56229618
H	3.18854600	2.19800700	-3.36270600	H	-2.64545919	2.50904118	3.35287724
H	2.07539600	3.29791900	-2.53645200	H	-1.44607011	3.42464724	2.43433717
C	1.58584500	3.38440900	0.18519400	C	-1.06997908	3.38067224	-0.29293302
H	0.82915200	3.31011900	-0.61104000	H	-0.30026602	3.24450323	0.48093903
C	2.38569300	4.66777200	-0.04180000	C	-1.69806912	4.76002934	-0.09444401
H	3.18673300	4.77490800	0.70006000	H	-2.49427518	4.94846336	-0.82461006

H	1.73059900	5.54102100	0.06259100	H	-0.93839807	5.53543840	-0.24066002
H	2.84257700	4.71719000	-1.03478000	H	-2.11911815	4.90035135	0.90566907
C	0.82888800	3.50903200	1.50949900	C	-0.35647003	3.34711724	-1.64585312
H	0.32215000	2.57333100	1.76859900	H	0.03933600	2.35119917	-1.88083813
H	0.07640400	4.30447600	1.44339100	H	0.49087404	4.04066329	-1.64165812
H	1.50449600	3.76901500	2.33376800	H	-1.02362307	3.65037726	-2.46120418
C	-3.63361300	1.28420900	-0.06906900	C	3.35913624	1.21868109	0.00883800
C	-3.52954200	2.67911100	-0.08343100	C	3.27238523	2.61539119	0.03465800
H	-2.55385800	3.15545700	-0.08746800	H	2.30183916	3.10670522	-0.00029200
C	-4.70980400	3.41835300	-0.08896600	C	4.44579232	3.35243224	0.10749201
H	-4.66464500	4.50250000	-0.09959000	H	4.39313432	4.43845532	0.12772401
C	-5.95312000	2.78071800	-0.07994800	C	5.70103839	2.72313120	0.15636201
H	-6.85846700	3.37968300	-0.08425400	H	6.60228249	3.32768724	0.21314902
C	-6.06050500	1.37849600	-0.06443400	C	5.79596839	1.33788810	0.13384001
H	-7.03849300	0.90633600	-0.05741800	H	6.76876350	0.85249006	0.17339601
C	-4.90085700	0.62999900	-0.05821200	C	4.62846333	0.57480304	0.06041600
C	-3.16804400	-0.86409400	-0.04172500	C	4.34936231	-0.83822306	0.02173100
C	-2.48937400	-2.08807100	-0.02208900	C	5.13505637	-1.99333215	0.04390500
H	-1.40459700	-2.12412800	-0.01667200	H	6.21904947	-1.92109914	0.10113201
C	-3.25577700	-3.25027900	-0.00630300	C	4.51600433	-3.23570723	-0.00730600
H	-2.75783000	-4.21425900	0.01000600	H	5.11685437	-4.14117730	0.00986300
C	-4.65143600	-3.18843700	-0.00817300	C	3.11604022	-3.33241524	-0.08313301
H	-5.22590100	-4.10952300	0.00513500	H	2.64816819	-4.31359931	-0.12417201
C	-5.33450600	-1.95769800	-0.02468400	C	2.31544717	-2.19998616	-0.10805801
H	-6.42037800	-1.93649900	-0.02428700	H	1.23395309	-2.28075016	-0.16929701
C	-4.59191600	-0.79468000	-0.04067200	C	2.93291121	-0.94608807	-0.05317100

Optimized **Cu-2** S_1 semi-coplanar:				Optimized **Cu-2** S_1 orthogonal:			
Cu	0.45354022	0.62410632	-0.33594955	Cu	-0.56925174	-0.29886182	-0.41732084
N	-2.13619643	-0.24446526	0.31184860	N	2.20324221	0.03784671	0.21942777
N	2.35225338	0.39628912	-0.29239044	N	-2.44848254	-0.08661712	-0.27415358
C	-1.41729628	0.75755328	-0.33332346	C	1.26316183	-0.63888817	-0.55913375
C	-2.34148297	1.88502580	-0.73732439	C	1.94248160	-1.75298708	-1.33384465
C	-3.49971011	1.29090940	-1.57544621	C	3.12042413	-1.14077071	-2.12859914
H	-4.17146921	2.09018669	-1.91406752	H	3.60413180	-1.90671116	-2.74867788
H	-3.07775159	0.82782911	-2.47328205	H	2.71944186	-0.38364535	-2.81043342
C	-4.27975134	0.24566608	-0.75994416	C	4.14119544	-0.49989883	-1.17460714
H	-5.29714925	0.59235350	-0.53923962	H	5.08365606	-1.06196746	-1.16426276
H	-4.38533941	-0.69682770	-1.30815431	H	4.39221955	0.52165622	-1.48074766
C	-3.56290597	-0.00036574	0.57732781	C	3.57447663	-0.49816646	0.25346844
C	-1.47109850	-1.47250626	0.58377338	C	1.81232525	1.21712052	0.90847630
C	-1.39549980	-2.45878662	-0.42276805	C	1.89912195	2.46283286	0.25403837
C	-0.73859962	-3.65978228	-0.14350628	C	1.55077831	3.62053809	0.95314302
H	-0.69526556	-4.43323016	-0.90726421	H	1.63180768	4.58653860	0.45976599
C	-0.14072810	-3.88055752	1.09204250	C	1.10372907	3.55660645	2.26701904
H	0.36289051	-4.82206715	1.29559216	H	0.84689235	4.46722279	2.80229657
C	-0.17871787	-2.88600114	2.05893741	C	0.97135822	2.32136615	2.88688155
H	0.30930769	-3.05074835	3.01664562	H	0.59701406	2.27103925	3.90676620
C	-0.82842558	-1.66996697	1.82307183	C	1.30410155	1.13810191	2.22109847
C	-0.78596438	-0.59282987	2.88716810	C	1.06585206	-0.18948004	2.91074593
H	-1.32725804	0.27016511	2.49172871	H	1.30325896	-0.97228611	2.18590230
C	0.65245574	-0.14279695	3.15743131	C	-0.40929614	-0.34862433	3.28819409
H	1.26006460	-0.96024155	3.56432603	H	-0.70754262	0.36742075	4.06352848
H	0.66503271	0.67912185	3.88343179	H	-0.59941461	-1.35704811	3.67553224
H	1.13014772	0.20767073	2.23613875	H	-1.05325853	-0.18850297	2.41769361
C	-1.45564861	-1.03501873	4.18998508	C	1.94851775	-0.37829013	4.14597004
H	-2.49552572	-1.33849921	4.03147091	H	3.01349542	-0.33069405	3.89772517
H	-1.44913091	-0.21661700	4.91948699	H	1.75446768	-1.35283881	4.60983791
H	-0.92786153	-1.88458168	4.63965358	H	1.74742084	0.39462249	4.89758054
C	-1.99530091	-2.24588587	-1.79685674	C	2.33806726	2.57190339	-1.19088765
H	-2.41954959	-1.24103317	-1.81027670	H	2.55063725	1.55989694	-1.54010419
C	-3.11101771	-3.25064642	-2.08926679	C	3.60268071	3.41733418	-1.34824713
H	-2.72766977	-4.27763894	-2.11846979	H	3.43435131	4.45425972	-1.03380121
H	-3.57314961	-3.03988294	-3.06100042	H	3.92430187	3.43579654	-2.39639126
H	-3.89486777	-3.21128828	-1.32471495	H	4.42776250	3.01860095	-0.74779389

C	-0.92260501	-2.28116376	-2.88677576	C	1.20427977	3.10924660	-2.06527521
H	-0.18153827	-1.49273296	-2.71532202	H	0.33965030	2.44109383	-1.99710242
H	-1.37373198	-2.11213721	-3.87184563	H	1.51685066	3.15833612	-3.11531548
H	-0.40282232	-3.24675368	-2.91887409	H	0.89182888	4.11419858	-1.75696189
C	-4.20882396	-1.14133894	1.33990849	C	4.47819109	0.28355000	1.18885017
H	-4.13186508	-2.08431566	0.78896687	H	4.55147298	1.33385264	0.88936961
H	-5.27057162	-0.91996868	1.49221881	H	5.48369338	-0.15004790	1.16379857
H	-3.74603049	-1.28498969	2.32035810	H	4.11875619	0.25477300	2.22106391
C	-3.64378504	1.31951268	1.35624004	C	3.46229488	-1.96881546	0.67548158
H	-3.14192879	1.19031290	2.32264917	H	3.04823708	-2.01223311	1.68996867
H	-4.69590627	1.54030994	1.57538275	H	4.46713066	-2.40675419	0.72577759
C	-2.99068647	2.47464051	0.55859971	C	2.56500016	-2.75709264	-0.30947662
H	-3.78370944	3.16769376	0.24391858	H	3.20139827	-3.45374643	-0.87353507
C	-2.01078234	3.23760386	1.44372819	C	1.53056753	-3.57181772	0.46031547
H	-1.15027303	2.59823251	1.67826371	H	0.81653410	-2.89390587	0.94500795
H	-2.49194683	3.53156427	2.38441043	H	2.01695523	-4.17520073	1.23634002
H	-1.63227135	4.14728592	0.96583577	H	0.96285472	-4.25169264	-0.18342776
C	-1.56645155	2.95935951	-1.53603110	C	0.93930868	-2.45126467	-2.28367328
H	-0.69918375	3.22065020	-0.91008636	H	0.07742405	-2.72432050	-1.65479694
C	-2.35765019	4.23916600	-1.80546658	C	1.46999310	-3.72945105	-2.93412503
H	-3.24496411	4.03863577	-2.41850855	H	2.34405667	-3.52317245	-3.56401674
H	-1.73792165	4.95409905	-2.35972917	H	0.70145526	-4.16715383	-3.58231200
H	-2.69212367	4.73488425	-0.88880445	H	1.75916410	-4.49259866	-2.20513796
C	-1.01211231	2.42805019	-2.85875128	C	0.41311083	-1.52387890	-3.38099369
H	-0.51149452	1.46407321	-2.71592485	H	0.11115224	-0.55516757	-2.96765700
H	-0.28980056	3.13605135	-3.28236072	H	-0.45327626	-1.97520195	-3.87905766
H	-1.80835830	2.29025791	-3.60072907	H	1.17454350	-1.34125004	-4.14936924
C	3.28915130	1.31888208	0.09155599	C	-3.27682505	-0.93458749	0.42182398
C	3.05633231	2.62880998	0.52542293	C	-2.90856193	-2.08841369	1.12159642
H	2.04297134	3.01474657	0.58093112	H	-1.87034907	-2.40311193	1.14973921
C	4.15916529	3.40316716	0.87542604	C	-3.91246734	-2.80654162	1.76588296
H	4.01179431	4.42194104	1.21938206	H	-3.65791048	-3.70474007	2.31915436
C	5.45530821	2.88781711	0.78338001	C	-5.24412873	-2.38758601	1.70633599
H	6.29858518	3.51492239	1.05597343	H	-6.00831881	-2.96623289	2.21600406
C	5.69235152	1.57509257	0.34217680	C	-5.61803910	-1.23510858	0.99537352
H	6.70824049	1.19612184	0.27736455	H	-6.65974262	-0.93025634	0.95725430
C	4.60976304	0.78918868	-0.00218458	C	-4.63384383	-0.51008253	0.35244335
C	4.43858992	-0.57661853	-0.47590623	C	-4.61564540	0.69454058	-0.46543374
C	5.28857902	-1.63011640	-0.74945352	C	-5.57574218	1.58157739	-0.91069288
H	6.36473260	-1.53078072	-0.64061532	H	-6.62368252	1.45410309	-0.65549541
C	4.73074787	-2.85157183	-1.16649788	C	-5.16863352	2.66469927	-1.70746301
H	5.38981311	-3.68683685	-1.38273938	H	-5.91373166	3.36909048	-2.06407555
C	3.34870693	-3.01693503	-1.29221994	C	-3.82656948	2.85519547	-2.04625577
H	2.94873219	-3.97889653	-1.59663452	H	-3.54438916	3.70397237	-2.66070757
C	2.47550997	-1.96809023	-1.01855362	C	-2.84603217	1.97224322	-1.60221741
H	1.39949653	-2.09214322	-1.07747417	H	-1.79942323	2.11193530	-1.84903763
C	3.03030951	-0.74425728	-0.62617943	C	-3.24823208	0.88996622	-0.81362986
Optimized **Cu-2** T_1 semi-coplanar:				Optimized **Au-3** S_0 semi-coplanar:			
Cu	-0.53757700	0.64896600	-0.07953000	C	3.37808100	1.65799600	-0.09638900
N	2.16801200	-0.18974800	-0.00118500	H	3.59869400	2.63670800	0.33860700
N	-2.38247500	0.32965900	-0.07320800	H	3.63028700	1.70810300	-1.16554200
C	1.30238000	0.90469400	0.06076800	C	3.65910900	-0.79110000	0.23321200
C	2.09694800	2.19221800	0.15500000	O	4.34002400	-1.79069900	0.17124700
C	3.03787700	2.08580200	1.37925300	C	-3.27662400	-1.45558000	-0.01550200
H	3.61898200	3.01044800	1.49290500	C	-4.67609600	-1.20026400	0.01040100
H	2.42578400	1.98029400	2.28052900	C	-5.57549000	-2.26951800	0.04615400
C	3.97720000	0.87526300	1.23921500	H	-6.64765800	-2.08706200	0.06630800
H	5.02479500	1.19117600	1.15771400	C	-5.08314200	-3.56856600	0.05570100
H	3.91546100	0.21758100	2.11301300	H	-5.77264500	-4.40807900	0.08347600
C	3.61431900	0.08675300	-0.02811500	C	-3.69817400	-3.80855400	0.02811000
C	1.63432800	-1.50505800	0.05242200	H	-3.33357500	-4.83311900	0.03401800
C	1.34733500	-2.09638900	1.30050100	C	-2.78384600	-2.76490000	-0.00856700
C	0.90156000	-3.42049500	1.33403100	H	-1.71298900	-2.95255300	-0.03290600
H	0.70183600	-3.89083700	2.29392300	C	1.40974000	0.20871000	0.03498700

C	0.71018900	-4.14362000	0.16219200	N	-2.56694700	-0.27438500	-0.04765300
H	0.37827000	-5.17797200	0.20579400	Au	-0.56454100	-0.05684600	-0.01486400
C	0.91911400	-3.52762300	-1.06507300	C	4.20733500	0.57832500	0.58940700
H	0.72723400	-4.07926000	-1.98231100	N	2.26553300	-0.86276800	0.06314300
C	1.36508700	-2.20437700	-1.14290500	C	-3.48853200	0.75097500	-0.04458900
C	1.48965800	-1.54196800	-2.49874100	C	-4.81404300	0.23536400	-0.00855000
H	1.86558900	-0.53089300	-2.32747400	C	-5.90010700	1.11501200	0.00161900
C	0.11275800	-1.40703200	-3.15565600	H	-6.91818200	0.73292100	0.02920600
H	-0.34058100	-2.38796000	-3.34412200	C	-5.66365800	2.48390200	-0.02345000
H	0.19594600	-0.88300300	-4.11537900	H	-6.50030800	3.17740700	-0.01576600
H	-0.56753700	-0.83546100	-2.51414900	C	-4.34949700	2.98193400	-0.05999500
C	2.46028400	-2.27148000	-3.42844100	H	-4.18569400	4.05689000	-0.08064200
H	3.45976800	-2.35244800	-2.98829200	C	-3.25366400	2.13014300	-0.07129400
H	2.55242100	-1.73421700	-4.37955400	H	-2.23782400	2.51735700	-0.10129000
H	2.11345700	-3.28685800	-3.65447900	C	1.68078400	-2.17979000	-0.04406900
C	1.48518200	-1.32628200	2.59643900	C	1.56629100	-2.75679300	-1.31415000
H	1.80621600	-0.31481500	2.33770200	C	1.00359300	-4.03199800	-1.39526600
C	2.52656100	-1.94997300	3.52673200	H	0.89769800	-4.50736100	-2.36643300
H	2.23287200	-2.95982900	3.83734900	C	0.57769200	-4.69877000	-0.25356000
H	2.64348000	-1.34401900	4.43297600	H	0.14320400	-5.69115000	-0.33592900
H	3.50515600	-2.02413500	3.03951400	C	0.69467700	-4.09521200	0.99306300
C	0.12935900	-1.19731900	3.29474600	H	0.34605600	-4.61949500	1.87809200
H	-0.59576900	-0.70645600	2.63581800	C	1.24446400	-2.81920700	1.12476200
H	0.22461900	-0.59493000	4.20607500	C	0.86361000	-1.85590700	-3.54887400
H	-0.27553600	-2.17591800	3.57971500	H	0.47676900	-2.81769000	-3.90380800
C	4.44211000	-1.18084500	-0.13262600	H	1.19424100	-1.28595600	-4.42484000
H	4.25681600	-1.85168100	0.71259700	H	0.03769800	-1.31483100	-3.07498800
H	5.50524900	-0.91776200	-0.13378300	C	2.02341700	-2.04725500	-2.57037300
H	4.22585900	-1.73166300	-1.05263100	H	2.37748600	-1.04928500	-2.28879100
C	3.87044800	1.02348200	-1.21531900	C	3.19977600	-2.78294200	-3.21530400
H	3.64184400	0.48834900	-2.14473400	H	2.90623400	-3.78779800	-3.54001500
H	4.93966600	1.26777200	-1.24959200	H	4.02910300	-2.88356900	-2.50789100
C	3.01312900	2.30812200	-1.10496800	H	3.55598500	-2.23719300	-4.09663200
H	3.68948700	3.15944100	-0.94583900	C	1.35785400	-2.17475100	2.49007300
C	2.24367700	2.54473000	-2.40041300	H	1.63270400	-1.12480800	2.34580800
H	1.47980300	1.76787100	-2.52673500	C	2.47023900	-2.83776400	3.30645800
H	2.92152100	2.50839500	-3.26180000	H	2.23487900	-3.89058200	3.50116000
H	1.73678700	3.51529500	-2.41783300	H	2.59052400	-2.33457000	4.27283000
C	1.14918600	3.40972000	0.28813100	H	3.42587100	-2.80155900	2.77272900
H	0.42836700	3.31153300	-0.53902500	C	0.02352400	-2.18029400	3.23676700
C	1.84082800	4.76634400	0.14911200	H	-0.76168400	-1.70523200	2.63857300
H	2.59805000	4.90886400	0.92984300	H	0.11846700	-1.63060000	4.18016300
H	1.10795000	5.57462000	0.25920400	H	-0.30322500	-3.19764300	3.47850200
H	2.33232500	4.89815400	-0.81943700	N	1.94279700	1.42256600	0.02616500
C	0.34423600	3.40315500	1.58993300	C	4.13704300	0.69868000	2.11983100
H	-0.08655600	2.41563700	1.78956500	H	3.11540800	0.60463400	2.49803400
H	-0.47159800	4.13465100	1.53952300	H	4.52637500	1.67438100	2.43043200
H	0.97083000	3.67592200	2.44791800	H	4.74449600	-0.08222400	2.58666200
C	-3.40742200	1.24595200	-0.09964900	C	5.65603900	0.68839000	0.12394300
C	-3.29367200	2.63782000	-0.15074300	H	5.73354500	0.57487500	-0.96230100
H	-2.31616200	3.11008300	-0.16679300	H	6.26302700	-0.09077600	0.58960600
C	-4.46892300	3.38459300	-0.17600000	H	6.06427300	1.66593000	0.40190400
H	-4.41515100	4.46789800	-0.21513700	C	1.07861300	2.56961300	-0.10587900
C	-5.71758900	2.75706800	-0.14998400	C	0.59594600	3.19697800	1.05293800
H	-6.61845500	3.36269400	-0.16989100	C	-0.17944600	4.34671600	0.89081000
C	-5.83300200	1.35897800	-0.09763800	H	-0.57190800	4.85030900	1.76980900
H	-6.81308500	0.89125600	-0.07879300	C	-0.46941800	4.84423100	-0.37331000
C	-4.67643900	0.60237300	-0.07156800	H	-1.07454600	5.74026700	-0.47840300
C	-4.38339500	-0.82414400	-0.01817200	C	-0.00916600	4.18160200	-1.50415900
C	-5.14515600	-1.97656600	0.02737700	H	-0.26847900	4.55826800	-2.48958700
H	-6.23057300	-1.93624500	0.02811900	C	0.76827400	3.02690000	-1.39660600
C	-4.48307100	-3.21485000	0.07353300	C	-0.47587600	2.27594700	3.12359200
H	-5.07057900	-4.12722300	0.11044500	H	-1.10360500	3.15823300	3.29117000
C	-3.08800900	-3.29601700	0.07487200	H	-0.28031200	1.81310300	4.09764800
H	-2.60299500	-4.26621100	0.11351000	H	-1.04636300	1.56739100	2.51321600

C	-2.30454000	-2.14592600	0.02981700
H	-1.22102800	-2.19864300	0.03535500
C	-2.96417900	-0.91524700	-0.01876000

C	0.84292800	2.64637900	2.44063100
H	1.41671200	1.72214600	2.33498400
C	1.66734600	3.61337800	3.29189500
H	1.13382200	4.55786500	3.44855500
H	2.62698400	3.84708400	2.81707900
H	1.87027900	3.17724800	4.27642300
C	1.19972000	2.29374900	-2.65053500
H	1.73093000	1.38275300	-2.35296800
C	2.15521200	3.13933700	-3.49520700
H	1.66499700	4.05377900	-3.84792200
H	2.48628100	2.57711400	-4.37542900
H	3.04189100	3.43716700	-2.92412500
C	-0.01891400	1.84829200	-3.46249100
H	-0.68791400	1.23072100	-2.85362200
H	0.29839900	1.26105500	-4.33116000
H	-0.59120700	2.70769900	-3.82873000

Optimized **Au-3** S_1 semi-coplanar:

C	3.41782400	1.39793300	-0.68637600
H	3.78263000	2.42547700	-0.58781100
H	3.50201300	1.12160600	-1.75091900
C	3.63155000	-0.92300600	0.20854500
O	4.27455900	-1.97305100	0.32996700
C	-3.37715000	-1.31607800	-0.18635600
C	-4.78054300	-1.07614500	-0.07265400
C	-5.66764900	-2.11381600	-0.28179100
H	-6.74046200	-1.96385400	-0.20114700
C	-5.15515700	-3.38431000	-0.60166600
H	-5.84571700	-4.20562200	-0.76672400
C	-3.77919100	-3.61295900	-0.70393900
H	-3.41755300	-4.60796700	-0.94337400
C	-2.86557200	-2.58492200	-0.49350000
H	-1.79308500	-2.74914300	-0.54855600
C	1.41431600	0.16018200	-0.02076000
N	-2.66758800	-0.17930400	0.05763700
Au	-0.58219600	-0.01029800	0.03337600
C	4.29022000	0.44464800	0.13720600
N	2.24731900	-0.92490400	0.16321500
C	-3.55686600	0.81678500	0.33134500
C	-4.89914200	0.33284600	0.26941100
C	-5.94788000	1.19969500	0.50758000
H	-6.97945300	0.86204900	0.46498500
C	-5.65733600	2.54323300	0.80492200
H	-6.47572500	3.23126500	0.99341300
C	-4.34075800	3.01234500	0.85344700
H	-4.15275600	4.05816200	1.07500000
C	-3.26802100	2.15939500	0.61233000
H	-2.24041700	2.51125800	0.62338700
C	1.58718100	-2.19198800	0.32008400
C	1.30598000	-2.95159700	-0.82251500
C	0.64563900	-4.17017400	-0.64651700
H	0.42525400	-4.78880100	-1.51270300
C	0.27392000	-4.60388700	0.62165500
H	-0.23702800	-5.55585600	0.74119900
C	0.55662200	-3.82421700	1.73715500
H	0.26332200	-4.17203900	2.72394600
C	1.21858300	-2.60098700	1.60782500
C	0.57063600	-2.52379500	-3.19847300
H	0.20450600	-3.54295100	-3.36963400
H	0.89413900	-2.12383600	-4.16665500
H	-0.26640500	-1.91394400	-2.83983900
C	1.72848200	-2.49334400	-2.20161300
H	2.05136500	-1.45047800	-2.11693200
C	2.92566400	-3.31384900	-2.68824700
H	2.65592100	-4.37102900	-2.80077300

Optimized **Au-3** S_1 orthogonal:

C	-3.54575200	1.12922500	0.39265600
H	-3.98186300	2.12909900	0.29379200
H	-3.75034600	0.78596900	1.42111300
C	-3.46122300	-1.15685900	-0.57814900
O	-4.01224800	-2.24615300	-0.78916700
C	3.51611300	0.32973500	-1.03092100
C	4.88526800	0.17348700	-0.67238900
C	5.87427600	0.46539800	-1.58977100
H	6.92547600	0.35556200	-1.34057200
C	5.49292000	0.91194800	-2.86727700
H	6.26188600	1.14461500	-3.59720200
C	4.14827700	1.06220200	-3.21576300
H	3.88791600	1.40833500	-4.21061300
C	3.13832900	0.77466500	-2.30189500
H	2.08875900	0.88509700	-2.55195900
C	-1.37284600	0.07955900	-0.03991200
N	2.69117400	-0.00747800	0.01160200
Au	0.62155400	0.04880200	-0.00477600
C	-4.21708000	0.16036300	-0.58387000
N	-2.10605400	-1.06265800	-0.35439100
C	3.49245700	-0.39359200	1.05445800
C	4.86990900	-0.30320000	0.70369500
C	5.83805500	-0.65055800	1.62368300
H	6.89483900	-0.59053600	1.38107400
C	5.42778600	-1.09114500	2.89450700
H	6.18036100	-1.36744200	3.62629000
C	4.07524900	-1.18476100	3.23246600
H	3.79248100	-1.53419200	4.22000400
C	3.08618900	-0.83929800	2.31632500
H	2.03088000	-0.91418400	2.55410500
C	-1.34940200	-2.28147500	-0.43808400
C	-1.18018000	-3.05085200	0.71964400
C	-0.42475600	-4.22130400	0.61990800
H	-0.28516600	-4.84528300	1.49875100
C	0.14225500	-4.60291400	-0.59024900
H	0.72560500	-5.51809500	-0.65118600
C	-0.04212600	-3.82169700	-1.72551600
H	0.39952600	-4.13359000	-2.66775500
C	-0.79215900	-2.64515400	-1.67218000
C	-0.86111600	-2.69744700	3.20247600
H	-0.49555500	-3.71116300	3.40322800
H	-1.35385200	-2.33886000	4.11388100
H	0.00424900	-2.05512500	3.00671100
C	-1.83614100	-2.66300700	2.02732800
H	-2.18285700	-1.62960700	1.92625700
C	-3.06195500	-3.54408500	2.28382700
H	-2.76855600	-4.59441000	2.40229900

H	3.74995500	-3.24517700	-1.97149300
H	3.27330400	-2.94875700	-3.66219700
C	1.55422000	-1.76707900	2.82565000
H	1.87491400	-0.78243600	2.47090500
C	2.72519500	-2.39328400	3.58773300
H	2.45305600	-3.38082800	3.97997200
H	3.01573800	-1.76113200	4.43541600
H	3.59021000	-2.51537900	2.92816900
C	0.34131600	-1.54154200	3.72721600
H	-0.47737700	-1.07793200	3.16500600
H	0.60713500	-0.87619100	4.55702300
H	-0.02802200	-2.47805700	4.16110800
N	2.03590600	1.36208700	-0.24669700
C	4.47452600	0.95762000	1.57074200
H	3.51866900	1.04471000	2.09459300
H	4.95283300	1.94488400	1.56652100
H	5.10819800	0.26639400	2.13554800
C	5.65095100	0.30859600	-0.54119800
H	5.54544000	-0.05597100	-1.56899700
H	6.27107600	-0.40587200	0.00441900
H	6.16203600	1.27798300	-0.56715000
C	1.19758100	2.49403300	-0.48437200
C	0.82393500	3.30096400	0.60515000
C	0.03791100	4.42888900	0.35572500
H	-0.24765300	5.07283700	1.18403600
C	-0.37749000	4.74133000	-0.93349700
H	-0.97938000	5.62862400	-1.11180300
C	-0.03388000	3.91000400	-1.99206200
H	-0.38186500	4.14582500	-2.99457500
C	0.74134600	2.76572000	-1.78695300
C	0.03328900	2.80159400	2.94303800
H	-0.54866100	3.72709500	3.02922900
H	0.36151600	2.52002600	3.95046800
H	-0.62868200	2.01232800	2.56958700
C	1.24208200	2.96998300	2.02134800
H	1.75362700	2.00577500	1.98607000
C	2.22254600	4.00991600	2.56646200
H	1.75644100	5.00060700	2.62910300
H	3.10528600	4.09561300	1.92336400
H	2.55891400	3.73247900	3.57224900
C	1.04006800	1.84184300	-2.95043600
H	1.53625700	0.95171000	-2.55110400
C	1.97521700	2.50389000	-3.96479100
H	1.50854500	3.39162700	-4.40812900
H	2.21499900	1.80954800	-4.77838900
H	2.91390700	2.82021700	-3.49683900
C	-0.24771800	1.35657900	-3.61916800
H	-0.89768900	0.86532400	-2.88640400
H	-0.01426700	0.63385300	-4.40959300
H	-0.80612400	2.18254900	-4.07461800

H	-3.76121500	-3.47549800	1.44493300
H	-3.57671800	-3.23149200	3.20063000
C	-1.03640200	-1.81489600	-2.91490100
H	-1.32835200	-0.81271900	-2.58205200
C	-2.19634400	-2.40388100	-3.72419100
H	-1.93230100	-3.39804500	-4.10585600
H	-2.43179000	-1.76314400	-4.58285200
H	-3.09116600	-2.50629600	-3.10308400
C	0.21507800	-1.65601400	-3.77581900
H	1.05518300	-1.27900100	-3.18243700
H	0.02248800	-0.94869300	-4.59108900
H	0.52204600	-2.60350200	-4.23389500
N	-2.12271100	1.22995400	0.13607700
C	-4.19683700	0.70792600	-2.01546600
H	-3.17526200	0.89855700	-2.35636400
H	-4.75892100	1.64775500	-2.07498600
H	-4.65587800	-0.01449100	-2.69827200
C	-5.65580600	-0.08144900	-0.13818600
H	-5.69232700	-0.47513600	0.88354000
H	-6.13457300	-0.81206300	-0.79367900
H	-6.22567600	0.85447400	-0.17054200
C	-1.44564000	2.39798100	0.60247600
C	-1.04244700	3.36421500	-0.33634700
C	-0.45843600	4.54407600	0.12967700
H	-0.15644900	5.30820800	-0.58178500
C	-0.26200200	4.75641200	1.48857300
H	0.18406500	5.68469600	1.83606500
C	-0.62579900	3.77461100	2.40124100
H	-0.45248300	3.93754500	3.46214600
C	-1.20762600	2.57718600	1.97751600
C	0.02416600	3.44178200	-2.62247400
H	0.28425800	4.50672400	-2.60760200
H	-0.11714200	3.15355800	-3.67087600
H	0.87562200	2.88235400	-2.22115100
C	-1.24360800	3.14869300	-1.82035000
H	-1.47986000	2.08989400	-1.95679100
C	-2.42306200	3.97619500	-2.33588600
H	-2.22693200	5.04898000	-2.21816100
H	-3.34117500	3.74176900	-1.78721900
H	-2.60249400	3.77878100	-3.39943700
C	-1.54782400	1.51011200	2.99820200
H	-1.86342200	0.61597300	2.45216200
C	-2.69095400	1.95153300	3.91480300
H	-2.40581700	2.83320000	4.50114900
H	-2.95290100	1.15151500	4.61716400
H	-3.58763900	2.20987600	3.34123300
C	-0.31549400	1.11218000	3.81190000
H	0.49432700	0.79869900	3.14479600
H	-0.55486800	0.27898500	4.48268600
H	0.05209500	1.94212300	4.42642000

Optimized **Au-3** T_1 semi-coplanar:

C	3.33712400	1.64031100	-0.38725100
H	3.65062700	2.65694800	-0.12801000
H	3.44998500	1.53120800	-1.47937600
C	3.66656800	-0.77980500	0.13146900
O	4.37237600	-1.79642500	0.11705500
C	-3.31990500	-1.48025400	0.03106200
C	-4.72448600	-1.24065600	0.03220300
C	-5.59776300	-2.30905300	0.05103000
H	-6.67353700	-2.16046300	0.05210400
C	-5.06363500	-3.61110000	0.06855100
H	-5.74203900	-4.45849800	0.08344500
C	-3.68447600	-3.83708400	0.06732300
H	-3.30730900	-4.85453700	0.08092600

Optimized **Ag-3** S_0 semi-coplanar:

Ag	0.63667500	-0.07373300	0.08642100
N	2.67983000	-0.25665900	0.10507700
N	-1.95321200	1.39239600	-0.08829500
C	3.42108600	-1.41410300	0.12162800
C	2.96218400	-2.73733400	0.15396500
H	1.89529000	-2.95147000	0.16830700
C	3.89947500	-3.76049300	0.16591800
H	3.55873500	-4.79322200	0.19074700
C	5.27978100	-3.49027300	0.14578400
H	5.98819500	-4.31447300	0.15574000
C	5.74179500	-2.18066700	0.11227300
H	6.81009500	-1.97487700	0.09541000
C	4.81847300	-1.13102500	0.09954400

C	-2.78594000	-2.77367800	0.04860500
H	-1.71103500	-2.93025300	0.04820500
C	1.39241400	0.20774200	-0.00147500
N	-2.62392400	-0.30275900	0.01029900
Au	-0.57830300	-0.07211900	0.00388300
C	4.24645800	0.61708700	0.29958800
N	2.29509200	-0.84988500	0.02648800
C	-3.53656000	0.71833600	-0.00244300
C	-4.86712100	0.21088100	0.01012600
C	-5.93152800	1.08965200	-0.00056800
H	-6.95791000	0.73494200	0.00855800
C	-5.66066600	2.47040800	-0.02414200
H	-6.49075100	3.17002900	-0.03268800
C	-4.35172400	2.95957900	-0.03686800
H	-4.17783400	4.03062300	-0.05518000
C	-3.26392900	2.09040300	-0.02610000
H	-2.24019900	2.45311800	-0.03517300
C	1.70637000	-2.15779800	-0.04554200
C	1.48671700	-2.73192100	-1.30370400
C	0.89558300	-3.99683300	-1.34946200
H	0.72297900	-4.47192100	-2.31162500
C	0.53406800	-4.65819700	-0.18101000
H	0.08289000	-5.64596700	-0.23409200
C	0.75134100	-4.05969700	1.05558600
H	0.46611300	-4.58353400	1.96416500
C	1.33766200	-2.79500800	1.14680100
C	0.76039600	-1.93556600	-3.58376800
H	0.44037900	-2.92475400	-3.93178600
H	1.07560400	-1.36250400	-4.46381600
H	-0.10505800	-1.43175300	-3.13856800
C	1.90541100	-2.02827900	-2.57611600
H	2.18105100	-1.00345400	-2.30772900
C	3.14234100	-2.70397200	-3.17327000
H	2.92119300	-3.73692900	-3.46855700
H	3.95635300	-2.72466100	-2.44176800
H	3.48399900	-2.16423900	-4.06473300
C	1.59208800	-2.15497800	2.49460500
H	1.87061800	-1.11205200	2.31436900
C	2.76884700	-2.84043600	3.19386000
H	2.53696000	-3.88989100	3.41331200
H	2.99867600	-2.33971100	4.14206900
H	3.65938800	-2.81399300	2.55807200
C	0.33933400	-2.13669500	3.36966700
H	-0.48192500	-1.62291400	2.85642600
H	0.54130900	-1.60623000	4.30765100
H	0.00420800	-3.14800000	3.62855200
N	1.95890600	1.46889500	0.02735500
C	4.36409100	0.90460700	1.80080300
H	3.38722600	0.87948900	2.29149400
H	4.80390600	1.89590800	1.96618300
H	5.00524000	0.15611000	2.27737200
C	5.62789000	0.66358500	-0.34733600
H	5.56701800	0.47033300	-1.42410700
H	6.27256700	-0.10017900	0.09264400
H	6.08593500	1.64813000	-0.19717700
C	1.06591400	2.57883100	-0.05945300
C	0.59589700	3.16233400	1.13128900
C	-0.22021900	4.29289400	1.04241100
H	-0.57719700	4.76526700	1.95421100
C	-0.57933500	4.82141000	-0.19221700
H	-1.20224400	5.71084900	-0.24431100
C	-0.15690100	4.19763200	-1.35997700
H	-0.46771700	4.59431200	-2.32332600
C	0.65237600	3.05887100	-1.31640200
C	-0.32707800	2.22437200	3.27189500
C	-1.41229300	0.19003200	0.00386700
C	-4.25819000	0.53285400	0.25718700
O	-4.31789100	-1.84668600	-0.12127500
C	-1.08360400	2.54047200	-0.16692100
C	-0.61988700	2.94646600	-1.42889100
C	0.17659000	4.09192700	-1.48671300
H	0.55389500	4.42897800	-2.44795200
C	0.50932500	4.79407000	-0.33530300
H	1.13491100	5.67962400	-0.40216900
C	0.06397600	4.35056000	0.90338800
H	0.35419400	4.88661900	1.80268600
C	-0.74127200	3.21552600	1.01506700
C	-0.91486800	2.17529600	-2.69960700
H	-1.47968800	1.27423000	-2.43466100
C	0.38148300	1.70828000	-3.36669700
H	0.15544200	1.09041000	-4.24250600
H	0.99095000	1.11623800	-2.67530100
H	0.98635700	2.55761900	-3.70244700
C	-1.77014900	2.99766500	-3.66635800
H	-2.00698300	2.41058900	-4.56064300
H	-1.23971300	3.90042400	-3.98991700
H	-2.71170300	3.31376700	-3.20336100
C	3.57775400	0.78382800	0.06936600
C	3.31012800	2.15859000	0.03813800
H	2.28413500	2.52155400	0.04120800
C	4.38192900	3.03908400	0.00230800
H	4.18949800	4.10946100	-0.02305600
C	5.71017300	2.57616200	-0.00233600
H	6.52790900	3.29156600	-0.03078500
C	5.98282600	1.21422800	0.02878900
H	7.01146500	0.85960000	0.02480000
C	4.92089000	0.30570700	0.06464500
N	-2.25446300	-0.88858600	-0.02698500
C	-3.65948700	-0.83635600	-0.01095900
C	-3.37302600	1.61708600	-0.34975900
C	-1.63419900	-2.19337500	-0.02893200
C	-1.27337700	-2.76725600	1.19892800
C	-0.66522600	-4.02328800	1.16874600
H	-0.37239800	-4.49667900	2.10133800
C	-0.42285700	-4.67217500	-0.03621600
H	0.05510700	-5.64779900	-0.03913900
C	-0.77920900	-4.07170100	-1.23698100
H	-0.57562600	-4.58193500	-2.17423600
C	-1.53445500	-2.07766700	2.52178300
H	-1.82329400	-1.04268900	2.31210800
C	-0.28183500	-2.01556100	3.39628100
H	-0.48478500	-1.43348800	4.30235300
H	0.54886300	-1.54129700	2.86204500
H	0.04526400	-3.01258800	3.71088200
C	-2.70377000	-2.74486500	3.25098900
H	-2.93065800	-2.21293000	4.18217800
H	-2.46122800	-3.78320400	3.50526500
H	-3.60378200	-2.75463600	2.62691600
C	-1.16864000	2.72666100	2.38176100
H	-1.79443300	1.84255400	2.23828900
C	0.04547600	2.28917600	3.20512500
H	-0.27609300	1.88238200	4.17077200
H	0.71922500	3.13089100	3.40095800
H	0.61931500	1.51733500	2.68038700
C	-2.00645500	3.76971200	3.12276600
H	-2.34533600	3.37055700	4.08527100
H	-2.88964100	4.05829200	2.54175700
H	-1.42759900	4.67792000	3.32496100
C	-4.35546600	0.67426700	1.78475000
H	-5.01142900	-0.10022500	2.19296800

H	-0.92384200	3.11237000	3.51218500	H	-4.77366300	1.65449800	2.03850200
H	-0.06062400	1.73617300	4.21674700	H	-3.38151100	0.58317900	2.27372300
H	-0.95446500	1.53400900	2.69640600	C	-5.64961700	0.61745900	-0.36279400
C	0.93608300	2.58081400	2.48616300	H	-6.09670000	1.59340500	-0.14553700
H	1.47582400	1.64836700	2.30530900	H	-5.60866000	0.48811300	-1.44924500
C	1.84776100	3.51558000	3.28229500	H	-6.29435900	-0.16295300	0.04672500
H	1.35311000	4.47252700	3.48830800	C	-1.76739100	-2.17905800	-2.57874500
H	2.77186500	3.72696600	2.73341300	C	-0.53763800	-1.98855500	-3.46827500
H	2.11853200	3.06346900	4.24364600	H	0.23210400	-1.40165300	-2.95565700
C	1.02690900	2.35616300	-2.60594700	H	-0.09358600	-2.95020300	-3.74867300
H	1.54185900	1.42772000	-2.34128800	H	-0.81263900	-1.46654600	-4.39204700
C	1.96665000	3.20919900	-3.46051700	C	-2.86151300	-2.98353400	-3.28369400
H	1.48050200	4.14306600	-3.76719500	H	-3.74125100	-3.08758400	-2.64081000
H	2.25835700	2.66935400	-4.36885700	H	-2.50751300	-3.98817600	-3.54207400
H	2.87749900	3.47291200	-2.91203000	H	-3.16386700	-2.48618300	-4.21246900
C	-0.21990800	1.94775500	-3.39337400	H	-2.17558400	-1.18357900	-2.37171600
H	-0.86874900	1.31303100	-2.77928000	C	-1.39273600	-2.81770700	-1.25872500
H	0.06585000	1.38082600	-4.28704500	H	-3.51596000	1.66090100	-1.43885400
H	-0.79948400	2.81881000	-3.72060700	H	-3.64403700	2.59584600	0.05624200
Optimized **Ag-3** S_1 semi-coplanar:				Optimized **Ag-3** S_1 orthogonal:			
Ag	0.65319700	-0.09577300	0.03337200	Ag	0.69254600	0.08792800	-0.00491800
N	2.78037600	-0.28865700	0.05200800	N	2.81433800	0.07380400	-0.01772000
N	-1.96934700	1.43442700	0.06724700	N	-2.16698900	1.16052500	-0.21333700
C	3.50631500	-1.43901700	0.12508900	C	3.62381200	-0.40561500	-1.01183100
C	3.00886800	-2.74562800	0.22680300	C	3.22377700	-0.97327600	-2.22658700
H	1.93842200	-2.93110400	0.25725700	H	2.16999600	-1.07560700	-2.46310100
C	3.93239000	-3.78513900	0.28422000	C	4.21621100	-1.40173900	-3.10343100
H	3.58097600	-4.80909800	0.36202300	H	3.93755000	-1.84626400	-4.05314700
C	5.30633800	-3.52852600	0.24145700	C	5.56738900	-1.26961500	-2.77346700
H	6.00488000	-4.35827900	0.28765300	H	6.32260300	-1.61264900	-3.47353700
C	5.80716200	-2.21799600	0.13893000	C	5.97350600	-0.70413300	-1.55133400
H	6.87916000	-2.04572500	0.10604100	H	7.02970500	-0.61516700	-1.31517300
C	4.90912500	-1.17067200	0.08078200	C	5.00196200	-0.27329300	-0.67158400
C	-1.39099700	0.18540500	0.02245000	C	-1.35940100	0.05960700	0.03068100
C	-4.24774400	0.54762800	0.33931600	C	-4.22504200	0.04060100	0.52954200
O	-4.35042700	-1.85543700	0.00802000	O	-3.90711300	-2.33013900	0.92436400
C	-1.08168200	2.54939500	-0.00844200	C	-1.52660000	2.32161800	-0.74014600
C	-0.65784100	3.02959500	-1.26207400	C	-1.25794500	2.42340800	-2.11812600
C	0.16084400	4.16180000	-1.29844400	C	-0.70337400	3.61083300	-2.60302000
H	0.48127500	4.55764900	-2.25898100	H	-0.51016600	3.71371500	-3.66805300
C	0.57887400	4.78146600	-0.12667000	C	-0.39347300	4.65929200	-1.74610500
H	1.20998800	5.66537600	-0.17318800	H	0.03084800	5.57868400	-2.14127400
C	0.20232600	4.25802800	1.10447800	C	-0.61581100	4.52490900	-0.38125100
H	0.55248800	4.72980300	2.01916100	H	-0.35580900	5.34142800	0.28746600
C	-0.62298400	3.13334400	1.18614500	C	-1.17401500	3.35721100	0.14433000
C	-1.03807600	2.33797000	-2.55568600	C	-1.54359100	1.28584600	-3.07810300
H	-1.56828300	1.41654000	-2.29671200	H	-1.83426700	0.41506800	-2.48282600
C	0.20458700	1.91765900	-3.34330500	C	-0.28855200	0.88432000	-3.85496900
H	-0.08571700	1.35960100	-4.24082900	H	-0.49309200	0.00770700	-4.48042700
H	0.84714500	1.27182200	-2.73411200	H	0.52566200	0.63348500	-3.16631800
H	0.79615800	2.78318800	-3.66352300	H	0.06004700	1.68977600	-4.51169500
C	-1.96374300	3.20846500	-3.40819400	C	-2.68825400	1.63185600	-4.03328800
H	-2.26260600	2.67599400	-4.31852400	H	-2.91311500	0.78301800	-4.68979900
H	-1.46355100	4.13606800	-3.71116000	H	-2.42539400	2.48698700	-4.66753300
H	-2.87097700	3.48353300	-2.85936100	H	-3.60096900	1.89193400	-3.48655800
C	3.66376100	0.74539600	-0.03898300	C	3.63537300	0.51994700	0.98330100
C	3.35765200	2.11002200	-0.13268600	C	3.24894200	1.08989000	2.20121900
H	2.32542900	2.44953500	-0.13640800	H	2.19782700	1.21884600	2.43720300
C	4.41949100	3.00546300	-0.21798300	C	4.25119000	1.47845500	3.08579600
H	4.21599300	4.06907200	-0.29154800	H	3.98310800	1.92291500	4.03858000
C	5.74264100	2.55323000	-0.20851100	C	5.59866300	1.30355100	2.76069900
H	6.55237200	3.27326100	-0.27559700	H	6.36178300	1.61551800	3.46671100
C	6.05139100	1.18413900	-0.11300900	C	5.99091700	0.73173200	1.53719300
H	7.08769100	0.85865800	-0.10714400	H	7.04454500	0.60586100	1.30633500

C	5.01326400	0.27736800	-0.02784500	C	5.00941400	0.33971700	0.65001300
N	-2.27967500	-0.87390100	-0.00867800	N	-2.05529400	-1.08865200	0.40868800
C	-3.65690800	-0.83013800	0.08592800	C	-3.40429200	-1.23410900	0.63076200
C	-3.35968700	1.61951700	-0.30177300	C	-3.57873700	0.98190700	-0.49064700
C	-1.66248900	-2.16617000	-0.12127700	C	-1.23614900	-2.25476900	0.58335000
C	-1.27618000	-2.82936600	1.05193600	C	-0.65875600	-2.49104800	1.83930800
C	-0.64545700	-4.06944200	0.92166500	C	0.16724400	-3.60901000	1.97704800
H	-0.34448300	-4.61333100	1.81298100	H	0.62457400	-3.82268600	2.93906800
C	-0.40369100	-4.61879800	-0.33308800	C	0.40582700	-4.45702100	0.90133800
H	0.08299700	-5.58735100	-0.41654400	H	1.04737400	-5.32538600	1.02766800
C	-0.78643900	-3.93402500	-1.48092500	C	-0.18132800	-4.20208600	-0.33249400
H	-0.59507600	-4.37262900	-2.45649900	H	0.00125500	-4.87807600	-1.16373700
C	-1.56750600	-2.24157300	2.41645400	C	-0.96399000	-1.59172700	3.01896100
H	-1.79289300	-1.18026300	2.27069600	H	-1.32479100	-0.64005200	2.61398200
C	-0.36534600	-2.31559800	3.35636500	C	0.27044900	-1.28277900	3.86370600
H	-0.59198100	-1.80132200	4.29748600	H	0.02457200	-0.53859400	4.63031900
H	0.51233100	-1.83451600	2.90860100	H	1.08203400	-0.88400700	3.24454400
H	-0.09822000	-3.34937000	3.60480100	H	0.64784800	-2.17163700	4.38266000
C	-2.80581400	-2.90652400	3.02454200	C	-2.08399800	-2.19984400	3.86898900
H	-3.06337100	-2.43833100	3.98231900	H	-2.36490800	-1.51839800	4.68142200
H	-2.62425700	-3.97299700	3.20663900	H	-1.75654000	-3.14588200	4.31797700
H	-3.66128400	-2.81722200	2.34771000	H	-2.96728100	-2.40228600	3.25588400
C	-0.99388400	2.56354500	2.53769300	C	-1.40382300	3.22629000	1.63435800
H	-1.53264500	1.63171700	2.35216400	H	-1.61424200	2.17126700	1.82894900
C	0.24790100	2.21187600	3.35855800	C	-0.16431100	3.60359000	2.44551400
H	-0.04355400	1.73449900	4.30147700	H	-0.32522100	3.38164700	3.50707500
H	0.84144100	3.10028000	3.60476900	H	0.07232300	4.67108600	2.36550200
H	0.89083400	1.51498400	2.80863900	H	0.71087700	3.04023500	2.10400800
C	-1.92352500	3.50727800	3.30219600	C	-2.61838800	4.04728200	2.07283600
H	-2.22089700	3.06400500	4.25973900	H	-2.81613300	3.91017900	3.14261900
H	-2.83216200	3.71596500	2.72688800	H	-3.51595400	3.74957800	1.52080900
H	-1.43196300	4.46486400	3.51256200	H	-2.45254900	5.11649200	1.89239200
C	-4.34373500	0.74785200	1.85686200	C	-4.27425900	0.67994700	1.92168500
H	-4.97820500	-0.02811700	2.29715300	H	-4.71591700	-0.01969000	2.63881900
H	-4.77949800	1.72772700	2.08868100	H	-4.88335500	1.59205500	1.90494000
H	-3.36034700	0.69177100	2.33222600	H	-3.27309000	0.94409900	2.27399000
C	-5.63969800	0.61833300	-0.28206700	C	-5.63627500	-0.30611400	0.06483200
H	-6.10547700	1.58707200	-0.06677700	H	-6.25629400	0.59708700	0.02232300
H	-5.59395900	0.48907300	-1.36914300	H	-5.62213900	-0.76411500	-0.93047500
H	-6.26950800	-0.17729500	0.12148100	H	-6.09350800	-1.02034500	0.75292500
C	-1.86869900	-1.96226400	-2.64450700	C	-1.68886200	-2.84411800	-1.84625400
C	-0.76676800	-1.87887800	-3.69927700	C	-0.71438300	-2.90808800	-3.02068800
H	0.13569100	-1.41415200	-3.28613600	H	0.10971800	-2.19979800	-2.88065100
H	-0.49474700	-2.86707800	-4.08807500	H	-0.28623100	-3.90940200	-3.14602100
H	-1.10367100	-1.27414400	-4.54938300	H	-1.22895200	-2.65112600	-3.95407800
C	-3.14283700	-2.60508700	-3.19931200	C	-2.86020900	-3.81312500	-2.03051800
H	-3.92435400	-2.62076600	-2.43333700	H	-3.56232200	-3.72120400	-1.19638000
H	-2.95166100	-3.63743400	-3.51715600	H	-2.50539600	-4.85040500	-2.07029800
H	-3.50999300	-2.04609200	-4.06860700	H	-3.39226600	-3.60150600	-2.96612900
H	-2.11289500	-0.93604800	-2.35102800	H	-2.09671300	-1.82858900	-1.81956600
C	-1.42031800	-2.69187200	-1.39683900	C	-1.01147100	-3.09365300	-0.51579600
H	-3.49294200	1.57740600	-1.39628500	H	-3.74668300	0.56955900	-1.50042500
H	-3.67569900	2.61654500	0.02438200	H	-4.06441500	1.96352000	-0.45909800
Optimized **Ag-3** T_1 semi-coplanar:				Optimized **Cu-3** S_0 semi-coplanar:			
Ag	0.64658500	-0.10466100	0.01005400	N	2.41596500	-0.33065300	0.14740800
N	2.74383300	-0.30088700	0.02853200	N	-1.74923200	1.47346300	-0.17618900
N	-1.96152400	1.44397000	0.02102600	C	3.09852200	-1.52678900	0.10695300
C	3.47478700	-1.45506900	0.06251700	C	2.56957500	-2.82035300	0.03332000
C	2.97718400	-2.76295000	0.09898300	H	1.49451000	-2.97582800	-0.00023100
H	1.90698000	-2.95053900	0.10461000	C	3.45047600	-3.89195600	0.00341200
C	3.90253900	-3.80295400	0.12711800	H	3.05399000	-4.90326600	-0.05271600
H	3.55257900	-4.82974100	0.15500100	C	4.84261600	-3.69649800	0.04313200
C	5.27527200	-3.54134100	0.11949900	H	5.50636100	-4.55675000	0.01803600
H	5.97522600	-4.37078400	0.14196700	C	5.37371700	-2.41456900	0.11184700

C	5.77540400	-2.22648400	0.08321600	H	6.45118200	-2.26645800	0.13980100
H	6.84706600	-2.05073300	0.07760100	C	4.50688900	-1.31822600	0.14341600
C	4.87453500	-1.18154700	0.05466600	C	-1.25748800	0.25522700	0.00125900
C	-1.37761600	0.18826200	-0.00015000	C	-4.08754300	0.73512200	0.20250800
C	-4.24782000	0.57901300	0.24870900	O	-4.25142800	-1.66140500	0.00850200
O	-4.35346300	-1.83841600	0.07595100	C	-0.83186000	2.56697000	-0.38604800
C	-1.06550200	2.55029300	-0.05989900	C	-0.25143600	2.73288400	-1.65391700
C	-0.62182200	3.01727800	-1.31207000	C	0.58789700	3.83492300	-1.84015700
C	0.19887900	4.14844300	-1.34962200	H	1.05559900	3.98797800	-2.80831900
H	0.53042800	4.53522500	-2.31013800	C	0.84026800	4.72828300	-0.80939200
C	0.60495100	4.77812600	-0.17921900	H	1.49930100	5.57561300	-0.97580400
H	1.23656500	5.66173200	-0.22652400	C	0.26607100	4.53119900	0.44156400
C	0.21696600	4.26308700	1.05231000	H	0.48664300	5.22499200	1.24636000
H	0.55926100	4.74091700	1.96690400	C	-0.58230600	3.44981600	0.67901800
C	-0.61190100	3.14097700	1.13419600	C	-0.48661100	1.77717600	-2.80623900
C	-0.98314800	2.31146000	-2.60375300	H	-1.08837300	0.93591100	-2.44429500
H	-1.50438900	1.38636700	-2.34053200	C	0.83480600	1.19740300	-3.31724900
C	0.26998900	1.89591900	-3.37715900	H	0.64425700	0.46029600	-4.10445700
H	-0.00829400	1.32524700	-4.27077200	H	1.39105900	0.70640200	-2.51079800
H	0.91322900	1.26234200	-2.75587000	H	1.47686400	1.97820000	-3.73932000
H	0.85599300	2.76318500	-3.70297400	C	-1.26710800	2.45552200	-3.93522500
C	-1.90901000	3.16555300	-3.47247700	H	-1.46690600	1.74162500	-4.74212100
H	-2.19308500	2.62361900	-4.38204800	H	-0.69872900	3.29069800	-4.36000400
H	-1.41508700	4.09612400	-3.77691600	H	-2.22574400	2.85170500	-3.58165900
H	-2.82470200	3.43520100	-2.93520900	C	3.37151200	0.66053400	0.20527200
C	3.63078800	0.73982200	-0.00184300	C	3.17753600	2.04601900	0.25588600
C	3.32138600	2.10416500	-0.04362200	H	2.17393900	2.46366300	0.25416300
H	2.28875900	2.44142800	-0.05378200	C	4.29478000	2.86749000	0.30417100
C	4.38311200	3.00449500	-0.07102500	H	4.15886800	3.94607600	0.34076500
H	4.17849500	4.06968600	-0.10353300	C	5.59580900	2.33378100	0.30578200
C	5.70551700	2.55321900	-0.05676500	H	6.45086300	3.00353400	0.34517600
H	6.51505800	3.27614600	-0.07842100	C	5.79498600	0.95955400	0.25741600
C	6.01619200	1.18129800	-0.01485900	H	6.80281500	0.54974100	0.25860400
H	7.05256700	0.85688400	-0.00505500	C	4.68546700	0.11076900	0.20568000
C	4.97757700	0.27277900	0.01230500	N	-2.14884700	-0.78617100	0.03921700
N	-2.28199200	-0.86906100	0.01020700	C	-3.54948500	-0.67477000	0.04364300
C	-3.65392100	-0.81447000	0.09509900	C	-3.15349600	1.72500300	-0.48637600
C	-3.33267500	1.61093800	-0.41906300	C	-1.59288100	-2.11536200	0.14242800
C	-1.67033700	-2.16609100	-0.04547300	C	-1.23845200	-2.60051900	1.41004700
C	-1.29361100	-2.78330000	1.15525100	C	-0.74394100	-3.90364700	1.48749400
C	-0.66407900	-4.02861900	1.08029700	H	-0.45951700	-4.30880800	2.45424100
H	-0.37290400	-4.53674300	1.99595400	C	-0.60661000	-4.68629500	0.34769200
C	-0.41320700	-4.62845500	-0.14914400	H	-0.22076000	-5.69858300	0.42894100
H	0.07063200	-5.60127900	-0.18991700	C	-0.94151900	-4.16946400	-0.89751200
C	-0.78451700	-3.98831800	-1.32656400	H	-0.81237500	-4.78134100	-1.78586500
H	-0.58657000	-4.46556200	-2.28266100	C	-1.38373400	-1.76976600	2.66771800
C	-1.58776300	-2.14349100	2.49532800	H	-1.64333600	-0.74823900	2.37224800
H	-1.89158500	-1.10999500	2.30343800	C	-0.06904100	-1.68450000	3.44477000
C	-0.35196700	-2.08619500	3.39256500	H	-0.17985900	-1.00831800	4.29998400
H	-0.58481100	-1.55601800	4.32351500	H	0.74094900	-1.30644100	2.81045700
H	0.46564400	-1.55303900	2.89281600	H	0.23663000	-2.66269000	3.83190000
H	0.00672500	-3.08614800	3.66367200	C	-2.52603300	-2.30183000	3.53652200
C	-2.75729700	-2.85732800	3.17825600	H	-2.66582400	-1.66695000	4.41899600
H	-3.01479800	-2.36022700	4.12129200	H	-2.31158200	-3.31922000	3.88349700
H	-2.50258100	-3.90014700	3.40409400	H	-3.46666200	-2.33095200	2.97595500
H	-3.63796000	-2.85403700	2.52837400	C	-1.15160700	3.19317200	2.05780700
C	-0.99303700	2.57994200	2.48689000	H	-2.08634900	2.63889700	1.93015800
H	-1.52459700	1.64369600	2.30228500	C	-0.19465600	2.30444200	2.86016100
C	0.24236300	2.24144700	3.32278600	H	-0.61433000	2.07629000	3.84700700
H	-0.05596700	1.76581900	4.26447500	H	0.76847900	2.80730800	3.00371900
H	0.82658500	3.13524100	3.57214900	H	0.00338300	1.35850000	2.34310600
H	0.89606500	1.54634100	2.78347500	C	-1.48561800	4.46884400	2.82783700
C	-1.93362800	3.52522800	3.23589700	H	-2.01559300	4.21482500	3.75206500
H	-2.23534800	3.08938900	4.19560000	H	-2.12217700	5.13971900	2.24074900
H	-2.83942500	3.72301900	2.65254400	H	-0.58370600	5.02021600	3.11540600

H	-1.44893300	4.48760300	3.44067200
C	-4.39972500	0.86884300	1.74632700
H	-5.04307400	0.11508400	2.21159500
H	-4.85210000	1.85641000	1.90095300
H	-3.43251800	0.85386700	2.25631100
C	-5.61614800	0.61354700	-0.42644600
H	-6.08722100	1.59336100	-0.28514100
H	-5.53106200	0.42191000	-1.50199600
H	-6.26183100	-0.15737400	-0.00053700
C	-1.85118600	-2.06212200	-2.57682900
C	-0.72701600	-1.99450300	-3.60915300
H	0.15343200	-1.49183300	-3.19252500
H	-0.42265500	-2.99051000	-3.95156500
H	-1.05657100	-1.43112100	-4.49011000
C	-3.10245000	-2.74323100	-3.13745200
H	-3.90116900	-2.74610700	-2.38913600
H	-2.88986700	-3.78248600	-3.41696300
H	-3.45919100	-2.21889500	-4.03228800
H	-2.11649700	-1.03110800	-2.32210400
C	-1.41708000	-2.74291200	-1.29693700
H	-3.42665100	1.50353700	-1.51334000
H	-3.65969600	2.62475000	-0.16389600

Optimized **Cu-3** S_1 semi-coplanar:

N	2.51122600	-0.31760600	0.12467600
N	-1.78699600	1.46800500	-0.25986600
C	3.17806500	-1.49389700	-0.05546200
C	2.61429300	-2.75483700	-0.29136500
H	1.53683200	-2.88053700	-0.34299500
C	3.48224200	-3.83155000	-0.44422900
H	3.07664800	-4.82159900	-0.62731300
C	4.86696300	-3.65618100	-0.35765200
H	5.52153800	-4.51404300	-0.47756200
C	5.43348900	-2.39237200	-0.11380500
H	6.51214300	-2.28259900	-0.04691300
C	4.59089400	-1.30821000	0.03790000
C	-1.24182500	0.22616000	-0.02337500
C	-4.10688900	0.69575100	0.03009700
O	-4.25677600	-1.71751200	0.21831600
C	-0.85901300	2.52313100	-0.51528000
C	-0.28646400	2.66753000	-1.79338500
C	0.59025800	3.73342200	-2.01235800
H	1.03011200	3.86819400	-2.99725800
C	0.91300900	4.61844200	-0.99103600
H	1.59600600	5.44237300	-1.18066800
C	0.37090100	4.43957900	0.27592700
H	0.64129300	5.12144200	1.07859200
C	-0.51681800	3.39190100	0.53711800
C	-0.58686500	1.70142800	-2.92164500
H	-1.18825800	0.88496700	-2.51036700
C	0.69352600	1.06822700	-3.46985100
H	0.44998500	0.32061700	-4.23329600
H	1.25233900	0.56905300	-2.66993900
H	1.35195400	1.81428200	-3.92963600
C	-1.38783200	2.37962600	-4.03552200
H	-1.63822000	1.65889700	-4.82257600
H	-0.81368300	3.19282700	-4.49529700
H	-2.32132600	2.80626500	-3.65252700
C	3.44900500	0.65291400	0.33261100
C	3.21823300	2.01973900	0.53455500
H	2.20802400	2.41786500	0.53346400
C	4.32607500	2.84224600	0.71604300
H	4.18025800	3.90639800	0.87298600
C	5.62217200	2.31819000	0.68909000
H	6.46917700	2.98256100	0.83047300

C	-4.17540500	0.98998800	1.71587900
H	-4.86622700	0.27656700	2.17496700
H	-4.55065000	2.00270600	1.90031400
H	-3.20547900	0.88601000	2.21143000
C	-5.47438600	0.83550200	-0.42458600
H	-5.88241200	1.84006600	-0.26979600
H	-5.43872400	0.63719900	-1.50083900
H	-6.14932800	0.10818500	0.03137000
C	-1.78544600	-2.32643200	-2.39529500
C	-0.55818700	-2.32765000	-3.30870800
H	0.27319000	-1.77985400	-2.85256800
H	-0.21383800	-3.34737800	-3.51412700
H	-0.79768100	-1.85734900	-4.26951800
C	-2.95653500	-3.09198600	-3.01385300
H	-3.83114000	-3.05839300	-2.35692300
H	-2.69542900	-4.14342800	-3.18038500
H	-3.22863300	-2.65763300	-3.98272500
H	-2.10057100	-1.28380800	-2.27656100
C	-1.44010100	-2.87168400	-1.02661100
H	-3.27658400	1.66721800	-1.57792400
H	-3.39522600	2.74838200	-0.18427300
Cu	0.58670500	-0.07193200	0.10524000

Optimized **Cu-3** S_1 orthogonal:

N	2.53865800	0.21074500	-0.10714800
N	-2.12157000	1.02367100	-0.24132300
C	3.38708500	-0.40080600	-0.99439600
C	3.03536100	-1.21418200	-2.07599500
H	1.99307400	-1.42870600	-2.28372700
C	4.06127500	-1.74322700	-2.85376500
H	3.81946800	-2.37792900	-3.69998100
C	5.39867600	-1.47232300	-2.55365600
H	6.18084400	-1.89863100	-3.17400600
C	5.75594600	-0.66495000	-1.45998900
H	6.80226800	-0.47422500	-1.24059400
C	4.74993100	-0.13086900	-0.67986200
C	-1.21508300	0.01951600	0.06084800
C	-4.03807000	-0.17568600	0.72505100
O	-3.50708300	-2.46323900	1.32325700
C	-1.59817000	2.16377600	-0.92374800
C	-1.38255800	2.12418400	-2.31379000
C	-0.93612200	3.28400500	-2.95307100
H	-0.78455800	3.27517300	-4.02967000
C	-0.67944100	4.44421700	-2.23490400
H	-0.33617900	5.33853500	-2.74851400
C	-0.85047900	4.45528600	-0.85604300
H	-0.63151800	5.36093600	-0.29714500
C	-1.30304100	3.32091400	-0.17865700
C	-1.61307800	0.86772500	-3.12898000
H	-1.82084600	0.05173100	-2.43055400
C	-0.35511700	0.47132400	-3.90327500
H	-0.51442300	-0.47733100	-4.42848200
H	0.49316500	0.34982300	-3.22054400
H	-0.08149500	1.22457700	-4.65098600
C	-2.80913000	1.02282800	-4.07106400
H	-2.99136200	0.09111700	-4.61952000
H	-2.63012100	1.81622800	-4.80659900
H	-3.72077500	1.27946000	-3.52079500
C	3.32545300	0.87739600	0.79971000
C	2.89523500	1.64002000	1.88983600
H	1.83587000	1.75406300	2.09422000
C	3.86564000	2.23518100	2.69136600
H	3.56295100	2.83133000	3.54603500
C	5.22426500	2.07582200	2.40663300
H	5.96186500	2.55206700	3.04499900

C	5.85555300	0.94853200	0.47366200	C	5.65892100	1.31311500	1.30939000
H	6.87213300	0.56653100	0.44719000	H	6.72014200	1.20386800	1.10626300
C	4.77000000	0.11292500	0.29382400	C	4.70870100	0.71338100	0.50647800
N	-2.16208500	-0.79787500	0.11926000	N	-1.79544700	-1.13973800	0.58383500
C	-3.54069200	-0.71229500	0.11489800	C	-3.11164100	-1.36804400	0.89771100
C	-3.14556100	1.60050200	-0.74841800	C	-3.52463800	0.70484000	-0.41855400
C	-1.58488300	-2.09644200	0.32476900	C	-0.85984700	-2.21096000	0.78499900
C	-1.24752100	-2.48034200	1.63030800	C	-0.14226700	-2.26482800	1.98983400
C	-0.68549800	-3.74610700	1.81349600	C	0.81796800	-3.26955900	2.13147400
H	-0.42098000	-4.07461700	2.81475300	H	1.39109000	-3.33805900	3.05144000
C	-0.47085700	-4.59465800	0.73334100	C	1.04778400	-4.18660200	1.11214100
H	-0.04175000	-5.58017000	0.89508400	H	1.79802000	-4.96259300	1.24063900
C	-0.80104700	-4.18378800	-0.55351700	C	0.31258300	-4.12097300	-0.06546100
H	-0.62793500	-4.85256000	-1.39274200	H	0.48791800	-4.85387900	-0.84854100
C	-1.52959300	-1.57385000	2.80971900	C	-0.44481600	-1.29962000	3.11775200
H	-1.71085500	-0.57011000	2.41251600	H	-0.86645300	-0.39585500	2.66256000
C	-0.34036200	-1.46422300	3.76226200	C	0.79816300	-0.87908900	3.89866600
H	-0.55545200	-0.73271300	4.54976300	H	0.54344900	-0.08107200	4.60578700
H	0.55855200	-1.13537400	3.22761800	H	1.58445000	-0.50886100	3.23217100
H	-0.11466600	-2.41803800	4.25309000	H	1.21410800	-1.70750100	4.48404800
C	-2.79964400	-2.03181600	3.53232100	C	-1.50098300	-1.90027600	4.05202500
H	-3.05427200	-1.33788100	4.34248700	H	-1.79203800	-1.17417600	4.82103600
H	-2.66066100	-3.02786200	3.97044100	H	-1.10150300	-2.78807400	4.55817900
H	-3.64236600	-2.08275700	2.83543300	H	-2.39211300	-2.20181200	3.49392200
C	-1.05378200	3.18783500	1.93660100	C	-1.49823100	3.35307400	1.32195500
H	-1.77457400	2.36997900	1.88144300	H	-1.51554800	2.31222200	1.65952200
C	0.06182100	2.74685200	2.88716500	C	-0.35784700	4.06129300	2.05320900
H	-0.33736400	2.56999900	3.89300600	H	-0.46094200	3.92078500	3.13546500
H	0.84774100	3.50844300	2.96518800	H	-0.35736000	5.14145800	1.86569900
H	0.52227700	1.81637900	2.53592300	H	0.61760800	3.67055000	1.74512900
C	-1.78175300	4.42521900	2.46139600	C	-2.84157200	3.99792000	1.67403300
H	-2.21542400	4.22204900	3.44728500	H	-3.01885000	3.96752200	2.75571300
H	-2.59231700	4.72266000	1.78704900	H	-3.67149400	3.48395200	1.17959900
H	-1.10374200	5.28029300	2.56801700	H	-2.85912600	5.04732400	1.35505300
C	-4.30786900	1.19780300	1.46568400	C	-4.06336200	0.58811000	2.05340600
H	-5.02678900	0.55682500	1.98589000	H	-4.39179600	-0.07669100	2.85904300
H	-4.69115400	2.22584200	1.46627300	H	-4.75798500	1.43477400	1.99757200
H	-3.37279700	1.18017400	2.03293500	H	-3.07223200	0.97360400	2.30967600
C	-5.44863900	0.65690100	-0.69632000	C	-5.43849400	-0.67464600	0.38286300
H	-5.89776100	1.65660600	-0.72193000	H	-6.13313400	0.16905000	0.29661600
H	-5.32974600	0.30365800	-1.72668800	H	-5.44126900	-1.22436600	-0.56489400
H	-6.13058000	-0.02636700	-0.18570700	H	-5.79563200	-1.35237100	1.16125700
C	-1.72356900	-2.48425700	-2.18488400	C	-1.49554400	-3.10004700	-1.51131000
C	-0.55785000	-2.64841200	-3.15971800	C	-0.66293300	-3.21231700	-2.78639600
H	0.32605100	-2.10419600	-2.80823000	H	0.08024300	-2.41029700	-2.84054100
H	-0.28016200	-3.70081400	-3.29130600	H	-0.13729500	-4.17184400	-2.85275400
H	-0.83045900	-2.25487300	-4.14599100	H	-1.31011400	-3.13127900	-3.66763600
C	-2.97554700	-3.22351200	-2.66316600	C	-2.56738300	-4.19238600	-1.44718600
H	-3.80100200	-3.06182100	-1.96340200	H	-3.17657400	-4.07259000	-0.54664600
H	-2.78972900	-4.30228200	-2.73371300	H	-2.10608700	-5.18746400	-1.42416800
H	-3.27770700	-2.86781600	-3.65560000	H	-3.22085800	-4.14275200	-2.32677800
H	-1.96142800	-1.41637000	-2.14202800	H	-2.00590500	-2.13181800	-1.54186800
C	-1.35607900	-2.92178300	-0.78366800	C	-0.65390600	-3.13013300	-0.25309500
H	-3.20844100	1.34284400	-1.81984500	H	-3.70383800	0.17562700	-1.37081300
H	-3.45039300	2.64826700	-0.65030700	H	-4.09151600	1.64142800	-0.45984100
Cu	0.61475300	-0.06815200	0.08597100	Cu	0.65221400	0.13143900	-0.07161200

Optimized **Cu-3** T_1 semi-coplanar:				Optimized **Au-1^{2CN}** S_0 semi-coplanar			
N	2.47118900	-0.38289300	0.06792100	Au	0.30859700	-0.01365400	-0.00412700
N	-1.75409100	1.51144800	-0.05290300	N	-2.45456900	1.11068700	0.03731600
C	3.13558700	-1.58322500	0.07273400	N	-2.48531200	-1.07545100	-0.04378000
C	2.56229200	-2.85777900	0.06219300	N	-4.86815500	1.48834700	0.05395500
H	1.48430900	-2.98453200	0.05066100	N	-4.90880800	-1.38470800	-0.04906500
C	3.42448300	-3.95107100	0.06822200	N	2.33350800	-0.02135300	-0.00361800
H	3.01184700	-4.95486100	0.06059100	C	-1.66625800	0.00623900	-0.00440600

C	4.81031200	-3.77150100	0.08400000	C	-3.78539800	0.73833800	0.02486800
H	5.46027000	-4.64112700	0.08855500	C	-3.80522700	-0.66556500	-0.02577600
C	5.38528800	-2.48867100	0.09368500	C	-6.01731400	-0.63373200	-0.01976000
H	6.46523500	-2.37404700	0.10483300	H	-6.96541200	-1.16301000	-0.03641500
C	4.54619500	-1.39145300	0.08802300	C	-5.99746200	0.76883300	0.03028200
C	-1.21270700	0.23566800	-0.02516900	H	-6.93021100	1.32448100	0.05197500
C	-4.07800100	0.74146100	0.11412000	C	-1.91777600	2.43854500	0.09563000
O	-4.26901100	-1.67178200	0.01461500	C	-1.66235800	2.99259900	1.35634900
C	-0.82245600	2.58760700	-0.12881200	C	-1.06814100	4.25515600	1.38749500
C	-0.32606200	3.01432400	-1.37584300	H	-0.84619500	4.71701000	2.34534900
C	0.50501400	4.13786200	-1.41411600	C	-0.74723600	4.92253100	0.21097400
H	0.87795500	4.49281400	-2.37169100	H	-0.28118500	5.90278700	0.25664200
C	0.87053600	4.79886800	-0.24756200	C	-1.01620900	4.34258800	-1.02346800
H	1.51075600	5.67608000	-0.29587700	H	-0.75632400	4.87311500	-1.93512600
C	0.43641200	4.31875000	0.98282500	C	-1.60862400	3.08141100	-1.10960400
H	0.75315400	4.81567600	1.89651000	C	-1.97469600	2.25799800	2.64309800
C	-0.40466000	3.20555200	1.06486900	H	-2.47624800	1.31724000	2.38957000
C	-0.63890400	2.27000800	-2.65852800	C	-2.93523500	3.05961700	3.52295700
H	-1.20219800	1.37108400	-2.39137500	H	-2.48846400	4.00771300	3.84245100
C	0.64465300	1.78963100	-3.33974400	H	-3.18688100	2.49028000	4.42451800
H	0.40302700	1.19478800	-4.22819600	H	-3.86290200	3.28601400	2.98696300
H	1.23107900	1.16140400	-2.65953000	C	-0.68720200	1.89838400	3.38867100
H	1.27565200	2.62756900	-3.65811100	H	-0.13836400	2.79824500	3.68860800
C	-1.48776500	3.11441500	-3.61080900	H	-0.02498500	1.29347200	2.75903700
H	-1.73619700	2.54454500	-4.51363300	H	-0.91809700	1.32616100	4.29429400
H	-0.95065600	4.01823100	-3.92247100	C	-1.87255800	2.44792200	-2.45932800
H	-2.42382800	3.42979800	-3.13728600	H	-2.34037900	1.47079300	-2.29489800
C	3.42497100	0.60529100	0.07885100	C	-2.85227100	3.28853800	-3.28038800
C	3.20210600	1.98484800	0.07515300	H	-3.79537400	3.43034600	-2.74218000
H	2.19433800	2.38742200	0.06457600	H	-3.06891300	2.79463500	-4.23406800
C	4.31834800	2.81714800	0.08473500	H	-2.43798100	4.27813900	-3.50377800
H	4.17867900	3.89341500	0.08142400	C	-0.56445600	2.20259800	-3.21472400
C	5.61002300	2.28421900	0.09807200	H	-0.04954900	3.14425200	-3.43608300
H	6.46337000	2.95541900	0.10568100	H	-0.76375700	1.69640900	-4.16588100
C	5.83233300	0.89620400	0.10154900	H	0.11626600	1.57543400	-2.62820000
H	6.84552500	0.50517800	0.11127600	C	-1.98733600	-2.41864500	-0.09322500
C	4.73707800	0.05425000	0.09157900	C	-1.75764700	-2.99242900	-1.34999500
N	-2.16295500	-0.78673400	-0.01243600	C	-1.20007500	-4.27172800	-1.37254800
C	-3.53218000	-0.67674300	0.02313400	H	-0.99886600	-4.74959000	-2.32711600
C	-3.10026900	1.71656400	-0.54872500	C	-0.88932600	-4.93601200	-0.19153000
C	-1.61171300	-2.11063500	0.00432300	H	-0.45186200	-5.92963400	-0.23048000
C	-1.32408300	-2.70265700	1.24284800	C	-1.13268800	-4.33644800	1.03879200
C	-0.81157400	-4.00225900	1.24257200	H	-0.88160300	-4.86512900	1.95396800
H	-0.59449400	-4.49237300	2.18777900	C	-1.68852600	-3.05820500	1.11639700
C	-0.58249200	-4.67895300	0.04879600	C	-2.05467800	-2.25931600	-2.64123100
H	-0.19188600	-5.69335400	0.06647400	H	-2.55260500	-1.31511200	-2.39371000
C	-0.84379400	-4.05664300	-1.16683400	C	-0.75720400	-1.91076600	-3.37519900
H	-0.65286300	-4.58830300	-2.09544100	H	-0.97468200	-1.34011400	-4.28516300
C	-1.60105500	-1.97366800	2.54038500	H	-0.21256100	-2.81630000	-3.66576900
H	-1.75364200	-0.91858600	2.29235500	H	-0.09620500	-1.30880000	-2.74141800
C	-0.41778100	-2.03552200	3.50494000	C	-3.01159400	-3.05481700	-3.53021000
H	-0.62434500	-1.42828900	4.39389600	H	-3.25364300	-2.48173300	-4.43198600
H	0.49182500	-1.64728000	3.03160400	H	-3.94448800	-3.27881800	-3.00242600
H	-0.21549000	-3.05776700	3.84539100	H	-2.56647700	-4.00356300	-3.84992700
C	-2.88880000	-2.49993000	3.17975600	C	-1.92645500	-2.40536000	2.46182800
H	-3.13452400	-1.92556000	4.08113600	H	-2.36943700	-1.41752100	2.29186200
H	-2.77911000	-3.55260700	3.46841300	C	-0.60745600	-2.18814700	3.20679500
H	-3.72352800	-2.42517500	2.47569800	H	-0.78708000	-1.66784000	4.15423100
C	-0.82621900	2.67604900	2.41807000	H	0.08603600	-1.58496400	2.61012500
H	-1.41681600	1.77571600	2.23553100	H	-0.11673100	-3.14124600	3.43403900
C	0.38899700	2.25920900	3.24889300	C	-2.92236800	-3.21229700	3.29710500
H	0.06719400	1.81730900	4.19925100	H	-3.11872800	-2.70452600	4.24789300
H	1.03785500	3.11274600	3.47925300	H	-2.53310400	-4.21070200	3.52631100
H	0.98624900	1.51285900	2.71273400	H	-3.87284700	-3.33346400	2.76689700
C	-1.70176600	3.67980700	3.16888300	C	3.14682700	1.08294900	-0.04353900

H	-2.03430900	3.26014000	4.12547600
H	-2.59057900	3.94188800	2.58475200
H	-1.15485900	4.60629500	3.38167200
C	-4.26989400	1.07670200	1.59780800
H	-4.96358700	0.36517300	2.05725300
H	-4.68121600	2.08759400	1.71062700
H	-3.32369200	1.03122500	2.14398500
C	-5.42020300	0.80704100	-0.60926400
H	-5.85579800	1.80848300	-0.51362900
H	-5.30652300	0.57925400	-1.67502700
H	-6.11090500	0.07608900	-0.18330300
C	-1.67038300	-2.09711500	-2.53655300
C	-0.45193400	-2.07717700	-3.45910800
H	0.39245900	-1.57360700	-2.97428700
H	-0.13264400	-3.08872800	-3.73645200
H	-0.68452600	-1.53811800	-4.38511900
C	-2.87983300	-2.76287900	-3.19674200
H	-3.74331800	-2.73576100	-2.52472400
H	-2.66868800	-3.81138700	-3.43988600
H	-3.14299700	-2.24760900	-4.12831400
H	-1.93358900	-1.05558200	-2.32731900
C	-1.36058400	-2.75887600	-1.21168300
H	-3.15763200	1.58008600	-1.64262500
H	-3.39699600	2.74918300	-0.33566600
Cu	0.62091800	-0.11136700	0.02960100

C	2.77393200	2.43295400	-0.09312200
H	1.72521900	2.72030300	-0.10272400
C	3.77248300	3.38648300	-0.12982600
H	3.51669200	4.44062400	-0.16849400
C	5.13749600	3.01317700	-0.11831100
C	5.51454600	1.66669500	-0.06840300
H	6.56649800	1.39742000	-0.06124900
C	4.51887500	0.69864900	-0.02997200
C	4.51824400	-0.74289500	0.02407100
C	5.51317500	-1.71163000	0.06313100
H	6.56531600	-1.44308800	0.05659900
C	5.13515100	-3.05783700	0.11287100
C	3.76988700	-3.43018300	0.12354200
H	3.51355800	-4.48418200	0.16208000
C	2.77205700	-2.47587600	0.08615800
H	1.72298600	-2.76191000	0.09488200
C	3.14580900	-1.12617300	0.03678900
C	6.13839700	4.02766300	-0.15895500
C	6.13534500	-4.07299800	0.15417500
N	6.95056100	4.86016800	-0.19258900
N	6.94702000	-4.90595400	0.18836300

Optimized **Au-1**^{2CN} S_1 semi-coplanar

Au	-0.35208400	0.17878900	0.01965700
N	2.60780700	0.85823500	-0.06351200
N	2.24778600	-1.27523000	0.07817300
N	5.05620100	0.84229200	-0.12715600
N	4.56293100	-2.07278600	0.06529400
N	-2.42473500	0.26727500	0.00801600
C	1.61992600	-0.06950500	0.01834400
C	3.85489000	0.25402700	-0.05794400
C	3.62309200	-1.11958400	0.03278300
C	5.80120500	-1.48329400	-0.00539700
H	6.64839300	-2.16390100	0.01249000
C	6.03101000	-0.12409100	-0.09453600
H	7.05464400	0.23778100	-0.14491200
C	2.34016700	2.25527200	-0.14640700
C	2.16924100	2.82748500	-1.41288300
C	1.86751200	4.18969300	-1.46830200
H	1.73347400	4.66869400	-2.43463900
C	1.74414900	4.94025500	-0.30430000
H	1.51501500	6.00082200	-0.36671400
C	1.92170600	4.34313000	0.93914300
H	1.83186200	4.94237500	1.84131600
C	2.22307600	2.98377700	1.04400400
C	2.32053300	2.01317800	-2.67995000
H	2.47924900	0.96933100	-2.39116700
C	3.55707100	2.46243900	-3.46120000
H	3.46502300	3.50598600	-3.78511300
H	3.69107400	1.84186200	-4.35462600
H	4.45449000	2.37380900	-2.84052400
C	1.05309700	2.05704800	-3.53405500
H	0.83031700	3.07365900	-3.87857200
H	0.18922500	1.69215600	-2.96587100
H	1.17096600	1.42311100	-4.42038600
C	2.44209200	2.33643000	2.39482800
H	2.52996600	1.25627700	2.23918000
C	3.76004100	2.81873500	3.00560400
H	4.59628000	2.59699400	2.33464400
H	3.94277200	2.32018800	3.96451200
H	3.73971000	3.90052900	3.18393500

Optimized **Au-1**^{2CN} S_1 orthogonal

Au	-0.40938200	-0.00144300	-0.00112700
N	2.40402600	-0.22727500	-1.06041800
N	2.40221200	0.23478800	1.06061100
N	4.81753500	-0.30462100	-1.44393400
N	4.81540000	0.31733000	1.44655800
N	-2.47497900	-0.00032000	-0.00214800
C	1.57509500	0.00216100	-0.00032900
C	3.73016900	-0.14224900	-0.68277400
C	3.72901500	0.15371100	0.68423100
C	5.94165900	0.15212300	0.67593900
H	6.88868200	0.26490700	1.19672400
C	5.94263000	-0.13750300	-0.67213300
H	6.89044000	-0.24887000	-1.19180200
C	1.92537900	-0.51518400	-2.37105300
C	1.69487300	-1.85207700	-2.72007500
C	1.21895600	-2.10873900	-4.00721200
H	1.03735800	-3.13440100	-4.31654200
C	0.98288100	-1.06948900	-4.90024100
H	0.61723200	-1.28867400	-5.89990700
C	1.21804000	0.24801500	-4.52356900
H	1.03582800	1.05048900	-5.23322300
C	1.69424000	0.55293000	-3.24707400
C	1.98822000	-2.98134300	-1.75547800
H	2.15124500	-2.54101000	-0.76614000
C	3.28034300	-3.69724600	-2.15784500
H	3.17934700	-4.15903600	-3.14745900
H	3.52203000	-4.48703800	-1.43696200
H	4.11550800	-2.99035300	-2.19238600
C	0.81533200	-3.95274900	-1.62861100
H	0.62207100	-4.48718200	-2.56579300
H	-0.10237200	-3.42296700	-1.34724600
H	1.02975600	-4.70379800	-0.85967700
C	1.98713500	1.98212400	-2.84307300
H	2.16716800	1.99300400	-1.76295400
C	3.26659800	2.47139800	-3.52675600
H	4.10752300	1.81595000	-3.27794600
H	3.51037900	3.48962400	-3.20185500
H	3.14877900	2.48132200	-4.61706200

C	1.25778700	2.56007200	3.33492900	C	0.80465400	2.91211000	-3.11177300
H	1.11561800	3.62178000	3.56766800	H	0.58509800	2.99764600	-4.18215800
H	1.42218200	2.03277600	4.28150700	H	1.02494600	3.91906100	-2.73906100
H	0.33015300	2.18085000	2.89023400	H	-0.09970100	2.54875300	-2.60951100
C	1.52252000	-2.50010300	0.15716200	C	1.92006700	0.51219700	2.37237100
C	1.17555400	-2.99165900	1.42204200	C	1.68639700	1.84601000	2.73158800
C	0.40709000	-4.15685500	1.47302400	C	1.20557000	2.09119100	4.01907400
H	0.12178700	-4.56689300	2.43815600	H	1.02135800	3.11376200	4.33661500
C	0.00881400	-4.80054100	0.30627000	C	0.96778700	1.04416200	4.90267400
H	-0.58228100	-5.71082700	0.36555500	H	0.59809500	1.25467000	5.90272200
C	0.37267300	-4.29057100	-0.93594100	C	1.20656600	-0.26972800	4.51622900
H	0.06281400	-4.80576800	-1.84129100	H	1.02325400	-1.07838600	5.21857000
C	1.13803500	-3.12623900	-1.03598500	C	1.68781500	-0.56334200	3.23890000
C	1.61648900	-2.29761400	2.69271000	C	1.98811800	2.98235100	1.77791300
H	2.15933900	-1.39067100	2.40830100	H	2.12279600	2.55226500	0.77958500
C	0.41822400	-1.86396400	3.53803500	C	0.84009400	3.98645900	1.68514300
H	0.75736800	-1.32323400	4.42899500	H	1.06104900	4.73975400	0.92033900
H	-0.17275100	-2.72421800	3.87390700	H	0.68082700	4.51673600	2.63100200
H	-0.23993400	-1.19989600	2.96519900	H	-0.09822800	3.48711600	1.41642200
C	2.58636200	-3.18074900	3.48009700	C	3.30507100	3.65904000	2.16882400
H	2.93757900	-2.65629100	4.37595600	H	3.55681700	4.45113600	1.45389600
H	3.45611700	-3.43660600	2.86651000	H	4.12176800	2.93010200	2.18347400
H	2.10663100	-4.11288300	3.80129300	H	3.22934700	4.11097800	3.16527300
C	1.54366500	-2.57790900	-2.38716800	C	1.98402100	-1.98905000	2.82503800
H	2.04277300	-1.61768800	-2.22293200	H	2.17351400	-1.99100600	1.74655900
C	0.32969500	-2.31079100	-3.27739800	C	0.79860900	-2.92044600	3.07543600
H	0.64728600	-1.86492700	-4.22677600	H	1.02083900	-3.92417300	2.69520700
H	-0.36477600	-1.61582500	-2.79037200	H	-0.10104500	-2.55123100	2.56906800
H	-0.21590100	-3.23293500	-3.50942100	H	0.57010700	-3.01530800	4.14317800
C	2.55666100	-3.50828300	-3.05790400	C	3.25720800	-2.48495600	3.51549600
H	2.88032200	-3.09110500	-4.01829800	H	3.50340700	-3.50038400	3.18376900
H	2.12284100	-4.49740500	-3.24728700	H	3.13026200	-2.50438000	4.60463700
H	3.43820500	-3.63545400	-2.42129700	H	4.10057800	-1.82780300	3.27969000
C	-3.23145700	1.36416700	0.05959700	C	-3.28650300	-1.08168700	0.20511500
C	-2.83200500	2.70534000	0.12942100	C	-2.88982000	-2.40007300	0.45636300
H	-1.77890800	2.96863700	0.14645100	H	-1.83741300	-2.65820500	0.50753800
C	-3.82425500	3.67298400	0.17246900	C	-3.88203200	-3.35292500	0.63424100
H	-3.56192500	4.72337800	0.22623500	H	-3.61941400	-4.38621600	0.82976800
C	-5.18154600	3.30423700	0.14622700	C	-5.23814300	-2.98971100	0.56297800
C	-5.58655200	1.94915200	0.07427800	C	-5.64155800	-1.65644600	0.31072900
H	-6.64301100	1.70329400	0.05612400	H	-6.69743100	-1.41243100	0.26327500
C	-4.60771100	0.98520800	0.03023300	C	-4.66058800	-0.71011100	0.13370500
C	-4.60179800	-0.46953600	-0.04685700	C	-4.65668400	0.72136200	-0.13837700
C	-5.57153100	-1.44185600	-0.10724400	C	-5.63228400	1.67320000	-0.31551400
H	-6.63028200	-1.20547200	-0.10566200	H	-6.68948000	1.43511100	-0.26822700
C	-5.15342600	-2.79388600	-0.17387500	C	-5.22139300	3.00422400	-0.56761800
C	-3.79304800	-3.15056100	-0.17912500	C	-3.86327300	3.35991600	-0.63874600
H	-3.51690600	-4.19768100	-0.22928400	H	-3.59489000	4.39172700	-0.83428300
C	-2.80937500	-2.17507000	-0.12006800	C	-2.87643900	2.40150400	-0.46074800
H	-1.75534500	-2.43693000	-0.12412800	H	-1.82242700	2.65308300	-0.51191900
C	-3.22314200	-0.83711000	-0.05535100	C	-3.28067000	1.08538800	-0.20956200
C	-6.18140400	4.31912300	0.19175900	C	-6.21710000	4.00792400	-0.75373200
C	-6.14373000	-3.81722400	-0.23660900	C	-6.23935700	-3.98792500	0.74903500
N	-6.99982100	5.14359300	0.22862800	N	-7.03188600	4.82253300	-0.90464500
N	-6.95376600	-4.64917500	-0.28781200	N	-7.05859200	-4.79806800	0.89989700
Optimized **Au-1^{2CN}** T_1 semi-coplanar				Optimized **Au-1^{CN}** S_0 semi-coplanar			
Au	-0.34909900	0.16206100	0.00898000	Au	0.42647800	0.32652300	-0.00382400
N	2.26544200	-1.26697800	0.05034800	N	-1.83018000	-1.61314000	-0.01164400
N	2.59838900	0.87714800	-0.05131700	N	-2.56992100	0.44484000	0.04833900
N	4.58807800	-2.02771800	0.05437600	N	-3.98928100	-2.75550900	-0.01009000
N	5.04202400	0.89275500	-0.09316100	N	-4.96170700	-0.05083600	0.06636000
N	-2.39790700	0.24833100	0.00248600	N	2.35530300	0.92329900	-0.02118300
C	1.61574000	-0.06598800	0.00563900	C	-1.44206300	-0.31094400	0.01319300
C	3.63382600	-1.09196500	0.02347900	C	-3.20910300	-1.69418100	0.00584900

C	3.84866400	0.29171800	-0.04463000	C	-3.68451600	-0.37221200	0.04312000
C	6.03123400	-0.05912900	-0.06396300	C	-5.76607100	-1.12214700	0.05077400
C	5.81952000	-1.42195300	0.00543600	H	-6.83468200	-0.92966600	0.06814800
C	1.55561400	-2.50127500	0.12116400	C	-5.29156200	-2.44184500	0.01352600
C	1.22240100	-3.00843100	1.38397400	H	-5.99285300	-3.27093500	0.00227600
C	0.46956800	-4.18388300	1.42914300	C	-0.89234000	-2.69582400	-0.06532000
C	0.06814400	-4.81981600	0.25899800	C	-0.48573500	-3.15493500	-1.32459000
C	0.41369300	-4.29170800	-0.98060400	C	0.48169100	-4.16051200	-1.35338900
C	1.16667300	-3.11864300	-1.07473600	H	0.83031000	-4.53847400	-2.31021600
C	1.66734100	-2.32196800	2.65734000	C	1.01285300	-4.67447800	-0.17602900
C	2.70513100	-3.17522200	3.38977600	H	1.76882100	-5.45345000	-0.21995200
C	0.47939400	-1.96594800	3.55152500	C	0.58688800	-4.19414800	1.05690600
C	1.53808700	-2.54390300	-2.42471000	H	1.01528400	-4.59955200	1.96923000
C	2.46487400	-3.49476800	-3.18420700	C	-0.37779400	-3.18840600	1.14056600
C	0.29204200	-2.18625700	-3.23651800	C	-1.03016000	-2.57492300	-2.61307100
C	2.31028500	2.27098200	-0.11396300	H	-1.79928800	-1.83645800	-2.35995400
C	2.15482400	2.86375000	-1.37318700	C	-1.70102200	-3.65098400	-3.46847400
C	1.82876000	4.22089500	-1.41010400	H	-0.98256800	-4.41445100	-3.78739600
C	1.66383000	4.94677200	-0.23546300	H	-2.13213000	-3.20302000	-4.37064100
C	1.82435700	4.32931900	1.00017300	H	-2.50233700	-4.15050800	-2.91386100
C	2.15200600	2.97458000	1.08646900	C	0.06859700	-1.83877000	-3.38367200
C	2.34710100	2.07578500	-2.65133500	H	0.86831600	-2.52427000	-3.68616900
C	1.10409800	2.12854300	-3.53993000	H	0.51605200	-1.04857300	-2.77040600
C	3.60185200	2.54959100	-3.38797700	H	-0.34334800	-1.38001300	-4.28969900
C	2.33686500	2.30603500	2.43207900	C	-0.81201000	-2.65255300	2.48844700
C	1.06858300	2.39635900	3.28174800	H	-1.56840500	-1.87741100	2.32254800
C	3.55580300	2.87995300	3.15696800	C	-1.46226200	-3.74931200	3.33391900
C	-3.20158000	-0.85870600	-0.04939700	H	-2.31867500	-4.19112900	2.81360500
C	-2.78907400	-2.19553100	-0.10652600	H	-1.81163500	-3.33685100	4.28697700
C	-3.77437500	-3.17072200	-0.15466100	H	-0.75178100	-4.55278900	3.55851800
C	-5.13395400	-2.81220600	-0.14769100	C	0.35993200	-1.99219300	3.21811800
C	-5.54962200	-1.45999300	-0.09047400	H	1.15172400	-2.71688900	3.43872100
C	-4.57683400	-0.48891700	-0.03990300	H	0.02279700	-1.56229200	4.16801200
C	-4.58189700	0.96741200	0.02519900	H	0.79612300	-1.18932800	2.61325100
C	-5.56299400	1.93015000	0.06677600	C	-2.54029800	1.87715700	0.07950300
C	-5.15950300	3.28554200	0.12681100	C	-2.50879200	2.51059300	1.32806800
C	-3.80273700	3.65580900	0.14477100	C	-2.40498800	3.90231100	1.33248700
C	-2.80934100	2.68860400	0.10544100	H	-2.36811000	4.43194100	2.28018100
C	-3.20814200	1.34793800	0.04642700	C	-2.33617200	4.61670800	0.14196100
C	-6.12568600	-3.83552900	-0.19989700	H	-2.25057300	5.69941100	0.16684900
C	-6.16008600	4.30043700	0.16963500	C	-2.37352900	3.95500100	-1.07985900
N	-6.93630600	-4.66720900	-0.24243300	H	-2.31479300	4.52541900	-2.00251400
N	-6.97860000	5.12475200	0.20435400	C	-2.47581500	2.56410300	-1.13910600
H	7.04969200	0.31859200	-0.09927400	C	-2.54124100	1.73564900	2.62859500
H	6.67500500	-2.09198100	0.02433700	H	-2.71798500	0.67989300	2.39428800
H	0.19816200	-4.60788700	2.39219100	C	-1.18994600	1.82433800	3.34277300
H	-0.51191400	-5.73743600	0.31384500	H	-1.20206600	1.22326700	4.25913000
H	0.09876800	-4.79867000	-1.88898300	H	-0.95758200	2.85906200	3.61945100
H	2.15501600	-1.38256700	2.37842900	H	-0.38074900	1.45803200	2.70099500
H	2.28136000	-4.13855500	3.69770600	C	-3.68857300	2.19504900	3.52924400
H	3.05831400	-2.65822600	4.28927300	H	-3.72404400	1.58243600	4.43683800
H	3.56607800	-3.37022100	2.74223700	H	-4.65160700	2.10725500	3.01548400
H	-0.05455300	-2.85998400	3.89425500	H	-3.56321900	3.23852700	3.83900700
H	-0.23208300	-1.32708700	3.01498200	C	-2.49163600	1.85064000	-2.47456500
H	0.82313500	-1.42300400	4.43932300	H	-2.59083000	0.77541500	-2.28811700
H	2.09195800	-1.61557000	-2.25264000	C	-1.17401700	2.06040900	-3.22406700
H	3.36875100	-3.69812600	-2.60095600	H	-1.17820700	1.49863800	-4.16492300
H	2.76372300	-3.05165300	-4.14092100	H	-0.32279600	1.71943500	-2.62420100
H	1.97111200	-4.45063500	-3.39557700	H	-1.01646400	3.11806200	-3.46405500
H	-0.31215100	-3.07331200	-3.46094300	C	-3.69669700	2.27814100	-3.31454400
H	0.57704700	-1.72548200	-4.18922500	H	-3.72022500	1.71817400	-4.25602400
H	-0.33587500	-1.47436200	-2.68729100	H	-3.65216100	3.34468700	-3.56211900
H	1.70718100	4.71548400	-2.37020100	H	-4.63420800	2.09413000	-2.77924200
H	1.41486300	6.00361800	-0.28345800	C	3.44303300	0.09522700	0.02312200
H	1.69967300	4.90815500	1.91164700	C	3.46765400	-1.30574600	0.08906400

H	2.50414200	1.02722100	-2.37877600
H	1.25051400	1.51082600	-4.43338900
H	0.88540400	3.14968400	-3.87339200
H	0.22673600	1.74933100	-3.00282300
H	3.76610900	1.94832100	-4.28954300
H	4.48202500	2.45552900	-2.74383700
H	3.51005300	3.59880400	-3.69330700
H	2.53224300	1.24353900	2.25566600
H	1.20868800	1.86743900	4.23146800
H	0.21942700	1.93990900	2.75934400
H	0.80921200	3.43611600	3.51347800
H	3.71171400	2.36257100	4.11048900
H	3.42672800	3.94780200	3.36969200
H	4.45677500	2.75917400	2.54695300
H	-1.73496800	-2.45688700	-0.11490100
H	-3.49964500	-4.21846700	-0.19843100
H	-6.60772300	-1.22104700	-0.08877400
H	-6.61894200	1.68198200	0.05618800
H	-3.54107600	4.70676900	0.19011700
H	-1.75660400	2.95363600	0.11846200

H	2.54201500	-1.87641500	0.11004500
C	4.69343500	-1.94051200	0.12639900
H	4.74392800	-3.02377600	0.17721300
C	5.89870000	-1.19834700	0.09873300
C	5.87948600	0.20040100	0.03263600
H	6.81364000	0.75422600	0.01229300
C	4.65346300	0.85179000	-0.00565900
C	4.24979500	2.23493800	-0.07332800
C	4.93445900	3.45151900	-0.12918900
H	6.02151600	3.47487500	-0.12539200
C	4.20463700	4.63120400	-0.19042300
C	2.79931700	4.60549700	-0.19578300
H	2.25029600	5.54247300	-0.24380200
C	2.09676900	3.40958000	-0.14072200
H	1.00873800	3.39051900	-0.14475700
C	2.82944000	2.22011800	-0.07962800
C	7.14460400	-1.88805300	0.13866000
N	8.15988800	-2.45667400	0.17129500
H	4.72276400	5.58508000	-0.23450600

Optimized **Au-1^CN^** S_1 semi-coplanar

Au	0.43408200	0.44040700	0.01444600
N	-1.69797500	-1.64431200	-0.01624500
N	-2.60165400	0.32488600	-0.00587600
N	-3.72865200	-3.01666500	-0.06241200
N	-4.96515400	-0.32189900	-0.04727200
N	2.41037200	1.07904300	0.01491700
C	-1.40760800	-0.31730700	0.00187400
C	-3.06942300	-1.84994100	-0.03750000
C	-3.65058200	-0.58330300	-0.03048600
C	-5.65423100	-1.50851200	-0.07342800
H	-6.73783000	-1.42363500	-0.08906300
C	-5.07809500	-2.76481900	-0.08028200
H	-5.72068800	-3.64128900	-0.10172700
C	-0.67863600	-2.64163200	-0.00449800
C	-0.14749600	-3.07049800	-1.22773700
C	0.89567200	-3.99846400	-1.18826500
H	1.32894900	-4.35484000	-2.11901700
C	1.38226700	-4.47384000	0.02496800
H	2.19134500	-5.19963700	0.03644500
C	0.83228200	-4.03104800	1.22332900
H	1.21697300	-4.41209400	2.16561400
C	-0.21239900	-3.10418500	1.23287700
C	-0.68138100	-2.56112800	-2.54918400
H	-1.43794100	-1.79989000	-2.33375400
C	-1.37424300	-3.68811700	-3.31788900
H	-0.67003000	-4.49039800	-3.56840100
H	-1.79880100	-3.30699800	-4.25365300
H	-2.18469300	-4.11764300	-2.72024700
C	0.41511400	-1.89071100	-3.37789100
H	1.20646200	-2.59843800	-3.65165000
H	0.87361500	-1.06494900	-2.82111700
H	-0.00484300	-1.48501700	-4.30540800
C	-0.81159800	-2.62593600	2.53801700
H	-1.57488100	-1.87748700	2.30272200
C	-1.51203200	-3.77607900	3.26397600
H	-2.29016100	-4.21213200	2.62923200
H	-1.98054500	-3.41512300	4.18663300
H	-0.80368800	-4.56858600	3.53310500
C	0.23741000	-1.94458100	3.41803700
H	1.03422100	-2.63920400	3.70913900
H	-0.22564300	-1.56388700	4.33562700
H	0.69680300	-1.09964400	2.89163700
C	-2.70858100	1.74594200	0.00499500

Optimized **Au-1^CN^** S_1 orthogonal

Au	-0.57483400	-0.00009800	0.23042100
N	2.20112100	-1.08459200	-0.22706300
N	2.20113800	1.08473700	-0.22614200
N	4.58528500	-1.47915900	-0.60768700
N	4.58530700	1.47960000	-0.60638800
N	-2.61054300	-0.00026500	0.57777900
C	1.38370900	0.00002500	-0.09641800
C	3.51189100	-0.69856300	-0.43606900
C	3.51190400	0.69886900	-0.43545100
C	5.69637400	0.68962600	-0.78501900
H	6.63229400	1.22116500	-0.93507600
C	5.69636400	-0.68904400	-0.78562400
H	6.63227900	-1.22046700	-0.93612500
C	1.72668300	-2.42639800	-0.16114800
C	1.68653500	-3.06141700	1.08683600
C	1.21400600	-4.37442300	1.12660800
H	1.17730100	-4.90231200	2.07556800
C	0.79802600	-5.01749600	-0.03387800
H	0.43773100	-6.04164500	0.01538700
C	0.84514000	-4.35945000	-1.25759600
H	0.52200100	-4.87510600	-2.15780700
C	1.31003500	-3.04597700	-1.34619000
C	2.17870600	-2.36731400	2.33894200
H	2.28085900	-1.30124500	2.10962300
C	3.56613100	-2.89208700	2.71895900
H	3.52447300	-3.96026600	2.96440700
H	3.95317800	-2.35665700	3.59388200
H	4.26768700	-2.75779500	1.88929400
C	1.18877600	-2.48464100	3.49692800
H	1.07059500	-3.52115900	3.83304000
H	0.20059100	-2.10869000	3.20725100
H	1.54239200	-1.90005500	4.35403300
C	1.39621800	-2.33586000	-2.68041600
H	1.59493900	-1.27742000	-2.48195000
C	2.57383400	-2.88061800	-3.49309100
H	3.50854100	-2.77370900	-2.93327100
H	2.67063800	-2.33708000	-4.44022200
H	2.43076600	-3.94320300	-3.72407600
C	0.08395100	-2.40860000	-3.46010100
H	-0.16568900	-3.43628000	-3.74798500
H	0.15966600	-1.81805100	-4.38038100
H	-0.74718500	-2.01341400	-2.86447100
C	1.72676000	2.42650300	-0.15902500

C	-2.72564700	2.40502700	1.24028400
C	-2.77871600	3.80027000	1.22466800
H	-2.79562300	4.34482900	2.16515300
C	-2.81580800	4.49916100	0.02308700
H	-2.86138000	5.58503200	0.03032200
C	-2.80389800	3.81519300	-1.18759500
H	-2.83956000	4.37127200	-2.12079100
C	-2.75063000	2.42040000	-1.22126600
C	-2.69350900	1.64562100	2.54950300
H	-2.62613200	0.57799500	2.31764500
C	-1.45822200	2.00896400	3.37483300
H	-1.42853200	1.41703800	4.29689300
H	-1.45866300	3.06856000	3.65673000
H	-0.54100600	1.80650900	2.80928200
C	-3.99005900	1.85969500	3.33267600
H	-3.97922500	1.27007000	4.25646100
H	-4.85438500	1.55078400	2.73599700
H	-4.12203400	2.91300400	3.60717500
C	-2.73824900	1.67641500	-2.53954500
H	-2.67822700	0.60562500	-2.32076500
C	-1.50657000	2.03942900	-3.37060700
H	-1.49006100	1.45964100	-4.30070700
H	-0.58638800	1.82149600	-2.81568800
H	-1.49998000	3.10271800	-3.63823200
C	-4.03974900	1.90969500	-3.30860300
H	-4.04094000	1.33144000	-4.23958700
H	-4.16681100	2.96713000	-3.56925700
H	-4.90036300	1.59860900	-2.70784000
C	3.48096700	0.22434200	0.00836500
C	3.44503200	-1.17331100	0.00623900
H	2.50072000	-1.71003300	0.01043600
C	4.65744200	-1.84993900	-0.00240100
H	4.67358900	-2.93408300	-0.00466000
C	5.87109100	-1.14079900	-0.00866900
C	5.90516600	0.27324200	-0.00650500
H	6.85971500	0.78910900	-0.01202400
C	4.70676800	0.95102800	0.00200400
C	4.32044600	2.35473200	0.00522400
C	4.99685500	3.55587200	0.00145400
H	6.08166600	3.59624600	-0.00467200
C	4.24673400	4.74804400	0.00518900
C	2.84667500	4.73759400	0.01255900
H	2.30579800	5.67780900	0.01505500
C	2.14429300	3.54004600	0.01662800
H	1.05881600	3.50756300	0.02237800
C	2.89050200	2.34851700	0.01294900
C	7.10212200	-1.86091000	-0.01801800
N	8.10742500	-2.44435200	-0.02581200
H	4.77051100	5.69840400	0.00203200

C	1.31019600	3.04718300	-1.34352400
C	0.84541100	4.36061400	-1.25375700
H	0.52236000	4.87712500	-2.15350800
C	0.79830600	5.01755400	-0.02944200
H	0.43810000	6.04169000	0.02073900
C	1.21420700	4.37340300	1.13047400
H	1.17752400	4.90043900	2.07991100
C	1.68665300	3.06040400	1.08952600
C	1.39639700	2.33825200	-2.67838200
H	1.59474300	1.27956800	-2.48083300
C	0.08430400	2.41210400	-3.45824900
H	0.16001400	1.82234100	-4.37903200
H	-0.16495900	3.44011200	-3.74528700
H	-0.74707400	2.01665700	-2.86313000
C	2.57435300	2.88336900	-3.49032400
H	2.67115900	2.34067500	-4.43793900
H	3.50891000	2.77562300	-2.93040900
H	2.43168500	3.94621700	-3.72034800
C	2.17881200	2.36518900	2.34102000
H	2.28128800	1.29937000	2.11069000
C	1.18864500	2.48114500	3.49894600
H	1.54229200	1.89589700	4.35558800
H	0.20063500	2.10513900	3.20874600
H	1.07006900	3.51732500	3.83596100
C	3.56602700	2.88996300	2.72178100
H	3.95309300	2.35375500	3.59621800
H	3.52404500	3.95788200	2.96829900
H	4.26775000	2.75670600	1.89208900
C	-3.22216800	-0.00095600	1.79428300
C	-2.60467300	-0.00167200	3.05576800
H	-1.52253500	-0.00172200	3.13406100
C	-3.42540700	-0.00230000	4.17553200
H	-2.98352600	-0.00286500	5.16597200
C	-4.81851200	-0.00222100	4.04195800
C	-5.44143100	-0.00150400	2.77869700
H	-6.52435100	-0.00146500	2.70492700
C	-4.64287200	-0.00086800	1.65606500
C	-4.88635200	-0.00005000	0.22010200
C	-6.01543800	0.00041000	-0.56703900
H	-7.01397600	0.00019200	-0.14312200
C	-5.84628500	0.00119200	-1.97029600
C	-4.57135600	0.00150200	-2.55890600
H	-4.48548900	0.00210300	-3.63957200
C	-3.42986500	0.00105000	-1.76641000
H	-2.43691000	0.00129200	-2.20354200
C	-3.59734600	0.00027700	-0.38129900
C	-7.00353500	0.00167600	-2.80528300
N	-7.94906400	0.00206800	-3.48064000
H	-5.43741700	-0.00273100	4.93322600

Optimized **Au-1**$^{\mathbf{CN}}$ T1 semi-coplanar

Au	0.45409300	0.44855300	0.00071600
N	-1.67467400	-1.63596300	-0.02032400
N	-2.58099100	0.33598000	-0.01993000
N	-3.70190700	-3.00466000	-0.05095200
N	-4.93893800	-0.31509400	-0.04985000
N	2.40878100	1.08123200	0.00428900
C	-1.37873500	-0.30375300	-0.00981300
C	-3.04062000	-1.84150000	-0.03748600
C	-3.62522500	-0.56977400	-0.03738600
C	-5.62986100	-1.50260300	-0.06492200
H	-6.71321400	-1.41629700	-0.07651100
C	-5.05348100	-2.75640200	-0.06538400
H	-5.69322300	-3.63491900	-0.07770900
C	-0.65493000	-2.63207500	-0.00232300

Optimized **Au-1**$^{\mathbf{2tBu}}$ S_0 semi-coplanar

N	-1.76015900	-0.00152400	0.00111700
N	3.02935300	1.09351700	-0.05460800
N	3.03186400	-1.09123400	0.05404000
N	5.44688300	1.43976500	-0.07241700
N	5.45020600	-1.43202900	0.06809700
C	-2.58175400	1.10562600	0.02509400
C	-2.23360900	2.46016400	0.05529100
H	-1.18878600	2.76600800	0.06003300
C	-3.25201300	3.39909200	0.08011200
H	-2.97815900	4.45125800	0.10460100
C	-4.62135900	3.04514700	0.07568600
C	-4.95167500	1.69286300	0.04344000
H	-5.99106900	1.37845600	0.03862400
C	-3.94707800	0.72007200	0.01751400

C	-0.14587300	-3.09100300	-1.22418500	C	-3.94740600	-0.72205600	-0.01607800
C	0.88983900	-4.02714600	-1.18111400	C	-4.95246400	-1.69435000	-0.04242000
H	1.30340000	-4.41020500	-2.11006200	H	-5.99170300	-1.37942700	-0.03797000
C	1.39286400	-4.47881300	0.03449900	C	-4.62279900	-3.04679400	-0.07463600
H	2.19475300	-5.21254200	0.04897800	C	-3.25362500	-3.40140500	-0.07858800
C	0.86901300	-4.00229500	1.23168700	H	-2.98030000	-4.45370800	-0.10304400
H	1.26627300	-4.36599500	2.17545000	C	-2.23476600	-2.46297100	-0.05334200
C	-0.16741200	-3.06602100	1.23762000	H	-1.19007800	-2.76930100	-0.05769800
C	-0.71021500	-2.61352700	-2.54466800	C	-2.58225400	-1.10826700	-0.02319000
H	-1.38728300	-1.77916900	-2.33531700	C	2.22220100	0.00023400	0.00025200
C	-1.53899800	-3.72347500	-3.19598200	C	4.35453600	0.70480400	-0.03575300
H	-0.91622400	-4.59597700	-3.42753700	C	4.35615600	-0.69953000	0.03315000
H	-1.98672800	-3.36818700	-4.13126600	C	6.56774100	0.70524600	-0.03723300
H	-2.34346300	-4.04319100	-2.52566300	H	7.50744800	1.24893400	-0.06440400
C	0.38019800	-2.08622700	-3.47671400	C	6.56936100	-0.69500600	0.03114800
H	1.08799700	-2.87209400	-3.76475400	H	7.51032400	-1.23659500	0.05682700
H	0.94424400	-1.27716200	-2.99785800	C	2.51537300	2.42877200	-0.12572400
H	-0.06785100	-1.69203900	-4.39586600	C	2.26368800	2.97393700	-1.39083800
C	-0.75338100	-2.56267400	2.53916100	C	1.69927200	4.24965300	-1.43345900
H	-1.43678000	-1.74128200	2.30189100	H	1.48075200	4.70462700	-2.39546200
C	-1.57778700	-3.66472200	3.20895800	C	1.40122400	4.93777300	-0.26305100
H	-2.37079700	-4.01084800	2.53805700	H	0.95649400	5.92749200	-0.31732900
H	-2.04069600	-3.29036700	4.12928300	C	1.66293200	4.36535000	0.97629700
H	-0.94912400	-4.52434800	3.47058600	H	1.41756200	4.91042600	1.88342000
C	0.32001800	-1.99958900	3.47012200	C	2.22651100	3.09202100	1.07330000
H	1.03489300	-2.76920700	3.78363000	C	2.54764300	2.21558600	-2.67029200
H	-0.14287900	-1.58952900	4.37492100	H	3.02646200	1.26562200	-2.40753200
H	0.87821900	-1.19447300	2.97796800	C	1.24540900	1.87962400	-3.40116500
C	-2.68790200	1.75647400	0.00354800	H	0.71885900	2.78957700	-3.71066000
C	-2.73482400	2.40032500	1.24631700	H	0.57201700	1.30304100	-2.75725800
C	-2.80234600	3.79500200	1.24693200	H	1.45442600	1.28783600	-4.29967000
H	-2.84734600	4.32775200	2.19316400	C	3.52168100	2.98074100	-3.56751800
C	-2.81995300	4.50818000	0.05335500	H	3.75144500	2.39398100	-4.46386300
H	-2.87851500	5.59331100	0.07279800	H	4.45924500	3.18995800	-3.04163900
C	-2.77162600	3.83994200	-1.16528400	H	3.09674900	3.93603300	-3.89558600
H	-2.79380300	4.40788600	-2.09153500	C	2.47710500	2.46409300	2.42809200
C	-2.70324400	2.44621900	-1.21516200	H	2.93448400	1.48071200	2.27127800
C	-2.73767400	1.62097200	2.54407400	C	3.46132700	3.29729300	3.25097200
H	-2.56335400	0.56755400	2.30278400	H	4.40932100	3.42604900	2.71793100
C	-1.60838000	2.06187300	3.47511800	H	3.66675000	2.80538600	4.20826500
H	-1.60212300	1.44466500	4.38083300	H	3.05713400	4.29257500	3.46789100
H	-1.72104800	3.10698400	3.78625100	C	1.16105700	2.23737700	3.17561400
H	-0.63470600	1.95514900	2.98271900	H	0.47794800	1.61521300	2.58687200
C	-4.10903600	1.71363900	3.21706100	H	0.65543600	3.18633500	3.38732700
H	-4.12683000	1.11379500	4.13429300	H	1.34803500	1.73437300	4.13114900
H	-4.88970100	1.34274800	2.54499200	C	2.52092400	-2.42767000	0.12500300
H	-4.34935900	2.74957300	3.48483400	C	2.23099500	-3.09039300	-1.07405900
C	-2.67969000	1.71340000	-2.53935600	C	2.47818900	-2.46106000	-2.42883000
H	-2.47416000	0.65798800	-2.33324700	C	1.16058000	-2.23643900	-3.17423000
C	-1.56475800	2.21785000	-3.45489100	C	3.46272400	-3.29184800	-3.25377900
H	-1.53889000	1.62787400	-4.37815500	H	2.93394200	-1.47689700	-2.27213300
H	-0.58798000	2.12818000	-2.96520400	H	0.47711300	-1.61602500	-2.58406400
H	-1.71047100	3.26710600	-3.73687100	H	1.34514800	-1.73241400	-4.12969500
C	-4.05362900	1.79056000	-3.20971200	H	0.65654400	-3.18625700	-3.38586500
H	-4.05608100	1.22052100	-4.14596800	H	4.41182300	-3.41909800	-2.72234500
H	-4.32000400	2.82842200	-3.44349700	H	3.06010900	-4.28778100	-3.47065200
H	-4.82445400	1.37870600	-2.55028900	H	3.66565500	-2.79896300	-4.21110100
C	3.48297600	0.22315900	0.00747300	C	2.27295100	-2.97452700	1.39011400
C	3.44484700	-1.17293200	0.01272500	C	2.55811900	-2.21674200	2.66964100
H	2.50061200	-1.70960500	0.01512400	C	3.53219400	-2.98253400	3.56626200
C	4.65752500	-1.85049300	0.01443700	C	1.25639800	-1.88034800	3.40125800
H	4.67295000	-2.93456200	0.01834200	H	3.03736000	-1.26699900	2.40686200
C	5.87054000	-1.14172600	0.01109000	H	4.46926700	-3.19244400	3.03979500
C	5.90569500	0.27187800	0.00565200	H	3.76295500	-2.39582500	4.46238000
H	6.85996100	0.78804000	0.00295100	H	3.10684900	-3.93747300	3.89480400

C	4.70662200	0.94870100	0.00373200
C	4.32201500	2.35458500	-0.00245800
C	5.00376000	3.55258900	-0.00878400
H	6.08864600	3.58855200	-0.00975200
C	4.25734300	4.74677900	-0.01434800
C	2.85792600	4.74056700	-0.01378800
H	2.31948300	5.68210500	-0.01841900
C	2.15178100	3.54429500	-0.00757900
H	1.06630500	3.51524800	-0.00721200
C	2.89460300	2.35297700	-0.00183300
C	7.10179300	-1.86281200	0.01304200
N	8.10678700	-2.44632800	0.01464800
H	4.78390800	5.69558300	-0.01945200

H	0.58302400	-1.30307900	2.75794000
H	0.72949200	-2.79021100	3.71043100
H	1.46610300	-1.28914100	4.29998900
C	-5.67789800	-4.15285100	-0.10922000
C	-5.50409300	-4.98887300	-1.38643800
C	-5.51232300	-5.06177400	1.11819400
C	-7.10248500	-3.59551900	-0.09745900
H	-5.62763500	-4.36354300	-2.27785600
H	-4.51168400	-5.44941900	-1.43596600
H	-6.24996400	-5.79295400	-1.42429300
H	-5.64201300	-4.48933000	2.04360000
H	-6.25839000	-5.86640400	1.10416500
H	-4.52035500	-5.52493900	1.14781400
H	-7.82229500	-4.42182800	-0.12231300
H	-7.29948800	-3.00849700	0.80685900
H	-7.29503900	-2.95922600	-0.96879000
C	-5.67592100	4.15173100	0.10978500
C	-5.50219900	4.98775300	1.38701500
C	-5.50940200	5.06048900	-1.11762400
C	-7.10078100	3.59511200	0.09750100
H	-5.62643900	4.36255300	2.27842700
H	-4.50956600	5.44777600	1.43692000
H	-6.24766100	5.79223100	1.42450700
H	-5.63903000	4.48805000	-2.04304200
H	-6.25505800	5.86550700	-1.10394000
H	-4.51718200	5.52313600	-1.14689000
H	-7.82018900	4.42178100	0.12201700
H	-7.29772100	3.00812800	-0.80685600
H	-7.29399300	2.95897200	0.96879700
C	1.66988300	-4.36481400	-0.97707900
C	1.41155900	-4.93878300	0.26225800
C	1.71081200	-4.25124700	1.43270400
H	1.49506700	-4.70751300	2.39472000
H	0.96865400	-5.92932100	0.31651100
H	1.42378800	-4.90954400	-1.88421200
Au	0.25009000	-0.00121400	0.00095700

Optimized **Au-1^{2tBu}** S_1 semi-coplanar

N	1.86596000	0.11652800	-0.04917400
N	-2.92172400	-1.15283400	0.22653800
N	-3.10768500	0.97190800	-0.14600300
N	-5.29860800	-1.74670500	0.35613600
N	-5.55377900	1.16404500	-0.14541700
C	2.65185400	-0.99494800	-0.13738000
C	2.24845900	-2.33394800	-0.23574000
H	1.19436900	-2.59852400	-0.24964000
C	3.23743200	-3.30240700	-0.30584000
H	2.93345100	-4.34165900	-0.38104900
C	4.61108400	-2.98689800	-0.27780700
C	4.99579000	-1.63326000	-0.17323400
H	6.04626800	-1.36542700	-0.14513700
C	4.03150000	-0.64583400	-0.10328200
C	4.05550200	0.80449400	0.01638300
C	5.05291100	1.75753700	0.09760400
H	6.09356900	1.45324100	0.07512400
C	4.71476400	3.12271600	0.20843700
C	3.35260900	3.48514800	0.23239900
H	3.08587700	4.53394500	0.31543400
C	2.33142000	2.55128800	0.15088200
H	1.28621200	2.84837600	0.16436000
C	2.68801500	1.20036600	0.04471700
C	-2.20305500	-0.02224500	0.01810900
C	-4.28415500	-0.88438400	0.19455800
C	-4.40362900	0.48148700	-0.04193700

Optimized **Au-1^{2tBu}** S_1 orthogonal

N	1.90112900	-0.00114600	-0.06965800
N	-2.93454500	-0.09136200	1.12230200
N	-3.01253300	0.06799900	-1.03799800
N	-5.33414500	-0.10940800	1.60631100
N	-5.44042700	0.11063400	-1.34499800
C	2.71742100	-1.09996000	-0.10585600
C	2.34824600	-2.44852000	-0.15548300
H	1.30079200	-2.73290100	-0.17615300
C	3.35898800	-3.39749700	-0.17414300
H	3.08117000	-4.44573100	-0.21049300
C	4.72338800	-3.04840100	-0.14555700
C	5.07505500	-1.68199000	-0.09918400
H	6.11797600	-1.38653500	-0.07730700
C	4.08598500	-0.71971200	-0.07986100
C	4.07905600	0.73712200	-0.02689600
C	5.05910800	1.70793100	0.01454100
H	6.10478500	1.42166700	0.01106200
C	4.69463600	3.07090000	0.06263700
C	3.32694200	3.40781500	0.06804200
H	3.03917900	4.45333800	0.10520200
C	2.32509300	2.45012200	0.02742200
H	1.27500100	2.72534200	0.03243900
C	2.70694100	1.10508300	-0.02166400
C	-2.14651100	-0.01503000	0.01184000
C	-4.27591900	-0.05640000	0.78674200
C	-4.32620000	0.04668000	-0.60464600

C	-6.48256800	-1.06330400	0.25169000	C	-6.48666600	-0.04562700	0.85837000
H	-7.38379900	-1.66059900	0.36526300	H	-7.41526300	-0.08108600	1.42209400
C	-6.60146700	0.29552700	0.01928400	C	-6.53598300	0.05737100	-0.51550600
H	-7.59352800	0.73572200	-0.04351700	H	-7.50278100	0.10194900	-1.01010600
C	-2.29975900	-2.42162500	0.41991900	C	-2.40623500	-0.18102200	2.44245000
C	-1.99124100	-3.19157000	-0.70885300	C	-2.13626200	-1.44951500	2.97160900
C	-1.31847400	-4.39776900	-0.50167500	C	-1.61355100	-1.51051000	4.26466300
H	-1.06626500	-5.02200300	-1.35496600	H	-1.39979600	-2.47788200	4.71090000
C	-0.97158700	-4.80981300	0.78065000	C	-1.36999800	-0.34997300	4.99053400
H	-0.44844800	-5.75175200	0.92333700	H	-0.96666000	-0.41696800	5.99753100
C	-1.29744900	-4.02589200	1.88168500	C	-1.64350300	0.89549800	4.43666900
H	-1.02461500	-4.35898400	2.87953600	H	-1.45276600	1.79495500	5.01577500
C	-1.97096000	-2.81308500	1.72334000	C	-2.16675500	1.00546700	3.14710700
C	-2.37481800	-2.74836700	-2.10456500	C	-2.43905700	-2.71003000	2.18992900
H	-2.81482400	-1.74877800	-2.03077800	H	-2.62468900	-2.42012500	1.15039600
C	-1.15269500	-2.63861700	-3.01686400	C	-1.26246800	-3.68493500	2.18109500
H	-0.66166200	-3.60899000	-3.15584500	H	-1.04602600	-4.07607000	3.18192400
H	-0.41708900	-1.94108600	-2.59977800	H	-0.35474300	-3.19965400	1.80433300
H	-1.44928100	-2.27041200	-4.00566300	H	-1.48890700	-4.54133400	1.53546600
C	-3.44531400	-3.67469800	-2.68524200	C	-3.71768800	-3.36316200	2.72198300
H	-3.75258500	-3.32803400	-3.67856700	H	-3.97408200	-4.24761200	2.12696900
H	-4.32766800	-3.69351500	-2.03732200	H	-4.55505500	-2.65914800	2.67863900
H	-3.07120000	-4.70055100	-2.78600500	H	-3.58894000	-3.67966500	3.76426400
C	-2.31944600	-1.95827800	2.92287600	C	-2.49986700	2.35647100	2.55118100
H	-2.82842100	-1.06014300	2.55880200	H	-2.70272100	2.20899400	1.48524000
C	-3.29466100	-2.68901100	3.84738800	C	-3.77622300	2.91154200	3.18900000
H	-4.20516900	-2.96400600	3.30510800	H	-4.60390900	2.20605000	3.06300400
H	-3.57517700	-2.04615000	4.68949500	H	-4.05529900	3.86436400	2.72394900
H	-2.84948000	-3.60369200	4.25653200	H	-3.63247800	3.08596200	4.26234000
C	-1.06073000	-1.50018900	3.66155300	C	-1.33395700	3.33877400	2.65518700
H	-0.39719100	-0.94413900	2.98901200	H	-0.43097000	2.92423600	2.19223800
H	-0.49953200	-2.35077600	4.06624000	H	-1.09738100	3.58556400	3.69664900
H	-1.32765600	-0.84503200	4.49890700	H	-1.58448400	4.27539500	2.14371900
C	-2.72549800	2.32570100	-0.37681500	C	-2.58514800	0.16843200	-2.39333200
C	-2.58367200	2.76196100	-1.69921200	C	-2.40378600	-1.01064400	-3.12623600
C	-2.87221300	1.85008500	-2.87235600	C	-2.68592700	-2.36958900	-2.52231900
C	-1.61774900	1.62092300	-3.71706800	C	-1.48392200	-3.30648000	-2.64150900
C	-4.03809400	2.38596200	-3.70475800	C	-3.94875500	-2.97468500	-3.14010300
H	-3.17830900	0.87732000	-2.47438700	H	-2.88038700	-2.22674600	-1.45449900
H	-0.81113100	1.19811200	-3.10687200	H	-0.59538300	-2.85973000	-2.18047700
H	-1.83113700	0.92256600	-4.53466300	H	-1.69351500	-4.25678800	-2.13679600
H	-1.25467800	2.55579700	-4.16020100	H	-1.24491200	-3.53214800	-3.68730600
H	-4.93260400	2.49667400	-3.08334300	H	-4.80276400	-2.30484800	-2.99711200
H	-3.80132700	3.36249500	-4.14357000	H	-3.81947900	-3.14250800	-4.21620800
H	-4.26974100	1.69641200	-4.52456500	H	-4.18075900	-3.93855700	-2.67213600
C	-2.46745700	3.14386200	0.73011400	C	-2.35461100	1.44196200	-2.92930600
C	-2.65396900	2.64697100	2.14797800	C	-2.59471300	2.69557000	-2.11536900
C	-3.83823500	3.35429700	2.81044300	C	-3.88935700	3.37677800	-2.56664900
C	-1.37090300	2.78138700	2.96843300	C	-1.40223700	3.65016100	-2.15877800
H	-2.89693000	1.58062100	2.10163100	H	-2.72853600	2.39392500	-1.07124500
H	-4.75288000	3.19506200	2.22997200	H	-4.73458300	2.68588400	-2.48380400
H	-3.99852200	2.96541300	3.82262400	H	-4.09662900	4.25727400	-1.94716600
H	-3.66384200	4.43422600	2.88651600	H	-3.81496200	3.70512900	-3.61052300
H	-0.54704300	2.23568600	2.49375400	H	-0.48509900	3.14412400	-1.83596600
H	-1.06740500	3.82898200	3.07968900	H	-1.23191900	4.04777400	-3.16597400
H	-1.51857900	2.36907600	3.97317800	H	-1.57827200	4.50318900	-1.49319900
C	5.77662300	4.21356700	0.30190600	C	5.73600000	4.18384000	0.10766400
C	5.60749800	5.18610900	-0.87683700	C	5.55377500	5.09223800	-1.11938400
C	5.60009900	4.97649500	1.62503400	C	5.53725900	5.00735200	1.39062000
C	7.19875100	3.65096900	0.25876800	C	7.16809200	3.64550000	0.09906300
H	5.72938400	4.66566500	-1.83311800	H	5.68955200	4.52724300	-2.04805800
H	4.62213000	5.66279800	-0.87512000	H	4.56010800	5.55059200	-1.14473300
H	6.36325300	5.97793900	-0.81857900	H	6.29489700	5.89929700	-1.09766600
H	5.71683900	4.30402900	2.48197800	H	5.65938500	4.38039000	2.28059000
H	6.35541900	5.76697700	1.70393300	H	6.27934700	5.81263300	1.43408500

H	4.61427100	5.44682200	1.69670900	H	4.54391600	5.46501200	1.43324400
H	7.91711300	4.47449300	0.32960100	H	7.87064100	4.48463600	0.13306900
H	7.39350700	2.96995500	1.09507600	H	7.37060300	3.01021500	0.96885600
H	7.39835900	3.11821600	-0.67797000	H	7.38320800	3.07192000	-0.80966300
C	5.63502200	-4.11425900	-0.35876000	C	5.77525300	-4.15223800	-0.16102200
C	5.42456300	-5.07455900	0.82342300	C	5.57544300	-5.05304000	1.06894600
C	5.44008000	-4.87709300	-1.67934200	C	5.61076700	-4.98730400	-1.44132400
C	7.07552700	-3.60120900	-0.30889900	C	7.20201600	-3.60097500	-0.12640100
H	5.55750200	-4.55381800	1.77808400	H	5.68545200	-4.47968500	1.99590900
H	4.42354700	-5.51745300	0.81686400	H	4.58600200	-5.52106600	1.07643200
H	6.15323500	-5.89198000	0.77430800	H	6.32472600	-5.85282700	1.06954800
H	5.58561900	-4.21337500	-2.53868600	H	5.74656400	-4.36631800	-2.33352200
H	6.16757000	-5.69404100	-1.74974800	H	6.36033400	-5.78651800	-1.46259000
H	4.43863200	-5.31254300	-1.75483800	H	4.62240200	-5.45375600	-1.50124200
H	7.76537000	-4.44941100	-0.37138100	H	7.91255300	-4.43395200	-0.13942700
H	7.29913500	-2.93122500	-1.14688600	H	7.41713400	-2.97014400	-0.99638800
H	7.28735300	-3.07136800	0.62679500	H	7.39279500	-3.01893500	0.78239000
C	-2.15885100	4.07642000	-1.90159700	C	-1.97683800	-0.88852400	-4.44990400
C	-1.89025300	4.91245100	-0.82368100	C	-1.74211400	0.36176900	-5.01027500
C	-2.04518900	4.45088500	0.47903400	C	-1.92962100	1.51538300	-4.25692600
H	-1.84030400	5.11604000	1.31384200	H	-1.74883100	2.48715600	-4.70835900
H	-1.56290900	5.93369000	-1.00009800	H	-1.41419100	0.43830800	-6.04361700
H	-2.03737200	4.44867800	-2.91546200	H	-1.83196000	-1.78298900	-5.04999700
Au	-0.21052300	0.09359200	-0.02326200	Au	-0.16037100	-0.01014300	-0.04235700
Optimized **Au-1^{2tBu}** T_1 semi-coplanar				Optimized **Au-1^{2OMe}** S_0 semi-coplanar			
N	-1.85872100	-0.17547900	-0.03077600	Au	0.16856900	-0.00021000	0.00042800
N	3.12692700	-0.94516200	0.04733500	N	-2.61200200	-1.09308100	-0.03125800
N	2.85553700	1.20424600	-0.02737800	N	-2.61153800	1.09360600	0.03061000
N	5.57231500	-1.03401000	0.10228000	N	-5.02987200	-1.43630800	-0.04098200
N	5.20114500	1.90424400	0.00112000	N	-5.02925200	1.43793300	0.03862600
C	-2.69766500	-1.25536200	-0.04366000	N	2.17782200	-0.00026800	0.00070400
C	-2.35592500	-2.61194000	-0.06608300	C	-1.80297500	0.00007600	-0.00003400
H	-1.31417900	-2.91995900	-0.07541600	C	-3.93655200	-0.70226600	-0.02024400
C	-3.38864000	-3.53754100	-0.07455700	C	-3.93625000	0.70339100	0.01868700
H	-3.13490300	-4.59245000	-0.09164200	C	-6.14955300	0.70165300	0.01780100
C	-4.74519900	-3.15702600	-0.06143000	H	-7.08973800	1.24500600	0.03243200
C	-5.06724300	-1.78289000	-0.03877400	C	-6.14985600	-0.69952300	-0.02096500
H	-6.10383400	-1.46489600	-0.02845600	H	-7.09027500	-1.24245200	-0.03627900
C	-4.05747500	-0.84140800	-0.02983200	C	-2.09910600	-2.42983000	-0.07408200
C	-4.01619800	0.61645200	-0.00618800	C	-1.85417800	-3.00408700	-1.32762400
C	-4.96979100	1.61476900	0.01720500	C	-1.28757700	-4.27942700	-1.34398000
H	-6.02299800	1.35755000	0.02014500	H	-1.07342200	-4.75607600	-2.29640400
C	-4.56811900	2.96821500	0.03894800	C	-0.98141800	-4.93971300	-0.15967900
C	-3.19207200	3.26931700	0.03490300	H	-0.53457100	-5.92935800	-0.19352500
H	-2.87434700	4.30686500	0.05088900	C	-1.23775200	-4.33937900	1.06753400
C	-2.21434100	2.28630100	0.01202300	H	-0.98658500	-4.86304400	1.98558800
H	-1.15780600	2.53973500	0.01066000	C	-1.80298300	-3.06504500	1.13832100
C	-2.63595600	0.95169600	-0.00835800	C	-2.14705400	-2.27585500	-2.62249300
C	2.17319900	0.02408300	-0.00451000	H	-2.63152200	-1.32362800	-2.37899600
C	4.39658800	-0.39457100	0.05690400	C	-3.11857900	-3.06667300	-3.49993800
C	4.22149700	0.99130800	0.00893800	H	-2.68815600	-4.02572900	-3.80936500
C	6.58787400	-0.10743200	0.09443700	H	-3.35539400	-2.50058900	-4.40767100
H	7.59553000	-0.51378500	0.12882300	H	-4.05293000	-3.27113100	-2.96654700
C	6.41525000	1.26045700	0.04772700	C	-0.84923700	-1.94750100	-3.36463800
H	7.28985800	1.90591900	0.04617300	H	-0.31714600	-2.86072800	-3.65441800
C	2.80448400	-2.33196800	0.09043100	H	-0.17825300	-1.35135300	-2.73621400
C	2.59569200	-2.93054500	1.33895400	H	-1.06484800	-1.37814700	-4.27601000
C	2.24012000	-4.28068700	1.35516900	C	-2.04992900	-2.40786300	2.47988800
H	2.07447800	-4.77832400	2.30717800	H	-2.50367800	-1.42625300	2.30288400
C	2.10138400	-4.99561200	0.17063700	C	-3.03712900	-3.22062700	3.31963200
H	1.82853700	-6.04722700	0.20243600	H	-3.98577000	-3.35649000	2.78948400
C	2.31766700	-4.37370200	-1.05418100	H	-3.24042600	-2.70887100	4.26694200
H	2.21331900	-4.94392300	-1.97358500	H	-2.63653600	-4.21292400	3.55613400

C	2.67353700	-3.02516200	-1.11912000
C	2.76177700	-2.15619000	2.62896900
H	2.96955300	-1.11332100	2.36950500
C	1.47963800	-2.16716000	3.46189400
H	1.20872300	-3.18190500	3.77650800
H	0.64202600	-1.75204000	2.88925400
H	1.60924000	-1.56098000	4.36596800
C	3.96525300	-2.67787600	3.41658700
H	4.11069100	-2.08741000	4.32855000
H	4.87583300	-2.61098200	2.81236700
H	3.82464100	-3.72488400	3.71076200
C	2.92701800	-2.35122000	-2.45044200
H	3.08802500	-1.28534500	-2.26050600
C	4.20494500	-2.89887300	-3.08975400
H	5.05865000	-2.75474000	-2.41948500
H	4.41335100	-2.38134900	-4.03332200
H	4.11278000	-3.97039500	-3.30435000
C	1.72081700	-2.46541000	-3.38265100
H	0.82751000	-2.03407100	-2.91604200
H	1.50200700	-3.50848900	-3.63970000
H	1.91227600	-1.92588600	-4.31735900
C	2.18502000	2.46103600	-0.07611600
C	1.81350800	3.06745500	1.13090100
C	2.17484500	2.45678900	2.46776800
C	0.94668000	2.26368800	3.35695800
C	3.26026900	3.28875900	3.15458900
H	2.59527300	1.46416900	2.27813600
H	0.19657500	1.64216100	2.85415400
H	1.23136200	1.76619900	4.29115700
H	0.47807700	3.21937300	3.61957600
H	4.14601200	3.36340300	2.51532500
H	2.90424400	4.30365700	3.36913800
H	3.55505000	2.82523600	4.10315000
C	1.87726700	3.00677700	-1.32888400
C	2.30615900	2.33093800	-2.61336300
C	3.42630700	3.12951000	-3.28394600
C	1.12467900	2.09208300	-3.55324800
H	2.71567900	1.34950600	-2.35382000
H	4.27932000	3.23381300	-2.60551500
H	3.76668900	2.62149700	-4.19367000
H	3.08372100	4.13304300	-3.56387000
H	0.34900200	1.49605100	-3.05846000
H	0.67146300	3.03278500	-3.88710500
H	1.45602700	1.54874200	-4.44556200
C	-5.57902900	4.10987500	0.06763000
C	-5.35494200	4.94788300	1.33694200
C	-5.37503600	4.99379900	-1.17360400
C	-7.02537800	3.61112100	0.07029900
H	-5.49291600	4.33871000	2.23694400
H	-4.34854600	5.37698300	1.37057100
H	-6.07353300	5.77483000	1.36920600
H	-5.52778200	4.41808800	-2.09305000
H	-6.09368900	5.82127300	-1.16386900
H	-4.36909800	5.42402900	-1.20759300
H	-7.70490500	4.46949400	0.09171800
H	-7.25781400	3.02861400	-0.82843200
H	-7.24319900	2.99577500	0.95066400
C	-5.82138900	-4.23777100	-0.07068800
C	-5.66655600	-5.09202200	-1.33954300
C	-5.65141100	-5.12814100	1.17116600
C	-7.23584400	-3.65481500	-0.05380000
H	-5.78228900	-4.47906100	-2.24011600
H	-4.68806400	-5.58033600	-1.38653300
H	-6.43320000	-5.87507600	-1.35700000
H	-5.75593900	-4.54142900	2.09039200
C	-0.73334600	-2.17042000	3.22300800
H	-0.23043400	-3.11633400	3.45390400
H	-0.91924600	-1.64800400	4.16828300
H	-0.04847400	-1.56167000	2.62239400
C	-2.09804600	2.43011300	0.07382700
C	-1.85369900	3.00420200	1.32755900
C	-1.28652400	4.27928200	1.34435100
H	-1.07276200	4.75578400	2.29693600
C	-0.97928300	4.93948700	0.16028400
H	-0.53199900	5.92892300	0.19447700
C	-1.23508800	4.33933700	-1.06712900
H	-0.98309700	4.86294400	-1.98499100
C	-1.80084400	3.06525900	-1.13835000
C	-2.14779500	2.27604400	2.62219300
H	-2.63250500	1.32403300	2.37832100
C	-0.85064100	1.94708700	3.36522600
H	-1.06712800	1.37776000	4.27640600
H	-0.31836000	2.86006300	3.65544500
H	-0.17948000	1.35069800	2.73721900
C	-3.11960500	3.06724200	3.49898400
H	-3.35728400	2.50123400	4.40653900
H	-4.05350100	3.27208800	2.96494600
H	-2.68901600	4.02612000	3.80873000
C	-2.04725400	2.40827200	-2.48011000
H	-2.50143500	1.42680400	-2.30343600
C	-0.73034800	2.17045500	-3.22253800
H	-0.91591900	1.64819300	-4.16796300
H	-0.04599700	1.56141900	-2.62161800
H	-0.22698900	3.11621800	-3.45307600
C	-3.03372600	3.22144400	-3.32031600
H	-3.23668400	2.70984200	-4.26778200
H	-2.63266400	4.21362500	-3.55650800
H	-3.98260600	3.35758000	-2.79066600
C	3.00146800	-1.10646800	0.03223400
C	2.65122200	-2.46222800	0.07180800
H	1.60628600	-2.76633800	0.07944500
C	3.66212100	-3.40548400	0.10190100
H	3.43228200	-4.46658300	0.13339200
C	5.02125300	-3.02619400	0.09291500
C	5.38285800	-1.68591700	0.05266600
H	6.42385600	-1.37964400	0.04520800
C	4.36645300	-0.71938900	0.02167800
C	4.36645900	0.71880700	-0.02117900
C	5.38286500	1.68532200	-0.05257500
H	6.42386200	1.37903500	-0.04552400
C	5.02126500	3.02560300	-0.09271500
C	3.66213500	3.40491500	-0.10118400
H	3.43229600	4.46601600	-0.13261500
C	2.65123500	2.46167500	-0.07066800
H	1.60630300	2.76580000	-0.07791900
C	3.00148100	1.10591100	-0.03120000
O	5.90761700	-4.06651800	0.12683000
C	7.27413500	-3.74090900	0.12062900
H	7.55662300	-3.19286000	-0.78908200
H	7.55150200	-3.13778000	0.99643200
H	7.81597000	-4.68828500	0.15138600
O	5.90763400	4.06591000	-0.12700500
C	7.27414600	3.74027700	-0.12135700
H	7.55114400	3.13713500	-0.99726800
H	7.81599000	4.68764000	-0.15234200
H	7.55699400	3.19222500	0.78824200

H	-6.41807000	-5.91137300	1.17533100
H	-4.67259600	-5.61763600	1.19210300
H	-7.96479000	-4.47188100	-0.06128400
H	-7.41996700	-3.05654100	0.84578700
H	-7.43053000	-3.03081100	-0.93346700
C	1.10990700	4.27173700	1.05873400
C	0.79174600	4.83893900	-0.17071300
C	1.17224700	4.21205700	-1.35238800
H	0.92091900	4.66578200	-2.30730700
H	0.24734600	5.77907600	-0.20821900
H	0.81071100	4.77174500	1.97605100
Au	0.19616600	-0.14674400	-0.02404100

Optimized **Au-1^{2OMe}** S$_1$ semi-coplanar

Au	0.20879900	-0.04728300	-0.00833400
N	-2.65000700	-1.01042700	-0.22910900
N	-2.55436200	1.09353100	0.27252100
N	-5.08687400	-1.29728100	-0.27624200
N	-4.95543200	1.58361700	0.40160900
N	2.28757600	-0.06088600	-0.01657000
C	-1.78902000	0.00737800	0.00866600
C	-3.96645900	-0.57830700	-0.11578000
C	-3.90465200	0.77392000	0.20429600
C	-6.10882100	0.86328400	0.23842600
H	-7.03471600	1.41673300	0.37352800
C	-6.17002000	-0.48400900	-0.07595200
H	-7.14275600	-0.95828300	-0.17949000
C	-2.20738300	-2.33505500	-0.51792900
C	-1.98981400	-2.68993700	-1.85437400
C	-1.49034700	-3.96893100	-2.10744500
H	-1.30583700	-4.27731400	-3.13313500
C	-1.22336800	-4.84931700	-1.06509300
H	-0.83375700	-5.84057800	-1.28112700
C	-1.45882100	-4.47030600	0.25202900
H	-1.25393600	-5.16949700	1.05870700
C	-1.95985600	-3.20233900	0.55340300
C	-2.26661700	-1.72662900	-2.98885800
H	-2.67579300	-0.80757000	-2.55708400
C	-3.32255800	-2.28703000	-3.94266500
H	-2.98004300	-3.21049600	-4.42418100
H	-3.54516300	-1.56043800	-4.73234400
H	-4.25042100	-2.50557700	-3.40421200
C	-0.97523100	-1.35219600	-3.71888600
H	-0.51174600	-2.22887200	-4.18681700
H	-0.24911500	-0.91490900	-3.02365600
H	-1.18088600	-0.61857000	-4.50712100
C	-2.21990500	-2.79345000	1.98789900
H	-2.61325100	-1.77182800	1.98150800
C	-3.28759700	-3.68547000	2.62359400
H	-4.21530300	-3.64744700	2.04351800
H	-3.50462000	-3.35105000	3.64444600
H	-2.95868400	-4.73006200	2.67507800
C	-0.92643100	-2.77749100	2.80423900
H	-0.46958400	-3.77322100	2.85277600
H	-1.12664200	-2.44937500	3.83079800
H	-0.19736900	-2.08822200	2.36230900
C	-1.99099100	2.37799600	0.52972200
C	-1.69729100	2.72638900	1.85307700
C	-1.09344900	3.96585500	2.07381600
H	-0.85235400	4.26963800	3.08903900
C	-0.79659900	4.81325900	1.01254100
H	-0.32653200	5.77435600	1.20330400
C	-1.10355700	4.43989000	-0.29161000
H	-0.87445600	5.11400600	-1.11282700
C	-1.70946500	3.21104700	-0.56086700

Optimized **Au-1^{2OMe}** S$_1$ orthogonal

Au	-0.25804800	0.00033200	-0.00403600
N	2.56093500	0.38098900	-1.01311800
N	2.55353600	-0.37694100	1.01634800
N	4.97825000	0.51667700	-1.37973500
N	4.96865500	-0.52123600	1.39711400
N	-2.32303500	0.00485300	0.00287400
C	1.72914800	0.00154200	-0.00160800
C	3.88932000	0.24644300	-0.64664200
C	3.88448700	-0.24227900	0.65988600
C	6.09596900	-0.24880700	0.65673100
H	7.04284500	-0.43949200	1.15540000
C	6.10038300	0.23451700	-0.63522500
H	7.05084200	0.41635700	-1.13041700
C	2.09883900	0.85476900	-2.27376600
C	1.85958300	-0.07596800	-3.29156300
C	1.39434500	0.41023800	-4.51483800
H	1.19814100	-0.28522200	-5.32684200
C	1.18549900	1.77109600	-4.70784900
H	0.82737500	2.13257500	-5.66819800
C	1.43860000	2.67235100	-3.67978600
H	1.27882500	3.73487600	-3.84385800
C	1.90145400	2.23104800	-2.43893700
C	2.10176800	-1.55607600	-3.08705300
H	2.41703700	-1.70339100	-2.04913700
C	3.24012000	-2.04878400	-3.98207200
H	2.99186400	-1.93402200	-5.04397100
H	3.44052300	-3.11027800	-3.79570600
H	4.15619900	-1.48390800	-3.78134500
C	0.81960100	-2.36409200	-3.29260800
H	0.45267700	-2.28316500	-4.32262900
H	0.02811200	-2.00801100	-2.62326700
H	0.99889300	-3.42512600	-3.08272700
C	2.19360100	3.21309100	-1.32472300
H	2.45731200	2.63702200	-0.43196300
C	3.40108600	4.08348100	-1.67776600
H	4.27633100	3.45635200	-1.87534100
H	3.63941300	4.76164300	-0.85005300
H	3.20533500	4.69263300	-2.56841400
C	0.96254600	4.05060000	-0.97712400
H	0.64965600	4.68043600	-1.81813700
H	1.17779900	4.70981800	-0.12801000
H	0.11942400	3.40416000	-0.70789800
C	2.07853100	-0.85817600	2.26932600
C	1.84559500	0.06527400	3.29534000
C	1.37996700	-0.42898700	4.51522200
H	1.19238400	0.25977900	5.33480100
C	1.16142600	-1.79000600	4.69563400
H	0.80327500	-2.15801600	5.65350000
C	1.40433200	-2.68343600	3.65819100
H	1.23594600	-3.74594000	3.81279200
C	1.86783300	-2.23408000	2.42044500

C	-2.02043900	1.80339700	3.00822700
H	-2.42754300	0.87597000	2.59288000
C	-0.76398100	1.43290900	3.79720300
H	-1.01194000	0.72267600	4.59444700
H	-0.30519500	2.31252900	4.26377300
H	-0.01801600	0.96738400	3.14277600
C	-3.10224200	2.41529700	3.90017900
H	-3.36293600	1.72647000	4.71189500
H	-4.00636200	2.62195800	3.31829100
H	-2.76174400	3.35496800	4.35121300
C	-2.05904500	2.81323700	-1.97941900
H	-2.43771500	1.78634200	-1.95817400
C	-0.83079800	2.82153300	-2.88987400
H	-1.10448600	2.49092800	-3.89836700
H	-0.05828800	2.14398300	-2.50829200
H	-0.39452200	3.82373700	-2.97502800
C	-3.18206300	3.70053200	-2.52094900
H	-3.46470500	3.38524300	-3.53191900
H	-2.87008700	4.75067800	-2.56879900
H	-4.06597900	3.63446100	-1.87825200
C	3.09482800	-1.13727000	0.19714500
C	2.72329000	-2.46836900	0.44771000
H	1.67495700	-2.75103200	0.48697200
C	3.72303300	-3.40152900	0.63103200
H	3.48985200	-4.44254100	0.82751500
C	5.08446100	-3.03470600	0.56310900
C	5.46719500	-1.70566800	0.30284300
H	6.51269400	-1.42423900	0.24417600
C	4.46860100	-0.76319600	0.12096600
C	4.46213200	0.65992400	-0.16206200
C	5.45174800	1.61161200	-0.34456600
H	6.49990500	1.33930300	-0.29047100
C	5.05639600	2.93850100	-0.59781200
C	3.69155700	3.29423700	-0.65727900
H	3.44821700	4.33433800	-0.84615700
C	2.70059700	2.35207000	-0.47358100
H	1.64996200	2.62772300	-0.50321600
C	3.08492200	1.02252700	-0.23183400
O	5.95679900	-4.04062000	0.75887800
C	7.34020400	-3.75406900	0.70345900
H	7.62743300	-3.37193700	-0.28336600
H	7.62686100	-3.02992600	1.47534500
H	7.84845500	-4.70061500	0.88771400
O	5.91922500	3.95255500	-0.79291800
C	7.30536400	3.67813100	-0.74329400
H	7.59580900	2.95988300	-1.51922400
H	7.80449700	4.62996400	-0.92516000
H	7.59918500	3.29440100	0.24094900

C	2.11346500	1.54310300	3.10644700
H	2.34748900	1.70764800	2.04966800
C	0.88304100	2.38885800	3.43492700
H	1.08164700	3.44630000	3.22467100
H	0.60306300	2.30648100	4.49167200
H	0.02396400	2.07215800	2.83240600
C	3.33899200	1.97091100	3.91658900
H	3.56080900	3.03083300	3.74560100
H	4.21436600	1.38201100	3.62377400
H	3.17301500	1.82855000	4.99125400
C	2.15559200	-3.20609000	1.29622600
H	2.36187200	-2.62077900	0.39416600
C	0.95147400	-4.09672200	0.99083400
H	1.16611700	-4.74194000	0.13097200
H	0.06943200	-3.49011800	0.75515100
H	0.69859800	-4.74549400	1.83748100
C	3.41164600	-4.02319600	1.60775500
H	3.64983100	-4.69383800	0.77382200
H	3.27034500	-4.63596100	2.50631500
H	4.26531300	-3.35852800	1.77442500
C	-3.13045400	1.05305600	0.34596000
C	-2.75546700	2.33764000	0.76664400
H	-1.70696400	2.60343100	0.85373300
C	-3.74983200	3.24625700	1.06472200
H	-3.51203400	4.25161100	1.39432900
C	-5.11223000	2.89662700	0.95040700
C	-5.49950600	1.60893300	0.52683000
H	-6.54542000	1.33852300	0.43787800
C	-4.50312500	0.69808000	0.22846600
C	-4.50449900	-0.68325800	-0.22576800
C	-5.50257900	-1.59186400	-0.52543700
H	-6.54803000	-1.31909200	-0.43812800
C	-5.11759200	-2.88063300	-0.94792000
C	-3.75589300	-3.23363400	-1.05979500
H	-3.52006700	-4.23975200	-1.38850200
C	-2.75980400	-2.32734500	-0.76034000
H	-1.71165800	-2.59562900	-0.84474900
C	-3.13258100	-1.04152700	-0.34126700
O	-5.97974200	3.86824300	1.27033000
C	-7.36694800	3.59740900	1.18514400
H	-7.65747600	3.34145400	0.15981700
H	-7.65372300	2.78674000	1.86473200
H	-7.86749800	4.51750900	1.48560800
O	-5.98683500	-3.85045700	-1.26873400
C	-7.37355500	-3.57678200	-1.18499600
H	-7.65814200	-2.76621100	-1.86563200
H	-7.87568600	-4.49616600	-1.48500600
H	-7.66442000	-3.31924200	-0.16017200

Optimized **Au-1**$^{\mathbf{2OMe}}$ T_1 semi-coplanar

Au	0.20811900	-0.12600500	0.01069300
N	-2.71942100	-0.92277700	-0.03483600
N	-2.45535500	1.22665400	0.04180000
N	-5.16549100	-1.02174000	-0.07145500
N	-4.80440200	1.91854100	0.03731400
N	2.26615600	-0.18536000	0.00612900
C	-1.76976700	0.04987700	0.00913200
C	-3.99210500	-0.37700900	-0.03271500
C	-3.82190400	1.00857300	0.01790200
C	-6.01607900	1.26988600	-0.00143400
H	-6.89342800	1.91168200	0.00994800
C	-6.18409300	-0.09857400	-0.05215300
H	-7.19067000	-0.50828000	-0.08017300
C	-2.38980600	-2.30768000	-0.08367500
C	-2.21425200	-2.90795000	-1.33650200

Optimized **Au-1**$^{\mathbf{OMe}}$ S_0 semi-coplanar

Au	0.36933500	0.26701000	-0.00462600
N	-1.99881200	-1.55174300	-0.02267400
N	-2.60673200	0.54830100	0.04690600
N	-4.22553400	-2.55462100	-0.02851600
N	-5.02473300	0.20588700	0.06359600
N	2.30410700	0.81426700	-0.02101400
C	-1.52626400	-0.27674800	0.00876200
C	-3.37973500	-1.54524600	-0.00666300
C	-3.77054700	-0.19537900	0.03789800
C	-5.89577200	-0.81294800	0.04216100
H	-6.94998900	-0.55287000	0.06122200
C	-5.50611000	-2.15886200	-0.00294200
H	-6.25814700	-2.94213600	-0.01887100
C	-1.13470600	-2.69297700	-0.07701300
C	-0.74710700	-3.16852900	-1.33594400

C	-1.84352800	-4.25378200	-1.36045900	C	0.15019200	-4.23709500	-1.36399100
H	-1.70223300	-4.75236700	-2.31589100	H	0.48279900	-4.62988100	-2.32062600
C	-1.65517800	-4.96254900	-0.17896100	C	0.63336200	-4.79466700	-0.18590000
H	-1.36823500	-6.01020100	-0.21659000	H	1.33631100	-5.62184100	-0.22904500
C	-1.83906600	-4.33914300	1.05031600	C	0.22800100	-4.29655300	1.04689200
H	-1.69389700	-4.90365400	1.96783000	H	0.61943000	-4.73645800	1.95981200
C	-2.21262600	-2.99564400	1.12283600	C	-0.66750800	-3.22891800	1.12949000
C	-2.42509500	-2.13754000	-2.62233600	C	-1.23836500	-2.54217400	-2.62408000
H	-2.63962800	-1.09707000	-2.35851500	H	-1.95708300	-1.75466100	-2.37079800
C	-3.64174000	-2.67556500	-3.37790600	C	-1.97339900	-3.56272000	-3.49453700
H	-3.49589800	-3.72135200	-3.67394000	H	-1.30572800	-4.37112100	-3.81314400
H	-3.81766400	-2.08871700	-4.28684200	H	-2.36406900	-3.07923900	-4.39684700
H	-4.53692900	-2.61792600	-2.75029900	H	-2.81240800	-4.01045700	-2.95144100
C	-1.16446700	-2.13333800	-3.48765600	C	-0.08487200	-1.87665500	-3.37861800
H	-0.88985800	-3.14492200	-3.80920300	H	0.66820800	-2.61307500	-3.68135300
H	-0.31752500	-1.70879200	-2.93612900	H	0.41050500	-1.12513500	-2.75383600
H	-1.32397800	-1.52861400	-4.38793100	H	-0.45673600	-1.38227000	-4.28333200
C	-2.41575000	-2.31918100	2.46175500	C	-1.07661100	-2.67179700	2.47668300
H	-2.68588200	-1.27548400	2.27283000	H	-1.79683200	-1.86309800	2.30922000
C	-3.57762100	-2.95875800	3.22390100	C	-1.77610200	-3.73402600	3.32662100
H	-4.49810000	-2.90859200	2.63319200	H	-2.65089200	-4.13992900	2.80758500
H	-3.74654400	-2.43465100	4.17159600	H	-2.10786900	-3.30095200	4.27685700
H	-3.37454500	-4.01159700	3.45329000	H	-1.10277700	-4.56718400	3.55767400
C	-1.12494300	-2.31226600	3.28223800	C	0.12577000	-2.06459100	3.20341900
H	-0.79141600	-3.33002100	3.51755400	H	0.88352200	-2.82540600	3.42246100
H	-1.27805900	-1.78206000	4.22942400	H	-0.18938900	-1.61890500	4.15374300
H	-0.32097300	-1.80740600	2.73387300	H	0.59759800	-1.28348700	2.59684900
C	-1.78675100	2.48438600	0.09175000	C	-2.48748900	1.97503300	0.09174400
C	-1.45806500	3.01762200	1.34457100	C	-2.40098900	2.59223900	1.34577700
C	-0.74206700	4.21629300	1.36852200	C	-2.21760000	3.97567300	1.36289700
H	-0.47197300	4.65834000	2.32393200	H	-2.13698100	4.49165300	2.31545800
C	-0.37153200	4.84979800	0.18721600	C	-2.12287600	4.69784000	0.17899000
H	0.18419600	5.78319200	0.22510600	H	-1.97396400	5.77341200	0.21353900
C	-0.71412700	4.29714700	-1.04220400	C	-2.21121300	4.05206200	-1.04875100
H	-0.42242000	4.80158400	-1.95972800	H	-2.12710900	4.62741700	-1.96635900
C	-1.43076000	3.10055200	-1.11467400	C	-2.39465100	2.67019300	-1.12038800
C	-1.86186900	2.32995400	2.63109100	C	-2.46265300	1.80805500	2.63963700
H	-2.34492600	1.38381200	2.36759700	H	-2.65415900	0.75714600	2.39529500
C	-0.64445900	1.99221600	3.49259600	C	-1.11995600	1.86530700	3.37232300
H	-0.95533700	1.44861300	4.39225200	H	-1.15556000	1.25418500	4.28143900
H	-0.11285200	2.89528400	3.81512100	H	-0.87156000	2.89187800	3.66472400
H	0.05972900	1.36133300	2.93783800	H	-0.31011500	1.49067500	2.73645000
C	-2.88955100	3.17047800	3.39149200	C	-3.61468800	2.28391200	3.52605100
H	-3.20904300	2.65080600	4.30212800	H	-3.67234800	1.67179200	4.43296700
H	-3.77218900	3.35158200	2.76969000	H	-4.57194400	2.21164400	2.99900000
H	-2.47132000	4.14063400	3.68548800	H	-3.47672300	3.32570200	3.83661100
C	-1.80085100	2.49968000	-2.45370900	C	-2.45603200	1.97140100	-2.46222400
H	-2.32201100	1.55601800	-2.26423100	H	-2.62611500	0.90345100	-2.28519200
C	-0.55638200	2.17381800	-3.28095900	C	-1.12434400	2.10085500	-3.20536800
H	-0.84164500	1.69714000	-4.22594800	H	-1.16084700	1.54788800	-4.15086300
H	0.10159800	1.48660900	-2.73632100	H	-0.29959000	1.70127000	-2.60477500
H	0.01686600	3.07722700	-3.52090600	H	-0.89772400	3.14808000	-3.43560600
C	-2.76923000	3.41199000	-3.20866700	C	-3.62723300	2.48465700	-3.30175500
H	-3.06774500	2.94975100	-4.15656500	H	-3.68534000	1.93456900	-4.24758500
H	-2.30962000	4.38113800	-3.43662700	H	-3.51088100	3.54762200	-3.54153800
H	-3.67041600	3.59168000	-2.61361900	H	-4.57663600	2.35974500	-2.77030700
C	3.07642000	-1.28356800	0.03683500	C	3.39610700	-0.03148000	0.02523400
C	2.70265700	-2.63491900	0.08175400	C	3.41997300	-1.42957500	0.09668200
H	1.65377300	-2.91719200	0.09554200	H	2.49438100	-2.00151400	0.12089700
C	3.70226800	-3.58595400	0.10672300	C	4.64683000	-2.06795400	0.13569800
H	3.46975400	-4.64464400	0.14133600	H	4.70907700	-3.15093700	0.19121400
C	5.06304200	-3.21206800	0.08882200	C	5.85411000	-1.33967000	0.10382900
C	5.44650200	-1.85636000	0.04371500	C	5.84349000	0.04796700	0.03205000
H	6.49172000	-1.56896100	0.03179100	H	6.76417900	0.62157500	0.00710300
C	4.44747900	-0.90120200	0.01729200	C	4.60597300	0.70563800	-0.00791300

C	4.44015900	0.55360800	-0.03008100	C	4.21929000	2.09149900	-0.08043000
C	5.42864300	1.51941600	-0.06853800	C	4.91978300	3.29988900	-0.14277400
H	6.47691800	1.24339300	-0.06927700	H	6.00763400	3.30668600	-0.14083600
C	5.03014000	2.87120800	-0.10885100	C	4.21026100	4.49157800	-0.20820100
C	3.66533500	3.23047100	-0.10984900	C	2.80364500	4.48747000	-0.21101000
H	3.41851400	4.28610100	-0.14101800	H	2.26816900	5.43248300	-0.26193700
C	2.67595800	2.26958000	-0.07333200	C	2.08371600	3.30288200	-0.14955000
H	1.62519100	2.54596800	-0.07513100	H	0.99514800	3.30239800	-0.15159800
C	3.06584100	0.92179900	-0.03346700	C	2.79493300	2.09830400	-0.08458400
O	5.93483000	-4.23275300	0.11761400	H	4.74354000	5.43714700	-0.25725400
C	7.32015500	-3.94323700	0.10389800	O	6.98708900	-2.10279100	0.14881900
H	7.60413300	-3.40956500	-0.81050900	C	8.21652100	-1.42312200	0.11988000
H	7.60993600	-3.35320800	0.98108700	H	8.33624500	-0.84023900	-0.80394000
H	7.82538500	-4.90844100	0.13260400	H	8.32734400	-0.74828100	0.98001100
O	5.89103500	3.90073300	-0.14850000	H	8.99235000	-2.19007000	0.16330500
C	7.27936500	3.62557000	-0.15409300				
H	7.56303000	3.03853900	-1.03528900				
H	7.77413100	4.59595100	-0.18969800				
H	7.58158400	3.09487500	0.75618000				
Optimized **Au-1^{OMe}** S_1 semi-coplanar				Optimized **Au-1^{OMe}** S_1 orthogonal			
Au	0.38181500	0.38816100	-0.04813800	Au	-0.49816800	-0.00016900	0.21527600
N	-1.87168000	-1.57058800	-0.14815400	N	2.28192000	1.08362700	-0.21994000
N	-2.64776000	0.42631200	0.16416200	N	2.28240200	-1.08331000	-0.21907200
N	-3.98132500	-2.82308000	-0.16384000	N	4.67109200	1.48193300	-0.57680100
N	-5.04511500	-0.08897000	0.25247200	N	4.67181100	-1.48079800	-0.57545200
N	2.38268700	0.95416000	-0.09649700	N	-2.53467600	-0.00029100	0.55570900
C	-1.50127900	-0.27409900	-0.00429900	C	1.46353500	0.00000600	-0.09764800
C	-3.25220600	-1.70129600	-0.07230100	C	3.59621600	0.69815600	-0.41583200
C	-3.75130400	-0.41783200	0.12557500	C	3.59654300	-0.69739800	-0.41522800
C	-5.80429500	-1.22550500	0.15856600	C	5.78380200	-0.68854900	-0.74217400
H	-6.87860300	-1.08408100	0.24682600	H	6.72213600	-1.21883000	-0.88236900
C	-5.30755700	-2.50307300	-0.03381800	C	5.78347000	0.69007500	-0.74281200
H	-6.00232800	-3.33725100	-0.09093800	H	6.72155300	1.22067200	-0.88349700
C	-0.91954600	-2.61957400	-0.31169400	C	1.80799800	2.42580100	-0.16057900
C	-0.51926900	-2.96366100	-1.60834500	C	1.40861100	3.04590400	-1.35109000
C	0.45961900	-3.95072300	-1.74026000	C	0.94622700	4.36069100	-1.26990500
H	0.79431300	-4.24214100	-2.73228600	H	0.63614400	4.87608900	-2.17494200
C	1.00965300	-4.56373500	-0.62025300	C	0.88404300	5.01974600	-0.04748500
H	1.76889200	-5.33177100	-0.74235600	H	0.52531100	6.04473700	-0.00353200
C	0.58834500	-4.20336300	0.65546700	C	1.28179300	4.37587200	1.11887500
H	1.01922900	-4.69480900	1.52386000	H	1.23198800	4.90355700	2.06734900
C	-0.38694300	-3.22025900	0.83654500	C	1.75157400	3.06173200	1.08624300
C	-1.11689900	-2.29558700	-2.82780100	C	1.50664700	2.33385500	-2.68340800
H	-1.82530000	-1.53673200	-2.48037000	H	1.71057300	1.27715200	-2.48178100
C	-1.90448300	-3.30415000	-3.66601700	C	2.68504000	2.88329000	-3.49144600
H	-1.25305000	-4.09736300	-4.05171000	H	2.53812400	3.94480500	-3.72521000
H	-2.37156900	-2.80563100	-4.52303300	H	2.78946500	2.33852300	-4.43714100
H	-2.69268100	-3.76983900	-3.06554800	H	3.61726200	2.78169000	-2.92649600
C	-0.04677900	-1.57548300	-3.64961300	C	0.19696400	2.39698800	-3.46842900
H	0.69606200	-2.27671700	-4.04771700	H	-0.05840100	3.42307700	-3.75738200
H	0.47918500	-0.83402000	-3.03703600	H	-0.63311500	1.99612200	-2.87530400
H	-0.50545200	-1.05564200	-4.49867800	H	0.28021100	1.80639100	-4.38813500
C	-0.85380200	-2.83789100	2.22497800	C	2.22313900	2.36690100	2.34576300
H	-1.53325300	-1.98516400	2.12833400	H	2.32349500	1.30024500	2.11874400
C	-1.64874200	-3.98425700	2.85412100	C	3.60768200	2.88606600	2.74363300
H	-2.50381400	-4.24814200	2.22335700	H	4.31953100	2.74896400	1.92315700
H	-2.02362900	-3.69253000	3.84190000	H	3.98114900	2.34942300	3.62375400
H	-1.02497500	-4.87751500	2.97885500	H	3.56688800	3.95453700	2.98833200
C	0.30852000	-2.38926800	3.11105500	C	1.21781300	2.48967900	3.48983000
H	1.03120600	-3.19721700	3.27507100	H	1.10043900	3.52702900	3.82391200
H	-0.06395100	-2.07246200	4.09190100	H	1.55706000	1.90386800	4.35194400
H	0.83901600	-1.54313900	2.65916200	H	0.23196400	2.11817300	3.18733600
C	-2.66715600	1.84148400	0.33678000	C	1.80921300	-2.42567900	-0.15807100
C	-2.58952500	2.35612500	1.63628400	C	1.75345400	-3.06022300	1.08947500

C	-2.55515000	3.74467000	1.77744200	C	1.28433700	-4.37456500	1.12372900
H	-2.49113200	4.17950300	2.77143000	H	1.23510300	-4.90121500	2.07280900
C	-2.60072100	4.57581800	0.66385300	C	0.88652000	-5.01992700	-0.04177600
H	-2.57499100	5.65459300	0.79309100	H	0.52832100	-6.04505000	0.00343200
C	-2.68512600	4.03395400	-0.61398200	C	0.94795200	-4.36219600	-1.26495200
H	-2.72760500	4.69338000	-1.47692500	H	0.63777700	-4.87876400	-2.16928600
C	-2.71999200	2.65123300	-0.80430700	C	1.40967500	-3.04728400	-1.34776500
C	-2.52849000	1.45167600	2.84879200	C	2.22495600	-2.36376200	2.34811700
H	-2.61218500	0.41691800	2.50122800	H	2.32542900	-1.29741800	2.11969300
C	-1.18298500	1.58504500	3.56487900	C	1.21938200	-2.48490400	3.49215000
H	-1.13234200	0.89476000	4.41507600	H	1.55852100	-1.89801500	4.35357600
H	-1.03260800	2.60195500	3.94650900	H	1.10177800	-3.52180500	3.82753800
H	-0.35618600	1.35212800	2.88374000	H	0.23366800	-2.11364600	3.18892600
C	-3.70594500	1.70920100	3.78995100	C	3.60936600	-2.88253600	2.74688300
H	-3.67480100	1.01379300	4.63642500	H	3.98280400	-2.34472300	3.62630200
H	-4.65635600	1.57023000	3.26465600	H	4.32130700	-2.74664500	1.92628400
H	-3.68325700	2.72839400	4.19324400	H	3.56845100	-3.95066300	2.99305000
C	-2.82334500	2.06241800	-2.19514900	C	1.50705600	-2.33663700	-2.68088600
H	-2.77302500	0.97267000	-2.10444300	H	1.70923700	-1.27937300	-2.48038400
C	-1.65285700	2.49703400	-3.07793100	C	0.19786800	-2.40270700	-3.46645800
H	-1.72283900	2.02131200	-4.06294600	H	0.28062400	-1.81285200	-4.38668300
H	-0.69696700	2.20746300	-2.62611500	H	-0.63320400	-2.00265900	-2.87417200
H	-1.64268100	3.58257200	-3.23136600	H	-0.05566300	-3.42947000	-3.75462800
C	-4.17614800	2.40300800	-2.82348800	C	2.68664400	-2.88525700	-3.48776600
H	-4.26579100	1.93919800	-3.81254100	H	2.79067700	-2.34145900	-4.43406400
H	-4.29692400	3.48592900	-2.94608400	H	2.54131600	-3.94725900	-3.72031700
H	-4.99339900	2.03604300	-2.19407700	H	3.61848000	-2.78168100	-2.92254800
C	3.40969800	0.10883100	0.08325600	C	-3.50976200	-0.00084500	-0.36763600
C	3.36156300	-1.29035400	0.30146300	C	-3.37564200	-0.00194600	-1.77648700
H	2.40886700	-1.81102800	0.33420200	H	-2.38955500	-0.00246800	-2.22875800
C	4.54623900	-1.96026500	0.45814700	C	-4.51466900	-0.00233700	-2.53667700
H	4.57081800	-3.03152500	0.62535300	H	-4.47426300	-0.00318600	-3.62001500
C	5.79101100	-1.27420500	0.39929000	C	-5.79979800	-0.00170100	-1.92717300
C	5.85673900	0.11449600	0.17286400	C	-5.95155300	-0.00063100	-0.52422200
H	6.80925800	0.62909500	0.12245400	H	-6.93407300	-0.00015900	-0.06741900
C	4.67016400	0.80319000	0.01451900	C	-4.81012500	-0.00021200	0.24735900
C	4.32717500	2.18699200	-0.23241200	C	-4.55677900	0.00084600	1.67587800
C	5.05548600	3.35801300	-0.40054900	C	-5.36094600	0.00177700	2.80574300
H	6.14137000	3.35322400	-0.35677500	H	-6.44425100	0.00186300	2.72456500
C	4.36320100	4.55135300	-0.62728700	C	-4.74537700	0.00261600	4.06281900
C	2.96644500	4.57182200	-0.68171700	C	-3.35453600	0.00251200	4.18151700
H	2.45285000	5.51239800	-0.85673100	H	-2.90187900	0.00318000	5.16811400
C	2.21660800	3.40676900	-0.51263700	C	-2.53204000	0.00156500	3.05258300
H	1.13051100	3.41815900	-0.54641200	H	-1.44960300	0.00147200	3.13540000
C	2.90882900	2.21969100	-0.29180700	C	-3.14710400	0.00074600	1.80707900
H	4.91819200	5.47473200	-0.76090900	H	-5.35937900	0.00336900	4.95784800
O	6.86354200	-2.05335900	0.56900800	O	-6.82028700	-0.00224900	-2.78331000
C	8.15377100	-1.46617100	0.52208200	C	-8.14825800	-0.00196700	-2.27893600
H	8.33950300	-1.00799000	-0.45529800	H	-8.33720400	-0.89874400	-1.68004900
H	8.27200300	-0.71753100	1.31287400	H	-8.33723200	0.89554400	-1.68115800
H	8.85593400	-2.28335100	0.68316500	H	-8.79480500	-0.00252100	-3.15524900
Optimized **Au-1**$^{\mathbf{OMe}}$ T_1 semi-coplanar				Optimized **Au-1**$^{\mathbf{2CF3}}$ S_0 semi-coplanar			
Au	0.37790100	0.40548200	-0.02896000	Au	0.23580400	0.02203400	-0.00292700
N	-1.82025800	-1.61164100	0.04235200	N	2.99241100	-1.11525500	0.03334200
N	-2.65817600	0.38665900	0.03376100	N	3.03755600	1.07063600	-0.04245000
N	-3.89207900	-2.91452900	0.12110300	N	5.40315100	-1.50919000	0.04681900
N	-5.03913800	-0.18122400	0.10232600	N	5.46283700	1.36370500	-0.04972500
N	2.35204700	0.99458600	-0.04999500	N	-1.78437300	0.02825100	-0.00032000
C	-1.48026000	-0.29257400	0.01255400	C	2.20992200	-0.00545700	-0.00496400
C	-3.19443500	-1.77188300	0.08174100	C	4.32540100	-0.75207700	0.02058500
C	-3.73455400	-0.48376800	0.07482800	C	4.35453900	0.65209700	-0.02688500
C	-5.76815900	-1.34592900	0.14333000	C	6.56665000	0.60506300	-0.02328800
H	-6.84810900	-1.22400000	0.16888300	C	6.53750400	-0.79693100	0.02360900
C	-5.23447400	-2.61825400	0.15234000	C	2.44815000	-2.43990600	0.08888900

H	-5.90429500	-3.47378900	0.18621500
C	-0.83632300	-2.64239500	0.02426100
C	-0.39827200	-3.12590800	-1.21538200
C	0.61297400	-4.08911800	-1.21255800
H	0.97431800	-4.48693000	-2.15723800
C	1.16000000	-4.54548200	-0.01803800
H	1.94390200	-5.29836200	-0.03451900
C	0.70334700	-4.04777400	1.19773800
H	1.13454700	-4.41383600	2.12580800
C	-0.30646700	-3.08399600	1.24338400
C	-0.99485200	-2.63237500	-2.51591400
H	-1.71266400	-1.84187400	-2.27595900
C	-1.76840700	-3.75330800	-3.21296200
H	-1.10764100	-4.58615300	-3.48187900
H	-2.23366000	-3.38095700	-4.13287400
H	-2.55711600	-4.13808400	-2.55834700
C	0.07049000	-2.01619900	-3.42343000
H	0.82441500	-2.75388700	-3.72288300
H	0.58252300	-1.19122300	-2.91442500
H	-0.39067200	-1.62086100	-4.33584000
C	-0.80699700	-2.54859500	2.56760700
H	-1.54122600	-1.76494100	2.35548100
C	-1.52561900	-3.64709200	3.35353900
H	-2.35727400	-4.05421100	2.76943000
H	-1.92560400	-3.24605900	4.29189100
H	-0.84449500	-4.47012700	3.60100700
C	0.32093800	-1.90717100	3.37674600
H	1.09421100	-2.63732800	3.64352700
H	-0.07329800	-1.48348400	4.30763000
H	0.79558600	-1.09911500	2.80788400
C	-2.71822200	1.80970700	0.01097500
C	-2.66551300	2.50050500	1.22764800
C	-2.67795000	3.89590500	1.17780700
H	-2.63948000	4.46403300	2.10368100
C	-2.74352200	4.56512700	-0.03923800
H	-2.75715900	5.65174600	-0.05893200
C	-2.79820500	3.85080700	-1.23112100
H	-2.85424800	4.38430100	-2.17637500
C	-2.78392600	2.45477200	-1.23018800
C	-2.60060000	1.77283500	2.55344600
H	-2.57653600	0.69857800	2.34499000
C	-1.31938500	2.11565100	3.31505500
H	-1.26851600	1.54638200	4.25053600
H	-1.27346100	3.18154200	3.56823300
H	-0.43531900	1.86845700	2.71569600
C	-3.85400900	2.04780400	3.38609700
H	-3.82122000	1.48098200	4.32363300
H	-4.75322000	1.75278700	2.83579600
H	-3.94068700	3.11133100	3.63862600
C	-2.85248600	1.67512200	-2.52582300
H	-2.72116700	0.61520800	-2.28574800
C	-1.72349100	2.06365300	-3.48049400
H	-1.76388700	1.44667200	-4.38560500
H	-0.74621300	1.91295900	-3.00738100
H	-1.79472200	3.11301100	-3.78979800
C	-4.23210600	1.82918100	-3.16959600
H	-4.29606000	1.23105400	-4.08596900
H	-4.43195000	2.87482800	-3.43310100
H	-5.01356500	1.49255900	-2.48066200
C	3.39674900	0.14864000	-0.04908600
C	3.36719700	-1.26644800	-0.04863600
H	2.42064300	-1.79887300	-0.05000900
C	4.56295400	-1.93548600	-0.04491300
H	4.60304400	-3.01894200	-0.04376600
C	5.79659200	-1.22920800	-0.04159600
C	2.18684200	-2.99435200	1.34821600
C	1.58714000	-4.25434100	1.37648100
C	1.26624900	-4.91900900	0.19847900
C	1.54038100	-4.33858000	-1.03454000
C	2.13831900	-3.07988900	-1.11763200
C	2.49827700	-2.26236400	2.63666800
C	3.45626400	-3.06699900	3.51658000
C	1.21014300	-1.90197700	3.38074400
C	2.40547900	-2.44438900	-2.46573600
C	3.37878700	-3.28874100	-3.29048800
C	1.09803900	-2.18770000	-3.21852600
C	2.55051200	2.41769800	-0.08699800
C	2.32280600	2.99718000	-1.34140800
C	1.77824400	4.28210900	-1.35901700
C	1.47764100	4.94630300	-0.17538500
C	1.71798700	4.34077500	1.05255000
C	2.26101200	3.05683300	1.12501900
C	2.60740400	2.26381600	-2.63529500
C	1.30328600	1.92839800	-3.36359200
C	3.56745200	3.05196200	-3.52741000
C	2.49434200	2.39672300	2.46767300
C	1.17350900	2.18586700	3.21126500
C	3.49629500	3.19220500	3.30662100
C	-2.59664400	-1.08003400	-0.02799000
C	-2.22411700	-2.43034700	-0.06626900
C	-3.22554800	-3.38325400	-0.09316500
C	-4.58459200	-3.01190700	-0.08318300
C	-4.96229200	-1.67586500	-0.04414100
C	-3.96745600	-0.69954700	-0.01667900
C	-3.97226600	0.74083400	0.02038700
C	-4.97330400	1.71011200	0.05111200
C	-4.60504800	3.04926000	0.09188700
C	-3.24894400	3.42986800	0.09893400
C	-2.24091000	2.48344200	0.06837100
C	-2.60363900	1.13091700	0.02892900
C	-5.60998000	-4.09533100	-0.08388600
C	-5.64421600	4.11965000	0.07556200
F	-5.60353900	-4.80067700	1.06759600
F	-5.39288500	-4.99366200	-1.06707800
F	-6.85905400	-3.62727400	-0.24536000
F	-6.86689600	3.65674400	0.38839500
F	-5.36028200	5.11174700	0.94387600
F	-5.75220700	4.70707100	-1.13573400
H	7.51814700	1.12824900	-0.03978300
H	7.46645700	-1.35900900	0.04307500
H	1.35991400	-4.71575400	2.33330500
H	0.79495100	-5.89686300	0.24184600
H	1.27897500	-4.86611000	-1.94749300
H	3.00158300	-1.32193900	2.38532600
H	3.00751700	-4.01511400	3.83340500
H	3.70723200	-2.49984600	4.41975600
H	4.38455900	-3.29378200	2.98174300
H	0.65984600	-2.80147500	3.67909900
H	0.54948200	-1.29607700	2.75054500
H	1.44052400	-1.33078400	4.28720200
H	2.87987000	-1.47092400	-2.29852400
H	4.32148300	-3.43868300	-2.75369300
H	3.59785300	-2.79310800	-4.24275800
H	2.95770700	-4.27481000	-3.51690500
H	0.57609100	-3.12509600	-3.44150800
H	1.29958000	-1.68026800	-4.16861800
H	0.42276300	-1.55760700	-2.62899600
H	1.57850500	4.76418400	-2.31178100
H	1.04952500	5.94413600	-0.21037900
H	1.47354500	4.86882900	1.96988000

C	5.84052900	0.18152300	-0.04180700
H	6.78532300	0.71220900	-0.03831100
C	4.64286700	0.86440600	-0.04533000
C	4.27600000	2.26911800	-0.04340200
C	4.98479200	3.46105300	-0.03779300
H	6.07118900	3.46904800	-0.03550700
C	4.26846000	4.66400100	-0.03433700
C	2.87216700	4.67059100	-0.03609300
H	2.34201300	5.61784400	-0.03273600
C	2.14262200	3.47953300	-0.04177000
H	1.05618700	3.47528500	-0.04218600
C	2.85880900	2.28831900	-0.04576300
H	4.80801600	5.60584900	-0.02969700
O	6.88180100	-2.00500100	-0.03769100
C	8.16528500	-1.39846700	-0.03262100
H	8.31137900	-0.78651700	-0.92884900
H	8.30443000	-0.78697300	0.86503400
H	8.87911600	-2.22114800	-0.03004200

H	3.09769300	1.31467100	-2.39141900
H	1.51121900	1.35742700	-4.27564400
H	0.76552600	2.83944600	-3.64973200
H	0.63990700	1.33157500	-2.72753100
H	3.80027500	2.47810300	-4.43116000
H	4.50474500	3.26687900	-3.00357700
H	3.12938300	4.00513300	-3.84381200
H	2.92980800	1.40633200	2.29318200
H	1.34899600	1.65989000	4.15641000
H	0.47593600	1.59076400	2.61139600
H	0.68983800	3.14162000	3.44255600
H	3.68864300	2.67867400	4.25517600
H	3.11471300	4.19254000	3.54040600
H	4.44782000	3.30851300	2.77712500
H	-1.17516400	-2.71778300	-0.07699500
H	-2.96424800	-4.43708200	-0.12742800
H	-6.01327800	-1.40448500	-0.03850600
H	-6.02247700	1.43118500	0.04915700
H	-2.99527200	4.48516300	0.13670900
H	-1.19372000	2.77731600	0.07877700

Optimized **Au-1^{2CF3}** S_1 semi-coplanar

Au	0.19675700	0.16568200	-0.02393400
N	2.83698300	-1.21834300	-0.03730600
N	3.13766900	0.92572100	0.05357800
N	5.17516200	-1.95218600	0.00201000
N	5.58663000	0.98119800	0.12174400
N	-1.88029000	0.19866100	-0.02681900
C	2.17814000	-0.03020300	-0.00837400
C	4.20899900	-1.02352400	0.00830200
C	4.40234300	0.35579900	0.06569200
C	6.58711400	0.04162000	0.11498500
H	7.60060200	0.43225400	0.15772400
C	6.39544900	-1.32598200	0.05994900
H	7.26188700	-1.98232600	0.06100500
C	2.14705700	-2.46517400	-0.09442800
C	1.77401700	-3.07718700	1.10956500
C	1.04594000	-4.26641800	1.02999100
H	0.74481300	-4.77096800	1.94411800
C	0.70625200	-4.81368600	-0.20301600
H	0.14321500	-5.74248300	-0.24606400
C	1.09103900	-4.18238800	-1.38100600
H	0.82379500	-4.62091500	-2.33874900
C	1.82234600	-2.99275200	-1.35067000
C	2.15606200	-2.48800600	2.45037000
H	2.60531200	-1.50648700	2.26881900
C	3.21803500	-3.35634300	3.12878300
H	2.83367100	-4.36220800	3.33628100
H	3.52689400	-2.90858800	4.08029900
H	4.10040900	-3.45083000	2.48761100
C	0.93507800	-2.26732500	3.34334800
H	0.43927300	-3.21141800	3.59741200
H	0.20235200	-1.61935100	2.84795600
H	1.23506200	-1.78721900	4.28170600
C	2.24909100	-2.31236600	-2.63359800
H	2.74654500	-1.37458400	-2.36667400
C	3.26923000	-3.17108200	-3.38386100
H	4.14228300	-3.36781000	-2.75335000
H	3.60666000	-2.65684100	-4.29094300
H	2.83708700	-4.13359600	-3.68234700
C	1.04560500	-1.95511900	-3.50678900
H	0.50097100	-2.84930900	-3.83204300
H	1.37402200	-1.41952100	-4.40482200
H	0.34730500	-1.30999300	-2.96070500
C	2.83271000	2.31701700	0.09744500

Optimized **Au-1^{2CF3}** S_1 orthogonal

Au	0.14411800	0.01703400	-0.04555700
N	2.91687600	0.06293800	1.12783600
N	2.99571900	-0.04435600	-1.03863700
N	5.31570100	0.05801000	1.60852300
N	5.42161000	-0.08385300	-1.34271900
N	-1.91943300	0.00799700	-0.07489000
C	2.12923100	0.01636100	0.01388000
C	4.25688600	0.03065800	0.79120100
C	4.30746600	-0.03708700	-0.60408500
C	6.51784700	-0.05578500	-0.51467500
H	7.48394700	-0.08960600	-1.01115500
C	6.46855400	0.01035600	0.86181100
H	7.39642700	0.02745500	1.42727600
C	2.38314600	0.13485100	2.44749600
C	2.15580500	1.39775300	3.00880800
C	1.62107900	1.44322300	4.29779500
H	1.43958900	2.40544200	4.76875300
C	1.32602600	0.27339000	4.98844700
H	0.91451500	0.32794200	5.99284100
C	1.55977100	-0.96632900	4.40370400
H	1.33036100	-1.87266000	4.95708900
C	2.09271100	-1.06129100	3.11697100
C	2.51560700	2.66713500	2.26670600
H	2.71734600	2.39811500	1.22432400
C	3.80050900	3.26614600	2.84552500
H	3.65602000	3.55856100	3.89271300
H	4.09689500	4.15790500	2.28095400
H	4.61690200	2.53822600	2.80161200
C	1.37154100	3.67989800	2.25767200
H	1.14269400	4.04959300	3.26379300
H	0.45801700	3.23609000	1.84517300
H	1.64146000	4.54622100	1.64295300
C	2.39321100	-2.40556500	2.48935900
H	2.55486600	-2.24269100	1.41855400
C	3.69095400	-2.97403800	3.07060900
H	4.51644300	-2.26858100	2.93033500
H	3.95087100	-3.91889600	2.57903800
H	3.58334200	-3.16700300	4.14496100
C	1.23147700	-3.38873800	2.62305800
H	1.04299800	-3.66354400	3.66715100
H	1.45727300	-4.31209500	2.07739600
H	0.30765500	-2.96361500	2.21359900
C	2.56389100	-0.12156100	-2.39429800

C	2.71208600	3.01106200	-1.11272400	C	2.36773600	-1.38738600	-2.96062800
C	2.37171300	4.36358600	-1.04768500	C	1.92958300	-1.43983000	-4.28514000
H	2.27556100	4.93553500	-1.96685600	H	1.77420100	-2.40487100	-4.75990100
C	2.16159400	4.98761300	0.17723700	C	1.69864300	-0.27350600	-5.00599400
H	1.90138300	6.04235500	0.20889100	H	1.36149500	-0.33321700	-6.03743200
C	2.29149000	4.27137700	1.36204900	C	1.90375600	0.96896300	-4.41669500
H	2.13193800	4.77138400	2.31379800	H	1.72846300	1.87299800	-4.99359200
C	2.63144200	2.91714300	1.34631500	C	2.34162100	1.07048100	-3.09488500
C	2.96040000	2.33476700	-2.44397900	C	2.65594300	-2.65418400	-2.18362700
H	3.11371000	1.26747400	-2.25514400	H	2.82623300	-2.37377100	-1.13896600
C	1.75526700	2.45847300	-3.37644200	C	1.47555400	-3.62474100	-2.20411400
H	1.94285200	1.91821600	-4.31141900	H	1.69173600	-4.49505800	-1.57389900
H	1.54410200	3.50324000	-3.63264900	H	1.26544500	-3.99292000	-3.21485100
H	0.85829600	2.03334300	-2.91083300	H	0.56693200	-3.14217100	-1.82535400
C	4.24260500	2.87231900	-3.08323100	C	3.94229900	-3.30643000	-2.69727500
H	4.44637100	2.35351000	-4.02698600	H	4.18207300	-4.19880600	-2.10741100
H	5.09516700	2.72060800	-2.41321200	H	4.78057500	-2.60593700	-2.62484000
H	4.15952900	3.94458900	-3.29720100	H	3.83624100	-3.61019400	-3.74575800
C	2.78792600	2.14087700	2.63646300	C	2.60801400	2.41919000	-2.46164800
H	2.99279400	1.09707500	2.37798800	H	2.76172800	2.25963500	-1.38932100
C	1.50135100	2.15748700	3.46266000	C	1.42276700	3.37222200	-2.61022200
H	1.62345600	1.55185200	4.36802200	H	1.62268700	4.30865400	-2.07678400
H	0.66479600	1.74509900	2.88617300	H	0.51036200	2.92743200	-2.19606800
H	1.23272500	3.17362800	3.77442800	H	1.22918800	3.62512700	-3.65899100
C	3.98967900	2.65614000	3.43085100	C	3.89865300	3.01863700	-3.02622300
H	4.12758600	2.06367700	4.34257300	H	4.12556200	3.97265300	-2.53614600
H	3.85275400	3.70326700	3.72590700	H	3.80627800	3.20305900	-4.10340800
H	4.90288200	2.58550600	2.83107900	H	4.74002000	2.33652300	-2.86625100
C	-2.64789700	-0.92942100	-0.01136000	C	-2.73981000	1.10413600	-0.09075900
C	-2.19975000	-2.25667300	-0.00707400	C	-2.35532600	2.44947600	-0.11735100
H	-1.13906700	-2.48982000	-0.01640400	H	-1.30569500	2.72321300	-0.12966100
C	-3.16143200	-3.26027600	0.00678800	C	-3.35995400	3.40786500	-0.12359800
H	-2.85305300	-4.29937000	0.00621700	H	-3.10218100	4.46122000	-0.13753700
C	-4.52165800	-2.94185200	0.01415100	C	-4.70617100	3.02770000	-0.10573400
C	-4.97502900	-1.60802100	0.00680600	C	-5.09706500	1.67830700	-0.07935600
H	-6.03946400	-1.39784300	0.00452100	H	-6.14970400	1.41864700	-0.06413700
C	-4.03597000	-0.60100900	-0.00484000	C	-4.10910500	0.71720000	-0.07076600
C	-4.08359300	0.85407600	-0.02059900	C	-4.09666500	-0.73914000	-0.03869500
C	-5.08714800	1.79618800	-0.01751300	C	-5.06787700	-1.71595800	-0.00917000
H	-6.13548800	1.51524000	-0.00023100	H	-6.12470300	-1.47299700	-0.00295400
C	-4.72385200	3.15754700	-0.02996200	C	-4.65489700	-3.05992800	0.01106300
C	-3.38876300	3.56585700	-0.04199200	C	-3.30367200	-3.41726700	-0.00030200
H	-3.15429800	4.62377900	-0.04650400	H	-3.02790700	-4.46585700	0.00663100
C	-2.36233600	2.62682000	-0.04176700	C	-2.31475700	-2.44128600	-0.02761300
H	-1.31789300	2.92348700	-0.04668200	H	-1.26094200	-2.69874500	-0.03901600
C	-2.71916400	1.27378600	-0.03235400	C	-2.72121200	-1.10331000	-0.04549500
C	-5.54309000	-4.04061200	0.09050800	C	-5.74808800	4.11036900	-0.14831200
C	-5.82574200	4.17733600	-0.07401700	C	-5.68597200	-4.15011100	0.09780300
F	-5.94363100	-4.23807000	1.35751800	F	-5.78095400	4.69723100	-1.35487400
F	-5.06218100	-5.20526900	-0.36278600	F	-5.48535200	5.07045800	0.75015000
F	-6.63787700	-3.74197900	-0.62582600	F	-6.97546100	3.63744600	0.10460400
F	-6.80661500	3.86841300	0.78903400	F	-6.89302900	-3.72751200	-0.30241100
F	-5.38985400	5.40698000	0.22479700	F	-5.34834100	-5.20387500	-0.65842100
F	-6.38245300	4.23140300	-1.29542700	F	-5.81270500	-4.59123500	1.35928100
Optimized **Au-1^{2CF3}** T_1 semi-coplanar				Optimized **Au-1^{CF3}** S_0 semi-coplanar			
Au	0.19108400	0.17081300	-0.01241700	Au	0.02746100	0.44481300	0.00731000
N	2.81817000	-1.23034400	0.00495200	N	-1.95039700	-1.77739100	0.00377600
N	3.13711300	0.91667200	0.02191700	N	-2.95988000	0.16319700	-0.00593100
N	5.14664900	-1.97756700	0.04555700	N	-3.93671300	-3.19899300	-0.00569400
N	5.58158700	0.95084900	0.06389100	N	-5.26345300	-0.64821800	-0.01846900
N	-1.86183400	0.20904900	-0.01805000	N	1.86398100	1.27818200	0.01154500
C	2.16135000	-0.03333800	0.00018000	C	-1.73986600	-0.43403300	0.00210900
C	4.18657400	-1.04611800	0.03167700	C	-3.30587400	-2.04258000	-0.00377800
C	4.39213900	0.33884000	0.04115200	C	-3.95460600	-0.79581600	-0.01000300

C	6.57709000	0.00412700	0.07764100	C	-5.91695400	-1.81803900	-0.02045500
H	7.59309100	0.38972800	0.09661700	H	-7.00184600	-1.77016100	-0.02735400
C	6.37443600	-1.36109400	0.06958600	C	-5.26966200	-3.06236200	-0.01420500
H	7.23430400	-2.02571400	0.08259600	H	-5.85337400	-3.97815400	-0.01626900
C	2.11797400	-2.47199500	-0.01881900	C	-0.87551900	-2.72548800	0.00877800
C	1.74651800	-3.05352600	1.20069400	C	-0.34744600	-3.13351200	-1.22265100
C	1.01101900	-4.23995100	1.15258800	C	0.74797200	-3.99806800	-1.19013300
H	0.71184600	-4.72148300	2.07965700	H	1.19249300	-4.33126900	-2.12358400
C	0.66163400	-4.81383000	-0.06549000	C	1.28408400	-4.42645700	0.01872300
H	0.09356400	-5.74041800	-0.08386800	H	2.14118600	-5.09400200	0.02269400
C	1.04227300	-4.21133300	-1.25991100	C	0.73418800	-4.00140900	1.22257100
H	0.76672900	-4.66986200	-2.20591100	H	1.16819700	-4.33703000	2.16008400
C	1.78045200	-3.02538500	-1.26083700	C	-0.36173200	-3.13719600	1.24496000
C	2.14063900	-2.43566300	2.52487400	C	-0.90123100	-2.64549700	-2.54475400
H	2.58179300	-1.45549000	2.31767800	H	-1.77095700	-2.01102700	-2.34005600
C	3.21481600	-3.28568900	3.20747600	C	-1.38427800	-3.81160200	-3.40884000
H	2.83729100	-4.28906700	3.43845700	H	-0.55745000	-4.47831900	-3.67829800
H	3.53236100	-2.81842700	4.14670800	H	-1.82551500	-3.43629400	-4.33884900
H	4.09038100	-3.38877400	2.55834000	H	-2.13958500	-4.40471400	-2.88250300
C	0.92981200	-2.20310300	3.42837600	C	0.12715000	-1.78233000	-3.27966500
H	0.44252000	-3.14400000	3.70927400	H	1.02170300	-2.36155300	-3.53505300
H	0.18743700	-1.56951300	2.92876500	H	0.44051000	-0.93361500	-2.66147300
H	1.23924000	-1.70164200	4.35238200	H	-0.29924700	-1.39160100	-4.21056500
C	2.20273200	-2.37676800	-2.56158500	C	-0.93009100	-2.65157400	2.56171800
H	2.69752000	-1.43105900	-2.31918700	H	-1.80304800	-2.02433400	2.34872900
C	3.22389100	-3.25207900	-3.29107400	C	-1.41100100	-3.81926500	3.42474100
H	4.09787300	-3.43152900	-2.65675300	H	-2.15687300	-4.41996000	2.89361300
H	3.55953600	-2.76026300	-4.21119200	H	-1.86337100	-3.44494100	4.34976800
H	2.79297900	-4.22257900	-3.56461900	H	-0.58129100	-4.47847600	3.70359800
C	0.99763400	-2.04480000	-3.44233300	C	0.08617100	-1.77919300	3.30271900
H	0.45418400	-2.94787700	-3.74414500	H	0.98326100	-2.35139400	3.56506200
H	1.32440100	-1.53215900	-4.35426600	H	-0.34976300	-1.39041400	4.23003000
H	0.29914700	-1.38626800	-2.91270700	H	0.39756400	-0.92907300	2.68544000
C	2.84431400	2.31085700	0.01717000	C	-3.12718700	1.58589500	-0.01315000
C	2.70186900	2.95792100	-1.21660300	C	-3.17999100	2.25222000	1.21726300
C	2.37467100	4.31518100	-1.19804800	C	-3.28111600	3.64384600	1.18287100
H	2.26248800	4.85085500	-2.13703200	H	-3.31791500	4.19931000	2.11571600
C	2.19846100	4.98957100	0.00522600	C	-3.32443300	4.32674300	-0.02708800
H	1.94916300	6.04743300	0.00062800	H	-3.39870500	5.41056500	-0.03260500
C	2.34841100	4.31950200	1.21446200	C	-3.26860700	3.63248000	-1.23004800
H	2.21609400	4.85875100	2.14875300	H	-3.29557700	4.17913000	-2.16841300
C	2.67454500	2.96214600	1.24510900	C	-3.16714400	2.24062300	-1.25029300
C	2.91501800	2.22793600	-2.52541800	C	-3.09951300	1.51925000	2.53976000
H	3.06446700	1.16782600	-2.29761600	H	-3.06367400	0.44347500	2.33432500
C	1.68988700	2.32441500	-3.43466400	C	-1.81222400	1.88174500	3.28424800
H	1.85207500	1.74609800	-4.35148400	H	-1.73687900	1.30982300	4.21602900
H	1.48032600	3.35994800	-3.72723900	H	-1.78821600	2.94732400	3.53937300
H	0.80126500	1.92488200	-2.93196200	H	-0.93002600	1.66124500	2.67293400
C	4.18544300	2.73017200	-3.21501500	C	-4.34148500	1.77609700	3.39487400
H	4.36530500	2.17100400	-4.14042400	H	-4.28722800	1.19860400	4.32441600
H	5.05253200	2.60231200	-2.55887600	H	-5.25295600	1.48694100	2.86110000
H	4.10314000	3.79298100	-3.47226100	H	-4.42876400	2.83435700	3.66554600
C	2.85892200	2.23641700	2.56056100	C	-3.07310600	1.49532200	-2.56503600
H	3.00393200	1.17410000	2.33973500	H	-3.03792100	0.42149000	-2.34936500
C	1.61942300	2.34664600	3.44847400	C	-1.77923300	1.85236600	-3.30064900
H	1.76136500	1.77062100	4.37004900	H	-1.69454200	1.27196000	-4.22636300
H	0.73588300	1.95283300	2.93245000	H	-0.90265200	1.63838800	-2.67902600
H	1.41364800	3.38489100	3.73393000	H	-1.75385700	2.91556400	-3.56538100
C	4.12133000	2.73239600	3.26924000	C	-4.30731100	1.74257600	-3.43412000
H	4.28147200	2.17632600	4.20013600	H	-4.24351400	1.15680300	-4.35785600
H	4.04207600	3.79699300	3.52009000	H	-4.39350900	2.79823300	-3.71510400
H	4.99814700	2.59513200	2.62813200	H	-5.22333200	1.45689400	-2.90628800
C	-2.63486500	-0.92214900	-0.02126800	C	3.04744400	0.58340000	0.01230500
C	-2.18817400	-2.24746500	-0.03679000	C	3.24234000	-0.80473600	0.01384000
H	-1.12811900	-2.48268600	-0.04837700	H	2.39372700	-1.48508200	0.01546700

C	-3.15188800	-3.25039000	-0.03885500
H	-2.84446700	-4.28964100	-0.05440400
C	-4.51080600	-2.92980700	-0.02792700
C	-4.96170900	-1.59566300	-0.01535900
H	-6.02547100	-1.38245800	-0.01585300
C	-4.01935300	-0.59137700	-0.01041400
C	-4.06629600	0.86535000	-0.00295900
C	-5.07220700	1.80501800	0.01409800
H	-6.11984000	1.52175400	0.02747200
C	-4.71022000	3.16618000	0.02229900
C	-3.37588500	3.57616900	0.01682300
H	-3.14244200	4.63425600	0.02824800
C	-2.34827300	2.63798300	0.00353100
H	-1.30423900	2.93572900	0.00263500
C	-2.70466200	1.28640000	-0.00724700
C	-5.53520000	-4.02752800	0.02955900
C	-5.81210400	4.18721200	-0.01026000
F	-5.94642700	-4.23493800	1.29125300
F	-5.05299300	-5.18870600	-0.43068500
F	-6.62289400	-3.71924200	-0.69324100
F	-6.80830800	3.85203800	0.82446500
F	-5.38144100	5.40653000	0.33450000
F	-6.34484800	4.27913000	-1.23973800

C	4.53936000	-1.28414500	0.01739500
H	4.71614700	-2.35600400	0.02446700
C	5.63988900	-0.40458500	0.02023600
C	5.45531300	0.97311100	0.01767400
H	6.31299200	1.63874700	0.02167700
C	4.15595800	1.47736300	0.01512400
C	3.59084300	2.80355500	0.01667800
C	4.12582300	4.09462000	0.01954100
H	5.20275500	4.24564000	0.02191200
C	3.26226800	5.18193800	0.01941600
C	1.86945700	4.99085500	0.01619500
H	1.21337200	5.85768900	0.01603200
C	1.31359900	3.71905400	0.01322400
H	0.23529900	3.57183500	0.01068500
C	2.18083600	2.62137000	0.01365100
C	7.01077100	-0.98841600	-0.01678300
F	7.29716500	-1.54659100	-1.21386400
F	7.16619700	-1.97365300	0.89291100
F	7.97005600	-0.07690500	0.22162900
H	3.66425800	6.19131600	0.02177900

Optimized **Au-1^{CF3}** S_1 semi-coplanar

Au	0.05753400	0.51442700	-0.03188900
N	-1.90947700	-1.73012900	-0.14027200
N	-2.95169300	0.15101200	0.11794400
N	-3.83106300	-3.25182400	-0.17674300
N	-5.25877900	-0.67741200	0.17461000
N	1.96294000	1.34272000	-0.05939200
C	-1.71700600	-0.39235300	-0.01403800
C	-3.26003300	-2.04204000	-0.08897900
C	-3.93057300	-0.83229600	0.07583600
C	-5.85792200	-1.90868100	0.08625000
H	-6.94284500	-1.91071000	0.15395000
C	-5.19223900	-3.10932400	-0.07681400
H	-5.76793400	-4.02952400	-0.13415900
C	-0.82069200	-2.64052700	-0.27760200
C	-0.36362000	-2.94634900	-1.56526300
C	0.74682700	-3.78618700	-1.67270300
H	1.12741800	-4.04568200	-2.65695200
C	1.36888500	-4.29456400	-0.53779200
H	2.23046300	-4.94905300	-0.64087000
C	0.88813100	-3.97709200	0.72838000
H	1.37602600	-4.38811800	1.60833800
C	-0.21963300	-3.14070900	0.88486800
C	-1.04665000	-2.39941500	-2.80018700
H	-1.83140400	-1.71036700	-2.47183400
C	-1.72848300	-3.52689600	-3.57815700
H	-0.99757000	-4.26231800	-3.93503500
H	-2.25606300	-3.12415000	-4.45033800
H	-2.45490200	-4.04441600	-2.94324200
C	-0.07802200	-1.60063800	-3.67291400
H	0.73253400	-2.22917500	-4.05996200
H	0.37087800	-0.77868800	-3.10300000
H	-0.60695300	-1.17243900	-4.53211800
C	-0.75337500	-2.80953400	2.26230000
H	-1.54420800	-2.06155200	2.14576500
C	-1.38863800	-4.04826400	2.89799100
H	-2.19199800	-4.43529000	2.26276400
H	-1.81138900	-3.80036000	3.87837700
H	-0.64842200	-4.84475000	3.03985900
C	0.32333100	-2.19289200	3.15545900
H	1.14776700	-2.89121600	3.34117000

Optimized **Au-1^{CF3}** S_1 orthogonal

Au	0.18825500	0.00871800	0.38565000
N	-2.50097900	1.07193500	-0.47135800
N	-2.50650000	-1.09560100	-0.39365300
N	-4.81372200	1.44782700	-1.17662400
N	-4.82118600	-1.50982300	-1.07004700
N	2.16002200	0.02326800	0.99857900
C	-1.71147700	-0.00532600	-0.19396400
C	-3.77394400	0.67567800	-0.83811600
C	-3.77759500	-0.72037300	-0.78738300
C	-5.89750500	-0.72870900	-1.41971800
H	-6.80722400	-1.26733900	-1.67141700
C	-5.89394400	0.64908300	-1.46940700
H	-6.80116800	1.17278200	-1.75888300
C	-2.04032300	2.41688500	-0.37625200
C	-2.18469100	3.08753600	0.84515600
C	-1.71851900	4.40133600	0.91787000
H	-1.82129200	4.95563800	1.84676500
C	-1.13145300	5.01136500	-0.18514800
H	-0.77784200	6.03637500	-0.11135100
C	-0.99917000	4.31895500	-1.38332200
H	-0.54419400	4.80890100	-2.23995500
C	-1.45125700	3.00341900	-1.50322500
C	-2.85753100	2.42912600	2.03073400
H	-2.94389200	1.35939700	1.81248100
C	-4.27596500	2.97928500	2.20267200
H	-4.25358900	4.05280100	2.42645600
H	-4.78797900	2.47039600	3.02780400
H	-4.85748900	2.83092500	1.28705000
C	-2.03536500	2.56202200	3.31185600
H	-1.94782900	3.60524300	3.63651200
H	-1.02300000	2.16623000	3.17006300
H	-2.51312100	2.00353200	4.12505200
C	-1.34121200	2.25757600	-2.81591300
H	-1.59636300	1.20970600	-2.62676200
C	-2.35879900	2.80369600	-3.82074100
H	-3.37346200	2.73143400	-3.41611600
H	-2.31624800	2.23583500	-4.75752100
H	-2.15540900	3.85631700	-4.05193000
C	0.08151500	2.28093100	-3.37358800
H	0.40083700	3.29532200	-3.63905400

H	-0.10304800	-1.91654500	4.12649800	H	0.14143600	1.66811700	-4.28045500
H	0.73926800	-1.28823700	2.69658100	H	0.79355300	1.88347800	-2.64104400
C	-3.16086700	1.55143600	0.27925700	C	-2.05081200	-2.43224900	-0.20426000
C	-3.21082800	2.07178200	1.57810300	C	-1.46707600	-3.10006900	-1.28843500
C	-3.37559300	3.45163500	1.71267300	C	-1.02180500	-4.40612800	-1.07625900
H	-3.42364500	3.88976800	2.70608600	H	-0.57147700	-4.95860700	-1.89633500
C	-3.48182200	4.26947400	0.59318500	C	-1.15494700	-5.01096300	0.16859200
H	-3.61275900	5.34123800	0.71725900	H	-0.80673600	-6.03014400	0.31409100
C	-3.42956500	3.72220100	-0.68416600	C	-1.73568500	-4.32118100	1.22685400
H	-3.52213400	4.37009800	-1.55197600	H	-1.83907700	-4.80755900	2.19300500
C	-3.26894100	2.34725000	-0.86781800	C	-2.19525300	-3.01329900	1.06185900
C	-3.10477100	1.17954500	2.79624500	C	-1.36236900	-2.44800000	-2.65069300
H	-2.90028500	0.16118100	2.45043400	H	-1.56238300	-1.37874400	-2.52406100
C	-1.94070200	1.59240100	3.69748500	C	0.03729900	-2.57480100	-3.25020300
H	-1.85264400	0.90094800	4.54337300	H	0.09391100	-2.01918000	-4.19332400
H	-2.08011000	2.60075500	4.10450700	H	0.29896700	-3.61688200	-3.46676500
H	-0.99516500	1.57671800	3.14300200	H	0.79369400	-2.17145100	-2.56683200
C	-4.43510300	1.14396600	3.55131800	C	-2.43756200	-3.00947100	-3.58533900
H	-4.36989300	0.46436200	4.40878900	H	-2.40193300	-2.50558600	-4.55835700
H	-5.23748000	0.79542000	2.89313100	H	-3.43371100	-2.86557700	-3.15436300
H	-4.70492600	2.13804400	3.92748800	H	-2.28688200	-4.08299600	-3.75217000
C	-3.23372100	1.74882800	-2.25789600	C	-2.86249200	-2.26877100	2.19849300
H	-3.02524300	0.67882800	-2.15819400	H	-2.94737400	-1.21767100	1.90306400
C	-2.11119500	2.35086600	-3.10358900	C	-2.03623600	-2.31015000	3.48319600
H	-2.07681900	1.86911000	-4.08747800	H	-2.51046400	-1.69351400	4.25543000
H	-1.13868600	2.20502200	-2.61864600	H	-1.02382700	-1.92721800	3.30987300
H	-2.25558800	3.42585000	-3.26351900	H	-1.94909900	-3.32733200	3.88212800
C	-4.59966900	1.88516500	-2.93388800	C	-4.28169200	-2.80100700	2.41461900
H	-4.58667600	1.41045600	-3.92172600	H	-4.78908400	-2.23273300	3.20302000
H	-4.87341900	2.93843200	-3.06837500	H	-4.26147900	-3.85577800	2.71462400
H	-5.37561500	1.40440100	-2.32929800	H	-4.86658200	-2.71689900	1.49302800
C	3.12076800	0.61290700	0.06037100	C	3.26811400	-0.01734100	0.17828700
C	3.23814600	-0.76785100	0.22750900	C	3.28871800	-0.08876900	-1.21253900
H	2.35921500	-1.40445500	0.27685100	H	2.36414200	-0.11974500	-1.77934400
C	4.52222400	-1.29919900	0.32514500	C	4.53164600	-0.12118500	-1.84284700
H	4.65229900	-2.36704700	0.45938300	H	4.58740600	-0.18010100	-2.92356400
C	5.64338300	-0.47086400	0.25621700	C	5.70736100	-0.08589200	-1.09436300
C	5.52512900	0.92034000	0.08446400	C	5.68801700	-0.01759100	0.31049900
H	6.41508100	1.53981100	0.03749900	H	6.61925700	-0.00014200	0.86748500
C	4.26061200	1.46147500	-0.01499700	C	4.46460400	0.01727500	0.94374300
C	3.72927300	2.80372700	-0.19484700	C	4.03281900	0.08115100	2.33288300
C	4.27555100	4.06179200	-0.33712800	C	4.67510100	0.13749400	3.55079300
H	5.35007200	4.21683900	-0.32657400	H	5.75825200	0.13938300	3.62091600
C	3.40608500	5.15669200	-0.49868400	C	3.89117500	0.19367100	4.71854100
C	2.01394800	4.99660800	-0.51622200	C	2.49181600	0.19391300	4.66708800
H	1.37857900	5.86690700	-0.64139900	H	1.92333500	0.23833400	5.58965600
C	1.44056900	3.74167100	-0.37322400	C	1.82655300	0.13826400	3.45070500
H	0.36451600	3.59503700	-0.37892000	H	0.74355500	0.13736100	3.38515900
C	2.30663200	2.64401600	-0.21398000	C	2.60511200	0.08168900	2.28242300
C	7.01773000	-1.07064000	0.30349400	C	7.03869300	-0.06580200	-1.78836600
F	7.51432600	-1.25104000	-0.93307200	F	7.53154200	1.18267700	-1.85291400
F	7.02590000	-2.26485600	0.91177800	F	6.96143800	-0.53048400	-3.04194500
F	7.88006000	-0.27420000	0.95641300	F	7.94329600	-0.80911000	-1.13095400
H	3.82707200	6.15062700	-0.61131200	H	4.38663800	0.23832400	5.68289500
Optimized **Au-1**$^{\mathbf{CF3}}$ T_1 semi-coplanar				Optimized **Cu-1**$^{\mathbf{FLR}}$ S_0 semi-coplanar			
Au	-0.02680600	0.56236000	-0.00200900	Cu	-0.55511200	0.58388100	-0.22012500
N	1.82143300	-1.77253900	-0.00945600	N	0.88627100	1.73040600	-0.27796300
N	2.97380600	0.06503200	0.00391600	N	-3.30682000	-0.14294600	0.14599700
N	3.65339000	-3.39348300	-0.01447400	N	-2.01170300	-1.89796500	0.07466800
N	5.22921600	-0.88512500	0.00314500	N	-5.42154800	-1.28951800	0.58072900
N	-1.89781900	1.41379000	-0.00542900	N	-3.71642300	-3.60261000	0.47757200
C	1.69956800	-0.41369600	-0.00166700	C	0.84194800	3.10901500	-0.26308600
C	3.14995900	-2.15343100	-0.00862100	C	-0.28461500	3.93889200	-0.29675100
C	3.89337100	-0.96851700	-0.00022500	H	-1.28552300	3.51436500	-0.34455800

C	5.75985900	-2.15286700	-0.00324100	C	-0.09058600	5.31306100	-0.26412100
H	6.84533900	-2.20854300	-0.00142500	H	-0.95532900	5.97208300	-0.28741200
C	5.02606200	-3.32157400	-0.01128100	C	1.19956700	5.86796700	-0.20108100
H	5.54737200	-4.27529500	-0.01570600	H	1.31819400	6.94795700	-0.17764600
C	0.68423500	-2.63169900	-0.01570400	C	2.32120300	5.04839600	-0.16892300
C	0.13154500	-3.01861900	1.21216000	H	3.31773400	5.48183000	-0.11945300
C	-1.01930600	-3.80958000	1.18211300	C	2.15209700	3.66144500	-0.19833000
H	-1.47272700	-4.12890400	2.11678600	C	3.05099200	2.53383200	-0.17029500
C	-1.58859600	-4.19534600	-0.02689100	C	4.43968400	2.39852500	-0.10771700
H	-2.48140200	-4.81540600	-0.03135400	H	5.08507500	3.27387300	-0.07445900
C	-1.01667600	-3.79611700	-1.23026500	C	4.96974600	1.11396400	-0.08792300
H	-1.46817300	-4.10487000	-2.16940800	C	6.35686200	0.65669600	-0.02812100
C	0.13431000	-3.00501000	-1.24899600	C	7.55390900	1.36934900	0.02429400
C	0.74921600	-2.60084200	2.52932900	H	7.55331900	2.45664700	0.02492800
H	1.60947700	-1.96131000	2.30829500	C	8.75696100	0.66667700	0.07585100
C	1.27082700	-3.81920300	3.29326200	H	9.69631400	1.21222700	0.11667600
H	0.45700200	-4.50660500	3.55299600	C	8.76890900	-0.72883000	0.07485500
H	1.75683100	-3.50578800	4.22421600	H	9.71537600	-1.26139100	0.11470600
H	2.00193400	-4.36628400	2.68945900	C	7.56916100	-1.44394900	0.02246100
C	-0.22833800	-1.77327900	3.36504400	H	7.58179800	-2.53204500	0.02229000
H	-1.11944300	-2.35181900	3.63597300	C	6.37031000	-0.75027100	-0.02869300
H	-0.55307800	-0.88408500	2.81192100	C	4.95665600	-1.30322300	-0.08269400
H	0.25074300	-1.44180100	4.29349300	C	4.13060300	-0.02846100	-0.12705000
C	0.75487000	-2.57265500	-2.56008700	C	2.75613300	0.08284500	-0.19300300
H	1.61532100	-1.93657800	-2.33000400	H	2.10835100	-0.79298300	-0.22389700
C	1.27651400	-3.78255600	-3.33729400	C	2.21348400	1.37712600	-0.21717700
H	2.00559300	-4.33783700	-2.73856000	C	4.73244300	-2.15446600	-1.33838900
H	1.76487300	-3.45879100	-4.26345800	H	4.96728300	-1.58289600	-2.24188600
H	0.46234300	-4.46573500	-3.60685900	H	3.68690700	-2.47802300	-1.39754800
C	-0.22035000	-1.73452600	-3.38801500	H	5.36754800	-3.04843000	-1.32057200
H	-1.11142700	-2.30914000	-3.66730100	C	4.63639100	-2.12159600	1.17495500
H	0.26081000	-1.39273200	-4.31164300	H	4.80071400	-1.52511200	2.07808100
H	-0.54536900	-0.85151600	-2.82522400	H	5.27147700	-3.01406100	1.23004400
C	3.26309300	1.45995200	0.01585600	H	3.58972000	-2.44479500	1.16059000
C	3.37972800	2.12808500	-1.20914400	C	-2.01710100	-0.54184700	-0.02415600
C	3.61821500	3.50339500	-1.17276700	C	-4.12282200	-1.23588800	0.36383100
H	3.71876400	4.05335700	-2.10489100	C	-3.28936200	-2.36695600	0.31328700
C	3.73389100	4.17502100	0.03936200	C	-5.86779300	-2.54160700	0.74997500
H	3.92432000	5.24504100	0.04864100	H	-6.93157900	-2.65889200	0.93465200
C	3.61484000	3.48309500	1.23970300	C	-5.03625300	-3.66994700	0.69910700
H	3.71283800	4.01729900	2.18120000	H	-5.45627100	-4.66097800	0.84374500
C	3.37574300	2.10749500	1.25231800	C	-3.68837700	1.23856300	0.13019800
C	3.26209400	1.39514300	-2.52845500	C	-4.16474300	1.77906000	-1.07244100
H	3.02507300	0.34893200	-2.31056700	C	-4.47084700	3.13998200	-1.08278700
C	2.11853200	1.95057000	-3.37834000	H	-4.83105300	3.60093300	-1.99733900
H	2.02124400	1.37603100	-4.30676500	C	-4.30961100	3.91669900	0.05963200
H	2.28850400	2.99899600	-3.65052400	H	-4.55036300	4.97565200	0.03025000
H	1.16723900	1.88789900	-2.83695700	C	-3.83446900	3.34927200	1.23488600
C	4.59608200	1.41415200	-3.27715800	H	-3.70397700	3.96919900	2.11715800
H	4.51835600	0.84539500	-4.21086000	C	-3.50761700	1.99270400	1.29683500
H	5.38528700	0.96658200	-2.66456900	C	-4.26983100	0.94363100	-2.33166100
H	4.89664900	2.43799200	-3.52977600	H	-4.27145900	-0.11282800	-2.04020400
C	3.25390200	1.35246000	2.55874600	C	-5.56567600	1.19597000	-3.10071700
H	3.00957000	0.31183800	2.32293900	H	-5.63799100	0.50316500	-3.94605000
C	2.11470100	1.90008800	3.41941400	H	-5.60649500	2.21189200	-3.50867100
H	2.01374800	1.30880800	4.33686100	H	-6.44148600	1.04972200	-2.46012000
H	1.16276200	1.85441000	2.87751400	C	-3.04055500	1.17673900	-3.21612600
H	2.29205400	2.94189900	3.71145700	H	-2.11352300	0.94947100	-2.67762400
C	4.58875000	1.34953000	3.30628200	H	-2.98895300	2.22255400	-3.54025200
H	4.50799600	0.76552000	4.23026800	H	-3.08258400	0.54307900	-4.10958800
H	4.89630800	2.36698400	3.57580100	C	-2.97485400	1.39558100	2.58255000
H	5.37428500	0.90712800	2.68526900	H	-2.72519500	0.34519000	2.39710700
C	-3.05999500	0.67234900	-0.00783400	C	-4.04353200	1.42414900	3.67766900
C	-3.17323400	-0.71703400	-0.01040000	H	-3.66522000	0.95109000	4.59081200
H	-2.29430900	-1.35487400	-0.00999800	H	-4.94675500	0.89184300	3.36054500

C	-4.45747000	-1.25870200	-0.01588300
H	-4.58452600	-2.33506900	-0.02144000
C	-5.57844000	-0.42943100	-0.02115000
C	-5.46262400	0.97324200	-0.02054000
H	-6.35336500	1.59332100	-0.03221900
C	-4.19847000	1.52241000	-0.01254400
C	-3.66750600	2.87911600	-0.01387600
C	-4.21926400	4.14246000	-0.01753400
H	-5.29473700	4.29054900	-0.02117500
C	-3.35220400	5.25103600	-0.01630100
C	-1.96012200	5.09826000	-0.01139700
H	-1.32647400	5.97862600	-0.01063400
C	-1.38334300	3.83544800	-0.00745400
H	-0.30691000	3.69226500	-0.00374300
C	-2.24675100	2.72768300	-0.00868000
C	-6.95560500	-1.02483200	0.02264500
F	-7.47830200	-0.94738600	1.25858600
F	-6.96077800	-2.31598800	-0.33326400
F	-7.79614600	-0.37162700	-0.79654100
H	-3.77584500	6.25015700	-0.01917400

H	-4.32778800	2.45301600	3.92625700
C	-1.68786500	2.09348600	3.02832000
H	-1.28653000	1.60753900	3.92462000
H	-1.86582000	3.14724100	3.26985700
H	-0.92125000	2.05578200	2.24629100
C	-0.81598300	-2.68025300	-0.02818300
C	-0.10960000	-2.96819300	1.14729300
C	1.06319000	-3.71379500	1.02010300
H	1.64044900	-3.95646600	1.90768200
C	1.50944400	-4.13708700	-0.22667400
H	2.42863000	-4.71086300	-0.30445500
C	0.79498400	-3.81699400	-1.37479700
H	1.16314700	-4.13981100	-2.34453200
C	-0.38859700	-3.07978000	-1.30085300
C	-0.54717700	-2.45090800	2.50129200
H	-1.53836000	-1.99689500	2.39195400
C	0.40819500	-1.35371700	2.98074200
H	1.42350000	-1.74477200	3.11336200
H	0.07246000	-0.94736300	3.94162500
H	0.46181000	-0.52908000	2.26071700
C	-0.67765300	-3.57610100	3.52803200
H	0.28824000	-4.05256100	3.72928800
H	-1.37138200	-4.34832200	3.17942100
H	-1.05269500	-3.17777300	4.47714500
C	-1.13882800	-2.70654500	-2.56168500
H	-2.05361000	-2.17720100	-2.27342700
H	0.62148800	-2.21976100	-3.75415600
C	-0.30660000	-1.74577000	-3.41496800
H	-0.03545900	-0.84778200	-2.84825400
H	-0.87126000	-1.43445200	-4.30089100
C	-1.55970400	-3.94641700	-3.35212100
H	-0.68959800	-4.51221900	-3.70357800
H	-2.17249000	-4.61498400	-2.73828500
H	-2.14304700	-3.65390400	-4.23221700

Optimized **Cu-1$^{\mathbf{FLR}}$** S_1 semi-coplanar

Cu	0.49983512	0.63685950	0.00878243
N	-0.99955749	1.79239858	0.00682386
N	2.03431281	-1.85178537	-0.00676129
N	3.29760024	-0.10008810	0.00455045
N	3.76340598	-3.59224992	-0.02895921
N	5.49589896	-1.18791915	-0.02671829
C	-0.97297809	3.17828076	0.01357747
C	0.14992791	4.00105413	0.01845695
H	1.15320418	3.58247445	0.01760713
C	-0.06094181	5.38149291	0.02491576
H	0.79596762	6.04837133	0.02876783
C	-1.35125912	5.91547746	0.02654777
H	-1.48494450	6.99291653	0.03158019
C	-2.47844212	5.08293477	0.02183367
H	-3.47657573	5.51231695	0.02325252
C	-2.28885162	3.70918196	0.01542922
C	-3.17033122	2.55479262	0.00970740
C	-4.53640373	2.37406905	0.00880817
H	-5.22297517	3.21608605	0.01268606
C	-5.01756050	1.05092819	0.00291267
C	-6.37941801	0.56575383	0.00093553
C	-7.59153813	1.26427254	0.00436410
H	-7.60869914	2.35053760	0.00922612
C	-8.77560826	0.53896550	0.00155012
H	-9.72850774	1.05963906	0.00418024
C	-8.75291741	-0.86066363	-0.00457842
H	-9.69006931	-1.40971253	-0.00664629
C	-7.54401177	-1.55981240	-0.00809855
H	-7.54120300	-2.64663987	-0.01289594

Optimized **Cu-1$^{\mathbf{FLR}}$** S_1 orthogonal

Cu	-0.44664695	0.66506209	-0.43025496
N	1.16541886	1.57542642	-0.81428880
N	-2.84136835	0.02167694	1.08923689
N	-2.56934596	-1.35783899	-0.55587705
N	-4.87479295	-0.92328078	2.07203153
N	-4.51732854	-2.79556719	-0.19403179
C	1.31492313	2.87037405	-1.29975951
C	0.31219916	3.74570305	-1.70259019
H	-0.73253147	3.45508244	-1.65904604
C	0.69518166	5.00965524	-2.15771350
H	-0.06706154	5.71226075	-2.47965776
C	2.03858113	5.38284739	-2.20149355
H	2.30850518	6.37214505	-2.55762879
C	3.04716359	4.50074036	-1.78932243
H	4.08958744	4.80444981	-1.82408722
C	2.68207902	3.24295655	-1.33916179
C	3.41221835	2.08894186	-0.84142713
C	4.74217548	1.80916037	-0.61880114
H	5.52064207	2.53805335	-0.82524365
C	5.06061139	0.53652599	-0.10702853
C	6.34979996	-0.02505563	0.23266957
C	7.63007340	0.53220065	0.15589817
H	7.77687770	1.54465506	-0.20957713
C	8.71317138	-0.23774677	0.55866174
H	9.71702459	0.17305611	0.50884650
C	8.52262190	-1.54177071	1.03049217
H	9.38276068	-2.12754681	1.34142684
C	7.24518019	-2.09994338	1.10677391
H	7.11285101	-3.11407300	1.47431872

C	-6.35737317	-0.84507956	-0.00539529	C	6.15828944	-1.33908179	0.70734362
C	-4.93405773	-1.36835017	-0.00860219	C	4.69031253	-1.71935009	0.68511841
C	-4.14149694	-0.07435574	-0.00237125	C	4.06113419	-0.44108158	0.16301279
C	-2.77944261	0.08301168	-0.00183839	C	2.73199190	-0.17612803	-0.05060389
H	-2.09192897	-0.75995575	-0.00674325	H	1.95262308	-0.90660768	0.14283364
C	-2.28640889	1.41096104	0.00461322	C	2.39996929	1.10510612	-0.54833398
C	-4.64054033	-2.20220016	1.24611380	C	4.44385969	-2.88860890	-0.27920297
H	-4.84667042	-1.62918064	2.15559213	H	4.80545758	-2.65181548	-1.28458290
H	-3.58984622	-2.51051640	1.25976215	H	3.37406129	-3.11398132	-0.34087213
H	-5.26153023	-3.10487166	1.25807900	H	4.96084933	-3.78792779	0.07239768
C	-4.64178366	-2.18840640	-1.27270190	C	4.17058546	-2.06621174	2.08612780
H	-4.84818554	-1.60523889	-2.17565993	H	4.33020066	-1.23722752	2.78252233
H	-5.26293347	-3.09076606	-1.29427942	H	4.68435466	-2.95131521	2.47631488
H	-3.59113964	-2.49653162	-1.28991743	H	3.09782306	-2.28561289	2.05103350
C	1.99520336	-0.49260405	0.00562008	C	-2.01241782	-0.25595252	0.03636421
C	3.33972436	-2.32017095	-0.01659268	C	-3.88644653	-0.87983039	1.16922349
C	4.15547668	-1.18888425	-0.01277566	C	-3.71950282	-1.76104847	0.09894750
C	5.13578030	-3.60634317	-0.04077037	C	-5.70407396	-1.98129270	1.78166148
H	5.59695568	-4.59077679	-0.05119818	H	-6.54761959	-2.11588295	2.45407000
C	5.94348666	-2.48473220	-0.04090198	C	-5.53770688	-2.85318319	0.72665389
H	7.02332861	-2.61080263	-0.05250807	H	-6.25243256	-3.66026741	0.58654666
C	0.84910450	-2.64200011	-0.00517432	C	-2.58510287	1.08839927	1.99906193
C	0.26432997	-2.97676143	-1.23328585	C	-3.25360568	2.30715140	1.81360622
C	-0.91116881	-3.73072683	-1.20657040	C	-2.94329853	3.35282215	2.68392001
H	-1.38684809	-4.01249531	-2.14248592	H	-3.43641922	4.31319487	2.56684544
C	-1.47428636	-4.13094726	0.00016115	C	-2.01411185	3.18060838	3.70471224
H	-2.38491710	-4.72437550	0.00213456	H	-1.78791021	4.00624212	4.37429592
C	-0.87714193	-3.77491622	1.20483271	C	-1.38438964	1.95496236	3.87987650
H	-1.32577913	-4.09355085	2.14202872	H	-0.67496457	1.82593159	4.69290896
C	0.29677066	-3.01878554	1.22641213	C	-1.65945457	0.88086514	3.03015894
C	0.86996034	-2.53894503	-2.54932318	C	-4.24419784	2.48671504	0.68067826
H	1.77121760	-1.95922972	-2.32717031	H	-4.62958659	1.49584034	0.41970506
C	1.29818026	-3.74700167	-3.38377301	C	-5.44479579	3.34625972	1.07024131
H	1.77789399	-3.41764458	-4.31247197	H	-6.18209458	3.34304022	0.25997429
H	0.43943278	-4.37338296	-3.65304439	H	-5.16316798	4.39044379	1.24981461
H	2.01084367	-4.36425495	-2.82731335	H	-5.92819788	2.96020103	1.97339603
C	-0.08530299	-1.62162119	-3.31527628	C	-3.53955599	3.05128988	-0.55673376
H	-0.34635871	-0.74177700	-2.71532528	H	-2.72811947	2.38611949	-0.87379040
H	-1.01536951	-2.13900038	-3.57948427	H	-3.11271983	4.03936827	-0.34369452
H	0.38122887	-1.27388554	-4.24412804	H	-4.24349188	3.15372072	-1.39138525
C	0.95410977	-2.64239264	2.53661812	C	-1.01829001	-0.47153922	3.25910636
H	1.76007053	-1.93575522	2.31455374	H	-1.24299029	-1.09837217	2.39132480
C	1.58996532	-3.87416877	3.18513184	C	-1.64162931	-1.14641827	4.48382376
H	2.10402701	-3.59636833	4.11252266	H	-1.21947950	-2.14821984	4.62659985
H	2.31919258	-4.32916410	2.50688718	H	-2.72519584	-1.24168449	4.35930974
H	0.83104359	-4.62696110	3.43041413	H	-1.45180377	-0.56456987	5.39389835
C	-0.02052273	-1.93410789	3.47767429	C	0.50303580	-0.38277856	3.37215742
H	0.49872247	-1.61351228	4.38794905	H	0.93440445	-1.38704276	3.45823163
H	-0.84545330	-2.58954399	3.78047837	H	0.81585127	0.18644043	4.25523112
H	-0.45078437	-1.04604129	3.00002448	H	0.93644058	0.10133396	2.48947674
C	3.65123084	1.28082361	0.00762412	C	-2.01278827	-1.96662111	-1.71825915
C	3.76978049	1.93984856	1.23841392	C	-1.28105570	-3.15603096	-1.57143910
C	4.02702460	3.31227292	1.21466728	C	-0.70022505	-3.70201815	-2.71598437
H	4.11731070	3.85537448	2.15167463	H	-0.12584645	-4.62021545	-2.64093033
C	4.16594559	3.99153036	0.00910824	C	-0.85127762	-3.09032095	-3.95687539
H	4.36732538	5.05957694	0.00982861	H	-0.39103583	-3.53207739	-4.83676055
C	4.05176606	3.30910816	-1.19711186	C	-1.59992021	-1.92773702	-4.07663891
H	4.16364003	3.84882372	-2.13384747	H	-1.72968490	-1.46888434	-5.05336591
C	3.79060056	1.93747553	-1.22190288	C	-2.20309851	-1.34457403	-2.95870757
C	3.59817324	1.20668189	2.55191136	C	-1.09917673	-3.79308385	-0.20722921
H	3.48600618	0.14114694	2.32784697	H	-1.98430233	-3.55194974	0.38959974
C	2.32452345	1.66261183	3.26738505	C	0.12149160	-3.19488458	0.49939093
H	2.36835852	2.72911895	3.51882214	H	1.03573052	-3.41431095	-0.06650207
H	2.18460256	1.10065392	4.19820477	H	0.23339014	-3.61471808	1.50639429
H	1.44300138	1.50053456	2.63556720	H	0.02390542	-2.10729638	0.59076167

C	4.83324734	1.35626920	3.44077972
H	5.00117994	2.40103272	3.72738216
H	5.72793848	0.99587309	2.92311857
H	4.71017712	0.77405616	4.36099807
C	3.65289351	1.19920873	-2.53609911
H	3.43832727	0.15024578	-2.30976970
H	2.62581908	2.79018351	-3.62678161
C	2.47755978	1.73893576	-3.35284584
H	1.54249231	1.66610249	-2.78493806
H	2.35987615	1.16406368	-4.27871532
C	4.96431409	1.23475375	-3.32223525
H	5.24648821	2.26099348	-3.58570088
H	5.77576859	0.79657440	-2.73220852
H	4.86668627	0.66350450	-4.25246193
C	-1.00169231	-5.31553181	-0.26203454
H	-0.07653469	-5.65287490	-0.74433952
H	-1.84971266	-5.74619768	-0.80413150
H	-1.00479917	-5.72294463	0.75479354
C	-3.06344032	-0.10765819	-3.11380258
H	-3.37827366	0.21077040	-2.11576323
H	-1.96231198	0.81843754	-4.75965309
C	-2.28476753	1.05057584	-3.73786873
H	-1.38935587	1.28299282	-3.14930324
H	-2.90942934	1.95031791	-3.78109570
C	-4.32755789	-0.43122441	-3.91359918
H	-4.08251007	-0.74964230	-4.93387528
H	-4.89457689	-1.23579944	-3.43428381
H	-4.97291626	0.45232208	-3.98183346

Optimized **Cu-1$^{\mathbf{FLR}}$** T_1 semi-coplanar

Cu	0.49199119	0.62296720	-0.00059833
N	-0.98690113	1.76932899	-0.00105214
N	2.02786750	-1.86499835	0.00101414
N	3.28431543	-0.10520022	0.00116387
N	3.76277457	-3.59243213	0.00294462
N	5.48323882	-1.18184741	0.00321747
C	-0.94871468	3.15689017	-0.00108636
C	0.18240251	3.96753041	-0.00107942
H	1.18161791	3.53990129	-0.00100715
C	-0.01603171	5.34988996	-0.00107657
H	0.84631014	6.00931747	-0.00105701
C	-1.30161461	5.89315735	-0.00108829
H	-1.42649209	6.97160339	-0.00108786
C	-2.43792143	5.07074382	-0.00109151
H	-3.43170061	5.50954057	-0.00108626
C	-2.25936566	3.69735172	-0.00108582
C	-3.15245250	2.54741739	-0.00104162
C	-4.52021083	2.38151652	-0.00102558
H	-5.19824758	3.23022984	-0.00106835
C	-5.01352997	1.06225927	-0.00094211
C	-6.38204770	0.58913113	-0.00093183
C	-7.58668478	1.29858431	-0.00103064
H	-7.59434833	2.38498614	-0.00112376
C	-8.77757246	0.58368735	-0.00101286
H	-9.72575919	1.11284705	-0.00109150
C	-8.76699971	-0.81575149	-0.00089792
H	-9.70891410	-1.35659793	-0.00088873
C	-7.56464349	-1.52551537	-0.00079414
H	-7.57134864	-2.61232040	-0.00070187
C	-6.37155708	-0.82076562	-0.00081137
C	-4.95274817	-1.35604280	-0.00067994
C	-4.14999873	-0.06878894	-0.00084411
C	-2.78445540	0.07780706	-0.00088404
H	-2.10427250	-0.77098349	-0.00079577
C	-2.28338753	1.39907721	-0.00100026
C	-4.66616673	-2.18504411	1.25900228
H	-4.86750861	-1.60519559	2.16522510
H	-3.61818679	-2.50236797	1.27423833
H	-5.29482796	-3.08219900	1.27584158
C	-4.66604004	-2.18550525	-1.26002639
H	-4.86727258	-1.60598461	-2.16648243
H	-5.29471392	-3.08265619	-1.27660766
H	-3.61806477	-2.50286671	-1.27503626
C	1.97651102	-0.50007488	0.00033720
C	3.33144961	-2.32570313	0.00215833
C	4.14475968	-1.18563765	0.00226109
C	5.13804048	-3.60178511	0.00392588
H	5.60171081	-4.58502144	0.00461683
C	5.93899601	-2.47922137	0.00404707

H	7.01958627	-2.59804747	0.00483530
C	0.84464469	-2.65664308	0.00043894
C	0.27366917	-3.00962731	-1.22940285
C	-0.90921983	-3.75200527	-1.20628842
H	-1.37380541	-4.04682807	-2.14379444
C	-1.49492719	-4.12313511	-0.00073079
H	-2.41198757	-4.70665155	-0.00118489
C	-0.91088176	-3.75127339	1.20540833
H	-1.37672810	-4.04556772	2.14245636
C	0.27192892	-3.00881288	1.22970115
C	0.90289222	-2.60071796	-2.54360699
H	1.80596772	-2.02589870	-2.31700890
C	1.33222341	-3.82700845	-3.35028842
H	1.82806578	-3.51843439	-4.27767547
H	0.47233028	-4.45137822	-3.62072234
H	2.03202368	-4.43939428	-2.77249850
C	-0.03104830	-1.68684817	-3.33915663
H	-0.28682447	-0.79262842	-2.75846294
H	-0.96432871	-2.19728793	-3.60568090
H	0.45160905	-1.36258504	-4.26831845
C	0.89932962	-2.59909274	2.54451415
H	1.80217766	-2.02356502	2.31878094
C	1.32875409	-3.82494102	3.35183692
H	1.82334880	-3.51587306	4.27972643
H	2.02962829	-4.43688744	2.77488322
H	0.46901260	-4.44990365	3.62139209
C	-0.03621564	-1.68586925	3.33891641
H	0.44523662	-1.36100802	4.26849460
H	-0.96932052	-2.19704106	3.60464378
H	-0.29220406	-0.79197780	2.75781339
C	3.63095347	1.27605480	0.00040822
C	3.75708966	1.93616332	1.22998498
C	4.03346644	3.30496538	1.20496425
H	4.14054466	3.84571692	2.14163395
C	4.17338157	3.98254077	-0.00138993
H	4.39097937	5.04745800	-0.00209786
C	4.03788206	3.30247084	-1.20684439
H	4.14837164	3.84129212	-2.14423070
C	3.76157613	1.93363295	-1.23005675
C	3.60448147	1.20059405	2.54402118
H	3.30817442	0.17173125	2.31767015
C	2.49830287	1.81222238	3.40462541
H	2.73261011	2.84303971	3.69534239
H	2.36531885	1.22895126	4.32305033
H	1.54381104	1.82103535	2.86525558
C	4.94148268	1.13943743	3.28517840
H	5.30225120	2.14367709	3.53811034
H	5.69958997	0.64841896	2.66664365
H	4.83636452	0.57331691	4.21785733
C	3.61355280	1.19536645	-2.54310684
H	3.31638886	0.16698635	-2.31571002
H	2.74586289	2.83549860	-3.70087681
C	2.51043388	1.80531843	-3.40881235
H	1.55409108	1.81534221	-2.87276541
H	2.38058945	1.22014883	-4.32647994
C	4.95313621	1.13248964	-3.27943588
H	5.31520552	2.13617422	-3.53271186
H	5.70878914	0.64208396	-2.65742946
H	4.85104833	0.56491029	-4.21156739

References

[1] R. Tang, S. Xu, T.-L. Lam, et al., *Angew. Chem. Int. Ed.* **2022**, 61, e202203982.
[2] R. Tang, S. Xu, L. Du, et al., *Adv. Opt. Mat.* **2023**, 11, 2300950.
[3] R. Tang, S. Xu, G. Cheng, et al., *Nat. Commun.* **2025**, 16, 7776.
[4] J.-G. Yang, X.-F. Song, J. Wang, et al., *Chem. - Eur. J.* **2021**, 27, 17834.
[5] C. N. Muniz, J. Schaab, A. Razgoniaev, et al., *J. Am. Chem. Soc.* **2022**, 144, 17916.
[6] F. Chotard, V. Sivchik, M. Linnolahti, et al., *Chem. Mater.* **2020**, 32, 6114.
[7] R. Hamze, S. Shi, S. C. Kapper, et al., *J. Am. Chem. Soc.* **2019**, 141, 8616.
[8] T.-y. Li, J. Schaab, P. I. Djurovich, M. E. Thompson, *J. Mater. Chem. C* **2022**, 10, 4674.
[9] S. Shi, M. C. Jung, C. Coburn, et al., *J. Am. Chem. Soc.* **2019**, 141, 3576.
[10] H.-J. Wang, Y. Liu, B. Yu, et al., *Angew. Chem. Int. Ed.* **2023**, 62, e202217195.
[11] S. Avula, B. H. Jhun, U. Jo, et al., *Adv. Sci.* **2024**, 11, 2305745.
[12] M. J. Frisch, G. W. Trucks, H. B. Schlegel, et al., Gaussian Inc. Wallingford CT 2016.
[13] J. P. Perdew, M. Ernzerhof, K. Burke, *J. Chem. Phys.* **1996**, 105, 9982.
[14] C. Adamo, V. Barone, *J. Chem. Phys.* **1999**, 110, 6158.
[15] S. Grimme, J. Antony, S. Ehrlich, H. Krieg, *J. Chem. Phys.* **2010**, 132, 154104.
[16] S. Grimme, S. Ehrlich, L. Goerigk, *J. Comput. Chem.* **2011**, 32, 1456.
[17] L. Radom, P. C. Hariharan, J. A. Pople, P. V. R. Schleyer, *J. Am. Chem. Soc.* **1973**, 95, 6531.
[18] M. J. Frisch, J. A. Pople, J. S. Binkley, *J. Chem. Phys.* **1984**, 80, 3265.
[19] M. Cossi, G. Scalmani, N. Rega, V. Barone, *J. Chem. Phys.* **2002**, 117, 43.
[20] E. Glendening, A. Reed, J. Carpenter, F. Weinhold, *University of Wisconsin, Madison* **1998**, 65.
[21] E. D. Glendening, C. R. Landis, F. Weinhold, *J. Comput. Chem.* **2013**, 34, 1429.
[22] M. P. Mitoraj, A. Michalak, T. Ziegler, *J. Chem. Theory Comput.* **2009**, 5, 962.
[23] G. te Velde, F. M. Bickelhaupt, E. J. Baerends, et al., *J. Comput. Chem.* **2001**, 22, 931.
[24] C. Fonseca Guerra, J. G. Snijders, G. te Velde, E. J. Baerends, *Theor. Chem. Acc.* **1998**, 99, 391.
[25] ADF 2019, SCM, Theoretical Chemistry, Vrije Universiteit, Amsterdam, The Netherlands, **2019**.
[26] E. van Lenthe, E. J. Baerends, J. G. Snijders, *J. Chem. Phys.* **1993**, 99, 4597.
[27] E. van Lenthe, E. J. Baerends, J. G. Snijders, *J. Chem. Phys.* **1994**, 101, 9783.
[28] E. van Lenthe, A. Ehlers, E.-J. Baerends, *J. Chem. Phys.* **1999**, 110, 8943.
[29] A. Klamt, G. Schüürmann, *J. Chem. Soc., Perkin Trans. 2* **1993**, 799.
[30] A. Klamt, *J. Phys. Chem.* **1995**, 99, 2224.
[31] M. Menéndez, R. Álvarez Boto, E. Francisco, Á. Martín Pendás, *J. Comput. Chem.* **2015**, 36, 833.

[32] S. Dapprich, G. Frenking, *J. Phys. Chem.* **1995**, 99, 9352.
[33] M. Xiao, T. Lu, *J. Adv. Phys. Chem.* **2015**, 04, 111.
[34] E. W. Stout, P. Politzer, *Theor. Chem. Acc.* **1968**, 12, 379.
[35] T. Lu, F. Chen, *J. Comput. Chem.* **2012**, 33, 580.
[36] W. Humphrey, A. Dalke, K. Schulten, *J. Mol. Graph.* **1996**, 14, 33.
[37] L. Falivene, Z. Cao, A. Petta, et al., *Nat. Chem.* **2019**, 11, 872.
[38] M. Nooijen, R. J. Bartlett, *J. Chem. Phys.* **1997**, 106, 6441.
[39] M. Nooijen, R. J. Bartlett, *J. Chem. Phys.* **1997**, 107, 6812.
[40] C. Riplinger, F. Neese, *J. Chem. Phys.* **2013**, 138, 034106.
[41] C. Riplinger, B. Sandhoefer, A. Hansen, F. Neese, *J. Chem. Phys.* **2013**, 139.
[42] A. K. Dutta, F. Neese, R. Izsák, *J. Chem. Phys.* **2016**, 145.
[43] C. Riplinger, P. Pinski, U. Becker, et al., *J. Chem. Phys.* **2016**, 144, 024109.
[44] A. K. Dutta, M. Nooijen, F. Neese, R. Izsák, *J. Chem. Phys.* **2017**, 146.
[45] R. Berraud-Pache, F. Neese, G. Bistoni, R. Izsák, *J. Chem. Theory Comput.* **2020**, 16, 564.
[46] F. Weigend, R. Ahlrichs, *Phys. Chem. Chem. Phys.* **2005**, 7, 3297.
[47] F. Neese, *Wiley Interdiscip. Rev. Comput. Mol. Sci.* **2012**, 2, 73.
[48] F. Weigend, M. Kattannek, R. Ahlrichs, *J. Chem. Phys.* **2009**, 130, 164106.
[49] I. Lyskov, M. Kleinschmidt, C. M. Marian, *J. Chem. Phys.* **2016**, 144.
[50] C. Lee, W. Yang, R. G. Parr, *Phys. Rev. B* **1988**, 37, 785.
[51] A. D. Becke, *J. Chem. Phys.* **1993**, 98, 1372.
[52] S. Grimme, M. Waletzke, *J. Chem. Phys.* **1999**, 111, 5645.
[53] M. Kleinschmidt, C. M. Marian, M. Waletzke, S. Grimme, *J. Chem. Phys.* **2009**, 130.
[54] J. Feng, A.-P. M. Reponen, A. S. Romanov, et al., *Adv. Funct. Mater.* **2021**, 31, 2005438.
[55] P. Li, Z. Wang, S. Wang, et al., *J. Phys. Chem. C* **2021**, 125, 26770.
[56] S. Lin, Q. Ou, Y. Wang, et al., *J. Phys. Chem. Lett.* **2021**, 12, 2944.
[57] J. P. Zobel, A. M. Wernbacher, L. González, *Angew. Chem. Int. Ed.* **2023**, 62, e202217620.
[58] M. R. Silva-Junior, M. Schreiber, S. P. A. Sauer, W. Thiel, *J. Chem. Phys.* **2008**, 129.
[59] J. L. Casals-Sainz, A. Fernández-Alarcón, E. Francisco, et al., *J. Phys. Chem. A* **2020**, 124, 339.
[60] A. D. Becke, *J. Chem. Phys.* **1988**, 88, 2547.
[61] P. Pyykkö, M. Atsumi, *Chem. - Eur. J.* **2009**, 15, 186.
[62] T. Ziegler, A. Rauk, *Theor. Chem. Acc.* **1977**, 46, 1.
[63] R. F. Nalewajski, A. M. Köster, K. Jug, *Theor. Chem. Acc.* **1993**, 85, 463.
[64] R. F. Nalewajski, J. Mrozek, G. Mazur, *Can. J. Chem.* **1996**, 74, 1121.
[65] R. F. Nalewajski, J. Mrozek, A. Michalak, *Int. J. Quantum Chem.* **1997**, 61, 589.
[66] R. F. Nalewajski, *J. Math. Chem.* **2008**, 44, 802.

[67] M. Mitoraj, A. Michalak, *J. Mol. Model.* **2007**, 13, 347.
[68] A. Michalak, M. Mitoraj, T. Ziegler, *J. Phys. Chem. A* **2008**, 112, 1933.
[69] R. C. Hilborn, *Am. J. Phys.* **1982**, 50, 982.
[70] R. A. Marcus, *J. Chem. Phys.* **1984**, 81, 4494.
[71] K. Schmidt, S. Brovelli, V. Coropceanu, et al., *J. Phys. Chem. A* **2007**, 111, 10490.
[72] S. Ren, J. Harms, M. Caricato, *J. Chem. Theory Comput.* **2017**, 13, 117.
[73] J. Föller, C. M. Marian, *J. Phys. Chem. Lett.* **2017**, 8, 5643.
[74] Q. Wan, J. Yang, W.-P. To, C.-M. Che, *Proc. Natl. Acad. Sci. U. S. A.* **2021**, 118, No. e2019265118.
[75] S. Xu, Q. Wan, J. Yang, C.-M. Che, *J. Phys. Chem. Lett.* **2024**, 15, 2193.
[76] J.-G. Yang, X.-F. Song, G. Cheng, et al., *ACS Appl. Mater. Interfaces.* **2022**, 14, 13539.
